# Regression to the mean can explain saturation of geomagnetic storms

**DOI:** 10.1038/s41586-026-10757-4

**Published:** 2026-07-15

**Authors:** Nithin Sivadas, David Sibeck, Varsha Subramanyan, Maria-Theresia Walach, Dogacan Su Ozturk, Banafsheh Ferdousi, Bayane Michotte de Welle

**Affiliations:** 1 Department of Physics, The Catholic University of America, Washington, DC USA; 2 NASA Goddard Space Flight Center, Greenbelt, MD USA; 3 Department of Physics, University of Illinois Urbana-Champaign, Champaign, IL USA; 4 School of Physics and Astronomy, Lancaster University, Lancaster, UK; 5 Department of Physics, Geophysical Institute, University of Alaska, Fairbanks, AK USA; 6 Air Force Research Laboratory, Albuquerque, NM USA

**Keywords:** Magnetospheric physics, Space physics, Statistics

## Abstract

Extreme space weather events on Earth occur during intervals of strong solar wind driving^1^. The solar wind drives plasma convection and currents in the near-Earth space environment^2^. For low values of the driver, the Earth’s response is linear, estimated by parameters such as the polar cap index based on ground magnetometer activity^3^. Curiously, for extreme solar wind driving, the Earth’s response appears not to increase beyond a saturation limit^4^. Theorists have advanced a host of explanations for this saturation effect, but there is no consensus^5^. Here we demonstrate that this saturation is a manifestation of the regression to the mean effect^6^ arising from random uncertainty in the timing and magnitude of solar wind measurements. Our results reveal that data analysis underpinning the saturation theories is nonlinearly biased, thereby challenging the validity of the theories. Correcting for the uncertainties reveals that the Earth’s response to solar wind driving is linear throughout, and that the impact of extreme geomagnetic storms can be twice as large as previously thought. We show that regression to the mean is a fundamental property of the relationship between measurement and the truth, where the truth corresponding to the measurement is closer to the mean. This effect is particularly pronounced for uncertain measurements of extreme values and is likely to manifest across various fields, from extreme climate studies to chronic medical pain.

## Abstract (web summary)

The saturation of geomagnetic activity with solar wind strength can be explained by a regression to the mean effect, suggesting that the impact of extreme geomagnetic storms can be twice as large as previously thought

## Main

A fundamental challenge in science is understanding how systems behave under rare and severe conditions. However, a critical, often-overlooked factor complicates this pursuit: uncertainty in measuring the conditions. Here we quantitatively demonstrate that random error can systematically distort our inferences of the system’s response, especially for extreme events. We resolve a long-standing puzzle in space physics—the apparent saturation of geomagnetic activity during extreme space weather^1^. We encourage readers to consult the supplementary sections in the sequence they are first referenced in the text.

## Saturation of geomagnetic activity

**Fig. 1:**
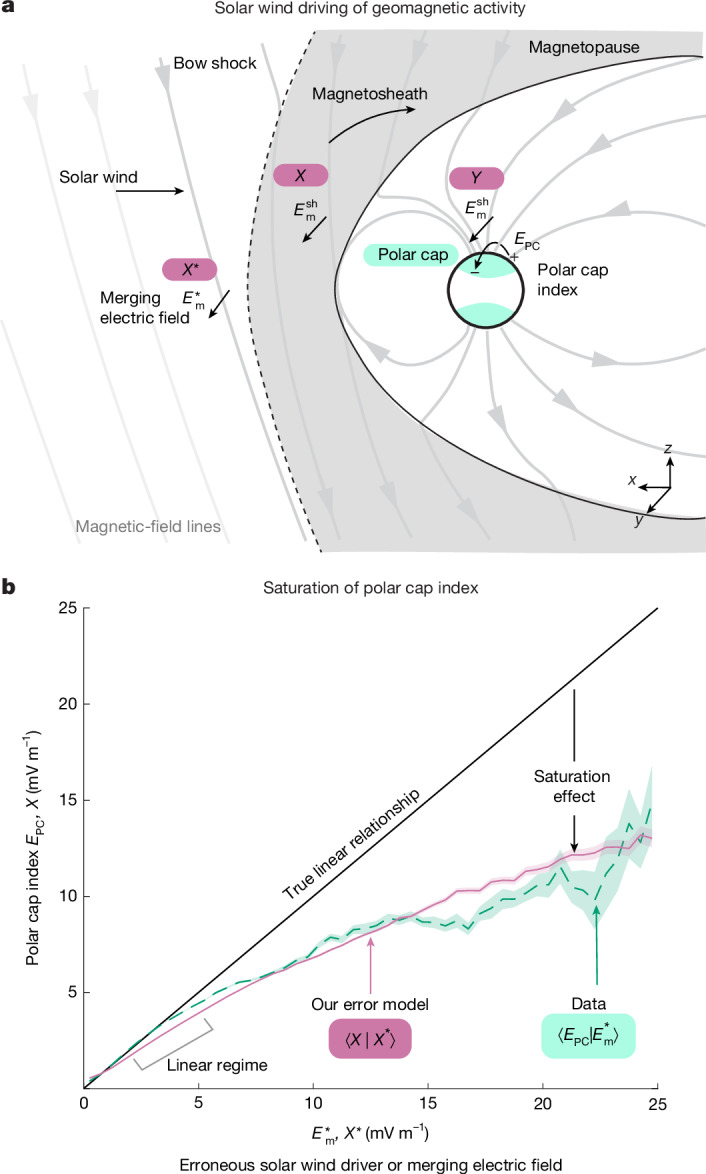
Saturation of geomagnetic activity with solar wind driving. **a**, Solar wind merging electric field (${E}_{{\rm{m}}}^{* }$) measured upstream of the bow-shock, is transformed to the shocked solar wind driver in the magnetosheath (${E}_{{\rm{m}}}^{\mathrm{sh}}$) downstream of the bow-shock, which corresponds to the dawn–dusk electric field that maps to the polar ionosphere (*E*_PC_). **b**, Observations from 1995 to 2019 (green curve) show that on average the PCI (proportional to the dawn–dusk electric field *E*_PC_) increases linearly at low values of solar wind driving, but deviates from linearity and saturates at large values of driving. The purple curve shows that the error model developed in this work predicts the same saturation effect owing to uncertainty in the solar wind driver instead of any physical mechanism. The transparent shaded area for both curves shows the 95% confidence interval.

Extreme geomagnetic storms with strong solar wind driving can increase the electric current strengths in the near-Earth space environment^7,8^, causing power outages, disruptions in satellite communication and ozone loss in the polar ionosphere^9,10^. The solar wind slows down through a bow-shock upstream of the Earth’s magnetosphere and drives plasma convection in the polar cap ionosphere (Fig. 1a and Supplementary Discussion 1a). Early observational studies showed that the cross-polar cap index (PCI), a measure of geomagnetic activity, increases linearly on average with the merging electric field (${E}_{{\rm{m}}}^{* }$), a measure of the solar wind driving^3^ (see Extended Data Table 2 for description of symbols). The PCI is estimated by a polar magnetometer from the ground in each hemisphere and is proportional to the cross-polar cap potential^11,12^, whereas ${E}_{{\rm{m}}}^{* }$ is measured from a spacecraft in the solar wind far upstream of Earth. As more measurements of rare and extreme solar wind driving accumulated through the years, surprisingly, there appeared to be an upper limit to the cross-polar cap potential beyond which increasing solar wind driving led to no increase in the potential (Fig. 1b and Extended Data Fig. 6). This inference from later measurements led to the emergence of ten different theories attempting to explain the saturation phenomenon^4,5^ (Extended Data Table 1). However, here we argue that there is no statistical evidence for the saturation of the geomagnetic response to strong solar wind driving, and its appearance in measurements is a result of uncertainty in solar-wind-driver measurements.

## Uncertainty and the problem of definition

Solar wind measurements are made mainly far upstream from the dayside reconnection site. They are, hence, an uncertain estimate of the actual solar wind driver close to the reconnection site in the magnetosheath, transformed during its journey, and influenced by local plasma and field conditions. Uncertainties in measurements are frequently attributed to instrumental error, but a surprising fact is that uncertainties can also result from the assumptions made while inferring data from measurements. This uncertainty stems from a ‘problem of definition’^13^ (for example, Supplementary Discussion 1b).

This problem of definition arises when using solar wind measurements made at the Lagrange point L1, approximately 230 Earth radii (*R*_E_) upstream of the reconnection site, as an estimate for the local magnetosheath conditions that drive the coupling of energy and plasma into the magnetosphere. The shocked solar wind driving (${E}_{{\rm{m}}}^{\mathrm{sh}}$) near the reconnection site differs from what is measured at L1 (refs. ^14,15^) owing to (1) variability in propagation times from the L1 point to the Earth, (2) spatial variability of the solar wind structure^16^, and (3) evolution of solar wind along that path owing to a variety of plasma processes changing the essential parameters influencing the reconnection rate, such as the local magnetic-field direction.

Uncertainties can be of two types: bias or unbiased random error. A bias is a consistent deviation of the true value from the measurement itself. Physical theories that explain relations between physical parameters or their respective measurements almost always attempt to explain consistent and deterministic deviations between them, excluding the random fluctuations in their values. By contrast, the unbiased random error often consists of these random fluctuations and is the random deviation of the measurements from the true value. Physical theories in space science generally explain average changes, not random fluctuations. Hence, an unknown bias can be misinterpreted as a physical effect (that is, an average change) when a theory is constructed to explain the bias. In short, physical theories intend to explain physical biases, and if we ignore bias stemming from random measurement error, we may mistake it for a physical phenomenon.

## Regression to the mean and the more-probable

A common goal of scientific analysis is to uncover the relations between two or more physical processes. Random errors in the measurement of these processes pose a difficulty in inferring relations between them, for example, in our case, the relation between solar wind driver and the geomagnetic response. However, it is widely believed that averaging the measurements can remove the effects of random error, as the underestimates will cancel out the overestimates. Supplementary Discussion 1c shows that this widespread belief is only partially true.

A popular technique to find the relations between two or more physical processes is regression analysis of their measurements. Here the uncertainty in the dependent variable is averaged away, but the uncertainty in the independent (or conditional) variable manifests as bias in the relation inferred between the variables, known as regression bias^17^. If the regression bias is nonlinear, it can create an appearance of saturation of the dependent variable for increasing values of the independent variable. This statistical phenomenon is a result of a regression to the mean^6,18^.

**Extended Data Fig. 1:**
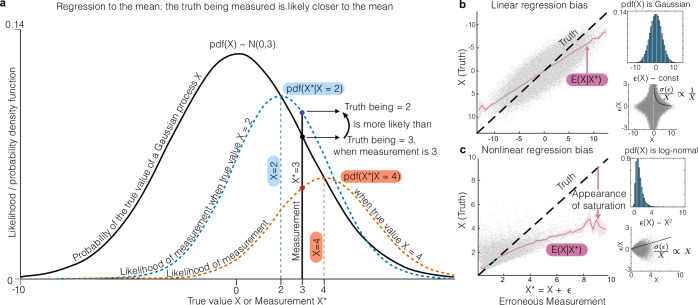
Explaining regression to the mean effect using probability theory and regression bias. **a**, For a physical parameter *X* which has an underlying probability distribution, some values are more likely than others, and hence when the measurement is X* = 3 the corresponding true value is more likely to be *X* = 2 than *X* = 3. **b**, The expected true value of a Gaussian process *X* has a linear bias when measurement is X* = *X* + *ϵ* with a constant random error $({\epsilon })$. **c**, The expected true value of a log-normal process *X* has a nonlinear bias when the measurement X* has random error *ϵ*(*X*) that varies with the strength of *X*. The transparent shaded area for regression curves shows a 95% confidence interval. Figure adapted from ref. ^18^ under a Creative Commons licence CC BY 4.0.

Supplementary Discussion 1d explains the regression to the mean effect from five different yet consistent vantage points. For instance, in the literature, regression to the mean is commonly understood as a statistical phenomenon where extreme measurements are more likely followed by measurements closer to the mean^6^. However, we argue that the phenomenon has a more fundamental origin. As discussed above, it manifests as regression bias in the relation inferred between two measurements^18^ (Extended Data Figs. 1b and 5). Most importantly, it is also a probabilistic effect where the true value being measured is more likely to be closer to the mean of the stochastic process than the measurement^18^ (Extended Data Fig. 1a).

The regression to the mean is not only a statistical phenomenon, although it commonly appears as one. It is not a result of a particular statistical method of inferring relations between parameters, although the particular method will be affected by it. At its core, regression to the mean is a fundamental property of the relation between the true value of a stochastic process and its measurement (Supplementary Discussion 1e).

The essence of regression to the mean is that the truth being measured regresses towards the mean, and this property of the relation between the truth and measurement manifests in many different forms depending on what one uses the measurements for. It is a fundamental logical consequence of the relation between the true value and its measurement, implying that for even a single measurement, the corresponding true value is more likely closer to the mean of the stochastic process. For a stochastic process with a general probability distribution, the effect is more aptly named the ‘regression to the more-probable’.

## Solar wind uncertainty and error model

Owing to regression to the mean, when an extreme value of the solar wind driver is measured at the L1 Lagrange point, the true driver value near the Earth is more likely smaller and closer to the mean. Therefore, the geomagnetic response to this smaller driver will also be smaller. When we do not account for this regression to the mean, as in most previous studies, the smaller geomagnetic response will be wrongly associated with the extreme value of the solar wind driver measured at L1. The more extreme the value of the measured solar wind driver, the greater the mismatch between the true driver and the measured value. As a result, the cross-polar cap potential response to the measured uncertain solar wind driver deviates from linearity during the rare and extreme values and hence, appears to saturate.

**Fig. 2:**
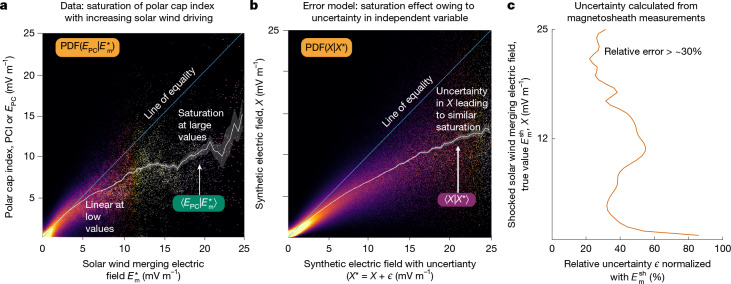
Regression bias predicted by the error model matches saturation effect from data. **a**, Conditional probability density function of 25 years of measurements $\mathrm{PCI}|{E}_{{\rm{m}}}^{* }\approx {E}_{\mathrm{PC}}|{E}_{{\rm{m}}}^{* }$. **b**, Conditional probability density function (PDF) reproduced by the error model PDF(*X*|X*), which is very similar to the measurements presented in **a**. The transparent shaded area for both regression curves shows the 95% confidence interval. **c**, Quantified relative uncertainty *ϵ*(*X*)/*X* owing to random variations in solar wind driver measured at L1 (${E}_{{\rm{m}}}^{* }$, X*) for a measured value of the shocked solar wind driver (${E}_{{\rm{m}}}^{\mathrm{sh}}$, *X*).

**Extended Data Fig. 2:**
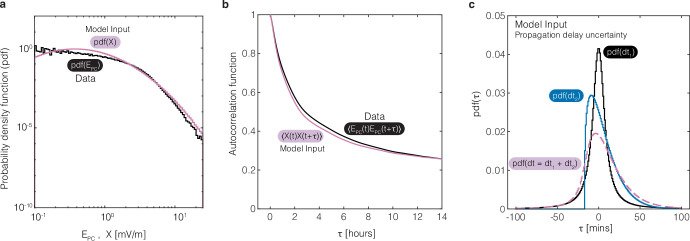
Stochastic properties of inputs to the error model. **a**, The black line shows the PDF of the polar cap index *E*_PC_ from 1995 to 2019. And the purple line shows the log-normal PDF of the model random variable *X* that best fits the PDF of *E*_PC_. **b**, The black dashed line shows the autocorrelation function of the polar cap index *E*_PC_ averaged over data from 1995 to 2019. And the purple line shows the autocorrelation function of the model random variable *X* that closely follows the ACF of *E*_PC_. **c**, The black line shows the uncertainty in the propagation delay from L1 to the bow-shock nose (*dt*_1_). The blue line shows the uncertainty in the propagation delay from the bow-shock nose to the polar cap ionosphere (*dt*_2_). The purple line shows the total uncertainty in the propagation delay from L1 to the polar cap (*dt*_1_ *+ dt*_2_).

To quantitatively test this surprising argument, we construct an error model X* = *X*(*t + *d*t*) + *ϵ*, where X* is the measured ‘uncertain’ solar wind driver at L1, and *X* is the ‘true’ driving by the shocked solar wind close to the reconnection site. d*t* is the uncertainty in the solar wind propagation time, and *ϵ* is the random magnitude changes in the driver (see details in Methods and Extended Data Fig. 2). Figure 2c presents the relative uncertainty (*ϵ*(*X*)/*X*) calculated from measurements with respect to the true shocked solar wind driver in the magnetosheath (*X*). It is at least 30%, and is heteroskedastic, that is, varying with the magnitude of the true value (*X*)^19^.

**Extended Data Fig. 3:**
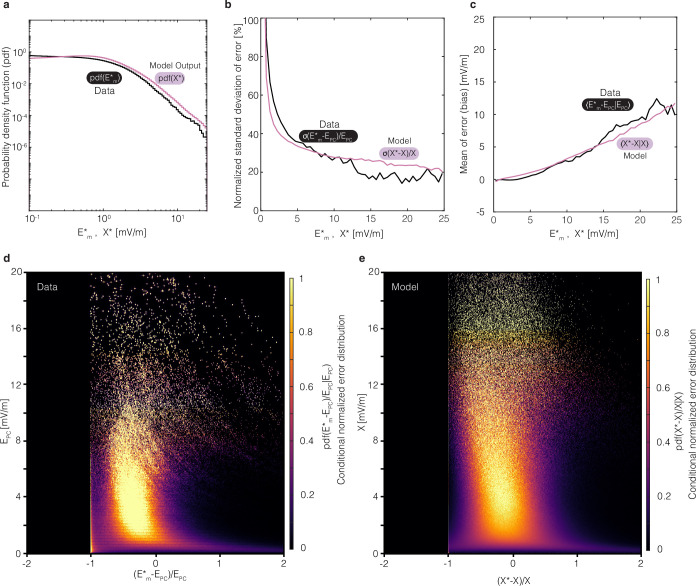
Statistics of the output of the error model. **a**, The model predicts with good correspondence the PDF of the estimate of the merging electric field propagated to the polar cap, with a probability density difference of less than ~0.1 between data and model for most values. ${E}_{{\rm{m}}}^{* }$ is data, and X* is model. **b**, The model predicts the nature of the random error between the estimate and the true value of the merging electric field projected on the ionosphere. The black line is the normalized uncertainty associated with ${E}_{{\rm{m}}}^{* }$ (data), and the purple line is the normalized uncertainty associated with X* (model). **c**, There is a nonlinear conditional bias in ${E}_{{\rm{m}}}^{* }$:⟨${E}_{{\rm{m}}}^{* }$- *E*_PC_|*E*_PC_⟩ that varies with *E*_PC_, which is reproduced very well by the model ⟨X*- *X*|*X*⟩. **d**, Conditional normalized error distribution from data, **e**, Conditional normalized error distribution from the model. Both data and model show similar trends in how the error varies with increasing strength of the true variable –a steep increase in the spread of the error from 0 to ~12 mV/m in the vertical axis, and then a decrease (or loss of power) beyond that point.

**Extended Data Fig. 4:**
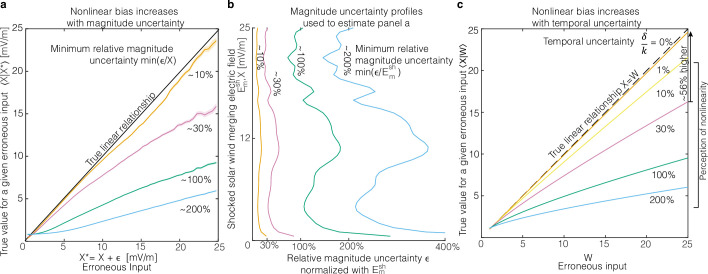
Sensitivity analysis of magnitude uncertainty. **a**, Nonlinear regression bias caused by heteroskedastic magnitude uncertainty increases with a measure of the uncertainty represented by the minimum relative magnitude uncertainty. **b**, The heteroskedastic variation of the magnitude uncertainty with the true value *X* is shown, for different values of the minimum relative magnitude uncertainty corresponding to the different regression bias curves in panel a. **c**, Nonlinear bias caused by uncertainty in time increases with a measure of the uncertainty represented by the dimensionless ratio of the time uncertainty (*δ*) and the autocorrelation constant (*k*).

**Extended Data Fig. 5:**
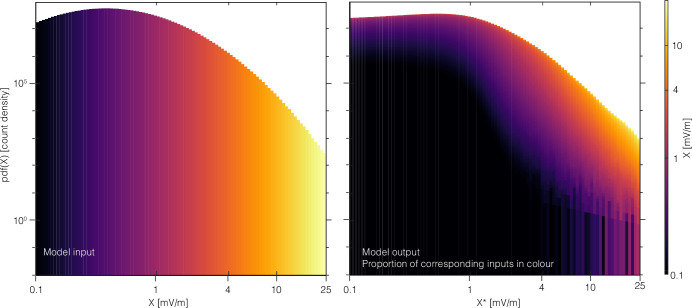
Another graphical explanation of the regression to the mean effect. (Left) Probability distribution function of the true variable *X*, with each bin coloured uniquely. (Right) The probability distribution function of the inaccurate estimate of *X*, that is, X* with the colour representing the magnitude of *X*. The amount of a particular colour per bin represents the proportion of *X* values misidentified as X*.

**Extended Data Fig. 6:**
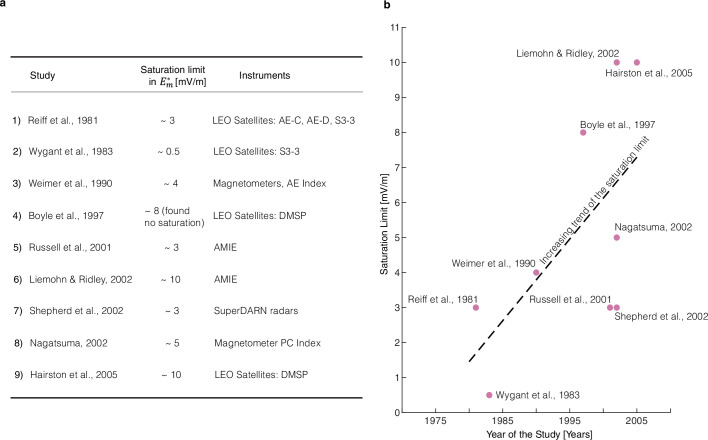
Increasing trend of the saturation limits observed by previous observational studies. **a**, Cross polar cap potential response to solar wind driving is linear up to a saturation limit, which varies depending on the observational study and the methodology they use. All except Boyle et al., 1997^47^ infer a saturation beyond the limit in E*_m_. See the studies here: 1^48^, 2^49^, 3^37^, 4^47^, 5^50^, 6^51^, 7^52^, 8^53^, 9^38^. **b**, The saturation limit of different studies presented here follows an increasing trend with the year of study (Pearson correlation coefficient ~0.61), with the latest studies including higher number of data samples.

When the error is heteroskedastic like some uncertainties related to the ‘problem of definition’, and *X* has a log-normal distribution like many solar wind parameters^20^, the likely true value for a given measurement acquires a nonlinear bias mimicking a saturation^18^ of *X* with increasing X* (Extended Data Fig. 1c). We prove analytically that such a nonlinear bias also increases with a dimensionless measure of uncertainty in time (Supplementary Discussion 1f, Extended Data Fig. 4c and Supplementary Methods 2c). By incorporating the calculated uncertainties into the Monte Carlo error model, it solves for X* and predicts an appearance of saturation of *X* given X* (Fig. 2b). This saturated curve, and the conditional probability distribution of *X* given X*, matches surprisingly well with the apparent saturation in the data (Fig. 2a). The precondition of this nonlinear saturation relation between PCI and ${E}_{{\rm{m}}}^{* }$ is the heteroskedastic nature of the error in ${E}_{{\rm{m}}}^{* }$, the propagation time uncertainty and the fact that the solar wind driver is a log-normal process with a particular autocorrelation time constant (Supplementary Discussion 1g). The error model is validated against data and robust to changes in the inputs (Supplementary Methods 2b and Extended Data Fig. 3).

## Saturation of the cross-polar cap potential explained

This surprising result from the error model shows that uncertainty in the solar wind driver leads to a biased inference that the geomagnetic response saturates with solar wind driving. This apparent saturation arises when comparing the solar wind driver at L1 to the PCI, which is proportional to the cross-polar cap potential^11^. Hence, there is currently no statistical evidence to suggest an upper limit to the energy transferred from the solar wind to the polar ionosphere. The ten prevailing theories and models of polar cap potential saturation were developed to explain this biased inference from existing erroneous measurements. As a result, these theories have not been tested or validated against corrected and unbiased data. We maintain that the original assumption—that the solar wind magnetic-field lines connect directly to the polar regions, driving ionospheric convection, and therefore are on average linearly related to the magnetic fluctuations in ground magnetometers and thus the PCI—holds true^21^.

## Correcting the effect of uncertainties

One way to address the effect of uncertainties is to reduce them. For instance, by relying on spacecraft measurements closer to Earth to estimate the correct driver. However, uncertainties are bound to exist in every measurement, and the problem of definition will almost always exist, so the method of reducing uncertainties is unlikely to eliminate them. Hence, alternatively, we statistically quantify the uncertainties, predict their effect and offset them from our inference. This method is achievable, albeit challenging, through scientific investigation of the relations between the truth and measurement, and the assumptions that underlie our inferences of the measurements.

Once we calculate uncertainties and develop an error model, we can derive the nonlinear bias in the regression analysis. We can subtract this bias (*b*) from the erroneous independent variable using ‘regression calibration’^19^ (Methods). Applying this bias correction to the solar wind driver (${E}_{{\rm{m}}}^{{\rm{c}}}={E}_{{\rm{m}}}^{* }-b$) reveals a surprisingly linear relationship with the PCI up to 15 mV m^−1^—without assuming any linearity or nonlinearity in the underlying relation between ${E}_{{\rm{m}}}^{\mathrm{sh}}$ and PCI, or their error model counterparts.

**Fig. 3:**
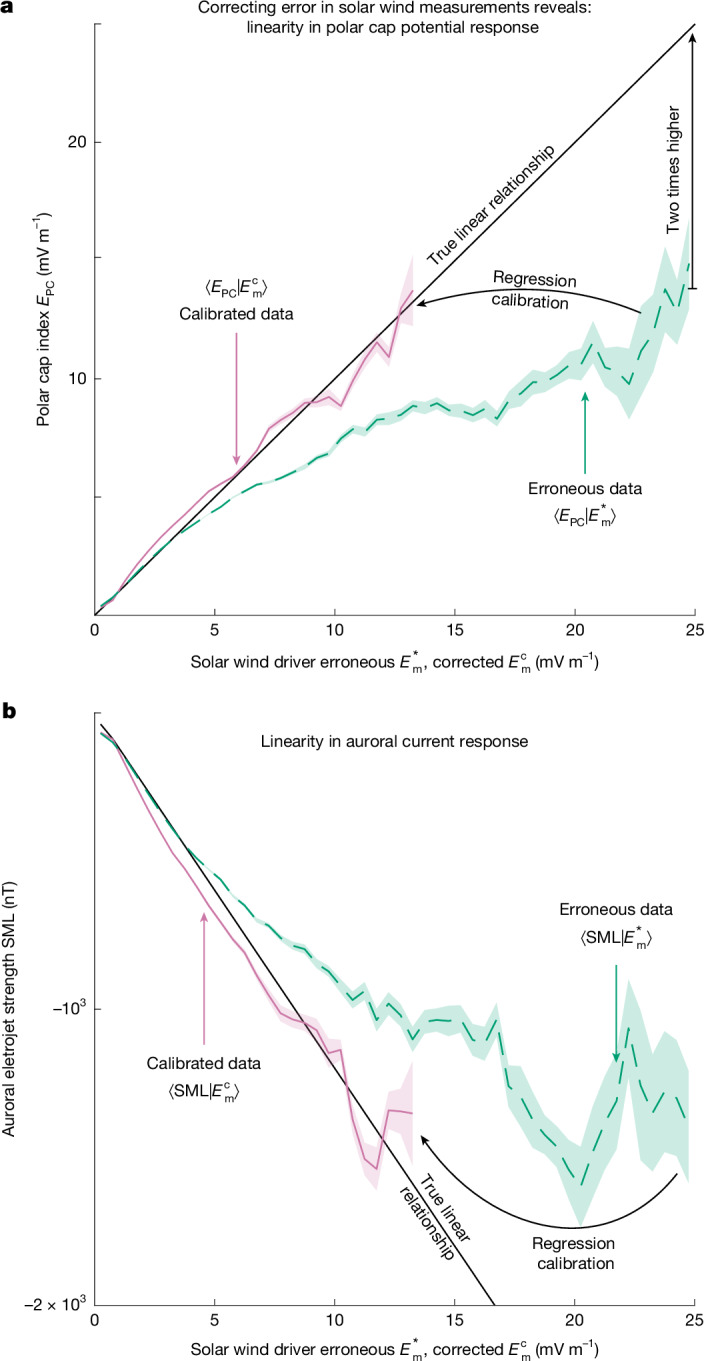
Correcting the effect of random errors in solar wind driver values reveals a linear geomagnetic response. **a**, The PCI varies linearly with solar wind driving on average (purple curve), after correcting the regression bias in the erroneous solar wind driver values (green curve). **b**, The westward auroral electrojet strength (SML) also varies linearly with solar wind driving (${E}_{{\rm{m}}}^{{\rm{c}}}$) on average (purple curve) after the same regression bias correction applied to the erroneous solar wind driver values (${E}_{{\rm{m}}}^{* }$) (green curve). The transparent shaded area for all the curves shows the 95% confidence interval.

Figure 3a shows the saturating green curve from erroneous data transforming into the linear purple curve after regression calibration. This demonstrates that the true geomagnetic response is linear, and that the apparent saturation of the geomagnetic response results from uncertainty in the solar wind driver and the regression to the mean effect. Beyond 15 mV m^−1^, insufficient data prevent conclusions about the shape of the relation between the driver and response. When the same calibration is applied to other measures of geomagnetic activity, such as the westward auroral electrojet (Fig. 3b and Supplementary Discussion 1h), its relation with the driver becomes linear, providing further evidence that geomagnetic response to solar wind driving does not saturate. This implies that we cannot rely on the magnetosphere to dampen the effects of extreme geomagnetic storms.

It is this linear relationship between the driver and response that a new (or old) physical theory of saturation needs to explain and validate its predictions against. The development of multiple saturation theories can be attributed to different inferences from data. Under-sampling bias, often confused with regression to the mean, leads to different saturation limits of the geomagnetic response depending on the sample size of a study (Supplementary Discussion 1i). Further evidence that saturation results from uncertainty in the solar wind driver is hiding in plain sight in the space science literature (Supplementary Discussion 1j).

## Conclusion and generalizability

When correctly interpreted, measurements reveal a linear rather than a saturated relation of geomagnetic response to the solar wind driver on average. Thus, extreme space weather could impact Earth far more than previously thought (twice the impact at solar wind strengths extrapolated to approximately 25 mV m^−1^). Although saturation at unobserved solar wind strengths remain possible, there is no statistical evidence for this so far. Our result is not an alternative theory competing with the existing theories of saturation (Extended Data Table 1), but rather a demonstration that available data do not show evidence for the saturation of geomagnetic response. Hence, it challenges the foundation of existing physical theories of saturation and highlights the need to revisit and validate them with calibrated solar wind driver estimates.

In any field where uncertainty in the driver of a system is significant, and its response to extreme and rare instances of driving are of interest, the regression to the mean effect can lead to the underestimation of the system’s response to extreme drivers. For example, this effect may have a role in climate models, which may underestimate extreme climate events such as heatwaves^22^, or the impact of strong earthquakes away from their epicentre, or severe medical symptoms and their resolution, such as the effectiveness of chronic-pain treatments^23^. In such fields, biases and even nonlinear biases may hide in plain sight and be misunderstood as a physical process.

As artificial intelligence or machine-learning models, which fall under the umbrella of nonlinear, non-parametric regression analysis, become popular within physics and other fields with large quantities of measurements, the nonlinear bias stemming from heteroskedastic uncertainty or temporal uncertainty is probably present. They may affect prediction and any physical inferences drawn from the model outputs, and hence we urge caution in their use and call for further study of how regression to the mean influences these models.

The insight that regression to the mean is fundamental to the relation between the truth and its measurement implies that the effect will manifest in different forms within different methods of inference. Hence, all researchers in the fields that attempt to use measurements to infer relations between the physical processes they represent need to be aware of the essence and impact of the logic of regression to the mean. Ignoring this effect can lead researchers and entire fields of enquiry down long, winding paths that deviate from the truth.

## Methods

The most common symbols used are explained in the Extended Data Table 2.

### Solar wind measurement uncertainty

There are several solar wind coupling functions that attempt to quantify the driving, one important one is the solar wind merging electric field or merging geoeffective field ${E}_{{\rm{m}}}^{* }$ (refs. ^4,24^).

1.1 $${E}_{{\rm{m}}}^{* }={V}_{\mathrm{sw}}{B}_{{\rm{T}},\mathrm{sw}}{\sin }^{2}({\theta }_{\mathrm{sw}}/2)$$

This field is also known as the Kan–Lee electric field and is calculated from solar wind parameters at Lagrange point L1 alone^24^. In equation 1.1, *V*_sw_ is the solar wind speed in km s^−1^, ${B}_{{\rm{T}},\mathrm{sw}}={({B}_{y}^{2}+{B}_{z}^{2})}^{1/2}$ is the transverse magnitude of the interplanetary magnetic field (IMF) in nT, and *θ*_sw_ = tan^−1^(*B*_*y*_/*B*_*z*_) is the transverse IMF clock angle in radians. *E*_m_ is a positive-valued quantity with the units of the electric field (mV m^−1^), reflecting the assumption that energy flows only from the solar wind to the magnetosphere. The above quantities are measured in geocentric solar magnetospheric (GSM) coordinates using spacecraft orbiting the L1 point approximately 230 *R*_E_ upstream from Earth. The GSM coordinates are convenient for studying the effects of the IMF components on magnetospheric and ionospheric phenomena. Its *x* axis points towards the Sun, and the *z* axis is the projection of Earth’s magnetic dipole axis onto the plane perpendicular to the *x* axis. For a purely southwards IMF, the merging electric field ${E}_{{\rm{m}}}^{* }$ is equal to the solar wind electric field *E*_sw_ = *V*_*x*,sw_*B*_*z*_, where *V*_*x*,sw_ is a component of the solar wind velocity along the *x* direction.

The dawn–dusk portion of the shocked solar wind convection electric field maps along the equipotential magnetic-field lines and drives the plasma convection down in the polar cap ionosphere. The convection corresponds to an electric field across the polar cap in the rest-frame of the Earth (*E*_PC_). The PCI is a measure of this field and part of the energy input into the Earth’s magnetosphere. The index has gone through many iterations over the past 50 years, but it is essentially the maximum amplitude of variations observed from magnetometers near the South and North Poles^3,25,26^. The current version of the index is scaled on a statistical basis of magnetic variations to the merging electric field, such that the index is highly correlated to the merging electric field. This makes the PCI independent of daily and seasonal variations and the local ionospheric properties. PCN is the index derived from the magnetometer in the northern polar cap and PCS is derived from the southern polar cap. We use the PCI as a proxy for *E*_PC_ (that is, PCI ≈ *E*_PC_), as it combines both indices to provide a positive-valued index, which is more accurate than the individual indices^26^. PCI = 0.5(PCS + PCN), with a condition that negative values of either PCS or PCN are set to 0. PCI or *E*_PC_ also has the units of the electric field, and several statistical analyses in the literature reveal that it is linearly proportional on average to the electric potential across the polar cap, called the cross-polar cap potential^11,27^. Fluctuations in PCI can occur owing to nightside magnetosphere processes^28^, but the above relation is still maintained on average.

The Kan–Lee electric field ${E}_{{\rm{m}}}^{* }$ is only an approximation of the true driver of the magnetosphere, that is, the shocked solar wind plasma ${E}_{{\rm{m}}}^{\mathrm{sh}}={V}_{\mathrm{sh}}{B}_{\mathrm{sh}}{\sin }^{2}({\theta }^{{\prime} }/2)$, the value of which is not easily available to us (Supplementary Methods 2a). This leads to the uncertainty and nonlinear regression bias discussed in this work. Instead, we have only an erroneous estimate of the true coupling function approximately propagated to the polar cap, ${E}_{{\rm{m}}}^{* }$. Hence, we need to reinterpret the literature as saying that low values of the erroneous estimate of the solar wind strengths ${E}_{{\rm{m}}}^{* }$ (not ${E}_{{\rm{m}}}^{\mathrm{sh}}$) correlate linearly with the polar cap potential. And at high values of ${E}_{{\rm{m}}}^{* }$, the polar cap potential saturates.

We calculate ${E}_{{\rm{m}}}^{* }$ by using WIND satellite measurements published in the OMNIWeb database^29^. The database provides the values corrected for the propagation delay of the solar wind from L1 to the bow-shock nose^30–32^. Then as frequently done, we apply a further correction of a constant delay of about 17 min to account for the propagation delay from the nose to the polar cap ionosphere^16,33^. Previous literature that discusses the polar cap potential saturation problem estimates ${E}_{{\rm{m}}}^{* }$ similarly. We use the WIND data from 1995 to 2019 and the polar cap indices from the same time range. Both data are 1-min averages; larger time averages will lead to additional uncertainties^34^. We only use data samples when both WIND measurements and polar cap indices are available.

${E}_{{\rm{m}}}^{* }$ can differ from the shocked solar wind driver in the magnetosheath ${E}_{{\rm{m}}}^{\mathrm{sh}}$ in two ways. One is through a consistent deterministic bias that is determined by the bow-shock that slows down the plasma. This deterministic bias is nearly zero as the tangential (and the largest) component of the electric field across the bow-shock remains unchanged. We do not concern ourselves with this deterministic bias, as it will manifest in the data as a physical effect anyway. However, ${E}_{{\rm{m}}}^{* }$ can also differ randomly from the true solar wind driver ${E}_{{\rm{m}}}^{\mathrm{sh}}$, owing to fluctuations in the plasma properties caused by physical processes between L1 and the reconnection site, acting in random directions. These random fluctuations contribute to the uncertainty in ${E}_{{\rm{m}}}^{* }$ when we use it as a proxy for the true driver ${E}_{{\rm{m}}}^{\mathrm{sh}}$. This random error is concerning, as it does not average away, and manifests as a regression bias in data analysis and is easily confused as a physical effect, hence we calculate and correct for it.

We categorize the random uncertainty in ${E}_{{\rm{m}}}^{* }$ relative to the true value ${E}_{{\rm{m}}}^{\mathrm{sh}}$ into three primary sources.

1. Uncertainty in propagation delay of the solar wind from L1 to the bow-shock nose (d*t*_1_).
2. Uncertainty in the propagation delay of the effect of solar wind forcing from bow-shock nose to the polar cap ionosphere (d*t*_2_).
3. Random variability in the shocked solar wind owing to spatial variation in the incoming solar wind and transformations of its plasma parameters during propagation through the bow-shock and the magnetosheath (*ϵ*).

Knowing the statistical distribution of the above uncertainty will allow us to construct a stochastic model of the estimate ${E}_{{\rm{m}}}^{* }$ as a function of the true driver ${E}_{{\rm{m}}}^{\mathrm{sh}}$ mapped to the polar cap ionosphere. In the following subsection, we develop a statistical error model of the estimate of the true shocked solar wind that drives the cross-polar cap convection from an estimate of its stochastic properties and uncertainty distributions. For the calculated uncertainties (Fig. 2c), the model predicts the polar cap potential saturation, which is strikingly similar to data from 25 years of observations (Figs. 1b and 2a,b).

### Statistical error model

To distinguish between data and model, we replace ${E}_{{\rm{m}}}^{\mathrm{sh}}$ with *X* when referring to the random variable corresponding to the shocked solar wind driver in the statistical model. We also replace the PCI, which is proportional to the convection electric field *E*_PC_ in the ionosphere, with a counterpart in the model *Y*_PC_. Hence, in the model, *X* is the shocked solar wind driver accurately time-shifted to the polar cap, and X* is its erroneous estimate (that is, solar wind driver measured at L1 or ${E}_{{\rm{m}}}^{* }$). We hypothesize the following statistical error model:

1.2 $${X}^{* }(t)=X(t+{\rm{d}}{t}_{1}+{\rm{d}}{t}_{2})+{\epsilon }(t)$$

We assume that X*, *X*, d*t*_1_, d*t*_2_ and *ϵ* are stochastic processes. In other words, they are each a collection of random variables in time (*t* ∈ *T*) with an associated probability distribution that determines the random value it might take at a given time *t*. Below we present our estimates of the probability distributions and autocorrelation functions of these stochastic processes. Using these estimates of *X*, d*t*_1_, d*t*_2_ and *ϵ*, we calculate X*. After which, we validate the model results by comparing them with the data ${E}_{{\rm{m}}}^{* }$.

#### Input *X*

If reconnected open-field lines in the polar cap region do not have large parallel resistances, accurate mapping of the shocked solar wind electric field onto the polar cap should result in *X*, such that *Y*_PC_ ∝ *X*. In agreement with this, in data, we see that the probability density function (PDF) of the PCI index or *E*_PC_ (and hence *Y*_PC_) and solar wind driver ${E}_{{\rm{m}}}^{* }$ are very similar, implying that the distribution of *Y*_PC_ is also similar to *X*. As a result, we assume that the PDF of *X* is similar to the PDF of *E*_PC_ (and ${E}_{{\rm{m}}}^{* }$) that we can estimate from data. It is noted that this is not surprising as *E*_PC_ is constructed to be highly correlated with ${E}_{{\rm{m}}}^{* }$, and from Kan and Lee’s arguments ${E}_{{\rm{m}}}^{* }$ is also correlated with ${E}_{{\rm{m}}}^{\mathrm{sh}}$, and hence all these parameters have similar PDFs. (Although we have measurements of ${E}_{{\rm{m}}}^{\mathrm{sh}}$ from near-Earth orbiting spacecraft, they are discontinuous and hence do not directly provide an unbiased PDF of *X*). Like many solar wind parameters, *E*_PC_ and ${E}_{{\rm{m}}}^{* }$ can be approximated to be a log-normal distribution^20^. Therefore, we assume the PDF of model input *X* to be a log-normal distribution that closely fits the PDF of *E*_PC_ from data (Extended Data Fig. 2a). We also assume that the adjacent values of *X* in time are correlated similarly to adjacent values of *E*_PC_ in time (Extended Data Fig. 2b), as fluctuations in time of shocked solar wind electric field should correlate with that of the cross-polar cap electric field in the ionosphere. In other words, we assume that in our model, *X* shares the stochastic properties of the PCI or electric field *E*_PC_. If the assumption fails, the results of the model, particularly the second-order statistics, will be inconsistent with what is observed in the data. Finally, we assume that *X* is a stationary process, that is, its probability distribution and autocorrelation function does not change with time.

#### Uncertainty in propagation delay from L1 to nose d*t*_1_

Solar wind parameters measured upstream are time-shifted to account for the delay in propagation of the wind to the bow-shock nose. Case and Wild estimate the uncertainty to be on the order of minutes^35^. On the basis of their results and consistent with other studies^36^, we assume d*t*_1_ to be an independent random process with a PDF of a Student’s *t*-distribution with shape factor 1.3, mean 0, and scale parameter approximately 8 min (Extended Data Fig. 2c and Supplementary Methods 2b). The distribution is zero mean and has a longer tail than the normal distribution.

#### Uncertainty in propagation delay from nose to polar cap d*t*_2_

Changes in the dayside shocked solar wind electric field propagate along equipotential open magnetic-field lines to the polar caps. The delay in this propagation is on average about 17 min^16^ but can vary from −5 min to 50 min^27^. Historically, researchers have used a constant propagation delay (*t*_2_) of about 10–30 min^37,38^ for this stage of propagation. However, the uncertainty of propagation time here is significant. We model the PDF of *t*_2_ as a Weibull distribution with mean approximately 17 min^16^, standard deviation approximately 25 min and shape factor 1.3 (Extended Data Fig. 2c and Supplementary Methods 2b). The Weibull distribution keeps the total delay *t*_2_ + d*t*_2_ positive and captures the broad spread in the propagation delay^16,26,27^.

#### Magnitude uncertainty *ϵ*

There are several other reasons for the magnitude of the shocked solar wind driver to be randomly different from its proxy measured at the L1. The IMF clock angle could change substantially after crossing the bow-shock, spatial variations in the solar wind can lead to a different part of the wind interacting with the Earth’s magnetosphere, and changes in the magnetospheric state or its history can lead to changes in the local plasma and field conditions in the magnetosheath. As the solar wind strength increases, the random variation in the field can increase owing to the increased spatial structuring of the solar wind, and any clock-angle variation during increased field strength can lead to larger variations in geoeffectiveness.

To estimate these random variations, we use direct evidence from a database of simultaneous measurements of plasma and field strengths in the magnetosheath. Using a gradient boost classification algorithm, measurements from near-Earth satellites such as THEMIS, MMS, DoubleStar and Cluster, are classified into solar wind, magnetosheath and magnetosphere regions^39^. Our interest is in the measurements made within the magnetosheath, close to the magnetopause in the subsolar region (|*Y*| < 5 *R*_E_ and |Z| < 5 *R*_E_). The distance from the magnetopause boundary is determined using a machine-learning-based empirical model of the boundary, which performs better than other magnetopause models available in the literature^40^. For specific values of the magnetosheath measurements (${E}_{{\rm{m}}}^{\mathrm{sh}}$), we calculate the variance in the measurements made at L1 and time-shifted to the bow-shock (${E}_{{\rm{m}}}^{* }$). This variance is an estimate of the uncertainty in the solar wind driver ${E}_{{\rm{m}}}^{* }$ for a given value of the shocked solar wind driver ${E}_{{\rm{m}}}^{\mathrm{sh}}$. The ‘magnitude uncertainty’ *ϵ* is set to the same variance, and we model it as a zero-mean Gaussian with a standard deviation that varies with the magnitude of *X* according to data. The magnitude uncertainty is not constant with the strength of the shocked solar wind driver, instead it increases until ${E}_{{\rm{m}}}^{\mathrm{sh}}\approx 12\,\mathrm{mV}\,{{\rm{m}}}^{-1}$, after which the statistics become poor and an accurate estimate of the variance becomes challenging (Supplementary Methods 2b). In this regime, we estimate the uncertainty in the solar wind driver relative to measurements of near-Earth satellites just upstream of the bow-shock (approximately 10 < *X* < 50 *R*_E_). This uncertainty will be the minimum uncertainty for values >12 mV m^−1^ (that is, a conservative estimate of the uncertainty), and this remains roughly the same with increasing shocked solar wind driver value. Figure 2c shows the relative error, *σ*(*ϵ*)/*X*, and how that varies with *X*. The heteroskedastic behaviour of this uncertainty is consistent with the observed statistical variations in the difference between solar wind driver and the PCI shown in Extended Data Fig. 3d,e. The variation in their difference increases up to a PCI of approximately 12 mV m^−1^ and then remains constant. We also assume that *ϵ* has an autocorrelation function similar to that of the difference between the observed solar wind driver ${E}_{{\rm{m}}}^{* }$ and the polar cap index PCI ≈ *E*_PC_.

Using the above estimates of uncertainties, we generate an ensemble of time series of *X*, d*t*_1_, d*t*_2_ and *ϵ*, and calculate the corresponding time series of the erroneous estimate X* using equation (1.2). The statistical properties of the model output agree remarkably with that of the data.

### Validation of error model

We validate this nonlinear error model by comparing the second-order statistics of the error model parameters with that of their counterparts in data. The error model predicts the PDF of the solar wind driver ${E}_{{\rm{m}}}^{* }$, the standard deviation of the normalized error, the conditional normalized error distribution and finally the regression bias stemming from the regression to the mean effect. This validation is the rationale for using the error model and is presented in the Supplementary Methods 2b. Here we also discuss a sensitivity analysis of the nonlinear regression bias, which increases with increasing uncertainty in the time of propagation, and with increasing uncertainty in the magnitude uncertainty *ϵ* (Extended Data Fig. 4). The analysis shows that output of the error model is robust to the input uncertainties and variance of input *X*. Supplementary Methods 2d summarizes the full computer code used.

### Correcting the error

The regression bias *b* = X* − ⟨*X*|X*⟩, which is the consistent deviation between the measurement and the likely true value given the measurement. The erroneous measurement X* can now be corrected to *X*^c^ by subtracting the bias from it *X*^c^ = X* − *b*, which simply reduces to *X*^c^ = ⟨*X*|X*⟩. Crucially, from the model output, we can calculate the conditional expectation of the true value given the erroneous estimate: *f*_r_(*x*) = ⟨*X*|X* =* x*⟩, that is, we have a statistical, functional relationship between the true value and the erroneous measurement. For the particular assumptions of uncertainties in measurement, *f*_r_(*x*) is the best estimate of the true value (*X*^c^ or ${E}_{{\rm{m}}}^{{\rm{c}}}$) given the erroneous value (X* or ${E}_{{\rm{m}}}^{* }$). Therefore, *f*_r_(*x*) can be used to statistically correct the erroneous estimate ${E}_{{\rm{m}}}^{* }$ to a most likely estimate of the true value ${E}_{{\rm{m}}}^{{\rm{c}}}$. ${E}_{{\rm{m}}}^{{\rm{c}}}$ is useful for revealing the true statistical correlation with other variables, such as the PCI (*E*_PC_) or auroral current strengths (SML). The process of statistically correcting for the uncertainty using the conditional expectation of the true value given the erroneous estimate ${E}_{{\rm{m}}}^{{\rm{c}}}={f}_{{\rm{r}}}({E}_{{\rm{m}}}^{* })$ for an unbiased regression analysis is called regression calibration^19^. Figure 3 shows the results of this calibration. SML is the westward auroral electrojet index from the SuperMAG database, which is a measure of the westwards currents flowing in the auroral regions^41,42^. It is calculated using ground magnetometers and procedures completely independent of the calculation of the PCI. As regression calibration of ${E}_{{\rm{m}}}^{* }$ to ${E}_{{\rm{m}}}^{{\rm{c}}}$ results in a linear relationship between both ${E}_{{\rm{m}}}^{{\rm{c}}}$ and *E*_PC_ (Fig. 3a) and ${E}_{{\rm{m}}}^{{\rm{c}}}$ and SML (Fig. 3b), it suggests that our findings are independent of the construction of the ionospheric response variables. The confidence intervals shown in Figs. 1–3 provide only an estimate of the reliability of the regression procedure, that is, it means 95% probability that repeating the procedure will contain the regression curve and does not imply that the true value or the true regression curve will lie within the confidence interval with a 95% probability.

### Temporal uncertainty can lead to a nonlinear regression function

In Supplementary Methods 2c, we analytically derive the following general result that temporal uncertainty in measurement can lead to a perception of nonlinearity in a linear system’s correlation response. For a linear system with response *Y*, input *X*, and the input with measurement error *W* = *X*(*t* + *Δ*), the biased system response ⟨*Y*|*W*⟩ is a function ${f}_{{\rm{r}}}^{* }(w,{\sigma }_{\delta }/k)$. Here *σ*_*δ*_ is a measure of the random uncertainty *Δ* in the time of measurement and *k* is the autocorrelation time constant. The system response is a function of the ratio *σ*_*δ*_/*k*. This ratio is a measure of uncertainty in the time of measurement. We numerically integrate the function, which is fully described in equation (1.21) in Supplementary Methods 2c, within the ranges of *Δ* for specific values of *σ*_*δ*_/*k* and generate Extended Data Fig. 4c. The figure shows that ${f}_{{\rm{r}}}^{* }(w,{\sigma }_{\delta }/k)$ varies nonlinearly with *w* when there is temporal uncertainty.

## Online content

Any methods, additional references, Nature Portfolio reporting summaries, source data, extended data, supplementary information, acknowledgements, peer review information; details of author contributions and competing interests; and statements of data and code availability are available at 10.1038/s41586-026-10757-4.

## Supplementary information

Supplementary Information This file contains Supplementary Discussion 1a–j and Supplementary Methods 2a–d. The Supplementary Discussion explains the space physics background, regression to the mean as a fundamental property, the interpretation of the error model and the linearity of the geomagnetic response after correcting for regression bias. Supplementary Methods describe the utility of the Kan–Lee electric field as a driver function, validation of the error model, the analytical derivation of how time uncertainty causes regression bias, and a summary of the data analysis procedure used in the computer code.

Peer Review File

## Figures and Tables

**Extended Data Table 1:**
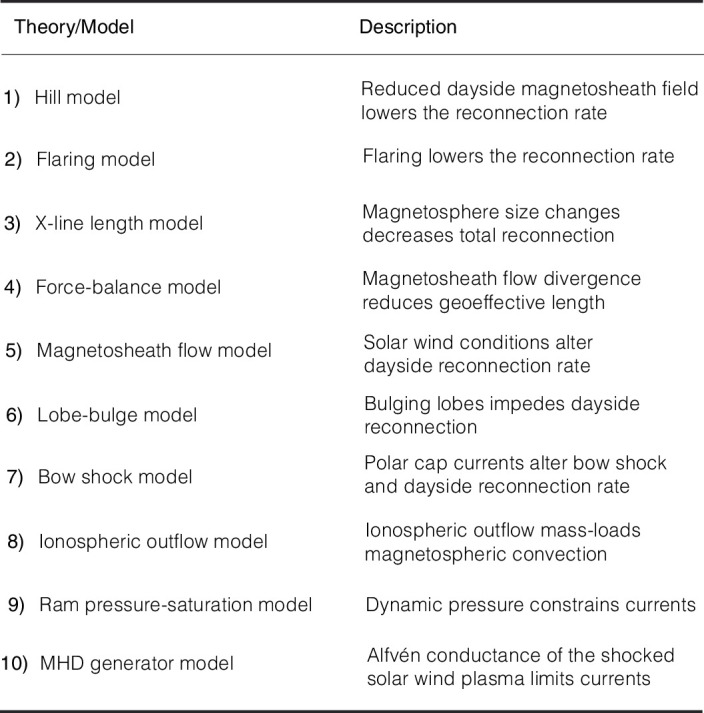
Theories of polar cap potential saturation

Ten models explaining cross polar cap potential saturation. See the following works for more details about each of the models: 1^54,55^, 2^56,57^, 3^58,59^, 4^60^, 5^59,61^, 6^62^, 7^63^, 8^64^, 9^65^,10^66,67^ and an assessment of most of them^5^. Table adapted with permission from ref. ^5^, Wiley.

**Extended Data Table 2:**
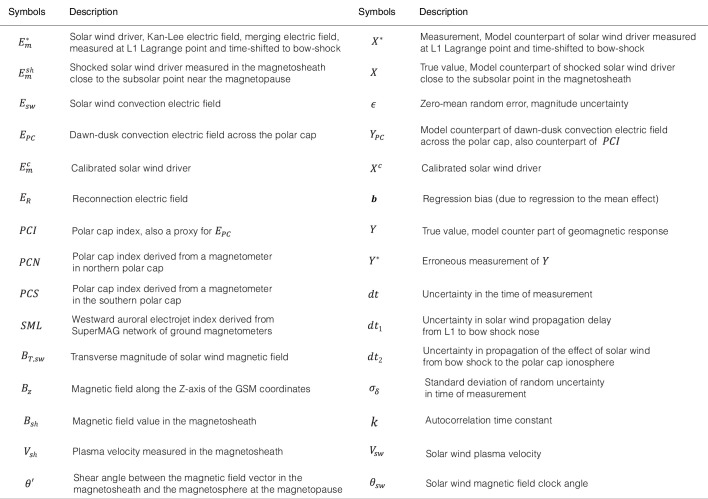
Common symbols used in this work

Variables representing measurements and their corresponding counterparts in the error model used commonly throughout the paper are summarized here.

## Data Availability

All data we have used are publicly available. We thank GSFC/SPDF OMNIWeb service for the WIND spacecraft measurements^43^. The WIND spacecraft measurements of the solar wind propagated to the bow-shock can be accessed from https://spdf.gsfc.nasa.gov/pub/data/omni/high_res_omni/sc_specific/. The MMS, Cluster, DoubleStar and THEMIS data can be accessed from the GSFC/SPDF web service https://spdf.gsfc.nasa.gov/. The PCI values PCN and PCS were downloaded from https://pcindex.org/archive. We thank O. Troshichev of the Arctic and Antarctic Research Institute for this data (https://pcindex.org/contacts). The auroral electrojet indices were downloaded from the SuperMAG database https://supermag.jhuapl.edu/indices/. For SuperMAG indices, we acknowledge the SuperMAG collaborators (https://supermag.jhuapl.edu/info/?page=acknowledgement). This Zenodo repository archives the data used in the paper^44^ (10.5281/zenodo.17546718). The broader dataset of magnetosheath measurements is published at 10.5281/zenodo.19671706 (ref. ^45^).

## Code availability

All the relevant data and the MATLAB and Python codes to process and visualize them have been curated and uploaded in the associated compute capsule (10.24433/CO.7006150.v1) and archived at 10.5281/zenodo.17559999 (ref. ^46^).
