# Supplementary material for: Regression to the mean can explain saturation of geomagnetic storms
Source: Nature. 2026 Jul 15;655(8125):1143–7. doi: 10.1038/s41586-026-10757-4 (PMC13421327; doi:10.1038/s41586-026-10757-4)
Supplement: Supplementary file 1 — This file contains Supplementary Discussion 1a–j and Supplementary Methods 2a–d. The Supplementary Discussion explains the space physics background, regression to the mean as a fundamental property, the interpretation of the error model and the linearity of the geomagnetic response after correcting for regression bias. Supplementary Methods describe the utility of the Kan–Lee electric field as a driver function, validation of the error model, the analytical derivation of how time uncertainty causes regression bias, and a summary of the data analysis procedure used in the computer code. [file 41586_2026_10757_MOESM1_ESM.pdf]

nature portfolio

https://doi.org/10.1038/s41586-026-10757-4

Supplementary information

Regression to the mean can explain saturation of geomagnetic storms

In the format provided by the authors and unedited

Contents

1 Supplementary Discussion 3
a. Physics of solar-wind magnetosphere coupling 3
b. An example of the problem of definition 4
c. Random error in the independent variable does not average away 4
d. Regression to the mean from different vantage points 4
e. Regression to the mean as a fundamental property of the relation between truth and measurement 5
f. Regression bias due to magnitude and time uncertainty 6
g. Interpretation of the Error Model output 7
h. A different measure of geomagnetic response is also linear 7
i. Historical development of the polar cap potential saturation problem 8
j. Evidence in Literature 8
2 Supplementary Methods 10
a. Kan-Lee electric field is an approximation of the true solar wind driver 10
b. Validation of the Error Model 10
c. Analytical derivation of non-linear regression bias due to uncertainty in time 12
d. Summary of data analysis procedure in the computer code 15
Additional References 18

1 Supplementary Discussion

a. Physics of solar-wind magnetosphere coupling

The Earth's magnetic field carves out a cavity in the oncoming solar wind, called the magnetosphere. It mostly keeps out the plasma and magnetic field associated with the solar wind except under specific local magnetic field conditions that lead to the reconnection of solar wind and magnetospheric magnetic field lines. Upstream from the reconnection-site, the supersonic and superalfvénic solar wind slows down through a bowshock, forming the magnetosheath just upstream from the magnetosphere's outer boundary—the magnetopause. When the shocked solar wind magnetic fields lie anti-parallel to those of the Earth, the field can undergo a topological change that opens up the Earth's magnetic field lines and reconnects them to the shocked solar wind magnetic field lines[2]. Through reconnection, the solar wind plasma and electric field can enter the Earth's magnetosphere and drive plasma convection in the magnetosphere. Conveyed to the polar ionosphere closer to the Earth, this convection electric field applies an electric potential across the polar cap on average[68] (See Figure 1a). The projection of the solar wind convection electric field along the dawn-dusk direction in the polar cap is the Kan-Lee or merging electric field (E_m^*)[24], which we refer to as solar wind driving[69].

In the 1970s, estimates of the cross-polar cap potential (and its corresponding dawn-dusk electric field) were made from single ground magnetometers situated in the northern and southern polar regions. The argument was that the perturbations of the magnetic field measured by the polar magnetometers give an excellent continuous estimate of the solar wind driving, as the polar regions magnetically connect to the solar wind[70]. Continuous estimates of solar wind driving from satellite missions were not yet available at the time[71]. As the magnetized plasma in the solar wind and magnetosphere is collisionless, the resistance along magnetic field lines is minuscule. Hence, the magnetohydrodynamic theory is valid, ensuring that plasma along an entire field-line moves as one[2]. Therefore, the solar wind electric field, resulting from the motion of the wind, at one end of the magnetic field line, would map along the field line to the polar ionosphere. This electric field then drives currents in the ionosphere that cause magnetic perturbations measured from ground magnetometers. Supporting this theory, the cross polar cap index (PCI), a measure of geomagnetic activity, increases linearly on average with the merging electric field (E_m^*), a measure of the solar wind driving[3]. The PCI is estimated by a polar magnetometer from the ground in each hemisphere and is proportional to the cross polar cap potential[11,12], while E_m^* is measured from a spacecraft in the solar wind far upstream of the Earth. Through the years, as more measurements of rare and extreme solar wind driving accumulated, the correlation between the geomagnetic response and the solar wind driver began to deviate from the linear relation. There appeared to be an upper limit to the cross-polar cap potential with increasing driving (See Figure 1b green curve).

The new inference that the cross-polar cap potential saturates with increasing solar wind driving led to the emergence of 10 different but sometimes overlapping theories and models attempting to explain the phenomenon (See Extended Data Table 1)[4,5]. The theories fall into two categories, one arguing that the conditions at the solar wind/magnetosphere interaction region (i.e., the dayside magnetosheath) during extreme solar wind driving lead to a consistent diminishing of the rate of reconnection or energy coupling from the solar wind to the magnetosphere. The other set of theories argues that processes within the magnetosphere slow down ionospheric plasma convection during extreme solar wind driving. All theories suggest that the energy transferred into the polar ionosphere during extreme space weather has, in essence, an upper limit. Hence, the theories imply that the Earth's magnetosphere shields us from extreme

geomagnetic storms by inhibiting energy transfer from the strong solar wind to the ionosphere, one way or the other.

In this paper, we present a radically different proposition. We argue that there is no statistical evidence for the saturation, and its appearance in measurements is a result of uncertainty in the driver. We correct for the effect of this uncertainty and demonstrate that the geomagnetic response varies linearly with solar wind driving, implying that the energy transfer from extreme solar wind conditions to the polar ionosphere can be far greater than currently believed. The importance of this work goes beyond space science to any correlation study between a measure of a system's input and its response, revealing a larger response for extreme input values.

b. An example of the problem of definition

A carpenter might measure the height of a door frame using a measuring tape. However, the door she creates using this height will likely not fit the frame. Even though the least count of the tape is small and does not contribute much to the uncertainty, there is uncertainty due to an implicit assumption that the height of the door is a one-dimensional quantity when it is in reality at least a two-dimensional quantity, i.e., there are multiple different heights for the door and the door frame, varying across its breadth and width. Hence, the experienced carpenter would use the carpenter's square to ensure that the edges of the door are perpendicular to the length, to match the door frame, and reduce this uncertainty.

c. Random error in the independent variable does not average away

The essence of regression analysis is to find the average of the measurement Y^* when the measurement X^* is a specific value 'x'. This is equivalent to the conditional expectation of Y^* given $X^* = x$, mathematically represented as $\langle Y^* | X^* = x \rangle$. Here, Y^* is the dependent variable and X^* is the independent or conditional variable. The averaging of Y^* indeed removes the effect of random error in Y^*, consistent with the widely held belief. But, crucially, the conditional or independent variable X^* cannot be averaged while simultaneously averaging the dependent variable Y^* for a given value of X^*. In other words, the random error in the conditional variable remains unaddressed and is not averaged away[17]. And, surprisingly, this unbiased random error in the conditional variable (X^*) manifests as a bias in the relation inferred between the variables Y^* and X^*. This *regression bias* is such that the average value of Y^* for a given X^* will be closer to the mean of Y, and if the regression bias is non-linear, it can create an appearance of saturation in Y^* for increasing X^*. This statistical phenomenon is a result of a *regression to the mean*[6,18].

d. Regression to the mean from different vantage points

There are five vantage points from which we can explain the regression to the mean effect.

Vantage point 1: Regression bias. The average value of a dependent variable given the independent variable will be biased closer to the mean value when there is some measurement uncertainty in the independent variable. Explained in Supplementary Discussion 1.c.

Vantage point 2: Repeat measurements. Extreme measurements are more likely followed by measurements closer to the mean. This is the most common understanding of the regression to the mean effect.

Vantage point 3: Likelihood of Stochastic Processes. Any physical process has an underlying probability distribution; therefore, some values of the physical process are more likely than others. For a stochastic process with a Gaussian distribution, the mean value coincides with the most likely or probable value. As a result, any uncertain measurement of the stochastic process is more likely measuring the true

value closer to the mean of the stochastic process than more extreme values, by the simple fact that values closer to the mean are more common and hence more likely than the extreme values. In other words, the true value being measured is more likely to be closer to the mean of the stochastic process than the measurement.

Vantage point 4: Probability Theory. Extended Data Fig. 1a geometrically shows that for a Gaussian process, the true value is likely closer to the mean than the measurement. The black line is the probability distribution of a Gaussian process, X. When this process is measured with some uncertainty, the measurement can take the values in the likelihood quantified by the dashed-blue curve when the true value is X=2 and the dashed-red curve when it is X=4. Since the true value is generally inaccessible, the goal of a measurement is to estimate the most probable true value corresponding to the measurement. Hence, when the measurement X*=3 (solid black vertical line), the probability that the corresponding true value X is also equal to 3 (black dot) is remarkably *less likely* than it being equal to 2 (blue dot), simply because X=3 is less likely to occur than X=2 for a Gaussian process with zero mean.

Vantage point 5: Relation between truth and measurement. From vantage point 4, and consistent with other three vantage points, we can infer that regression to the mean is a property of the relation between truth and measurement, the effects of which are observable in statistical methods, and can be examined through probability theory. This new vantage point is the general result presented in this manuscript, further explanation is provided in Supplementary Discussion 1.e. The fact that regression to the mean is not only a statistical effect, but also a property of the relation between truth and measurement, imply that the effect is present even where a single measurement is made, and hence is likely ubiquitous.

e. Regression to the mean as a fundamental property of the relation between truth and measurement

By fundamental property we mean a property that exists in reality, independent of the methodology we use to study it, as opposed to an effect that arises from the methodology. For example, an undersampling bias is an effect that arises purely from the number of samples we use in our analysis. All methods that use sufficient samples will never know of the undersampling bias. We argue that the regression to the mean effect, on the contrary, exists in reality as a property of the relation between truth and measurement independent of the methodology we use.

The first evidence for this comes from the fact that the phenomenon is observable in multiple statistical methods that use populations with inherent variability (e.g., different types of regression analysis, repeat measurements, machine learning, estimating conditional probability distributions), and also observable through probability theory when inferring the truth corresponding to the measurement. Supplementary Discussion 1.d explains regression to the mean effect from different vantage points. The second evidence is the mathematical result that can be derived using probability theory, graphically illustrated using Extended Data Fig. 1a, which shows that the effect only depends on the nature of the truth and measurement i.e., their likelihood of occurrence and their uncertainty. This implies that even when one makes a single measurement, the regression to the mean effect leads to a bias in the true value corresponding to the measurement according to the stochastic properties of the phenomenon being measured and the uncertainty in the measurement.

Here, an example of a familiar but different fundamental property of relations between things, e.g., the electric field, might be instructive and help us see the features of a fundamental property. An electric field

is a fundamental property of the relationship between two or more charges. This relation, like all relations, has a qualitative aspect and a quantitative aspect. The quality of electric field is that it displaces the two charges relative to each other. The quantitative aspect is that the displacement depends on the nature of both the charges, its sign, magnitude, and distance between them. It was demonstrated to exist through experiments conducted by Faraday, but that does not mean it is only an experimental effect. It was proven to be mathematically consistent by Maxwell, but that does not mean it is only a mathematical effect or "field-theoretic" effect. This relationship between charges exists in reality, irrespective of the tools we use to observe it.

Similarly, when we say regression to the mean or the more-probable, is a fundamental property of the relationship between truth and measurements, it also implies, this relation, like all relations, has a qualitative and quantitative aspect. The quality of this property is that the truth is separated from the measurement by some measure. The distance of separation depends on the nature of the truth and the measurement, i.e., the stochastic properties of the truth, the degree of uncertainty of the measurement, and how it changes with time, space and magnitude of the truth. It can be demonstrated to exist through experiments with a large number of measurements (i.e., statistical methods), but that does not mean it is only a statistical effect. It can be proven to be mathematically consistent using probability theory (Extended Data Fig. 1a), but that does not mean it is only a probabilistic effect. This relationship between truth and measurement exists in reality, irrespective of the tools we use to observe this relationship.

The existence of this relation between truth and measurement arises only because they are different from each other. Here we provide a philosophical insight into why that is the case. In our universe, truth is generally only accessible through measurements - this is because the universe is a complex system. Complex systems have many processes, that the most prominent process will have a systematic effect that we can observe, which we may call a "law", or we infer as the physical process of our interest. However, on the flip side, it will have other processes that appear as random perturbations, which may not be of our interest, and hence appear as randomness. This forces a separation between truth and measurements, leading them to be both different things. Truth is not measurement, and measurement is not the truth. However, there is an inseparable connection between them. Truth cannot be accessed without measurements, and measurements have no independent existence without the truth. This firmly establishes the existence of at least one, if not many, relationships between truth and measurement. One such relation is the regression to the mean of the truth, where the randomness manifests as uncertainty in measurements, and stochastic variations in the truth, leading to a separation of the truth from measurement in quantifiable ways.

f. Regression bias due to magnitude and time uncertainty

As shown by Sivadas & Sibeck, 2022[18] using a simple Monte-Carlo error model, if X has a Gaussian distribution, and the error ϵ is also Gaussian and independent of X like most instrumental errors (i.e., homoskedastic error[19]), then the likely true value for a given measurement (i.e., the average of X given X^* or $\langle X|X^*\rangle$) will have a linear bias. The purple curve in Extended Data Fig. 1b shows this linear bias as a lower slope than the dashed-black line of equality representing the curve if there were no uncertainty (i.e., the true unbiased relationship). However, suppose the error ϵ depends on X, and varies with the magnitude of X (i.e., heteroskedastic error[19]) like some uncertainties related to the 'problem of definition', and X has a log-normal distribution like many solar wind parameters[20]. In that case, the key point is that the likely true value for a given measurement will have a larger and non-linear bias that mimics a *saturation* of X with increasing X^*. Extended Data Fig. 1c demonstrates this in the purple curve whose slope has a non-

linear bias with respect to the black-dashed line of equality representing the true unbiased relationship. Supposing that X is the true solar wind driver near the Earth, and X^* is the uncertain measurement far from Earth, the response Y of the Earth will be driven by the true driver X and, crucially, not by the measurement X^*. If the response Y is linearly related to X (i.e., $Y \propto X$), then naturally, the response Y will also appear to saturate with increasing value of the measurement X^*.

Furthermore, if we keep aside the magnitude uncertainty (i.e., set $\epsilon = 0$), and examine the effect of uncertainty in time dt, we observe that it will lead to a non-linear bias in the average of X given X^* as well, with increasing deviation from linearity with growing uncertainty in time defined by the ratio of the temporal uncertainty (δ) and the autocorrelation time constant (k) of the stochastic process. Extended Data Fig. 4c shows quantitatively how the average of the true value X given the measurement X^* has a non-linear bias in its slope with respect to the dashed-black line of equality representing the true unbiased relationship. This non-linear bias increases with increasing ratio of δ/k , a dimensionless measure of the uncertainty in time. In the Supplementary Methods 2.c, we derive analytically from first principles this non-linear bias and its dependence on temporal uncertainty, which will be relevant for any study with measurement of systems that have a delay in their drivers or response, due to propagation of information, or dynamics of the system e.g., seismic waves from earthquakes[72], or response of medical patients to a specific chronic pain treatment[23].

g. Interpretation of the Error Model output

Once the calculated uncertainty values are worked into the error model, and X is assumed to have a log-normal distribution with an autocorrelation coefficient of ~100 minutes observed in solar wind measurements, the Monte-Carlo error model solves for X^* and predicts an appearance of saturation of X given X^* (See Figure 2b). This saturated curve, and the conditional probability distribution of X given X^*, matches surprisingly well with the saturation that appears in the data for the polar cap index PCI given the merging electric field estimated from solar wind measurements E_m^* (See Figure 2a). The solution of the error model is consistent with data as viewed through both the conditional expectation $\langle PCI|E_m^*\rangle$ and the conditional probability distribution of $pdf(PCI|E_m^*)$ (See Figure 2a-b). Further validation of the error model with second order statistics of data is presented in the Methods section and Supplementary Methods 2.b. Here PCI is analogous to Y in the error model, E_m^* is analogous to X^*, and E_m^{sh} analogous to X. The similarity in the curves $\langle PCI|E_m^*\rangle$ and $\langle X|X^*\rangle$, indicate that the geomagnetic response is proportional to the true shocked solar wind driver i.e., $PCI \propto E_m^{sh}$ and equivalently $Y \propto X$. The preconditions of this non-linear saturation relation between PCI and E_m^* are: 1) the heteroskedastic nature of the error in E_m^*, 2) the propagation time uncertainty, 3) the fact that the solar wind driver is a log-normal process with a particular autocorrelation time constant.

h. A different measure of geomagnetic response is also linear

Our finding that uncertainties in the solar wind driver results in the saturation of the polar cap index, raises the possibility that other measures of the geomagnetic response previously reported to saturate (or not)[73] based on uncertain solar wind driver estimates may be incorrect. The SML index, which is the westward auroral electrojet strength, measured by completely different magnetometers located at high latitudes ($\sim 65^\circ N$) around the planet, also appears to saturate with erroneous solar wind driving estimates. However, the corrected solar wind driver values, calibrated exactly the same way as above, show a linear relationship with the westward auroral electrojet strength as well—a further confirmation that the high-latitude geomagnetic response does not saturate with solar wind driving, once we remove the effect of

uncertainties. Figure 3b shows the linear relation of the SML index with corrected solar wind driver in the purple curve, once the regression calibration is applied to the erroneous data in the saturating green curve.

i. Historical development of the polar cap potential saturation problem

The regression to the mean of extreme solar wind driver values can sometimes be confused with under-sampling bias. Under-sampling is a statistical effect entirely distinct from the regression to the mean effect. The under-sampling bias reduces with increasing samples, however, the regression to the mean remains the same even with infinite samples. It is easy to see this from the analytical derivation in the methods section and Supplementary Methods 2.c (See Equation 1.21), which is immune to any sampling bias. However, under-sampling has had an important impact on the development of the polar cap saturation problem.

When examining space science literature on this problem, we observe from the 1980s to the 2000s (See Extended Data Fig. 6a-b) that as more and more solar wind data accumulates, the polar cap potential's saturation limit trends upwards from ~0.5 mV/m to ~10 mV/m. As extreme values of solar wind drivers are rare, their samples in data analysis can be poor. The number of samples increases with time, and hence the bias of under-sampling also diminishes with time. As shown by Extended Data Fig. 6b, due to this bias, observations suggest different relations between solar wind driving and polar cap potential, with different saturation limits and even no saturation (i.e., Boyle et al., 1997[47]). Since inferences from data do not agree with each other, the developers of a theory of saturation are also confounded by the question of which observational study they ought to explain. Such a diversity of inferences from data itself confuses the development of theoretical explanations. Hence, it is not surprising that multiple theories have emerged over the years (See Extended Data Table 1) without a unifying theory explaining the saturation effect quantitatively, as there are multiple different quantitative relations between solar wind driving and polar cap potential depending on the study.

Our work, that carefully addresses uncertainties in the driver with a sufficiently large database of 25 years of measurements, brings a resolution to the contradiction between the different inferences and shows that the evidence overwhelmingly concludes that the relation between the driver and the response is linear. It is this linear relationship between the driver and response that a new (or old) physical theory or model needs to explain and validate their predictions against.

j. Evidence in Literature

Some previous data-based studies like Boyle et al. (1997)[47] have found that solar wind driving varies linearly with cross-polar cap potential using spacecraft measurements after a meticulous filtering of data. Pulkkinen et al. 2016[74] found that on average, the auroral electrojet index varies linearly with the shocked solar wind electric field in the magnetosheath tangential to the magnetopause (a proxy for the local reconnection electric field). Additionally, they found that this magnetosheath electric field varies non-linearly (or saturates) with the solar wind electric field. Their findings are entirely consistent with our explanation that the variance of solar wind parameters relative to the local magnetosheath values is the source of the saturation effect and not some magnetosheath phenomenon. The increased variance observable within their own data suggests that the variance in the driver leads to the saturation, and not any physical effect that causes a consistent reduction in the local magnetosheath electric field as they suggest. Borovsky 2021[73] found that the ionospheric response of PCI and SML indices varies linearly with solar wind driving as quantified by the $S_{(1)(9b)}$ index. The $S_{(1)(9b)}$ index is developed using the method of canonical correlation analysis applied to a set of solar wind parameters and another set of geomagnetic responses. From many parameters in both sets, the method extracts a small number of canonical variate

pairs that contain the majority of shared information, relegating the uncertainty (and variance) that causes a lack of correlation to an orthogonal variate pair. As we have shown, since the uncertainty leads to the saturation effect, by reducing the uncertainty in the new driver $S_{(1)(9b)}$, Borovsky 2021 implicitly removed the non-linear regression bias in the relation between $S_{(1)(9b)}$ and geomagnetic response parameters like PCI and SML index. Hence, they have a linear relationship through the entire range of the solar wind driver $S_{(1)(9b)}$, consistent with our result.

2 Supplementary Methods

a. Kan-Lee electric field is an approximation of the true solar wind driver

The Kan-Lee electric field is an approximation of the component of the reconnection electric field ($E_R = V_{sh}B_{sh}\sin(\theta'/2)$) perpendicular to the geomagnetic field that creates the cross-polar cap electric field (which is equal to $V_{sh}B_{sh}\sin^2(\theta'/2)$ due to a geometrical argument presented first by Kan and Lee)[24]. The approximation assumes that the shear angle between the magnetosheath and magnetospheric magnetic field θ' is equal to θ_{sw}. Furthermore, the approximation assumes $V_{sh}B_{sh} \cong V_{sw}B_{T,sw}$, which is true most of the time 1) as the solar wind velocity component tangential to the bowshock is negligible compared to the normal component, and 2) as the tangential component of the electric field across a normal MHD shock is conserved. This allows the Kan-Lee electric field (E_m^*) to estimate the cross polar electric field E_{PC} with just solar wind parameters. Our examination of data shows that for electric field values <3mV/m, which is 95% of all available data points in 25 years, the Kan-Lee electric field (E_m^*) propagated (or time-shifted) to the polar cap ionosphere from the L1 point is linearly proportional to the cross polar electric field E_{PC}. This is well-documented in literature and is also observed for other forms of the solar wind coupling functions and polar cap index [4,26,75].

b. Validation of the Error Model

As time and magnitude uncertainty increase, the regression bias also increases. A lower uncertainty than estimated by us will result in a decrease in regression bias. Therefore, a conservative estimate of both time and magnitude uncertainty is made by us to present a defensible case for our result.

For magnitude uncertainty, when E_m^{sh} > 12 mV/m, we used uncertainties from simultaneous L1 measurements and solar wind values upstream of the bow shock, adopting a conservative estimate of the uncertainty (~30% relative error). For simultaneous magnetosheath measurements, the sample size is small, with fewer than 10 data points per bin, suggesting no extreme geomagnetic storms fall within the constraints of simultaneous L1 and sheath measurements near the subsolar point.

Similarly, for the time uncertainty dt_1, we use a scale parameter of ~8 minutes and a Student's t-distribution with shape factor 1.3 to simulate the narrow but heavy-tail distribution seen in Case & Wild's results[35]. Case & Wild estimate propagation delays directly by cross-correlating solar wind measurements from ACE and Cluster satellites, and compare these calculated delays with propagation delay estimates from the OMNI database we use. The regression bias caused by our estimated Student's t-distribution is slightly lower than the equivalent normal distribution with ~8 minutes 1σ standard deviation (i.e., it is a conservative estimate). Recently, Tasnim et al.[36] estimated the propagation delay using a combination of cross-correlation and validation methods, reporting a ~18-minute 2σ standard deviation in propagation delay uncertainty, consistent with our estimate.

For the time uncertainty dt_2, we use a standard deviation of ~25 minutes, consistent with the results of Milan et al.[16] and Stauning[27]. Milan et al.[16] derive the propagation delay to the ionosphere using cross-correlation of field-aligned currents and solar wind B_z. They find a time-lag distribution peaking at ~17 minutes with a broad peak ranging from 10-30 minutes. Stauning's[27] cross-correlation of PCN index with the solar wind merging electric field just upstream of the bow shock shows propagation delays from -5 to 50 minutes with an average of 20 minutes. We estimate the probability distribution of dt_2 as a Weibull distribution (shape factor 1.3) to capture the broad spread and ensure a positive total delay. The total regression bias caused by this distribution is again slightly lower than that of the equivalent normal distribution with a ~25-minute standard deviation, making it a conservative estimate. In other words, using

a normal distribution for propagation time uncertainties would yield a similar regression bias as seen in our results, implying the results are insensitive to the heaviness of the distribution's tail.

Using the Monte Carlo simulation, we estimate that a 30% change in our estimated magnitude uncertainty will lead to only a ~12.18% change in the regression bias, with all other parameters held constant and dt=0. Using the analytical equation [1.21] in Supplementary Methods 2.c, we estimate that a 30% change in our estimated time uncertainty will lead to only a ~9.65% change in the regression bias, with all other parameters held constant and $\epsilon = 0$. Similarly, we estimate that a 30% change in our estimated standard deviation of X will lead to only a ~5.9% change in the regression bias given dt=33 minutes and $\epsilon = 0$. These numbers show that the regression bias predicted by the error model is insensitive to a 30% error in the model inputs, especially since the sensitivity is less than the minimum relative magnitude error of ~30% (See Figure 2c). Therefore, for a change in the input, we see a much smaller change in the error model's output. In other words, the error model is robust to change in the estimated input parameters. Note all these values are calculated for regression bias observed in X for a given X*=25mV/m, as it is the most extreme and rare measurement and has the maximum sensitivity of all the measurements.

To further validate our error model with the inputs we have estimated, we compare the statistics of the error model's outputs with those of the data.

- The model predicts the pdf of the erroneous estimate of the shocked solar wind driver E_m^*. As shown in Extended Data Fig. 3a, the pdf of X^*closely fits the pdf of E_m^*.

- The model predicts the standard deviation of the normalized error. A good way to visualize how the uncertainty varies with the magnitude of X^* is to calculate the standard deviation of the normalized error $\sum(X^* - \mathrm{X})/\mathrm{X}$. This quantity largely matches with its counterpart in data $\sum(E_m^* - E_{PC})/E_{PC}$ (See Extended Data Fig. 3b). This match implies the shocked solar wind driver $E_m^{sh} \propto E_{PC}$, and our assumption is consistent with even the second order statistics. This also aligns with the fact that E_{PC} is constructed by maximizing the correlation with E_m^*[3], hence all the contribution to the variance comes from the difference between E_m^{sh} and E_m^*, instead of uncertainties in the measurement of E_{PC}. If the values we assume for the uncertainties dt_1, dt_2, and ϵ, are inadequate, the model will underestimate or overestimate the statistical variation in the difference between E_m^* and E_{PC} shown in Extended Data Fig. 3b.

- Furthermore, the model predicts the conditional normalized error distribution itself. A more detailed picture of the statistical properties of E_m^* can be obtained by plotting the conditional probability density of the normalized error given X or E_{PC}. In Extended Data Fig. 3d-e, the left plot is the conditional pdf of normalized error calculated from the data, and the plot on the right is the same from the model. Both have a similar structure, with the spread in error increasing up to ~12 mV/m and then decreasing. This is consistent with our estimate of magnitude uncertainty in Figure 2c.

- Finally, the model shows that the estimates of random, unbiased uncertainties in the solar wind input (X^*) lead to a conditional bias in the estimate of the true driver X, with the bias varying with the magnitude of X^*. This bias exactly reproduces that seen in the data. In Extended Data Fig. 3c, the black line is plotted from data and shows the conditional bias in the erroneous estimate E_m^* given its strength. It matches remarkably with that calculated by the model – shown in purple. This non-linearly increasing bias is ultimately a result of the regression to the mean effect[6,18,19].

Extended Data Fig. 5 is another way to explain this effect. The left panel shows the probability distribution of the true value X. Each bin associated with X is assigned a unique color. The panel on the right shows the pdf of the error-prone estimate of X, i.e., X^*. However, the colors still preserve their correspondence to the true value. The share of color in a particular X^* bin describes the proportion of X values misidentified as X^*. In each X^* bin, a larger proportion of lower X values are misidentified as X^*. In other words, for large X^*, X is less than expected. Converting this into the terminology of observations, we get back to the statement of the problem introduced in the main paper: for large values of E_m^*, E_m^{sh} is less than expected or appears to saturate.

c. Analytical derivation of non-linear regression bias due to uncertainty in time

Here we analytically derive the result that temporal uncertainty in measurement can lead to a perception of non-linearity in a linear system's correlation response. For this, we first assume a linear system with an input random variable X and output response variable Y, such that

$$Y = X \quad [1.3]$$

The input is measured or estimated with a temporal uncertainty represented by the random variable Δ. The erroneous estimate is W and is related to the true input X as

$$W = X(t + \Delta) \quad [1.4]$$

where the statistics of $X$and Δ are known

$$X \sim f_X(x)$$
$$\Delta \sim f_\Delta(\delta)$$

Our goal here is to find the erroneous regression function $\langle Y|W\rangle = f_r^*(w)$ and see how different it is from the true regression function $\langle Y|X\rangle$. Based on equation [1.3], $\langle Y|X\rangle$ is simply $f_r(x) = x$. We also note that since the system has a simple linear response $\langle Y|W\rangle = \langle X|W\rangle = f_r^*(w)$. For the rest of the section, our goal is to derive the function $\langle X|W\rangle$ or $f_r^*(w)$.

Firstly, we note that since $W(t)$ is a stochastic process, from equation [1.4], the conditional distribution

$$f_{W|\Delta}(w|\Delta = \delta) = f_{X(t+\delta)}(w)$$

But since X is assumed to be a stationary process,

$$f_X(w) = f_{X(t+\delta)}(w)$$
$$\Rightarrow f_{W|\Delta}(w|\Delta = \delta) = f_X(w) \quad [1.5]$$

However,

$$f_W(w) = \int f_{W,\Delta}(w, \delta) d\delta$$
$$= \int f_{W|\Delta}(w|\Delta = \delta) f_\Delta(\delta) d\delta \quad [1.6]$$

And from equation [1.6],

$$f_W(w) = \int f_X(w) f_\Delta(\delta) d\delta$$

As we assume X and Δ are independent, and $\int f_{\Delta}(\delta)d\delta = 1$, hence

$$f_W(w) = f_X(w) \quad [1.7]$$

That is, **the marginal distribution of W and X has the same functional form**.

From equations [1.5] and [1.7], it follows that W **and Δ are also independent**.

$$\begin{aligned} f_{W,\Delta}(w,\delta) &= f_{W|\Delta}(w|\delta)f_{\Delta}(\delta) \\ &= f_X(w)f_{\Delta}(\delta) \\ &= f_W(w)f_{\Delta}(\delta) \end{aligned} \quad [1.8]$$

Now we estimate the joint probability distribution function of W, X, and Δ.

$$f_{W,X,\Delta}(w,x,\delta) = f_{W|X,\Delta}(w|x,\delta)f_{X,\Delta}(x,\delta)$$

Since $X$and $\Delta$are independent,

$$f_{W,X,\Delta}(w,x,\delta) = f_{W|X,\Delta}(w|x,\delta)f_X(x)f_{\Delta}(\delta) \quad [1.9]$$

By integrating the above over the range of Δ, we can calculate the marginal pdf of W and X.

$$f_{W,X}(w,x) = \int f_{W|X,\Delta}(w|x,\delta)f_X(x)f_{\Delta}(\delta)d\delta \quad [1.10]$$

Finally, we can calculate the conditional pdf of X given W.

$$f_{X|W}(x|w) = \frac{f_{W,X}(w,x)}{f_W(w)}$$

From equation [1.7], we see that

$$f_{X|W}(x|w) = \frac{f_{W,X}(w,x)}{f_X(w)}$$

Using this and equation [1.10], we get

$$f_{X|W}(x|w) = \int f_{W|X,\Delta}(w|x,\delta)\frac{f_X(x)}{f_X(w)}f_{\Delta}(\delta)d\delta \quad [1.11]$$

Hence from the definition of expectations we get,

$$\langle X|W\rangle = f_r^*(w) = \int x f_{X|W}(x|W=w)dx \quad [1.12]$$

To solve equation [1.12], we need to assume a functional form for the pdf of X and Δ. $f_X(x)$ is assumed to be lognormal distribution, and $f_\Delta(\delta)$ to be a normal distribution. A lognormal random variable X has a corresponding normally-distributed random variable Z such that

$$Z \sim \phi(z, \mu_Z, \sigma_Z)$$
$$Z = \log X$$
$$\mu_Z = \log\left(\frac{\mu_X^2}{\sqrt{\mu_X^2 + \sigma_X^2}}\right) \quad [1.13]$$
$$\sigma_Z = \sqrt{\log\left(1 + \frac{\sigma_X^2}{\mu_X^2}\right)}$$

Here, the function ϕ is the general normal distribution.

$$\phi(x, \mu, \sigma) = \frac{1}{\sqrt{2\pi}\sigma} e^{-(x-\mu)^2 / 2\sigma^2} \quad [1.14]$$

Therefore,

$$f_X(x) = \frac{1}{x}\phi(\log x, \mu_Z, \sigma_Z) \quad [1.15]$$

Additionally, as $X(t)$ is a stochastic process, the random variable at each time instance correlates to some degree with adjacent time instances. We define this using an autocorrelation function of the following form

$$\rho_X(\delta, k) = e^{-\delta / k} \quad [1.16]$$

Here $\delta = |t_2 - t_1|$ is the absolute difference in time between two random variables, $X(t_2)$ and $X(t_1)$. For our case, this is also the error in measurement time corresponding to $\Delta \sim f_\Delta(\delta)$. ***k* is the autocorrelation time constant**.

The normally distributed uncertainty in time is defined as

$$f_\Delta(\delta) = \phi(\delta, 0, \sigma_\delta) \quad [1.17]$$

Here σ_δ **is a measure of the random uncertainty in the time of measurement** Δ.

From equations [1.13] and [1.16], we can estimate the autocorrelation function corresponding to the normal random variable Z

$$\rho_Z(\delta, k) = \frac{1}{\sigma_Z^2}\log\left(1 + \rho_X(\delta, k)\left[e^{\sigma_Z^2} - 1\right]\right) \quad [1.18]$$

From the results of the bivariate lognormal distribution, we can derive the functional form of $f_{W|X,\Delta}(w|x,\delta)$ to be the following

$$f_{W|X,\Delta}(w|x,\delta) = \frac{1}{w}\phi\left(\log w, \mu_Z + \rho_Z(\delta,k)[\log x - \mu_Z], \sigma_Z\sqrt{1-\rho_Z(\delta,k)^2}\right) \quad [1.19]$$

From equations [1.11],[1.12],[1.15],[1.17], and [1.19], we can calculate the regression function$\langle X|W\rangle = f_r^*(w)$.

$$f_r^*(w) = \int_0^{\infty}\int_{-\infty}^{\infty} \frac{\phi\left(\log w, \mu_Z + \rho_Z(\delta,k)[\log x - \mu_Z], \sigma_Z\sqrt{1-\rho_Z(\delta,k)^2}\right)}{\frac{\phi(\log x, \mu_Z, \sigma_Z)}{\phi(\log w, \mu_Z, \sigma_Z)}\phi(\delta, 0, \sigma_\delta)} d\delta dx$$

[1.20]

By integrating the above integral within the ranges of x, and substituting $\frac{\delta}{k} \to u$, we can show that the above integral is a function of the ratio $\frac{\sigma_\delta}{k}$. This ratio as a measure of uncertainty in the time of measurement.

$$f_r^*\left(w, \frac{\sigma_\delta}{k}\right) = \int_{-\infty}^{\infty} w^{\rho_Z(u)} e^{\frac{1}{2}[1-\rho_Z(u)][2m_Z + (1+\rho_Z(u))\sigma_Z^2]} \frac{1}{\sqrt{2\pi}\,(\sigma_\delta/k)} e^{-\frac{u^2}{2(\sigma_\delta/k)^2}} du$$

$where\ u = \delta/k$
[1.21]

We numerically integrate the above integral within the ranges of Δ for specific values of σ_δ/k and generate Extended Data Fig. 4c. The pdf of X is similar to that assumed in the previous section, with the mean $\mu_X = 1.12$ and standard deviation $\sigma_X = 1.15$ The figure shows that $f_r^*(w, \frac{\sigma_\delta}{k})$ varies non-linearly with w when the temporal uncertainty ratio is larger than 0%, compared to $f_r(x) = x$ (which is linear) when $W = X$ or the temporal uncertainty ratio is 0%. Therefore, we have found that the regression function $\langle Y|W\rangle = f_r^*(w, \frac{\sigma_\delta}{k})$ is non-linear with respect to w for values of $\frac{\sigma_\delta}{k} > 0$. For low values of the temporal uncertainty ratio $\frac{\sigma_\delta}{k} < \sim 0.1$, the regression bias is approximately linear. Finally, the numerical integration of the analytical solution described by [1.21] was compared with an independent Monte Carlo solution of the error model stated in equation [1.4], with the results being almost identical, confirming that our derivation is correct.

d. Summary of data analysis procedure in the computer code

The primary computer code (Code 1) constructs a Monte-Carlo simulation to solve a non-linear error model to show that the polar cap index saturation observed in data is a result of the regression to mean effect stemming from random error in the shocked solar wind driver estimates.

Step 1: Preparing and loading observations data. First 1-minute resolution WIND spacecraft data already time-shifted to bow-shock nose from the OMNI database, Polar Cap Index (PCI) data, and the magnitude uncertainty (in the form of standard deviation) varying with shocked solar wind driver (derived

in Code 3) is loaded. All missing values, and out-of-range values, are converted into a 'nan' value, PCI is calculated from PCN and PCS as described in the Methods section. The Kan-Lee electric field at L1 (E_m^*) is calculated from WIND spacecraft measurements of plasma and field parameters. Finally, all values of E_m^* and PCI that are not simultaneous are removed from the analysis. The difference between E_m^* and PCI from data is also calculated as a measure of the total error or difference between the driver and the response. Finally, the autocorrelation function of E_m^* , PCI, and $E_m^* -$ PCI is calculated.

Step 2: Constructing model input. We compare the pdf of E_m^* and PCI, create a log-normal pdf that best fits the PCI index values, which has mean 1.114 and standard deviation 1.14 and, in the log-space, has mean -0.2518 and standard deviation 0.85. We fit a spline-function on the autocorrelation values and compare the autocorrelation functions of E_m^* , PCI, and $E_m^* -$ PCI. From these values derived from measurements, we create the first input to the model: a stochastic process X which is the hypothetical true stochastic process (i.e., shocked solar wind driver value), with also an autocorrelation functions that is similar to that observed in data. For this we use an external function by Lienhard (2021) to generate multivariate lognormal random numbers with correlation. Then we construct random variables representing time uncertainties: dt1 and dt2 with distributions and parameters derived from previous literature and discussed in the Methods section. Next, we construct a stochastic process $\epsilon(t)$ representing the magnitude uncertainty, which has zero mean, and a variance varying with the magnitude of X according to the uncertainty derived directly from simultaneous magnetosheath and solar wind measurements using Code 3. $\epsilon(t)$ also has an autocorrelation function with values similar to that derived from the autocorrelation function of $E_m^* - PCI$.

Step 3: Solving the Monte-Carlo Error Model. By plugging in the above inputs of X, dt_1, dt_2 and ϵ, into the following error model $X^* = X\ (t + dt_1 + dt_2) + \epsilon$, we estimate the stochastic process X^*. These stochastic processes are represented by an ensemble of 16000 realizations, with each realization consisting of 2^{12} time samples (i.e., ~4096 minutes). This can be increased to improve the statistics at the very low probability tail region of the log-normal distribution (i.e., for solar wind driver values > 15 mV/m). The resulting value of X^* is the solution of the error model. From this we calculate the conditional expectation of $\langle X|X^*\rangle$, and notice that there is an appearance of a saturation effect on the average shocked solar wind driver given solar wind driver values measured at L1. This is then compared with the conditional expectation of $\langle PCI|E_m^*\rangle$, and we notice that the curves have a good correspondence, suggesting the regression to the mean effect is the cause for the saturation of the polar cap index with solar wind driver value measured at L1.

Step 4: Validating the Monte-Carlo Error Model. We verify the reliability of the error model by comparing many second-order statistics, as well as full probability distribution and conditional probability distributions with that of their counter parts in measurements. We list here the different statistics from the model that have been compared to show good correspondence with their counterparts in data: probability density function of input - $pdf(X)$, probability density function of output - $pdf(X^*)$, the autocorrelation function of the input - $\langle X(t)X(t+\tau)\rangle$, autocorrelation function of the output - $\langle X^*(t)X^*(t+\tau)\rangle$, normalized marginal error distribution - $pdf\left(\frac{X^*-X}{X}\right)$, the normalized marginal error distribution

conditioned on X - $pdf\left(\frac{X^*-X}{X}|X\right)$, the normalized marginal error distribution conditioned on X^* - $pdf\left(\frac{X^*-X}{X}|X^*\right)$, standard deviation of normalized error - $\sigma(X^* - X)/X$, mean of error - $\langle X^* - X\rangle$ and finally the conditional $pdf(X|X^*)$.

Step 5: Saturation effect in another geomagnetic response parameter (SML). We load the westward auroral electrojet index (SML) from SuperMag database, remove all the data gaps, and select only the data points that are simultaneous with the time-shifted Kan-Lee electric field measured from L1. We then assume a counter part for SML in the model, $Y_{SML} = -120X + \epsilon_Y$, where ϵ_Y is a Gaussian-distributed random error in the estimate of the SML value with zero mean and $\sigma_{\epsilon_Y} = 5$ units. When the conditional expectation of Y_{SML} given X^*, $\langle Y_{SML}|X^*\rangle$, is calculated we observe that it also saturates very similar to the saturation of SML index with solar wind driver values.

Step 6: Regression calibration: Correcting for the regression to the mean effect. The bias caused by the regression to the mean effect is quantified by $b = X^* - \langle X|X^*\rangle$, and this can be removed from X^* to estimate the corrected solar wind driver function $X^c = X^* - b = \langle X|X^*\rangle$. This is carried out in the code by a one-dimensional linear interpolation of the solar wind driver value measured at L1 and time-shifted to the bow-shock (E_m^*) with a look up table consisting of sample points $x_i \in [0,25]$ and its corresponding values $f(x_i) = \langle X|X^* = x_i\rangle$ for each query point E_m^*. Therefore, the result is $E_m^c = f_r(E_m^*)$. We now plot the conditional expectation $\langle PCI|E_m^c\rangle$ and $\langle SML|E_m^c\rangle$ all derived directly from data. They reveal a linear relation of PCI and SML with the corrected solar wind driver function (or the true shocked solar wind driver value). We refer interested readers to the following literature for further discussion on the distributions of solar wind parameters, and geomagnetic response measures[17,18,73,76,77].

Code 2 integrates the analytical formula in Eq [1.21] to solve for the regression bias stemming from uncertainty in the time of the measurement of a stochastic process. The code outputs a sensitivity analysis of the regression bias for different values of the ratio of time uncertainty to the autocorrelation time constant. Lastly, we validate the analytical solution independently; by developing an error model of only time uncertainty and solving it using the Monte Carlo method. The sensitivity analysis from the analytical result and the Monte Carlo solution match very well, confirming our analytical derivation is correct.

Code 3 uses simultaneous magnetosheath satellite measurements and measurements of spacecraft at L1 from the OMNI database, to calculate the variance of the L1 solar wind driver estimates for a given value of the shocked solar wind driver close to magnetopause subsolar point. The resulting variance is the magnitude uncertainty, and a spline fit of the variation of the magnitude uncertainty with increasing value of the shocked solar wind driver is used in Code 1 to reconstruct the magnitude uncertainty ε and its heteroskedastic variation with the magnitude of X in the error model in Code 3.

Code 4 uses probability theory to show that the true value corresponding to an uncertain measurement of a Gaussian random process is biased towards the mean. This is a demonstration of the regression to the mean effect through the lens of probability theory.

Additional References

68. Cowley, S. ~W. ~H. & Lockwood, M. Excitation and decay of solar wind-driven flows in the magnetosphere-ionosphere system. *Ann. Geophys.* **10**, 103–115 (1992).

69. Lockwood, M. Solar Wind—Magnetosphere Coupling Functions: Pitfalls, Limitations, and Applications. *Space Weather* **20**, e2021SW002989 (2022).

70. Troshichev, O. A. & Andrezen, V. G. The relationship between interplanetary quantities and magnetic activity in the southern polar cap. *Planet. Space Sci.* **33**, 415–419 (1985).

71. Dunham, D. ~W. ISEE-3, the first libration-point satellite. in *Bulletin of the American Astronomical Society* vol. 11 805–806 (1979).

72. Bonilla, M. G., Mark, R. K. & Lienkaemper, J. J. Statistical relations among earthquake magnitude, surface rupture length, and surface fault displacement. *Bulletin of the Seismological Society of America* **74**, 2379–2411 (1984).

73. Borovsky, J. E. On the Saturation (or Not) of Geomagnetic Indices. *Frontiers in Astronomy and Space Sciences* **8**, 740811 (2021).

74. Pulkkinen, T. I. *et al.* Magnetosheath control of solar wind-magnetosphere coupling efficiency. *J. Geophys. Res. Space Phys.* **121**, 8728–8739 (2016).

75. Doyle, M. A. & Burke, W. J. S3-2 measurements of the polar cap potential. *J. Geophys. Res. Space Phys.* **88**, 9125–9133 (1983).

76. Vörös, Z. *et al.* Probability density functions for the variable solar wind near the solar cycle minimum. *J. Geophys. Res. Space Phys.* **120**, 6152–6166 (2015).

77. Borovsky, J. E. Noise and Solar-Wind/Magnetosphere Coupling Studies: Data. *Frontiers in Astronomy and Space Sciences* **9**, 990789 (2022).
