# Supplementary material for: Regression to the mean can explain saturation of geomagnetic storms
Source: Nature. 2026 Jul 15;655(8125):1143–7. doi: 10.1038/s41586-026-10757-4 (PMC13421327; doi:10.1038/s41586-026-10757-4)
Supplement: Supplementary file 2 — Peer Review File [file 41586_2026_10757_MOESM2_ESM.pdf]

nature portfolio

Peer Review File

Regression to the Mean can Explain Saturation of Geomagnetic Storms

Corresponding Author: Dr Nithin Sivadas

Any redactions in this file are there to maintain patient confidentiality, the confidentiality of unpublished data, or to remove third-party material.

This file contains all reviewer reports in order by version, followed by all author rebuttals in order by version.

Version 0:

Reviewer comments:

Referee #1

(Remarks to the Author)
Review of "Regression to the Mean of Extreme Geomagnetic Storms"

The authors highlight in this significant study a regression-to-the-mean effect of Earth's responses to extreme solar wind driving. As investigated in the study, this effect is argued as the major cause for the commonly accepted saturation effect of the Sun-Earth response link. Meanwhile, this effect is also regarded to cover up a linear throughout response of Earth's environment to solar wind driving especially under extreme circumstances, which the authors think is more likely to be the reality. While regression to the mean is a common concept in the field of mathematics and statistics, this study is of prime significance because it is the first one so well detailed, both from the data side and the analyzing one, to demonstrate that random uncertainty in solar wind measurements may fundamentally impact current understanding of the Sun-Earth energy transfer system. It is clearly concluded that extreme geomagnetic storms may have twice the impact on Earth than previously thought, which goes against the commonly accepted view that a saturation level appears to exist for Earth's responses to extreme solar wind driving. This discovery is of significant interest to the entire space science community, including engineers, experimentalists, data scientists, modelers, and theorists, when they cope with the sophisticated system of Sun-Earth connection and the devastating space weather effects. Furthermore, it should bear broad impact and be of much interest as well to those studying random, uncertain measurement systems and their underlying impacts on various aspects of our society and beyond.

Before I can recommend the paper for acceptance, I have a number of specific comments and remarks that I would like the authors to address.

1. The first one concerns the statistical analysis of solar wind measurements and polar cap indices. As the authors mentioned in the paper, 25 years of WIND data and the polar cap indices from the same time range are adopted for the investigation. Then how to deal with such a large database is key to the research purpose. I would have appreciated to see a proper description regarding the data analysis procedure and the distribution statistics of both solar wind driving and polar cap/geomagnetic activity at different levels. On one hand, the results presented in the paper can be further justified and also become reproducible to readers. On the other hand, the spread of the variables can be easily recognized for better perception of the regression-to-the-mean effect between the measurements and the truth, especially under extreme conditions.

2. The second point relates to the way correcting the effect of uncertainties. As stated in the paper, the adopted method includes statistically quantifying the uncertainties, predicting their effect and eventually offsetting them from the inference. The Methods section also outlines explicitly three primary sources of the random uncertainty to deal with in the study. One essential step is to develop an error model to assess the non-linear bias associated with the measurements of solar wind driving. Then how do the authors justify the rationality of such a model and the calculated bias connected to different sources of the uncertainty? For instance, does it depend on solar wind driving condition or the regression analysis/calibration methodology? Furthermore, is it possible, based upon conjunction observations from multiple satellites (after all we have to more or less rely on these in situ measurements), to verify how reliable these analysis estimates can be.

3. Figure 4 shows Earth's responses to solar wind drivers after correcting the regression bias in the measurements. It also presents no data available beyond 15 mV/m after the correction. One reason can be the data absence in this quantity range. But it is natural to think what will the relationship look like with (Em)^c > 15 mV/m, say, very extreme solar wind driving conditions. To be even further, extreme conditions not just include super strong solar wind driving but also substantially quiet solar activity. How about the Earth's responses under such extremely quiet solar wind driving conditions? Does it keep a linear property or have the non-linear effect? It can be very informative to have a look at these conditions or at least give some discussions.

4. Since quite a large number of variables have been introduced in the text to refer to different parameters in association with solar wind driving, polar cap indices, geomagnetic activity, etc., which can be confusing to and difficultly followed by readers, a suggestion is that the authors can produce a table in the Supplementary to list and describe clearly them.

5. Figure 2 is a reproduction by combining Figs. 1-3 of Sivadas and Sibeck (2022). Since this is a submission to Nature, it is suggested to replace Figure 2 if available.

(Remarks on code availability)
The code provides a very useful aid to understand what the authors have done to deal with correcting the effect of uncertainties in association with measurements of solar wind driving and geomagnetic responses of Earth.

Referee #2

(Remarks to the Author)
This manuscript describes an alternate (and believable) explanation for a long-standing problem in magnetospheric physics and how the space-physics community improperly interprets the data pertaining to this problem. For decades the community has come up with a number of physical explanation for the "problem", but the analysis of the authors demonstrate that in fact there is no "problem". This research is very original and it is significant for magnetospheric physics and for other statistical problems in other fields of science and medicine. The methodology is correct and the data is excellent. The authors also use numerical experiments to demonstrate the statistical concept of the data analysis. The conclusions are robust. The references are appropriate and extensive. This reviewer has no suggestions for improvements. This is an extremely thorough analysis. It is well written with very informative figures: extremely clear and convincing and with very clear descriptions of the methodology.

(Remarks on code availability)
The codes involve straightforward statistical analysis.

Referee #3

(Remarks to the Author)
This manuscript aims to explain the perceived saturation of Earth's polar cap potential drop with an interpretation that has broader implications for interpretation of regression analyses in a variety of settings. A model is presented to encapsulate sources of error or uncertainty in the causal chain from upstream solar wind monitor to measurement of a proxy for the polar cap potential drop (PCI), and some key observed features are reproduced. By performing a calibration based on their error model the observed saturation in PCI disappears and the relationship is found to be linear across the measured range.

On the whole I find much of the paper to be well laid out with reasonable arguments. However, I am not convinced that this work shows that the response to upstream solar wind driving is linear, simply that a reasonable linear model can be constructed that reproduces the approximate observed form. Explaining the apparent saturation with a model that is actually linear in response would support the statement that (line 101) "…there is no statistical evidence for the saturation of geomagnetic response to strong solar wind driving…", though there are questions about the sensitivity of the model to the assumptions made in its derivation that have not been answered in this manuscript. I also have concerns about the meaning of the "magnitude uncertainty" (epsilon) that would seem to invalidate the interpretation of the model for the specific application (not necessarily in general). That is, the work is interesting but is not sufficiently thorough to be as impactful as it could be, and the specific application may not be a good choice for highlighting the regression artifacts. At this time I would not recommend this for publication until the key issues are addressed. Once addressed, I'm still not convinced that the effect described here is actually new, but is instead an interesting exposition in regression modeling. As such, it is not clear that the manuscript would be suitable for Nature, even after revision.

Some more specific comments:
The manuscript starts from a mildly ahistorical position. Starting on line 77 the text states that "[a]s more measurements of rare and extreme solar wind driving accumulated through the years, surprisingly, the correlation between the geomagnetic response and the solar wind driver began to deviate from the linear relation." Prior to this statement the manuscript notes a linear response (citing a recent manuscript by Troshichev et al.), then suggests that the saturation is a "new inference" (line 89) from "more recent measurements" (line 90). The reader is then referred to a table of different hypotheses for saturation of polar cap potential or ionospheric convection. All bar one of these hypotheses have associated citations from the last ~25 years, so could charitably be called recent. I note that the oldest of these is based on an argument presented by Hill et al. (1976) discussing line-tying effect at Mars and Mercury. That is, this is a general physical model not directly based on observations of saturation (apparent or otherwise) at Earth. The supplementary information must be consulted before finding reference to the early work of Reiff et al., where the text states "we observe from the 1980s to the 2000s … as more and more

solar wind data accumulates, the polar cap potential's saturation limit moves from 0.5 mV/m to 10 mV/m". As noted in the accompanying table Reiff et al. found an approximate saturation limit higher than subsequent work by Wygant et al. Similar saturation limits to the work by Reiff et al. were obtained by Russell et al. and Shepherd et al. in the early 2000s. The progression of the apparent saturation limit with time and increased data is not supported by the presented chronology.

One key finding in Reiff et al. (1981) is that "[t]he degree of correlation between interplanetary parameters and the polar cap potential drop turns out to be quite sensitive to the way in which the magnetosheath field B1 is inferred from the (observed) solar wind field Bsw." While this is a rationale for their amplification-limitation procedure in deriving their empirical model, it's important to note the implications. Their "saturated" model is linear once the amplification-limitation has been applied, i.e., the relationship between the sheath driving and the response is linear, but the relationship between upstream solar wind driving and response is not. This is essentially the position taken in this manuscript, though the arguments about how it arises are different.

It is also not clear to me that the term "regression to the mean" is being used in a way that is consistent with standard statistical usage. The description is also not always clear or consistent.
For example, the manuscript states that "regression to the mean is not a statistical phenomenon", when the generally accepted meaning is (as used in Barnett et al., cited by the authors) that "relatively high (or relatively low) observations are likely to be followed by less extreme ones nearer the subject's true mean." As such, it is inherently a statistical phenomenon reflecting the relative likelihood of drawing a sample from the tail of a distribution instead of the bulk of the distribution. The example provided in text, associated with figure 2, was previously published and is not inconsistent with the standard definition and interpretation, so it's not clear to me that there's any support for the position that this is anything other than a statistical effect. The "fundamental logical consequence" referred to on line 201 is noted in terms of likelihood on line 203 and is referring to a stochastic process. Perhaps the implication is that "a statistical phenomenon" is perhaps only an apparent phenomenon, and if something is rooted in reality then it is no longer a statistical phenomenon? Either this interpretation is different and not new (previously published by Sivadas and Sibeck) or it is not different to the historical interpretation of regression to the mean and is instead a more nuanced examination of the impact on specific problems.

On the specific example problem shown in the manuscript: the relationship being shown is that between upstream solar wind measurements and the PCI. The authors note on line 224 that the solar wind is modified through the bow shock and magnetosheath. The so-modified solar wind is what is physically coupling to the magnetosphere, primarily through reconnection. Thus, epsilon in this model includes the effect of shocking the solar wind plasma at the bow shock and any further evolution of this now more-turbulent magnetized fluid. While crossing a shock includes effects that could be considered random, there are also systematic effects on the plasma. The density increases and the bulk speed decreases. The compression increases the magnetic field strength (e.g., the amplification referred to by Reiff et al.). This is no longer strictly a measurement effect and is instead aiming to capture a physical process. That is, even with perfect measurements of plasma at L1 and magnetosheath, and perfect knowledge that they were connected by a streamline, assuming no evolution outside of crossing the bow shock the values of X and X* would still be different. The presented model includes this process implicitly in the epsilon term and then describes it as an uncertainty. This fundamentally impacts the interpretation of the statistical model presented as the argument is that the relationship between PCI and upstream solar wind is really linear and due to measurement uncertainty, while simultaneously rolling the effect of the bow shock on plasma properties into the "uncertainty". Without additional work to clarify this and to distinguish the effects, it appears that this work is entirely consistent with Reiff et al. (1981), but without explicitly capturing the physical processes affecting the evolution of the plasma. How much of the nonlinear relationship, seen here as a conditional bias, is due to a physical process rather than a measurement uncertainty? If this cannot be distinguished then we have a useful tool for empirical modeling, but not a physical explanation that disproves the physical hypotheses for saturation relative to the upstream measurement.

On a related note, the sensitivity of the results to the assumptions in the model are not given. While some justification is given for selected values, such as dt1 and dt2, it is unclear whether the results are sensitive to these values. For example, extended data figure 4 shows a comparison of the modeled PDF to the empirical PDF and the caption states that it "predicts the pdf … almost exactly". There are clear deviations and differences in shape, and while the correspondence is good, it is far from exact and is presented with no uncertainties or testing for similarity to the observed distribution. How much of the correspondence can be tuned by selection of different values for dt1, dt2, or epsilon? The same requirement would apply to extended data figure1, though that is not claimed to be an "almost exact" match.

I note also that Nature requires that all uncertainties should be defined in the figures. While presumed confidence intervals are shown in multiple figures (e.g., figure 4), they are not described in the figures or figure captions. The text is further insufficient to properly describe the confidence intervals.

The software used (per inspection of the provided code) is not cited or acknowledged in the manuscript.

(Remarks on code availability)
I have read the code, but have not attempted to run any of it. Some comments based on that reading follow.

Code comments include a request to not share without permission, which is at odds with the provided license.

The data files for the Python code appear to be provided as "pickles", which may not be platform-independent. This file type also can have implicit version requirements for dependencies that are not clearly spelled out in a readme.

A Dockerfile is provided so that this should be reproducible, but rebuilding images from a Dockerfile can result in different

dependency stacks.

Version 1:

Reviewer comments:

Referee #1

(Remarks to the Author)
I have read through the reply letter and the revised manuscript. I think that the authors have made excellent work to validate their analyses and polish the article with more important details and more convincing results.

I agree with the statement in the article that the regression-to-the-mean effect is not an alternative theory competing with the existing theories of saturation but a demonstration for further validation of the latter. This conclusion has important implications in both space sciences and various other fields.

I would like to recommend the acceptance of the manuscript at the present form. Meanwhile, I will draw the attention of the authors in the following aspect, which may be of interest to future investigation: there exists the coupled nature of the magnetosphere-ionosphere-atmosphere system under the impact of solar activity. How to deal with this coupling complexity so as to accurately quantify the regression-to-the-mean effect can be intriguing.

(Remarks on code availability)
The code provides a very useful aid to understand what the authors have done to deal with correcting the effect of uncertainties in association with measurements of solar wind driving and geomagnetic responses of Earth.

Referee #3

(Remarks to the Author)
This manuscript aims to explain a likely common source of non-linear bias in regression analyses, specifically applied to the case of strong solar wind driving of Earth's magnetosphere, exemplified by the response in polar cap voltage (or proxies thereof). I appreciate the thoroughness of many of the authors' responses. Some of my concerns remain unaddressed or inadequately addressed and these are discussed below. My overall finding is that the manuscript is both interesting and useful but is overstating its novelty and is occasionally overly confident or narrow in context in the service of telling a coherent story. Whether this manuscript is suitable for Nature is clearly an editorial decision, but I believe the work would be much more compelling and impactful if the uncertainties and sensitivities were adequately explored and this has only been partly addressed in the revisions.
I will limit my comments to my previous comments and the responses, so items will be identified by the numbers used in the author responses.
3.0.1 – I find the argument based on the fraction of data displaying saturation uncompelling. There are numerous physical examples of saturation that represent a change in real behavior when approaching driving that exceeds the norm. For example, in MOSFET (field effect transistors) there is an approximately linear response to applied voltage until a saturation limit. Whether a saturation limit is in the regime of typical driving or extreme driving is irrelevant to whether or not there is a real saturation effect. The argument that linearity in part of the parameter space implies that our expectation should be that it remains linear is not supported. Please note that I am explicitly not saying that I believe there is saturation in the magnetospheric driving, just that the argument here is inadequately supported. In the manuscript the authors show that a regression bias should exist, but the extent to which that invalidates the previous studies showing saturation is not sufficiently demonstrated. This was the central point in my previous review.
3.0.2 – Yes, the authors correctly point out that the model presented is a model resulting in a non-linear bias. I had intended this as a description of an additive (i.e., linear) model, which does not necessarily require that the relationship between predictor and outcome is linear. It is also true that the "true" driver and outcome have a linear relationship in the presented model. Certainly my phrasing was poorly chosen and ambiguous, however, the main point was that the model presented can approximately reproduce the form of observed saturation and that is a necessary but not sufficient condition for claiming that the saturation is due to nonlinear bias. The comment continued to then question whether sufficient evidence had been presented to establish that the outcome was robust to changes in the estimated parameters.
3.0.3 and 3.7 – The authors claim that the inputs to the error model are constructed directly from data and measurements, but it's not clear that this is completely supported in the manuscript. I appreciate the addition of extended data figure 4, which goes some way to addressing the gap. The response states that the assumptions used are derived directly from measurements, which is true only if "derived" is used to mean "based on". The response further states that the model results are validated "making a sensitivity analysis superfluous". I disagree on this point. Showing qualitative agreement is a useful validation (though I note it is not quantitative) but it would still be important to understand how robust the result is to differences in the assumed parameters as this is not addressed by the presented validation.
The claim that the assumptions in the error model are derived directly from measurements is not entirely supported. The dt1 estimate is based on a single paper and the chosen distribution and parameters are not explained. Similarly, the estimate of dt2 cites a couple of papers, but is light on justification of the specific distribution and parameters. On line 525 the authors state that "[historically], researchers have used a constant propagation delay (t2) of about ~20 minutes" without support. The support is better for the magnitude uncertainty, but separating the data-derived quantities from the assumptions should be clearer. Finally, the authors note that "the statistics become poor" at high values of sheath electric field, but no estimates of uncertainty are given. Were this review for a different journal I might be inclined to just treat these as assumptions and recommend softening the language somewhat to not draw the conclusions in such a strong fashion. Given that this is for

Nature, I feel that the bar should be higher than it has been drawn in this manuscript.
3.0.5.2 – The explanation of how the saturation effect impacts this specific problem is new. There is, of course, a sliding scale of novelty and this manuscript does have genuinely novel aspects. Whether this is sufficiently novel to justify a paper in Nature is the context for the previous comment, and my opinion appears to be different from the other reviewers. However, while I agree with the authors' statement that " even if one believes that this insight is not new, it is important to point out …", it is again in the context of this submission being to Nature that I'm not convinced that "important to point out" warrants publication in Nature as opposed to the plethora of other respectable journals. However, that is clearly an editorial decision.
3.0.6 – The authors have perhaps over-interpreted this comment. My use of the Reiff result was simply illustrative and not intended to be a sole point of comparison. I also agree with the authors that the specific result is new and likely impactful (though the degree is debatable). I disagree that the authors have shown that the general result is particularly new though it is, again, useful.
3.2 – Making a testable prediction is indeed necessary, but being testable and being tested are two different things. Interpretation of the Hill paper as "clearly" arising "from some observational inference" is a possible interpretation, but the paper is not presented that way. The inference, if it was a motivating factor, could have been based on physical intuition (i.e., drawn from understanding of the physics underlying the presented model) with a throwaway reference to unshown data being added later. I find this to be an overstatement designed to tell a simple story rather than embracing the nuance.
3.3 – New extended data figure 5 is problematic. The inclusion of Boyle et al. with a saturation limit despite their explicit statement that they did not see evidence of saturation is misleading. The line to guide the eye is also misleading. Note that the studies from 2002-2005 span the range of 3-10 mV/m, and no trend is evident. In the spirit of quantitative support, I calculated the linear correlation for the displayed points (with and without the Boyle study) and found that while the correlation was approximately 0.6 in both cases, the confidence intervals on the correlation included zero. Thus showing the trend line without noting that it's also consistent with no trend is misleading.
The Boyle study is also interesting in this case. Given that the nonlinear bias should be present in any such study, how did their data selection avoid the apparent saturation seen in other studies? Other questions that arise here include the data sources. Presenting this uncritically as an increasing trend sidesteps the natural question of how much the present model explains of the apparent saturation in previous studies.
3.4 – Again, the authors' summary of my comment is not consistent with my intent (or, I believe, with the full comment). The quote I provided from Reiff et al. was, in fact, the key rather than the specifics of how they dealt with it. That is, they identified that the relationship between interplanetary drivers and the polar cap voltage was sensitive to the way in which the sheath field is inferred from the solar wind field. The conclusion was that the observed relationship between upstream solar wind and polar cap voltage seemed nonlinear, even though the relationship between the proximate driver (sheath field) and the response is linear. That is one of the insights presented here that certainly has not been emphasized in other literature to my knowledge. They obviously did not explain that in the same way as the authors do in the present manuscript. I noted in my comment that Reiff et al. explained this using a physical process which is clearly different from the present explanation. What I was pointing out is that the authors present the fact that a linear relationship between sheath field and response can show a nonlinear relationship between upstream field and response as being a new insight rather than noting that this was already posited by Reiff et al and thus this provides an explanation for their realization. The response claims that any theory involving reduction in dayside reconnection rate claims this position, but I do not recall this being clearly stated in other literature and I do not see this being given as context for the present work. My concern here is in the accurate presentation of prior literature, sufficient credit where due for prior work, and a clear delineation between the contributions of this work and what has come before.
3.5.1 – The authors' response here presents a number of useful descriptions of the phenomenon, but fundamentally I believe that the disagreement here is in what "statistical" means. I am likely to not be alone in interpreting each of the five vantage points as being statistical in nature. As anyone who has spent significant time with measurement knows, the measurement is not the "truth". Even a single count of a cosmic ray in a detector is biased in a way that can be explained by Poisson statistics - yes, it's more complicated than that, but the point is that being a fundamental issue not tied to any specific method does not mean it's not a statistical effect, at least not in the way that the term is widely understood. The authors' own explanation relies on "the stochastic properties of the truth, the degree of uncertainty of the measurement" (which would widely be interpreted as being statistical) and while this is important to highlight and may be valuable to bring to a wider audience, it's not a new insight.
3.5.2, 3.5.3 – As noted in my previous comment, I do not believe that this interpretation is strictly accurate. A new description of something that is known can be valuable but does not make the result new. The "new vantage points" are useful, if broadly equivalent, descriptions. But they do not represent new insight, just a nice presentation of insights already known.
3.6.1 – The authors' response is clearly correct, as they have defined the term to be zero mean. I am not entirely sure what I was intending to question (since the model term is self-evidently zero mean), or whether it was a misunderstanding, but certainly the presence of any bias in reality could be confounding to the modeling and may represent an unexplored source of bias. However, I also appreciate that the core point of the manuscript is that even with no real bias there is an apparent bias.
3.6.2 – The response here is perhaps stronger than can be supported by the presented evidence. It doesn't show that the underlying data show no saturation. It shows that a nonlinear bias arising from nonlinear error can display a similar saturation effect. The degree to which the saturation arises from the regression to the mean effect (i.e., what fraction of the apparent saturation is due to this effect, which obviously depends on the assumed dt1, dt2 and epsilon) is important to clearly characterize to show that this invalidates, rather than modifies, the previous studies showing saturation. The sensitivity plots help to show that this can, for given assumptions, give a degree of saturation entirely consistent with the data. They also show that the result could indeed be sensitive to those assumptions and the difference (at least to me) between this being a Nature paper or being published elsewhere lies in showing that the result holds for a reasonable and justifiable range of parameters.
3.8.1 – Noting the difference in probability density is not a quantitative measure of uncertainty. There are numerous methods to assess correspondence, and even seeing similar qualitative results for different assumptions would go a long way to

addressing the robustness of the result. While the authors have drawn attention to the log scale potentially highlighting small differences in the tail, I note that this also minimizes the much larger differences at higher probability density.
3.8.2 – As discussed earlier, the input parameters are not entirely “calculated directly from data”. While the paper does not show any tuning of parameters, that was not the question asked. If the assumed parameters of the error distributions are changed, given that many of them are loosely derived, how does the result vary. It may well be that the result is relatively insensitive in a reasonable range of parameter-space or assumption-space, but this is not clear and is somewhat important to showing that the presented explanation is indeed a full explanation and not just an explanation that reduces the amount of saturation. Figure AR-4 does indeed show some of this although it is not directly connected to the degree of apparent saturation expected. I disagree that this is beyond the scope of this work and while I welcome the authors’ intent to address this in some future paper, I also believe that it is necessary for establishing the impact of the presented work in an unbiased manner. I would expect that this is explored to some extent in a Nature paper to justify the publication in Nature.

(Remarks on code availability)

Version 2:

Reviewer comments:

Referee #3

(Remarks to the Author)
The authors have substantially addressed my comments with the inclusion of the additional discussion regarding the estimates of dt1 and dt2, as well as the additional support from the figures included in the response (Figure AR-6 especially). The revisions to the text associated with these responses has provided good additional support. I have a few remaining comments below that I would like the editor and/or authors to consider that I believe would strengthen this work.

I would still caution against overly strong statements throughout the work that are not fully supported. For example, the abstract states that "we demonstrate that the saturation is a manifestation of the regression to the mean effect arising from random uncertainty in the timing and magnitude of solar wind measurements" while on page 37 the authors say "the regression to the mean effect is the cause for the saturation of the polar cap index with solar wind driver value measured at L1". The authors have demonstrated that an apparent saturation can be explained by this effect, and with the current revision that the expected degree of apparent saturation is close to that observed and is reasonably robust to the assumptions, not that the saturation (i.e., all observed saturation) explicitly is a manifestation of this effect.

Regarding the authors' assertion that the model is not additive (ARv2 3.0.2.1): As written the model is an additive model ($X^* = X + e$, or $X^* = X(t+dt1+dt2) + e$), and this remains true if the systematic terms and error term do not interact, even if those terms are nonlinear. A heteroscedastic error term does not necessarily imply that the model is not additive. If the underlying model is not additive then the presentation masks the interaction terms and the interaction should be made explicit so that the reader does not see an additive model. While considering this it also occurred to me that the model could be phrased as an example of Jensen's inequality, where near a peak the variation is locally concave and thus, due to uncertainty, the measured value would be biased downward, and vice versa for troughs. This is then the regression to the mean described (although without reference to Jensen's inequality) and implies that the error is dependent on the convexity/concavity (second derivative) of the data over any given interval. This would also provide a simple conceptual explanation for the results of Boyle et al., since the convexity/concavity of each interval studied should be net close to zero and thus the bias will be close to zero. Providing a conceptual explanation for the Boyle result within the same framework as the explanation for apparent saturation increases the support for the work.

(Remarks on code availability)

Open Access This Peer Review File is licensed under a Creative Commons Attribution 4.0 International License, which permits use, sharing, adaptation, distribution and reproduction in any medium or format, as long as you give appropriate credit to the original author(s) and the source, provide a link to the Creative Commons license, and indicate if changes were made.
In cases where reviewers are anonymous, credit should be given to 'Anonymous Referee' and the source.
The images or other third party material in this Peer Review File are included in the article's Creative Commons license, unless indicated otherwise in a credit line to the material. If material is not included in the article's Creative Commons license and your intended use is not permitted by statutory regulation or exceeds the permitted use, you will need to obtain permission directly from the copyright holder.
To view a copy of this license, visit https://creativecommons.org/licenses/by/4.0/

Response to Referees 1

Referee 1 1
Referee 2 7
Referee 3 8
Some more specific comments: 11
List of changes from Initial Submission to Revision v1 29
List of editorial changes from Revision v1 to Editorial v2 32
References 33

Appended with this document are the following:
Appendix 1: Revision 1 with Markup 34
Appendix 2: Editorial 1 with Markup 86

To the Editor and Referees:

The following is our response to the reviewer's comments. For every comment, we identify the essence and summarize it next to the label **RC** (Reviewer's Comment) and then provide a summary of the response **AR** (Authors' Response). Following that we add a more detailed response where necessary. We follow this format for the ease of both the Editor and the Reviewers.

A list of revisions is provided under "List of changes from Initial Submission to Revision v1". These changes are also provided below next to the label Revision, when a revision is necessary. The line numbers and section numbers provided in this letter are associated with the file in **Appendix 1**, which is the version of the revised paper with markup before editorial changes were made. The final form of the article contains editorial changes in which a lot of text has been moved to new supplementary sections, without any changes made in the content. These changes are listed in "List of editorial changes from Revision v1 to Editorial v2", and the associated changes are shown in a markup version of the final article shown in **Appendix 2**.

Referee 1

Referee #1: solar wind, radiation belt observations

Review of "Regression to the Mean of Extreme Geomagnetic Storms"

> The authors highlight in this significant study a regression-to-the-mean effect of Earth's responses to extreme solar wind driving. As investigated in the study, this effect is argued as the major cause for the commonly accepted saturation effect of the Sun-Earth response link. Meanwhile, this effect is also regarded to cover up a linear throughout response of Earth's environment to solar wind driving especially under extreme circumstances, which the authors think is more likely to be the reality. While regression to the mean is a common concept in the field of mathematics and statistics, this study is of prime significance because it is the first one so well detailed, both from the data side and the analyzing one, to demonstrate that random uncertainty in solar wind measurements may fundamentally impact current understanding of the Sun-Earth energy transfer system. It is clearly concluded that extreme geomagnetic storms may have twice the impact on Earth than previously thought, which goes against the commonly accepted view that a saturation level appears to exist for Earth's responses to extreme

solar wind driving. This discovery is of significant interest to the entire space science community, including engineers, experimentalists, data scientists, modelers, and theorists, when they cope with the sophisticated system of Sun-Earth connection and the devastating space weather effects. Furthermore, it should bear broad impact and be of much interest as well to those studying random, uncertain measurement systems and their underlying impacts on various aspects of our society and beyond.

Before I can recommend the paper for acceptance, I have a number of specific comments and remarks that I would like the authors to address.

1. The first one concerns the statistical analysis of solar wind measurements and polar cap indices. As the authors mentioned in the paper, 25 years of WIND data and the polar cap indices from the same time range are adopted for the investigation. Then how to deal with such a large database is key to the research purpose. I would have appreciated to see a proper description regarding the data analysis procedure and the distribution statistics of both solar wind driving and polar cap/geomagnetic activity at different levels. On one hand, the results presented in the paper can be further justified and also become reproducible to readers. On the other hand, the spread of the variables can be easily recognized for better perception of the regression-to-the-mean effect between the measurements and the truth, especially under extreme conditions.

RC 1.1
Present a proper description of data analysis procedure and distribution statistics of solar wind and geomagnetic activity at different levels.

AR: Highlight existing description of data analysis procedure and distribution, create a new supplementary methods section 2.c describing the data analysis presented in the code.

Revision:
3.c Adding reference to new supplementary section “Supplementary Methods 2.c summarizes the full computer code used.” (Line 668)
3.d.iii Added new Supplementary Methods 2.c Summary of data analysis procedure in the computer code (Line 1221-1306)

Due to the word limit, we have focused on presenting the minimally necessary evidence and analysis to support the main conclusions of the paper. Line 569 to Line 712 in the Methods section presents a summary of the full data analysis, more details are presented in the attached code which includes a very detailed description of the procedure. However, based on the reviewer’s comments we understand that a reader might wish to learn more details. Hence, we have now added an additional supplementary methods section 2.c summarizing the data analysis procedure followed in the code - which further helps with reproducibility. In Line 668 we reference this section in the Methods as follows: “Supplementary Methods 2.c summarizes the full computer code used.” We further note that our code is uploaded on CodeOcean as a capsule, access to which was provided to us by Springer Nature. The capsule will be published along with the manuscript and allow anyone to carry out a reproducible run online through the CodeOcean system.

The distribution of the solar wind driver function (E_m) and the PCI index, its autocorrelation coefficient, are presented as the black line (i.e., Data) in Extended Data Fig 1 (New Extended Data Fig 1a), Extended Data Fig 2 (New Extended Data Fig 1b), and Extended Data Fig 3 (New Extended Data Figure 1c). We also show how the conditional probability distribution of the geomagnetic activity changes with increasing strength of the solar wind driving in Figure 3a. Furthermore, in the

Supplementary Methods 2.c. we refer the readers to Voros et al., 2015 and Sivadas & Sibeck, 2022 for further details of Solar Wind parameter distributions, and Borovsky, 2021, 2022a, 2022b for distributions and behavior of geomagnetic activity response.

2. The second point relates to the way correcting the effect of uncertainties. As stated in the paper, the adopted method includes statistically quantifying the uncertainties, predicting their effect and eventually offsetting them from the inference. The Methods section also outlines explicitly three primary sources of the random uncertainty to deal with in the study. One essential step is to develop an error model to assess the non-linear bias associated with the measurements of solar wind driving. Then how do the authors justify the rationality of such a model and the calculated bias connected to different sources of the uncertainty? For instance, does it depend on solar wind driving condition or the regression analysis/calibration methodology? Furthermore, is it possible, based upon conjunction observations from multiple satellites (after all we have to more or less rely on these in situ measurements), to verify how reliable these analysis estimates can be.

RC 1.2
What is the justification for the specific error model used, and can its reliability be verified?

AR: The error model is logically the simplest error model whose reliability is verified by the similarity between the second-order statistics observed from simultaneous measurements of solar wind and geomagnetic activity response and the error model input and output. Supplementary methods section 2.a: "validation of the error model" shows the rationale for using the specific error model.

Revision:
8. Added phrase to indicate that validation of the error model also provides the rationale for using it: "This validation ~~are~~is the rationale for using the error model and is presented in the Supplementary Methods Section 2.a."

Space physics studies up to this point implicitly use the following error model

$$X^* = X(t + dt) + \epsilon$$

where $dt = 0$ and $\epsilon = 0$. This model has no good rationale, as we have shown in our manuscript. However, when appropriate values derived from observations are given to dt and ϵ, then for a specific true value X with a probability distribution, the error model will output a value for the erroneous measurement X*. Once you have this result, we have the task of evaluating whether the resulting X* and its properties are consistent with data. Validity is established if the probability distribution of X* matches the probability distribution of solar wind driver measurements at L1 (Extended Data Fig. 4/ New Extended Data Fig. 2a, the figure is in log-scale so any difference in probability values is less than 0.1). Furthermore we check for consistency in second-order statistics, like the standard deviation of the difference in the true value and measurement (X*-X), which can be compared with the standard deviation of the difference between E_m^* (the measure of solar wind driver estimate at L1) and E_{PC} (estimate of the shocked solar wind driver/ polar cap index) i.e., Extended Data Fig. 5/ New Extended Data Fig. 2b. An even better method of verifying reliability is available through the comparison of the conditional pdf of the same, shown in Extended Data Fig. 6/ New Extended Data Fig. 2d-e. These reliability tests are described in Supplementary Methods Section 2a, line 1056-1096. Hence, we answer

in the affirmative that *it is possible based on conjunction observations from either multiple satellites or ground-based measurements and their second-order statistics to verify the reliability of the error model.* The reason is that first-order statistics like the mean, can be influenced by physical phenomenon (or physical biases as we described in Line 134-145), however the second order statistics is influenced by random variations and uncertainty. If the random variations observed in the measurements can be reproduced by our error model based on calculated uncertainty estimates, then the regression bias it predicts is reliable.

If our error model is incorrect, we will observe that the second-order statistics from the model output will be less consistent with data. The corollary of this is that a more improved error model can be constructed, which will have an even better agreement in its second-order statistics (or even higher-order statistics like the conditional probability distribution function) with that of the simultaneous multi-satellite/ground-based measurements.

We thank the reviewer for raising the question, as we realize now that the phrase 'validation of the error model' should be supplemented by another phrase 'rationale for using the error model', so that readers understand that they are the same. We modify line 666-668 to reflect this: "This validation is the rationale for using the error model and is presented in the Supplementary Methods Section 2.a."

3. Figure 4 shows Earth's responses to solar wind drivers after correcting the regression bias in the measurements. It also presents no data available beyond 15 mV/m after the correction. One reason can be the data absence in this quantity range. But it is natural to think what will the relationship look like with (Em)^c > 15 mV/m, say, very extreme solar wind driving conditions. To be even further, extreme conditions not just include super strong solar wind driving but also substantially quiet solar activity. How about the Earth's responses under such extremely quiet solar wind driving conditions? Does it keep a linear property or have the non-linear effect? It can be very informative to have a look at these conditions or at least give some discussions.

RC 1.3.1
What does the geomagnetic response look like beyond E_m^c>15 mV/m?

AR: We do not currently have statistical evidence to determine what the geomagnetic response will look like beyond E_m^c > 15 mV/m, however, we may expect the initial assumption of linearity to continue until the drag force of the ionosphere plasma equals the shocked solar wind convection (Vasyliunas, 1975).

The reason for lack of data beyond E_m^c>15mV/m is the rarity of stronger solar wind driving. With more time and data in hand, future space scientists will record stronger geomagnetic storms. Though they will also be subject to the regression-to-the-mean effect, they will add more data to the available statistics, and we will be able to uncover the nature of the geomagnetic response beyond 15mV/m. However, until then we have no choice but to state that we do not have statistically meaningful data to extend the relationship beyond 15mV/m.

Nevertheless, when theorists develop a physical theory based on the corrected data, such a theory will be able to predict the geomagnetic response beyond 15 mV/m. We believe that the initial assumption that solar wind driving maps the shocked solar wind convection electric field to the polar cap, and hence creates a linear response in the polar cap potential to driving is likely true. And hence a linear relationship will continue until ionospheric plasma drag becomes strong enough to oppose the shocked solar wind convective force (Vasyliunas, 1975).

RC 1.3.2
How does geomagnetic activity look like for “extreme” quiet solar wind conditions?

AR: Regression to the mean effect is particularly pronounced for extreme strong solar wind conditions, because they are *rare* values. In contrast, extreme quiet solar wind driver values are very common and hence regression to the mean effect is imperceptible in that regime. Therefore, for “extreme” quiet solar wind (driver) conditions the geomagnetic response will be linear.

4. Since quite a large number of variables have been introduced in the text to refer to different parameters in association with solar wind driving, polar cap indices, geomagnetic activity, etc., which can be confusing to and difficultly followed by readers, a suggestion is that the authors can produce a table in the Supplementary to list and describe clearly them.

RC 1.4
Producing a table in the Supplementary to list and describe the different variables will help the reader.

AR: We have added a new extended data table as per the reviewer’s suggestion.

Revision:
7. Added reference to new Extended Data Table 2: Symbols used in the manuscript. “The most common symbols used are explained in the Extended Data Table 2.” (Line 465-466)
15. Added New Extended Data Table 2| **Common symbols used in this work.** It contains a list of all the commonly used symbols in the manuscript and their description. (Line 1421-1428)

Symbols	Description	Symbols	Description
E_m^*	Solar wind driver, Kan-Lee electric field, merging electric field, measured at L1 Lagrange point and time-shifted to bow-shock	X^*	Measurement, Model counter part of solar wind driver measured at L1 Lagrange point and time-shifted to bow-shock
E_m^{sh}	Shocked solar wind driver measured in the magnetosheath close to the subsolar point near the magnetopause	X	True value, Model counter part of shocked solar wind driver close to the subsolar point in the magnetosheath
E_{sw}	Solar wind convection electric field	ϵ	Zero-mean random error, magnitude uncertainty
E_{PC}	Dawn-dusk convection electric field across the polar cap	Y_{PC}	Model counterpart of dawn-dusk convection electric field across the polar cap, also counter part of PCI
E_m^c	Calibrated solar wind driver	X^c	Calibrated solar wind driver
E_R	Reconnection electric field	b	Regression bias (due to regression to the mean effect)
PCI	Polar cap index, also a proxy for E_{PC}	Y	True value, model counter part of geomagnetic response
PCN	Polar cap index derived from a magnetometer in northern polar cap	Y^*	Erroneous measurement of Y
PCS	Polar cap index derived from a magnetometer in the southern polar cap	dt	Uncertainty in the time of measurement
SML	Westward auroral electrojet index derived from SuperMAG network of ground magnetometers	dt_1	Uncertainty in solar wind propagation delay from L1 to bow shock nose
$B_{T,sw}$	Transverse magnitude of solar wind magnetic field	dt_2	Uncertainty in propagation of the effect of solar wind from bow shock to the polar cap ionosphere
B_z	Magnetic field along the Z-axis of the GSM coordinates	σ_δ	Standard deviation of random uncertainty in time of measurement
B_{sh}	Magnetic field value in the magnetosheath	k	Autocorrelation time constant
V_{sh}	Plasma velocity measured in the magnetosheath	V_{sw}	Solar wind plasma velocity
θ'	Shear angle between the magnetic field vector in the magnetosheath and the magnetosphere at the magnetopause	θ_{sw}	Solar wind magnetic field clock angle

Extended Data Table 2 | Common symbols used in the paper and their description. Variables representing measurements and their corresponding counter parts in the error model used commonly through the paper are summarized here.

5. Figure 2 is a reproduction by combining Figs. 1-3 of Sivadas and Sibeck (2022). Since this is a submission to Nature, it is suggested to replace Figure 2 if available.

RC 1.5
Replace figure 2 if available.

AR: The authors have full copyright to the figure, and have the permission to copy or modify it through the CC BY 4.0 License with an acknowledgement to Sivadas and Sibeck (2022). We add the following attribution at the end of the figure 2 caption: "This figure is adapted from Sivadas & Sibeck 2022, used under CC BY 4.0."

Revision:
6. Added copyright information under Figure 2: ". ~~Figure reproduced~~This figure is adapted from Sivadas & Sibeck 2022[22], used under CC BY 4.0." (Line 181-182)

Referee #1 (Remarks on code availability):

The code provides a very useful aid to understand what the authors have done to deal with correcting the effect of uncertainties in association with measurements of solar wind driving and geomagnetic responses of Earth.

AR: We thank the reviewer for appreciating the usefulness of the code.

Referee 2

Referee #2: measurement biases in solar wind physics, simulations of saturation of geomagnetic response

Referee #2 (Remarks to the Author):

This manuscript describes an alternate (and believable) explanation for a long-standing problem in magnetospheric physics and how the space-physics community improperly interprets the data pertaining to this problem. For decades the community has come up with a number of physical explanation for the "problem", but the analysis of the authors demonstrate that in fact there is no "problem". This research is very original and it is significant for magnetospheric physics and for other statistical problems in other fields of science and medicine. The methodology is correct and the data is excellent.

The authors also use numerical experiments to demonstrate the statistical concept of the data analysis. The conclusions are robust. The references are appropriate and extensive. This reviewer has no suggestions for improvements. This is an extremely thorough analysis. It is well written with very informative figures: extremely clear and convincing and with very clear descriptions of the methodology.

Referee #2 (Remarks on code availability):

The codes involve straightforward statistical analysis.

AR: The authors are grateful to the referee for highlighting the manuscript's strengths. While incorporating the editorial changes, we have taken care to preserve the clarity of the descriptions in the Main Text and Methods sections that the referee noted, while moving some discussions to the Supplementary section.

Referee 3

Referee #3: uncertainties in the solar property inference from L1 to magnetosheath

Referee #3 (Remarks to the Author):

This manuscript aims to explain the perceived saturation of Earth's polar cap potential drop with an interpretation that has broader implications for interpretation of regression analyses in a variety of settings. A model is presented to encapsulate sources of error or uncertainty in the causal chain from upstream solar wind monitor to measurement of a proxy for the polar cap potential drop (PCI), and some key observed features are reproduced. By performing a calibration based on their error model the observed saturation in PCI disappears and the relationship is found to be linear across the measured range.

On the whole I find much of the paper to be well laid out with reasonable arguments. However, I am not convinced that this work shows that the response to upstream solar wind driving is linear, simply that a reasonable linear model can be constructed that reproduces the approximate observed form.

RC 3.0.1
"I am not convinced that this work shows that the response to upstream solar wind driving is linear."

AR: 95% of all available data points in 25 years follows a linear relationship according to our simple regression analysis (See line 516-519 and AR-Figure 1 bottom-right panel) and as shown by all studies of polar cap saturation in New Extended Data Figure 5. The data that shows the non-linear response or saturation is less than 5% of the total data. If one is convinced that for 95% of the data, the geomagnetic response is linear, then our manuscript suggests that before concluding that the 5% outliers imply a saturation effect, we must quantify the random error in the solar wind driver input and correct for the regression bias that it creates. Once we carry out this correction, the outliers continue the linear relationship that 95% of the data already shows.

RC 3.0.2
The work shows "simply that a reasonable linear model can be constructed that reproduces the approximate observed form."

AR: It is important to clarify that this work does not present any linear model. It presents a non-linear error model, which takes as input the stochastic properties of the solar wind driver and quantifiable random error in time and magnitude, and produces as output the bias in the shocked solar wind driver estimate that actually drives the geomagnetic response. This non-linear bias when corrected from our analysis, reveals a linear geomagnetic response directly from the data without any need to construct a linear model. If the random error values were lower than our estimates, then the corrected data will not show a geomagnetic response that is linear, it will have some non-linearity.

Explaining the apparent saturation with a model that is actually linear in response would support the statement that (line 101) "…there is no statistical evidence for the saturation of geomagnetic response to strong solar wind driving…", though there are questions about the sensitivity of the model to the assumptions made in its derivation that have not been answered in this manuscript.

RC 3.0.3
Questions about the sensitivity of the model to the assumptions made in its derivation have not been answered in this manuscript.

AR: The inputs to this error model are constructed directly from data and measurements. As a result, we show a validation of the error model (Line 661-671, and Supplementary Methods Section 2.a). A sensitivity analysis is also presented in Figure 3c and is also analytically calculable using Equation 1.21 that we have derived analytically in this manuscript (Supplementary Methods Section 2.b) for the first time. Furthermore, we have now added New Extended Data Figure 4, that presents a similar sensitivity analysis to Figure 3c, but for magnitude uncertainty. Finally, we have also responded to the reviewers concerns regarding the sensitivity analysis in RC 3.7 below. A more complex and detailed sensitivity analysis, for example, how the different functional forms of heteroskedastic uncertainty affect the regression bias, can be presented in a future manuscript, but its not essential for the results presented in this paper.

Revision:
9. Adding references to figures that provide sensitivity analysis: "Additionally, we show a sensitivity analysis of the non-linear regression bias, which increases with increasing uncertainty in the time of propagation (See Figure 3c), and with increasing uncertainty in the magnitude uncertainty (See Extended Data Figure 4)." (Line 669-671)
12. New Extended Data Figure 4 added as sensitivity analysis of magnitude uncertainty. "Extended Data Figure 4| **Sensitivity analysis of magnitude uncertainty.**"

I also have concerns about the meaning of the "magnitude uncertainty" (epsilon) that would seem to invalidate the interpretation of the model for the specific application (not necessarily in general).

RC 3.0.4
Magnitude uncertainty has rolled into it systematic effects from the physics of the bow-shock.

AR: We provide below (in RC 3.6.1) 3 different proofs that demonstrate that the magnitude uncertainty is a purely random error with zero mean, and no systematic effects are rolled into it. Hence, this does not invalidate our conclusions or its interpretation.

That is, the work is interesting but is not sufficiently thorough to be as impactful as it could be, and the specific application may not be a good choice for highlighting the regression artifacts.

RC 3.0.5.1
"Specific application may not be a good choice".

AR: The geomagnetic response saturation is a fundamental and long-standing problem in space physics. The application of the regression to the mean effect to resolve this problem is a very good choice for two reasons: 1. It overturns our current understanding of the geomagnetic response, i.e., it shows that is likely a linear response instead of a saturating response. Hence it has implications in a large number of studies within space physics and space weather forecasting. 2. It demonstrates the significant impact the regression to the mean effect might have on our inference from data, therefore highlights its importance to other fields.

At this time I would not recommend this for publication until the key issues are addressed. Once addressed, I'm still not convinced that the effect described here is actually new, but is instead an interesting exposition in regression modeling. As such, it is not clear that the manuscript would be suitable for Nature, even after revision.

RC 3.0.5.2
"Once addressed, I'm still not convinced that the effect described here is actually new".

AR: In RC 3.4 and RC 3.6.1,3.6.2,3.6.3, we show that our explanation of the saturation effect is new and has not previously been shown i.e., the phenomenon results from the regression to the mean effect due to random error. This space physics result calls into question several theories of saturation, and hence is of substantial impact. Therefore, it will bring the wider scientific community's attention to the regression to the mean effect, which is often missed or ignored. Furthermore, we show in our responses, that our finding that regression to the mean effect is a property of the relation between measurement and truth, and not only a methodological effect, is new and useful - as it reveals that the effect is ubiquitous, and will manifest in many different methods, single measurements, and also entirely different fields. Finally, even if one believes that this insight is not new, it is important to point out that the regression to the mean is widely misunderstood and also unknown to many physical scientists.

RC 3.0.6
"It is not clear that the manuscript would be suitable for Nature, even after revision".

AR: We understand that the primary reasons for the above comment is the possibility that : 1) The space physics result is not novel, because the magnitude uncertainty contains physical effects, and hence reproduces the same results as those presented by Reiff et al., 1981. 2) The general result that regression to the mean effect is a property of the relation between truth and its corresponding measurement is already presented by Sivadas and Sibeck, 2022, and hence it is not novel. In our responses (In RC 3.4, 3.6.1, 3.6.2, 3.6.3, and RC 3.5.1, 3.5.2, 3.5.3), we have clarified that neither of the above possibilities are true, and instead our space physics result is new and impactful, and the general insight is also new and useful as it shows the ubiquitous nature of the effect. Given that we have addressed both concerns of novelty, we hope that the reviewer will reconsider this position as we submit the revised manuscript.

Some more specific comments:

The manuscript starts from a mildly ahistorical position. Starting on line 77 the text states that “[a]s more measurements of rare and extreme solar wind driving accumulated through the years, surprisingly, the correlation between the geomagnetic response and the solar wind driver began to deviate from the linear relation.” Prior to this statement the manuscript notes a linear response (citing a recent manuscript by Troshichev et al.), then suggests that the saturation is a “new inference” (line 89) from “more recent measurements” (line 90). The reader is then referred to a table of different hypotheses for saturation of polar cap potential or ionospheric convection. All bar one of these hypotheses have associated citations from the last ~25 years, so could charitably be called recent.

RC 3.1
The manuscript suggests the saturation is an inference based on “recent” measurements, while the hypothesis surrounding it can be called recent only charitably.

AR: The authors use the phrase “more recent measurements”, as these measurement are *more recent* than the initial measurements by Troshichev et al. 1979, which inferred a linear relationship between solar wind driver and geomagnetic response. Since “recent” is a subjective term, we use “more recent” as a relative term, relative to a previous time frame when the first hypothesis of polar cap potential varying linearly with solar wind driving developed in the field. Since the referee has revealed to us a potential misunderstanding while using this term, we reword this phrase “the more recent measurements” as “later measurements”.

Revision:
4. Modified the phrase “the more recent” to “later”. (Line 92)

I note that the oldest of these is based on an argument presented by Hill et al. (1976) discussing line-tying effect at Mars and Mercury. That is, this is a general physical model not directly based on observations of saturation (apparent or otherwise) at Earth.

RC 3.2
The manuscript suggests that all saturation theories are directly based on observations of saturation, but that is not the case for the Hill et al., 1976 model, which is a “general physical model”.

AR: Hill et al., 1976 specifically states that ``For Earth, the limiting potential of about 55 kV” [calculated from their theory] ``is in reasonable accord with observations", suggesting their theory derives validity from some observational inference that there exists a "limiting potential of about 55 kV".

Theories are taken seriously as a scientific theory by other scientists only when they make predictions that can be confirmed by observations. Hill et al., 1976 specifically states that ``For Earth, the limiting potential of about 55 kV” [calculated from their theory] ``is in reasonable accord with observations". Though Hill et al., 1976 provides no citation for the observations, clearly their theory arises from some observational inference that there exists a "limiting potential". It is true that Hill et al., 1976 attempts a physical explanation for a limiting potential, however the theory arises from existing observations of saturation at Earth as most "general physical models".

The supplementary information must be consulted before finding reference to the early work of Reiff et al., where the text states "we observe from the 1980s to the 2000s … as more and more solar wind data accumulates, the polar cap potential's saturation limit moves from 0.5 mV/m to 10 mV/m". As noted in the accompanying table Reiff et al. found an approximate saturation limit higher than subsequent work by Wygant et al. Similar saturation limits to the work by Reiff et al. were obtained by Russell et al. and Shepherd et al. in the early 2000s. The progression of the apparent saturation limit with time and increased data is not supported by the presented chronology.

RC 3.3

Authors claim that the saturation limit increases with time due to increased sample size of observational studies, but this contradicts the fact that Wygant et al., 1983 has a lower saturation limit than Reiff et al., 1981. Hence, Extended Data Table 2 [New Extended Data Figure 5a] does not support the authors' claim.

AR: We clarify that we ***do not*** mean that the saturation limit presented in observational studies monotonically increases with time, but rather it shows a statistical trend that increases with time. We add New Extended Data Fig 5b demonstrating the increasing trend of the saturation limit with time.

Revision:

13. New Extended Data Figure 5 added as a combination of previous Extended Data Table 2 as panel a, and a new figure showing the increasing trend of the saturation limits as panel b.
13.a Modified line "When examining space science literature on this problem, we observe from the 1980s to the 2000s (See Extended Data Figure 5a-b) that as more and more solar wind data accumulates, the polar cap potential's saturation limit ~~moves~~trends upwards from ~0.5 mV/m to ~10 mV/m." (Line 922-924)

We thank the reviewer for highlighting a possibility that in this statistics-heavy manuscript, a statement about data accumulation through time, can be misunderstood as a non-statistical strictly monotonically increasing effect. We understand that the statement "we observe from the 1980s to the 2000s… as more and more solar wind data accumulates, the polar cap potential's saturation limit moves from 0.5 mV/m to 10 mV/m" can be misunderstood as a monotonic increase of the saturation limit from 0.5 mV/m to 10 mV/m. Hence, we edit this sentence in Supplementary Discussion 1a Line 922-924 to be more clear: "When examining space science literature on the problem, we observe from the 1980s to the 2000s (See Extended Data Figure 5a-b) that as more and more solar wind data accumulates, the polar cap potential's saturation limit trends upwards from ~0.5 mV/m to ~10 mV/m." We also add a new figure, New Extended Data Fig. 5b, which shows graphically this increasing trend of the saturation limit from observational studies with increasing time.

a

Study	Saturation limit in E_m^* [mV/m]	Instruments
1) Reiff et al., 1981	~ 3	LEO Satellites: AE-C, AE-D, S3-3
2) Wygant et al., 1983	~ 0.5	LEO Satellites: S3-3
3) Weimer et al., 1990	~ 4	Magnetometers, AE Index
4) Boyle et al., 1997	~ 8 (found no saturation)	LEO Satellites: DMSP
5) Russell et al., 2001	~ 3	AMIE
6) Liemohn & Ridley, 2002	~ 10	AMIE
7) Shepherd et al., 2002	~ 3	SuperDARN radars
8) Nagatsuma, 2002	~ 5	Magnetometer PC Index
9) Hairston et al., 2005	~ 10	LEO Satellites: DMSP

b

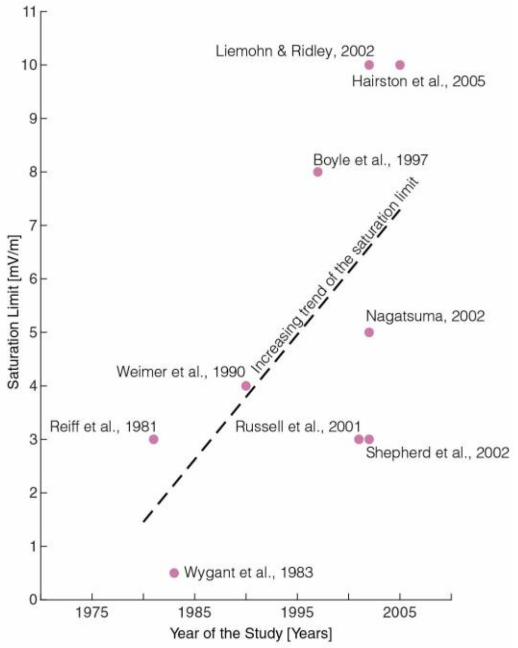

Extended Data Figure 5: a) Table showing the saturation limits concluded by different observational studies and the corresponding instruments used to measure the geomagnetic response **b)** The linear least-squares fit (i.e. the trend line) of a scatter plot of the different saturation limits of observational studies of polar cap potential saturation from 1980-2005, show an increasing saturation limit with time.

One key finding in Reiff et al. (1981) is that "[t]he degree of correlation between interplanetary parameters and the polar cap potential drop turns out to be quite sensitive to the way in which the magnetosheath field B1 is inferred from the (observed) solar wind field Bsw." While this is a rationale for their amplification-limitation procedure in deriving their empirical model, it's important to note the implications. Their "saturated" model is linear once the amplification-limitation has been applied, i.e., the relationship between the sheath driving and the response is linear, but the relationship between upstream solar wind driving and response is not. This is essentially the position taken in this manuscript, though the arguments about how it arises are different.

RC 3.4
Reiff et al., 1981's "position" (i.e., conclusion) is essentially the same as the author's even though the authors' arrive at their conclusion through different means.

AR: The referee's definition of "position" will imply that all saturation theories that propose a process that reduces the day-side reconnection rate as a cause for the saturation, have "essentially the same position". However, this is factually incorrect. Reiff et al., 1981's conclusion is entirely distinct from ours: they conclude that a physical process in the sheath diminishes the day-side reconnection rate, we conclude that regression bias caused by zero mean random error of shocked solar wind driver estimates create an appearance of saturation in data without any physical process that diminishes the day-side reconnection rate.

Any theory that subscribes to the claim that the day-side reconnection rate is diminished due to some physical process will find themselves in the same “position” as the referee puts it: “the relationship between the sheath driving and the response is linear, but the relationship between upstream solar wind driving and response is not”. In our case, we show that these physical theories **do not** correspond with the correct interpretation of data. When the regression bias from zero-mean random error of the shocked solar wind driver is corrected in the data, then the real relationship of the driver and cross-polar cap potential is linear. In other words, if Reiff et al., 1981 had the correctly inferred data, no amplification-limitation procedure would need to be used, as the magnetopause boundary would move inward to balance the magnetic pressure in the magnetosheath. Instead, Reiff et al., 1981 rely on an arbitrary and forced cut-off of solar wind with high magnetic field values and its forced reduction to a low value. Evidence for the lack of validity of Reiff et al., 1981’s underlying physical theory can easily be seen when comparing the Reiff et al., 1981 results with Boyle et al., 1997 and Hairston et al., 2005, neither of which see any saturation of polar cap potential at 3mV/m regimes, and instead see a linearity until 8mV/m and 10mV/m respectively without using Reiff's “amplification limitation” procedure, despite using low-earth orbit satellites like Reiff et al., 1981 to measure the cross-polar cap potential values. If the theory behind the amplification limitation procedure holds true, saturation should occur at similar limits, and not move to higher values with larger sample size of data. In fact, the sample size of Reiff et al., 1981 is 32 events, with 3 data points (i.e., outliers) that deviate from linearity. By contrast Boyle et al., 1997 and Hairston et al., 2005 have 100s to 1000s of data points, suggesting that Reiff et al., 1981 is likely mistaking undersampling bias for a physical effect proposed by Hill et al., 1976.

It is also not clear to me that the term “regression to the mean” is being used in a way that is consistent with standard statistical usage. The description is also not always clear or consistent. For example, the manuscript states that “regression to the mean is not a statistical phenomenon”, when the generally accepted meaning is (as used in Barnett et al., cited by the authors) that “relatively high (or relatively low) observations are likely to be followed by less extreme ones nearer the subject’s true mean.” As such, it is inherently a statistical phenomenon reflecting the relative likelihood of drawing a sample from the tail of a distribution instead of the bulk of the distribution. The example provided in text, associated with figure 2, was previously published and is not inconsistent with the standard definition and interpretation, so it's not clear to me that there’s any support for the position that this is anything other than a statistical effect. The “fundamental logical consequence” referred to on line 201 is noted in terms of likelihood on line 203 and is referring to a stochastic process. Perhaps the implication is that “a statistical phenomenon” is perhaps only an apparent phenomenon, and if something is rooted in reality then it is no longer a statistical phenomenon? Either this interpretation is different and not new (previously published by Sivadas and Sibeck) or it is not different to the historical interpretation of regression to the mean and is instead a more nuanced examination of the impact on specific problems.

RC 3.5.1

The authors' claim that "regression to the mean is not a statistical phenomenon", but there is no evidence presented by the authors' to claim that this is anything but a statistical phenomenon. The authors' claim that regression to the mean is "a fundamental logical consequence of the relationship between the true value and its measurement" is still explained only in terms of likelihood, and hence its purely a statistical phenomenon. Their description of the effect is not always clear or consistent.

AR: We do NOT claim that "regression to the mean is **not** a statistical phenomenon" in this manuscript. We claim that "regression to the mean ***itself is not*** a statistical phenomenon, though it can appear as one", by which we claim regression to the mean is **not only** a statistical phenomenon, it has a more fundamental origin. In the manuscript, we explain the regression to the mean effect in 5 different ways, each from a different vantage point. All the different vantage points are consistent with each other (and have to be, if they are a reflection of reality). The fifth vantage point is from the relationship between truth and measurements, which is entirely consistent with the other 4 vantage points, and is derivable only by combining all of them, and is not yet presented in any study we know of. We make edits in the manuscript to clarify the above points, and also present a new Supplementary Discussion 1.c and 1.d. Section 1.c summarizes the different vantage points. Section 1.d explains with examples of another fundamental property i.e., electric field, to clarify that regression to the mean is a fundamental property and not only a statistical or probabilistic effect, even though we observe it only through a statistical method or probability theory.

Revision:

5. Modified line 169-170 "The regression to the mean ~~itself~~ is not only a statistical phenomenon, though it ~~can appear~~commonly appears as one."

3.a. Adding reference to new supplementary section: "We explain why this property is a fundamental one in Supplementary Discussion 1.d." (Line 173-174)

3.b. Adding reference to new supplementary section: "In Supplementary Discussion 1.c, we explain the regression to the mean effect from five different yet consistent vantage points." (Line 202-203)

3.d.i. Added new supplementary section: Supplementary Discussion 1.c Regression to the mean from different vantage points (Line 963-996)

3.d.ii. Supplementary Discussion 1.d Regression to the mean as a fundamental property of the relation between truth and measurement (Line 997-1052)

We thank the referee for pointing out an important misunderstanding that may arise in the reader's mind: regression to the mean is not a statistical phenomenon. To avoid this misunderstanding, we modify the Line 169 as : "The regression to the mean ~~itself~~ is not **only** a statistical phenomenon, though it **commonly** ~~can~~ appear**s** as one."

We explain the regression to the mean effect from 5 vantage points, and have included this as a Supplementary Discussion 1.c:

1. Vantage point 1: Regression bias: the average value of one dependent variable given the other independent variable will be biased closer to the mean value when there is some measurement uncertainty in the independent variable.

2. Vantage point 2: Repeat measurements: extreme measurements are more likely followed by measurements closer to the mean (which is the most popular understanding of the phenomenon).

3. Vantage point 3: Likelihood of stochastic process: any uncertain measurement of a stochastic process is more likely measuring the true value closer to the most-probable value, as values closer to the most-probable value are more likely to occur.

4. Vantage point 4: Probability theory: Figure 2a geometrically shows that for a Gaussian process, for a given measurement the true value is likely closer to the mean.

5. Vantage point 5: Relationship of truth and measurement: From point 4, and consistent with other three points, we can infer that regression to the mean is a property of the relation between truth and measurement, the effects of which are observable in statistical methods, and can be examined through probability theory.

We have referenced this section in the Main Text in line 202-203: "In Supplementary Discussion 1.c, we explain the regression to the mean effect from five different yet consistent vantage points."

The referee raises a concern regarding our claim that "regression to the mean is a fundamental property of the relation between truth and measurement": "it's not clear to me that there's any support for the position that this is anything other than a statistical effect".

We address this concern by including a supplementary discussion section 1.d, which is also provided below. On Line 173, we add the following sentence referring the reader to this section: " We explain why this property is a fundamental one in Supplementary Discussion 1.d."

By fundamental property we mean a property that exists in reality independent of the methodology we use to study it, as opposed to an effect that arises from the methodology. For example, an undersampling bias is an effect that arises purely from the number of samples we use in our analysis. All methods that use infinite samples will never know of the undersampling bias. We argue that the regression to the mean effect, on the contrary, exists in reality as a property of the relation between truth and measurement independent of the methodology we use.

The first evidence for this comes from the fact that the phenomenon is observable in multiple statistical methods that use populations with inherent variability (e.g., different types of regression analysis, repeat measurements, machine learning, estimating conditional probability distributions), and also observable through probability theory when inferring the truth corresponding to the measurement. The second evidence is the mathematical result that can be derived using probability theory, graphically illustrated using Figure 2a, which shows that the effect only depends on the nature of the truth and measurement i.e., their likelihood of occurrence and their uncertainty. This implies that even when one makes a single measurement, the regression to the mean effect leads to a bias in the true value corresponding to the measurement according to the stochastic properties of the phenomenon being measured and the uncertainty in the measurement.

Here, an example of a familiar but different fundamental property of relations between things, e.g., the electric field, might be instructive and help us see the features of a fundamental property. An electric

field is a fundamental property of the relationship between two or more charges. This relation, like all relations, has a qualitive aspect and a quantitative aspect. The quality of electric field is that it displaces the two charges relative to each other. The quantitative aspect is that the displacement depends on the nature of both the charges, its sign, magnitude, and distance between them. It was demonstrated to exist through experiments conducted by Faraday, but that does not mean it is only an experimental effect. It was proven to be mathematically consistent by Maxwell, but that does not mean it is only a mathematical effect or "field-theoretic" effect. This relationship between charges exists in reality, irrespective of the tools we use to observe it.

Similarly, when we say regression to the mean or the more-probable, is a fundamental property of the relationship between truth and measurements, it also implies, this relation, like all relations, has a qualitative and quantitative aspect. The quality of this property is that the truth is separated from the measurement by some measure. The distance of separation depends on the nature of the truth and the measurement, i.e., the stochastic properties of the truth, the degree of uncertainty of the measurement, and how it changes with time, space and magnitude of the truth. It can be demonstrated to exist through experiments with a large number of measurements (i.e., statistical methods), but that does not mean it is only a statistical effect. It can be proven to be mathematically consistent using probability theory (Figure 2a), but that does not mean it is only a probabilistic effect. This relationship between truth and measurement exists in reality, irrespective of the tools we use to observe this relationship.

The existence of this relation between truth and measurement arises only because different from each other. Here we provide a philosophical insight into why that is the case. In our universe, truth is generally only accessible through measurements - this is because the universe is a complex system. Complex systems have many processes, that the most prominent process will have a systematic effect that we can observe, which we may call a "law", or we infer as the physical process of our interest. However, on the flip side, it will have other processes that appear as random perturbations, which may not be of our interest, and hence appear as randomness. This forces a separation between truth and measurements, leading them to be both different things. Truth is not measurement, and measurement is not the truth. However, there is an inseparable connection between them. Truth cannot be accessed without measurements, and measurements have no independent existence without the truth. This firmly establishes the existence of at least one, if not many, relationships between truth and measurement. One such relation is the regression to the mean of the truth, where the randomness manifests as uncertainty in measurements, and stochastic variations in the truth, leading to a separation of the truth from measurement in quantifiable ways.

RC 3.5.2
The authors' claim that regression to the mean is "a fundamental logical consequence of the relationship between the true value and its measurement", is not new and already published in Sivadas and Sibeck, 2022.

AR: In Sivadas and Sibeck, 2022 and Figure 2a, we have presented using probability theory the argument for why truth is closer to the mean of the stochastic process. However, the conclusion from this result, that the regression to the mean is a property of the relation between truth and measurement is being presented for the first time in this manuscript.

As discussed above, Sivadas and Sibeck, 2022, presents the vantage point 4: explanation of the regression to the mean using probability theory. Using this, and the three other vantage points, we infer from that the essence of the regression to the mean effect: that it is a fundamental logical consequence of the relation between truth and measurement, and that it is **not** a result of the methodology we use. This is new and is presented for the first time in this manuscript. This new vantage point is important because it makes clear that the regression to the mean effect is ubiquitous. As it is a property of something as foundational as the relationship between measurement and truth, it is likely present even when one makes a single measurement (i.e., non-statistical methods), and also across many statistical methods, including machine-learning methods, and other fields of study.

RC 3.5.3
The authors' claim that regression to the mean is "a fundamental logical consequence of the relationship between the true value and its measurement", is not different from the historical interpretation of the regression to the mean effect as a statistical effect.

AR: Our claim that the regression to the mean is "a fundamental logical consequence" is consistent with the statistical explanation that "extreme measurements are more likely followed by measurements closer to the mean"; yet it is a new explanation that reveals that the source of this effect lies in the nature of the separation between truth and measurement (i.e., their relation) in our universe. The consistency of our explanation with the previous understanding of the regression to the mean as a purely statistical effect, does not make our result the same as the previous one; in fact, it is a necessary condition - as the statistical effect is an observable material fact.

As discussed above, we present a new interpretation of the regression to the mean effect - as a property of the relation between truth and its corresponding measurement, which is different from its interpretation that regression to the mean is a statistical effect, yet entirely consistent with it. The consistency of our interpretation with the historical interpretation is not a flaw, rather an essential feature and one that strengthens its validity.

Another important point to stress is that the regression to the mean effect, though known from 1886 (Galton 1886), is widely misunderstood and entirely ignored within many fields of science. Our manuscript demonstrates how this effect contributes to a foundational problem in space physics (entirely resulting from random error), and hence brings attention of the wider scientific community to this effect. Furthermore, we believe that the deeper interpretation of regression to the mean as a property of the relation between measurement and truth, can be more easily understood by physicists and scientists as opposed to the interpretation that its a statistical effect that occurs when one uses particular statistical methods, which is usually understood correctly only by experts in statistics.

On the specific example problem shown in the manuscript: the relationship being shown is that between upstream solar wind measurements and the PCI. The authors note on line 224 that the solar wind is modified through the bow shock and magnetosheath. The so-modified solar wind is what is physically coupling to the magnetosphere, primarily through reconnection. Thus, epsilon in this model includes the effect of shocking the solar wind plasma at the bow shock and any further evolution of this now more-turbulent magnetized fluid. While crossing a shock includes effects that could be considered

random, there are also systematic effects on the plasma. The density increases and the bulk speed decreases. The compression increases the magnetic field strength (e.g., the amplification referred to by Reiff et al.). This is no longer strictly a measurement effect and is instead aiming to capture a physical process. That is, even with perfect measurements of plasma at L1 and magnetosheath, and perfect knowledge that they were connected by a streamline, assuming no evolution outside of crossing the bow shock the values of X and X* would still be different. The presented model includes this process implicitly in the epsilon term and then describes it as an uncertainty. This fundamentally impacts the interpretation of the statistical model presented as the argument is that the relationship between PCI and upstream solar wind is really linear and due to measurement uncertainty, while simultaneously rolling the effect of the bow shock on plasma properties into the "uncertainty". Without additional work to clarify this and to distinguish the effects, it appears that this work is entirely consistent with Reiff et al. (1981), but without explicitly capturing the physical processes affecting the evolution of the plasma. How much of the nonlinear relationship, seen here as a conditional bias, is due to a physical process rather than a measurement uncertainty? If this cannot be distinguished then we have a useful tool for empirical modeling, but not a physical explanation that disproves the physical hypotheses for saturation relative to the upstream measurement.

RC 3.6.1
Epsilon (the magnitude uncertainty) includes not only "measurement uncertainty" but also systematic effects on the plasma due to the bow shock.

AR: Epsilon (the magnitude uncertainty) is calculated as the variance of solar wind driver for a given shocked solar wind driver value in the magnetosheath, and hence has a zero-mean. This implies there is no component of systematic physical effects rolled into epsilon. Below we show three arguments that prove this point.

Let us assume the magnitude uncertainty ε has two components as the referee suggests: a random error component u and a systematic physical effect or bias b.

$$\varepsilon = u + b$$

If we average or take the expectation of ε, then we will get:

$$\langle\varepsilon\rangle = \langle u + b\rangle = \langle u\rangle + \langle b\rangle = 0 + b = b$$

If the magnitude uncertainty ε has any component of systematic physical effects, then the average of ε will leave behind a non-zero value equal to the bias $b \neq 0$ caused by the systematic physical effect.

As described in the manuscript (Line 639-642), the way we calculate the magnitude uncertainty ε is by calculating "the variance in the measurements made at L1 and time-shifted to the bow shock (E_m^*)" for "specific values of the magnetosheath measurements (E_m^{sh})". Or in other words, $\varepsilon \sim var(E_m^* | E_m^{sh})$, and due to the way in which variance is defined, there is no element of systematic effect or bias contained in the variance. The magnitude uncertainty ε in the error model [Line 643-644], "is set to the same variance, and we model it as a zero-mean Gaussian with a standard deviation that varies with the magnitude of X according to data." Therefore, we should expect that there is no element of systematic physical effect or bias rolled into the magnitude uncertainty or $\langle\varepsilon\rangle = 0$.

Proof 1: Average of magnitude uncertainty is zero. The proof of this is in the following figure. The top panel shows the probability density function of the magnitude uncertainty, ε, and the mean of it is 0.002 mV/m, proving that there is no element of bias.

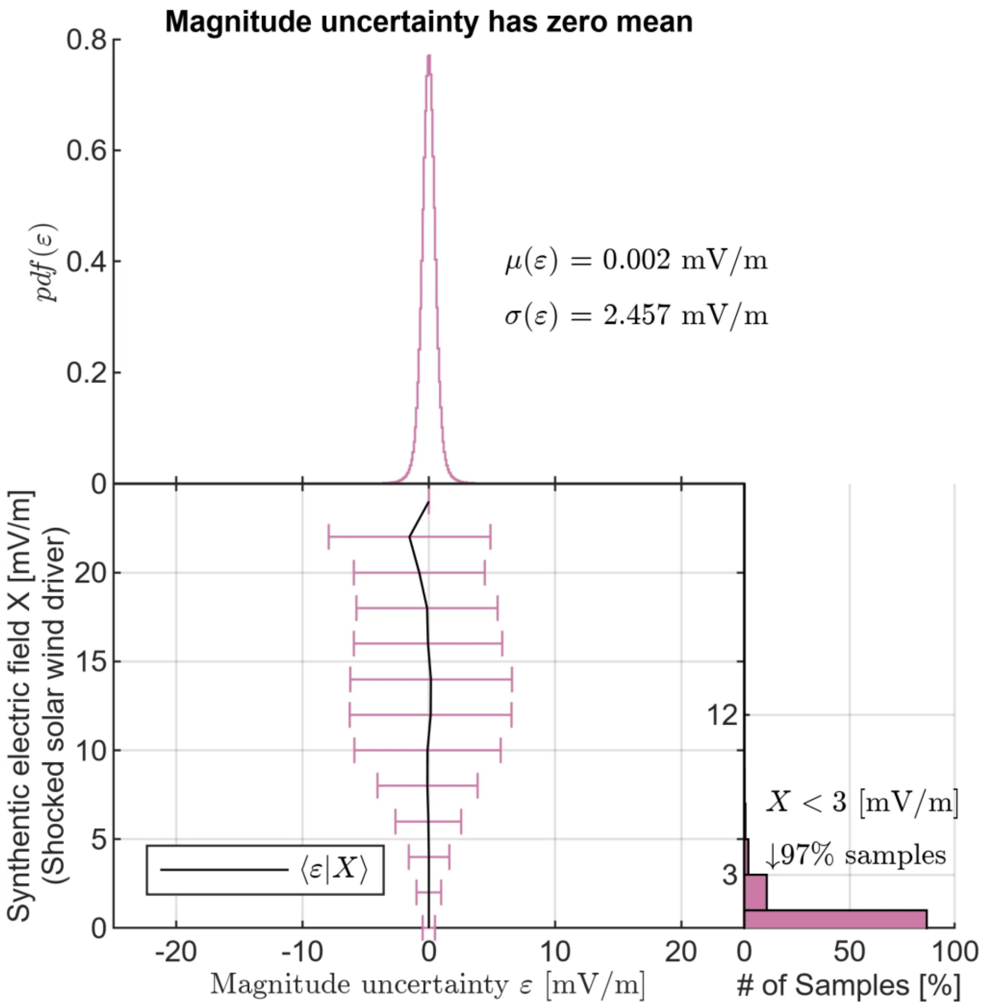

AR-Figure 1: (Top Panel) Shows the pdf of the magnitude uncertainty, which has zero-mean, implying no systematic effects - either physical or otherwise is part of the magnitude uncertainty. (Bottom left) Shows how the mean of the magnitude uncertainty remains 0 with the strength of the true value X, implying here too there is no systematic effects, physical or otherwise, rolled into the magnitude uncertainty. (Bottom right) Shows the probability distribution of shocked solar wind driver value, suggesting that ~97% of the samples have value less than 3 mV/m.

Proof 2: Average of magnitude uncertainty given shocked solar wind driver value is also zero. Even if one is persuaded by the previous argument, one might wonder if there is any systematic physical effect or bias that is present at high values of solar wind driver, but not within the more common and low values.

If bias $b(x) \neq 0$, then

$$\langle \varepsilon | X = x_i \rangle = \langle u + b(x) | X = x_i \rangle = \langle u | X = x_i \rangle + \langle b(x) | X = x_i \rangle$$
$$= 0 + b(x_i) = b(x_i)$$

However, in the above figure, the bottom panel shows the mean of the magnitude uncertainty for a given value of X (solid black line), which is 0 mV/m for nearly all values. For values of $X > 15$ mV/m, as the number samples available reduces (see right panel), the mean value goes up to -1.5 mV/m at a value of $x_i = 23$ mV/m, still within the standard deviation of the magnitude uncertainty for a given value of X (i.e., $std(\varepsilon|X)$) shown by the pink error bars. In other words, even in average of the magnitude uncertainty for a given shocked solar wind driver value, there is NO systematic effects or bias.

Proof 3: Average of erroneous solar wind driver at L1 given shocked solar wind driver value is also zero. When the above zero-mean magnitude uncertainty is the basis of the following error model $X^* = X + \varepsilon$, one might wonder if there are any systematic effects rolled into the magnitude uncertainty how would that affect the error model.

If $\varepsilon = u + b(x)$, then the average of X^* given a specific value of $X = x_i$

$$\langle X^* | X = x_i \rangle = \langle X + \varepsilon | X = x_i \rangle = x_i + b(x_i)$$

In other words, if there is any systematic physical effect, the conditional expectation of X^* given X, will be equal to the value of $X = x_i$ but with an added bias $b(x_i)$.

However, when we do this exact calculation on the error model $X^* = X + \varepsilon(X)$, we get the following result:

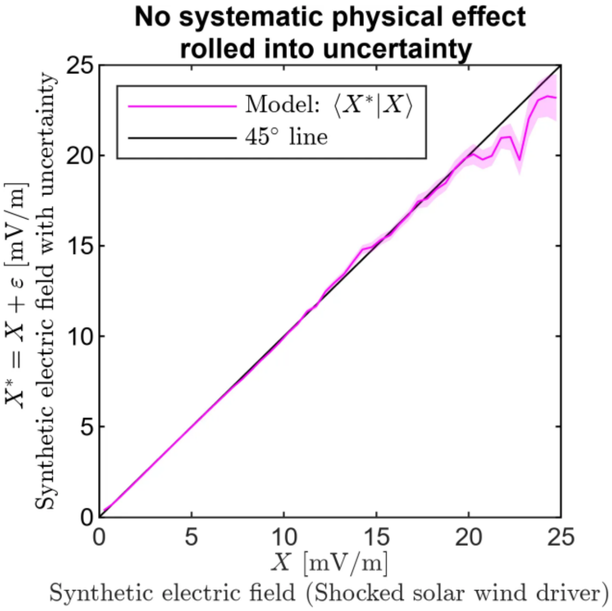

AR-Figure 2: Synthetic electric, X, against the synthetic electric field with uncertainty, X*. The relationship shows that no systematic uncertainty is rolled into the result.

For nearly all values of $X = [0, 20]$, $\langle X^* | X = x_i \rangle = x_i$, hence $b(x_i) = 0$, or in other words there is no sign of bias or systematic physical effect on the error model. For values beyond 20 mV/m there is likely minor variability related to undersampling.

The above three results confirm that there is no systematic effect rolled into the magnitude uncertainty, and that the bias in the magnetosheath driver for a given solar wind driver calculated in the manuscript is only from the regression to the mean effect arising from zero-mean random error. This is the central result of the error model we have constructed, which ought to be evident from its construction alone, and it is also the reason why the result is surprising and novel.

RC 3.6.2
Without distinguishing the systematic physical effect causing the saturation from random error, the manuscript does not disprove the physical saturation theories.

AR: The manuscript does not disprove saturation theories, instead it calls them into question as the data underlying them shows no saturation - hence the theories have no material basis given the correct data. As shown above, there is not an element of systematic physical effects in our error model, the result presented in the manuscript is purely from the regression to the mean effect. This makes the findings of our study unique and novel (See RC 3.0.5.2 and RC 3.0.6).

The referee is correct that our theory does not disprove the physical saturation theories. However, it does lead to a conclusion that is even more significant: there is no evidence in data for the saturation the physical theories are constructed to explain!

As shown above, our work distinguishes the systematic physical effect from the random error, as our error model purely calculates the effect of the random error. Once the effect (bias) stemming from random error is corrected, whatever geomagnetic response to the solar wind driver is revealed by the corrected data is what a physical theory must explain. We have stated this clearly in our conclusion section 7, line 364-369: “Our result is not an alternative [physical] theory competing with existing [physical] theories of saturation, but rather a demonstration that available data does not show evidence for the saturation of geomagnetic response. Hence, it challenges the foundation of existing physical theories of saturation, and they may have to be revisited and validated with the calibrated solar wind driver estimates.”

RC 3.6.3
The manuscript is entirely consistent with Reiff et al., 1981, by implicitly capturing systematic physical effects within the magnitude uncertainty epsilon, hence not novel.

AR: As shown in RC 3.4 and RC 3.6.1-3.6.2, our manuscript is entirely different from Reiff et al., 1981, especially since the saturation effect described by us is purely from zero-mean random error and not any systematic effects (originating from physical processes or measurement bias). While Reiff et al., 1981 uses an arbitrary and forced cut-off of solar wind with high magnetic field values (i.e., they introduce an artificial systematic bias) and its forced reduction to a low value, to explain the saturation effect.

On a related note, the sensitivity of the results to the assumptions in the model are not given. While some justification is given for selected values, such as dt1 and dt2, it is unclear whether the results are sensitive to these values.

RC 3.7

Sensitivity of results to the assumptions in the model are not given.

AR: The assumptions we use in the error model are derived directly from measurements, and the model result are already validated using second-order statistics, making a sensitivity analysis superfluous. However, we have already provided a sensitivity analysis of time uncertainty in Figure 3c, and also in Equation 1.21 (Line 1209-1210). However responding to the reviewers interest for a sensitivity analysis of magnitude uncertainty, we have now added New Extended Data Figure 4. See also RC 3.0.3.

Revision:

9. Adding references to figures that provide sensitivity analysis: "Additionally, we show a sensitivity analysis of the non-linear regression bias, which increases with increasing uncertainty in the time of propagation (See Figure 3c), and with increasing uncertainty in the magnitude uncertainty (See Extended Data Figure 4)." (Line 669-671)

12. New Extended Data Figure 4 added as sensitivity analysis of magnitude uncertainty. "Extended Data Figure 4| **Sensitivity analysis of magnitude uncertainty.**"

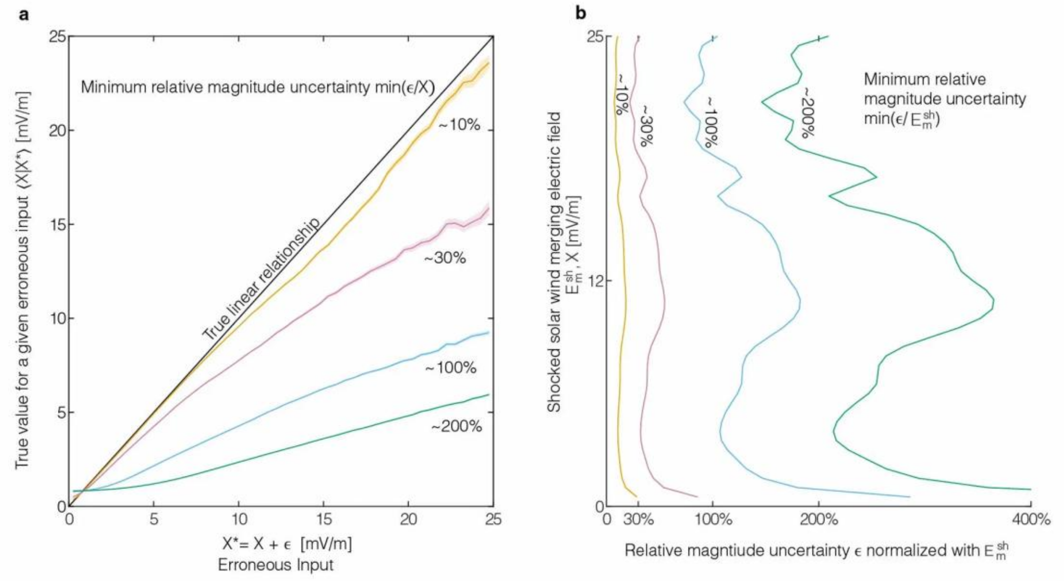

Extended Data Figure 4: Sensitivity analysis of regression bias with magnitude uncertainty. a, Non-linear regression bias caused by heteroskedastic magnitude uncertainty increases with a measure of the uncertainty represented by the minimum relative magnitude uncertainty. **b,** The heteroskedastic variation of the magnitude uncertainty with the true value is shown, for different values of the minimum relative magnitude uncertainty corresponding to the different regression bias curves in panel a.

In this manuscript, inputs of time uncertainty, and magnitude uncertainty are directly calculated from measurements, except that for time uncertainty values we have used previously well-established estimates from studies that have directly calculated them from measurements. These are not only assumptions, they are model inputs that have been calculated carefully from data. We have provided the sensitivity of these input parameters to the output of concern: i.e., regression bias caused by them, in Figure 3c and the newly added New Extended Data Figure 4. We have referenced this sensitivity analysis in the methods section Line 669-671 using the following line "Additionally, we show a sensitivity analysis of the non-linear regression bias, which increases with increasing uncertainty in the time of propagation (See Figure 3c), and with increasing uncertainty in the magnitude uncertainty (See

Extended Data Figure 4).”

For example, extended data figure 4 [New Extended Data Figure 2a] shows a comparison of the modeled PDF to the empirical PDF and the caption states that it “predicts the pdf … almost exactly”. There are clear deviations and differences in shape, and while the correspondence is good, it is far from exact and is presented with no uncertainties or testing for similarity to the observed distribution. How much of the correspondence can be tuned by selection of different values for dt1, dt2, or epsilon? The same requirement would apply to extended data figure1 [New Extended Data Figure 1a], though that is not claimed to be an “almost exact” match.

RC 3.8.1
Probability density functions shown in Extended Data Figure 4 [New Extended Data Figure 2a] of data and model, have a correspondence that is far from exact.

AR: We agree with the referee that the phrase “almost exactly” may be misleading, and hence we use the phrase the referee has used: “good correspondence” in the manuscript. We quantify the uncertainty by stating that the difference in probability density between model output and data is ~<0.1 for any given X*, and also note that the figure is in log-scale and hence the difference in shape may appear larger for values with lower probability density.

Revision:
16. Modified figure caption of Extended Data Figure 2a, “The model predicts with good correspondence the pdf of the estimate of the merging electric field propagated to the polar cap, ~~almost exactly~~with a probability density difference of less than ~0.1 between data and model for most values.” (Line 1331-1334)

The AR-Figure 3 below shows the Extended Data Figure 4 [New Extended Data Figure 2a] in a linear scale (right panel), instead of the log-scale shown in the manuscript (left panel). This makes it clear that they have a good correspondence with the probability density being less than 0.1 for nearly all values of X*. We use the log-scale to highlight the differences in the tail of the distribution, which is the focus of this work, as that cannot be viewed in the linear-scale.
We have made the following change in the captions of the New Extended Data Figure 2a: "The model predicts with good correspondence the pdf of the estimate of the merging electric field propagated to the polar cap, with a probability density difference of less than ~0.1 between data and model for most values. E_m^* is data, and X^* is model.”

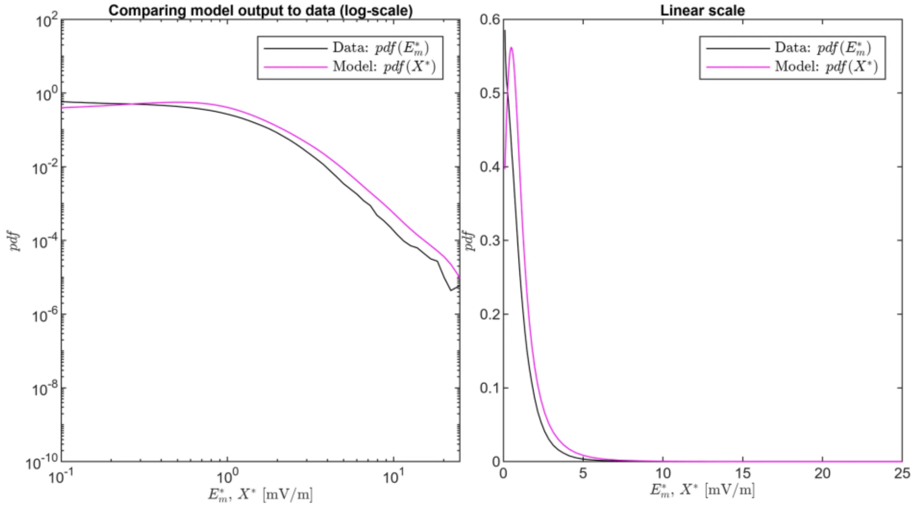

AR-Figure 3: a) Probability density function of model output compared with its counter part in data in log-scale, b) Probability density function of model output compared with its counter part in data in linear scale.

RC 3.8.2

How much of this correspondence can be tuned by selection of different values for dt1, dt2, or epsilon.

AR: In this paper, we do not tune any input parameters, they are calculated directly from data and placed into the error model. The argument is that any lack of correspondence of the model output with that observed in the data, is a result of effects other than the random error and the regression to the mean effect or because our uncertainty estimates made from data need improvement. Hence assuming our estimates of random error are correct, the lack of correspondence will be a result of either instrument bias or physical processes. Sensitivity analysis of the regression bias for given time uncertainty (dt1+dt2) and magnitude uncertainty (epsilon) is presented in Figure 3c and the newly added Extended Data Figure 10 [New Extended Data Figure 4].

Revision:

9. Adding references to figures that provide sensitivity analysis: "Additionally, we show a sensitivity analysis of the non-linear regression bias, which increases with increasing uncertainty in the time of propagation (See Figure 3c), and with increasing uncertainty in the magnitude uncertainty (See Extended Data Figure 4)." (Line 669-671)

12. New Extended Data Figure 4 added as sensitivity analysis of magnitude uncertainty. "Extended Data Figure 4| **Sensitivity analysis of magnitude uncertainty.**"

A detailed sensitivity analysis is not essential because our inputs are directly calculated from measurements, however we have already presented a sensitivity analysis of the regression bias for different time uncertainty and added one now for the magnitude uncertainty in the manuscript.

Additionally, we note that our input probability distribution (Extended Data Figure 1/ New Extended Data Figure 1a) is a best-fit log-normal distribution on a proxy for shocked solar wind driver, and hence they both are not the same distribution. We use a log-normal distribution instead of an empirical distribution that exactly mimics data because our non-linear error model does not just use random variables, instead it uses stochastic processes with adjacent random variables correlated through an autocorrelation function in time. Using a known distribution such as a log-normal distribution allows us to produce such a stochastic process synthetically, this is far more complex with an empirical distribution.

Below, in AR-Figure 4, we show that changes in the input pdf of X leads to a corresponding change in the output pdf of X* that our error model solves for, for a given uncertainty. When the spread of the input distribution increases or decreases, you see a corresponding and expected change in the output pdf of X*. Such detailed sensitivity analysis will be presented in a future publication.

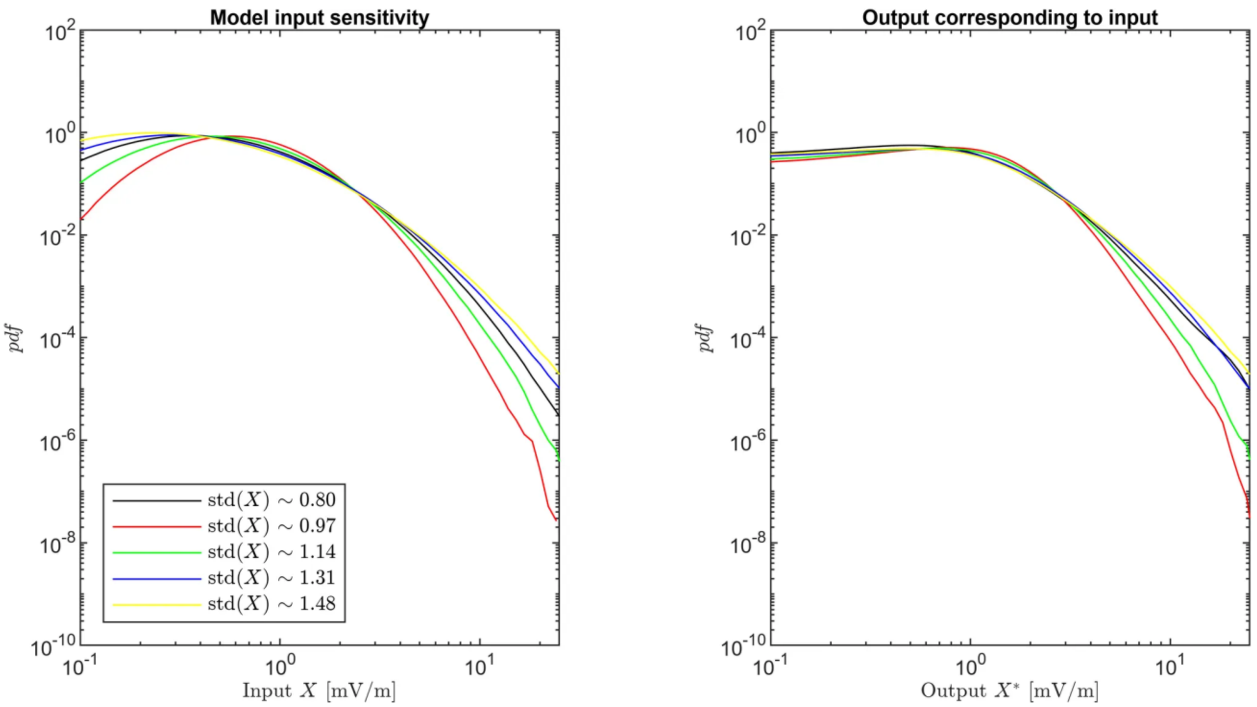

AR-Figure 4: Sensitivity analysis of error model with variation in the pdf of input X a) Probability density functions of different input X each with an increasing standard deviation parameter, b) Probability density function of output X* with corresponding input, which also increases in its spread with increasing standard deviation of the input X.

I note also that Nature requires that all uncertainties should be defined in the figures. While presumed confidence intervals are shown in multiple figures (e.g., figure 4), they are not described in the figures or figure captions. The text is further insufficient to properly describe the confidence intervals.

RC 3.9

Confidence intervals not described in the figures or figure captions.

AR: We include in the figure captions that the shaded area shows a 95% confidence interval. In the methodology section we add statements pointing out that confidence interval is only a measure of the probability that 95% of the intervals will contain the result of a process, in our case - the biased regression curve. But it does not tell us anything about the probability of whether the true value (or the unbiased regression curve) will lie within the confidence interval.

Revision:

2. Under figure captions, clarifying the meaning of the confidence interval, by adding the following sentences.

   2.a Figure 1: The transparent shaded area for both curves shows a 95% confidence interval. (Line 89-90)

   2.b Figure 2: The transparent shaded area for regression curves shows a 95% confidence interval. (Line 182)

   2.c Figure 3: The transparent shaded area for both regression curves shows a 95% confidence interval; (Line 234-235)

   2.d Figure 4: The transparent shaded area for both regression curves shows a 95% confidence interval; (Line 337)

   2.e Adding an explanation for the confidence interval in methods section "We note here that the confidence intervals shown in Figures 1-4 only provide an estimate of the reliability of the regression procedure, i.e., it means 95% probability that repeating the procedure will contain the result and does not mean the true value or the true regression curve will lie within the confidence interval with a 95% probability." (Line 693-696)

The software used (per inspection of the provided code) is not cited or acknowledged in the manuscript.

RC 3.10

Software used is not cited or acknowledged in the manuscript.

AR: In line 728-733, we have cited the software used. The provided code-capsule is private and not under the author's control during initial submission of the manuscript.

Revision:

10. Adding references to the code "[49]" (Line 726) and data "[50]" (Line 730) archives pointing to the zenodo repositories as follows [RC 3.10]:

    a. 49. Sivadas, N. *et al.* Simultaneous measurements of solar wind from L1, shocked solar wind from Magnetosheath, and measures of geomagnetic response from ground-based indices. Preprint at https://doi.org/10.5281/zenodo.17546718 (2026).

    b. 50. Sivadas, N. Code for Regression to the Mean of Extreme Geomagnetic Storms. Preprint at https://doi.org/10.5281/zenodo.17559999 (2026).

Referee #3 (Remarks on code availability):

I have read the code, but have not attempted to run any of it. Some comments based on that reading follow.

Code comments include a request to not share without permission, which is at odds with the provided license.

RC 3.11
A comment to not share without permission is there in the code.

AR: We thank the referee for catching this comment, as it was placed before submission for peer-review. We have removed this now.

The data files for the Python code appear to be provided as "pickles", which may not be platform-independent. This file type also can have implicit version requirements for dependencies that are not clearly spelled out in a readme.

RC 3.12
Pickle data files can have dependencies.

AR: We use the code-capsule environment that Springer Nature has given us access, to create a reproducible run environment where all dependencies are checked and frozen. It can be run by anyone in the future without worrying about broken dependencies.

A Dockerfile is provided so that this should be reproducible, but rebuilding images from a Dockerfile can result in different dependency stacks.

RC 3.13
Running Docker files can result in dependency stacks.

AR: If this manuscript is accepted, the code-capsule environment should theoretically be able to run this work exactly as the reviewer or authors have run it. Furthermore, we have an extensively commented code, that anyone can retrace, and reconstruct.

List of changes from Initial Submission to Revision v1

(Line numbers mentioned in this document correspond to **Appendix 1: Revision 1 with Markup**)

1. Added titles to figure captions.
    a. Figure 1: Saturation of geomagnetic activity with solar wind driving. (Line 83)
    b. Figure 2: Explaining regression to the mean effect using probability theory and regression bias. (Line 176)
    c. Figure 3: Regression bias predicted by the error model matches saturation effect from data. (Line 232)
    d. Figure 4: Correcting the effect of random errors in solar wind driver values reveals a linear geomagnetic response. (Line 333)
2. Under figure captions, clarifying the meaning of the confidence interval, by adding the following sentences. [RC 3.9]
    a. Figure 1: The transparent shaded area for both curves shows a 95% confidence interval. (Line 89-90)
    b. Figure 2: The transparent shaded area for regression curves shows a 95% confidence interval. (Line 182)
    c. Figure 3: The transparent shaded area for both regression curves shows a 95% confidence interval; (Line 234-235)
    d. Figure 4: The transparent shaded area for both regression curves shows a 95% confidence interval; (Line 337)
    e. Adding an explanation for the confidence interval in methods section "We note here that the confidence intervals shown in Figures 1-4 only provide an estimate of the reliability of the regression procedure, i.e., it means 95% probability that repeating the procedure will contain the result and does not mean the true value or the true regression curve will lie within the confidence interval with a 95% probability." (Line 693-696)
3. Adding reference to new supplementary sections
    a. "We explain why this property is a fundamental one in Supplementary Discussion 1.d." (Line 173-174) [RC 3.5.1]
    b. "In Supplementary Discussion1.c, we explain the regression to the mean effect from five different yet consistent vantage points." (Line 202-203) [RC 3.5.1]
    c. "Supplementary Methods 2.c summarizes the full computer code used." (Line 668) [RC 1.1]
    d. Added 3 new supplementary sections:
        i. Supplementary Discussion 1.c Regression to the mean from different vantage points (Line 963-996) [RC 3.5.1]
        ii. Supplementary Discussion 1.d Regression to the mean as a fundamental property of the relation between truth and measurement (Line 997-1052) [RC 3.5.1]
        iii. Supplementary Methods 2.c Summary of data analysis procedure in the computer code (Line 1221-1306) [RC 1.1]
4. Modified the phrase "the more recent" to "later". (Line 92) [RC 3.1]
5. Modified line 169-170 "The regression to the mean ~~itself~~ is not only a statistical phenomenon, though it ~~can appear~~commonly appears as one." [RC 3.5.1]
6. Added copyright information under Figure 2: ". ~~Figure reproduced~~This figure is adapted from Sivadas & Sibeck 2022[22], used under CC BY 4.0." (Line 181-182) [RC 1.5]

7. Added reference to new Extended Data Table 2: Symbols used in the manuscript. "The most common symbols used are explained in the Extended Data Table 2." (Line 465-466) [RC 1.4]
8. Added phrase to indicate that validation of the error model also provides the rationale for using it: "This validation ~~are~~is the rationale for using the error model and is presented in the Supplementary Methods Section 2.a." (Line 666-668) [RC 1.2]
9. Adding references to figures that provide sensitivity analysis: "Additionally, we show a sensitivity analysis of the non-linear regression bias, which increases with increasing uncertainty in the time of propagation (See Figure 3c), and with increasing uncertainty in the magnitude uncertainty ε (See Extended Data Figure 4)." (Line 669-671) [RC 3.0.3][RC 3.7]
10. Adding references to the code "[49]" (Line 726) and data "[50]" (Line 730) archives pointing to the zenodo repositories as follows [RC 3.10]:
    a. 49. Sivadas, N. *et al.* Simultaneous measurements of solar wind from L1, shocked solar wind from Magnetosheath, and measures of geomagnetic response from ground-based indices . Preprint at https://doi.org/10.5281/zenodo.17546718 (2026).
    b. 50. Sivadas, N. Code for Regression to the Mean of Extreme Geomagnetic Storms. Preprint at https://doi.org/10.5281/zenodo.17559999 (2026).
11. Consolidated Extended Data Figures into multi-panel ones:
    a. "Extended Data Figure 1 | **Stochastic properties of inputs to the error model**" contains three panels of the previous Extended Data Figures 1,2, and 3.
    b. "Extended Data Figure 2 | **Statistics of the output of the error model**" contains 5 panels of the previous Extended Data Figures 4, 5, 6, and 7.
    c. "Extended Data Figure 3 | **Another graphical explanation of the regression to the mean effect**" is the previous Extended Data Figure 8.
12. New Extended Data Figure 4 added as sensitivity analysis of magnitude uncertainty. "Extended Data Figure 4| **Sensitivity analysis of magnitude uncertainty.**" [RC 3.0.3][RC 3.7]
13. New Extended Data Figure 5 added as a combination of previous Extended Data Table 2 as panel a, and a new figure showing the increasing trend of the saturation limits as panel b. [RC 3.3]
    a. Modified line "When examining space science literature on this problem, we observe from the 1980s to the 2000s (See Extended Data Figure 5a-b) that as more and more solar wind data accumulates, the polar cap potential's saturation limit ~~moves~~trends upwards from ~0.5 mV/m to ~10 mV/m." (Line 922-924) [RC 3.3]
14. Extended Data Table 1| Theories of polar cap potential saturation, is the same table as before with a more detailed caption with references. It also provides the copyright information for the table adapted from Borovsky et al., 2008.
    a. "Ten models explaining cross polar cap potential saturation. See the following works for more details about each of the models: 1[65,66], 2 [67,68] , 3 [69,70], 4 [71, 5 70,72], 6 [73], 7 [74], 8 [75], 9 [76],10 [77,78] and an assessment of most of them[15]."
    b. "Adapted with permission from [Joseph E. Borovsky, Benoit Lavraud, Maria M. Kuznetsova, Table A1, Polar cap potential saturation, dayside reconnection, and changes to the magnetosphere., Journal of Geophysical Research: Space Science, Publisher: John Wiley and Sons] Copyright 2009 by American Geophysical Union."(Line 1410-1418)
15. New Extended Data Table 2| **Common symbols used in this work.** It contains a list of all the commonly used symbols in the manuscript and their description. (Line 1421-1428) [RC 1.4]
16. Modified figure caption of Extended Data Figure 2a, "The model predicts with good correspondence the pdf of the estimate of the merging electric field propagated to the polar cap,

~~almost exactly~~with a probability density difference of less than ~0.1 between data and model for most values." (Line 1331-1334) [RC 3.8.1]

List of editorial changes from Revision v1 to Editorial v2

(Line numbers mentioned in this document correspond to **Appendix 2: Editorial 1 with Markup**)

1. Moving paragraphs and sentences from the Main Text to additional Supplementary Discussion for the purpose of making the Main Text concise and reducing the total number of words. The words were reduced from **~4569 to ~2935.**
    a. As part of the moving text to supplementary sections the following supplementary discussions sections were created: 1a. Physics of solar-wind magnetosphere coupling, 1b. An example of the problem of definition , 1c. Random error in the independent variable does not average away, 1f. Regression bias due to magnitude and time uncertainty, 1g. Interpretation of the Error Model output, 1h. A different measure of geomagnetic response is also linear.
    b. Please note that we have not added new information in these supplementary sections, we have only moved sentences.

2. Moved paragraph from methods section to make it concise. An explanation of Kan-Lee Electric field has been moved from Line 637-650 to New “Supplementary Methods 2.a Kan-Lee electric field is an approximation of the true solar wind driver” (Line 1470-1484)

3. As per nature format all the Figures, Figure Legends and Extended Data legends have been moved after the Main Text in a new section “Figure Legends”. (Line 497 to 585)

4. A few edits and removal to the sentences have been made throughout the Main Text to further reduce the words as much as possible, while maintaining clarity.

References

1. Sivadas, N. & Sibeck, D. G. Regression Bias in Using Solar Wind Measurements. *Frontiers in Astronomy and Space Sciences* **0**, 159 (2022).
2. Borovsky, J. E. Noise, Regression Dilution Bias, and Solar-Wind/Magnetosphere Coupling Studies. *Frontiers in Astronomy and Space Sciences* **0**, 45 (2022).
3. Sivadas, N. & Sibeck, D. G. Regression Bias in Using Solar Wind Measurements. *Frontiers in Astronomy and Space Sciences* **0**, 159 (2022).
4. Borovsky, J. E. On the Saturation (or Not) of Geomagnetic Indices. *Frontiers in Astronomy and Space Sciences* **8**, 175 (2021).
5. Vörös, Z. *et al.* Probability density functions for the variable solar wind near the solar cycle minimum. *J. Geophys. Res. Space Phys.* **120**, 6152-6166 (2015).
6. Borovsky, J. E. Noise and Solar-Wind/Magnetosphere Coupling Studies: Data. *Frontiers in Astronomy and Space Sciences* **0**, (2022).
7. Vasyliunas, V. M. Concepts of Magnetospheric Convection. in *The Magnetospheres of the Earth and Jupiter* (ed. Formisano, V.) 179-188 (Springer Netherlands, Dordrecht, 1975).
8. Reiff, P. H., Spiro, R. W. & Hill, T. W. Dependence of polar cap potential drop on interplanetary parameters. *J. Geophys. Res.* **86**, 7639–7648 (1981).
9. Hill, T. W., Dessler, A. J. & Wolf, R. A. Mercury and Mars: The role of ionospheric conductivity in the acceleration of magnetospheric particles. *Geophys. Res. Lett.* **3**, 429–432 (1976).

Title: Regression to the Mean of Extreme Geomagnetic Storms

Authors: Nithin Sivadas[1,2*], David Sibeck[2], Varsha Subramanyan[3], Maria-Theresia Walach[4], Dogacan Su Ozturk[5], Banafsheh Ferdousi[6], Bayane Michotte de Welle[2]

Affiliations:

[1]Department of Physics, The Catholic University of America, Washington, D.C., USA

[2] NASA Goddard Space Flight Center, Greenbelt, Maryland, USA

[3]Department of Physics, University of Illinois Urbana-Champaign, USA

[4]Department of Physics, Lancaster University, United Kingdom

[5]Department of Physics, Geophysical Institute, University of Alaska, Fairbanks, AK, USA

[6]Air Force Research Laboratory, Albuquerque, NM, USA

*Corresponding author. Email: nithinsivadas90@gmail.com

Abstract: Extreme space weather events on Earth occur during intervals of strong solar wind driving. The solar wind drives plasma convection and currents in the near-Earth space environment. For low values of the driver, the Earth's response is linear, estimated by parameters such as the polar cap index based on ground magnetometer activity. Curiously, for extreme solar wind driving, the Earth's response appears not to increase beyond a saturation limit. Theorists have advanced a host of explanations for this saturation effect, but there is no consensus. Here, we demonstrate that the saturation is a manifestation of the *regression to the mean* effect arising from random uncertainty in the timing and magnitude of solar wind measurements. Our results reveal that data analysis underpinning the saturation theories is non-linearly biased, thereby challenging the validity of the theories. Correcting for the uncertainties reveals that the Earth's response to solar wind driving is linear throughout, and that the impact of extreme geomagnetic storms can be twice as large as previously thought. We show that regression to the mean is a fundamental property of the relationship between measurement and the truth, where the truth corresponding to the measurement is closer to the mean. This effect is particularly pronounced for uncertain measurements of extreme values and is likely to manifest across various fields, from extreme climate studies to chronic medical pain.

One-Sentence Summary: Extreme geomagnetic storms may have twice the impact on Earth than previously thought.

MAIN TEXT

1. Saturation of Geomagnetic Activity

From assessing the stability of buildings during major earthquakes to estimating the effects of hazardous radiation on biological organisms, a fundamental challenge in science is understanding how systems behave under rare and severe conditions. However, a critical, often-overlooked factor complicates this pursuit: uncertainty in measuring the conditions. In this paper, we demonstrate quantitatively that random error can systematically distort our inferences of the system's response, especially for extreme events. We leverage this crucial insight to resolve a long-standing puzzle in space physics—the apparent saturation of geomagnetic activity during extreme space weather[1].

Space physicists have long studied the effects of solar wind on the dynamics of the plasma and electromagnetic fields around the Earth. In particular, extreme geomagnetic storms with strong solar wind driving can increase the electric current strengths in the near-Earth space environment[2,3], causing nationwide power outages, disruptions in global satellite communication networks, and increased ozone loss in the polar ionosphere [4,5]. The Earth's magnetic field carves out a cavity in the oncoming solar wind, called the magnetosphere. It mostly keeps out the plasma and magnetic field associated with the solar wind except under specific local magnetic field conditions that lead to the reconnection of solar wind and magnetospheric magnetic field lines. Upstream from the reconnection-site, the supersonic and superalfvénic solar wind slows down through a bowshock, forming the magnetosheath just upstream from the magnetosphere's outer boundary—the magnetopause. When the shocked solar wind magnetic fields lie anti-parallel to those of the Earth, the field can undergo a topological change that opens up the Earth's magnetic field lines and reconnects them to the shocked solar wind magnetic field lines[6]. Through reconnection, the solar wind plasma and electric field can enter the Earth's magnetosphere and drive plasma convection in the magnetosphere. Conveyed to the polar ionosphere closer to the Earth, this convection electric field applies an electric potential across the polar cap on average[7] (See Figure 1a). The projection of the solar wind convection electric field along the dawn-dusk direction in the polar cap is the Kan-Lee or merging electric field (E_m^*)[8], which we refer to as solar wind driving[9].

In the 1970s, estimates of the cross-polar cap potential (and its corresponding dawn-dusk electric field) were made from single ground magnetometers situated in the northern and southern polar regions. The argument was that the perturbations of the magnetic field measured by the polar magnetometers give an excellent continuous estimate of the solar wind driving, as the polar regions magnetically connect to the solar wind[10]. Continuous estimates of solar wind driving from satellite missions were not yet available at the time[11]. As the magnetized plasma in the solar wind and magnetosphere is collisionless, the resistance along magnetic field lines is minuscule. Hence, the magnetohydrodynamic theory is valid, ensuring that plasma along an entire field-line moves as one[6]. Therefore, the solar wind electric field, resulting from the motion of the wind, at one end of the magnetic field line, would map along the field line to the polar ionosphere. This electric field then drives currents in the ionosphere that cause magnetic perturbations measured from ground magnetometers. Supporting this theory, the cross polar cap index (PCI), a measure of geomagnetic activity, increases linearly on average with the merging electric field (E_m^*), a measure of the solar wind driving[12]. The PCI is estimated by a polar magnetometer from the ground in each hemisphere and is proportional to the cross polar cap potential[13,14], while E_m^* is measured from a spacecraft in the solar wind far upstream of the Earth. As more measurements of rare and extreme solar wind

driving accumulated through the years, surprisingly, the correlation between the geomagnetic response and the solar wind driver began to deviate from the linear relation. There appeared to be an upper limit to the cross-polar cap potential beyond which increasing solar wind driving led to no increase in the potential (See Figure 1b green curve).

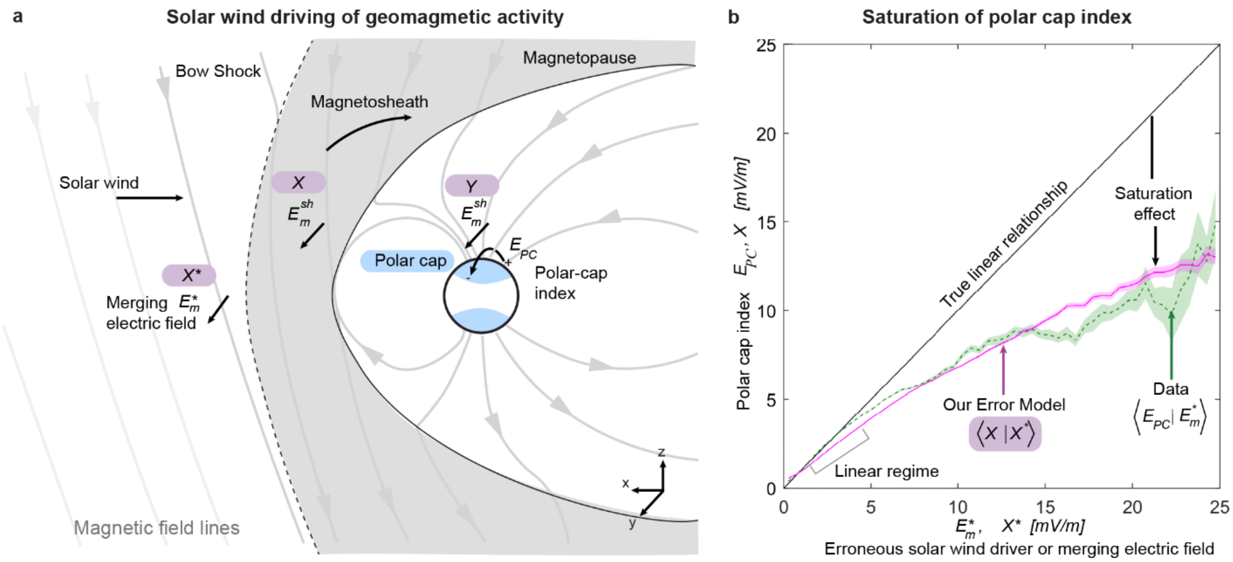

*Figure 1 <u>**Saturation of geomagnetic activity with solar wind driving.**</u> a) Solar wind merging electric field (E_m^*) measured upstream of the bow-shock, is transformed to the shocked solar wind driver in the magnetosheath (E_m^{sh}) downstream of the bowshock, which corresponds to the dawn-dusk electric field that maps to the polar ionosphere (E_{PC}); b) Observations from 1995-2019, green curve, shows that on average the cross polar cap index (∝ the dawn-dusk electric field E_{PC}) increases linearly at low values of solar wind driving, but deviates from linearity and saturates at large values of driving. The magenta curve shows that the error model developed in this work predicts the same saturation effect due to uncertainty in the solar wind driver instead of any physical mechanism. <u>The transparent shaded area for both curves shows a 95% confidence interval.</u>*

The new inference that the cross-polar cap potential saturates with increasing solar wind driving from ~~the more recent~~<u>later</u> measurements led to the emergence of 10 different but sometimes overlapping theories and models attempting to explain the phenomenon (See Extended Data Table 1)[15,16]. The theories fall into two categories, one arguing that the conditions at the solar wind/magnetosphere interaction region (i.e., the dayside magnetosheath) during extreme solar wind driving lead to a consistent diminishing of the rate of reconnection or energy coupling from the solar wind to the magnetosphere. The other set of theories argues that processes within the magnetosphere slow down ionospheric plasma convection during extreme solar wind driving. All theories suggest that the energy transferred into the polar ionosphere during extreme space weather has, in essence, an upper limit. Hence, the theories imply that the Earth's magnetosphere shields us from extreme geomagnetic storms by inhibiting energy transfer from the strong solar wind to the ionosphere, one way or the other.

In this paper, we present a radically different proposition. We argue that there is no statistical evidence for the saturation of geomagnetic response to strong solar wind driving, and its appearance in measurements is a result of uncertainty in solar wind driver measurements. We correct for the effect of this uncertainty and demonstrate that the geomagnetic response varies linearly with solar wind driving, implying that the energy transfer from extreme solar wind conditions to the polar ionosphere can be far greater than currently believed. The importance of

this work goes beyond space science to any correlation study between a measure of a system's input and its response, revealing a larger response for extreme input values.

2. Uncertainty and the Problem of Definition

The solar wind measurements are made mainly far upstream from the day-side reconnection site. They are, hence, an uncertain estimate of the actual solar wind driver close to the reconnection site in the magnetosheath, transformed during its journey, and influenced by local plasma and field conditions. Uncertainties in measurements are commonly attributed to instrumental error, but a surprising fact is that uncertainties can also result from the assumptions that we make while inferring data from measurements. A carpenter might measure the height of a door frame using a measuring tape. However, the door she creates using this height will likely not fit the frame. Even though the least count of the tape is small and does not contribute much to the uncertainty, there is uncertainty due to an implicit assumption that the height of the door is a one-dimensional quantity when it is in reality at least a two-dimensional quantity, i.e., there are multiple different heights for the door and the door frame, varying across its breadth and width. Hence, the experienced carpenter would use the carpenter's square to ensure that the edges of the door are perpendicular to the length, to match the door frame, and reduce this uncertainty. This uncertainty stems from a "problem of definition"[17].

A similar problem of definition arises when using solar wind measurements made at the Lagrange point L1, ~230 R_E upstream of the reconnection site, as an estimate for the local magnetosheath conditions that drive the coupling of energy and plasma into the magnetosphere. The shocked solar wind driving (E_m^{sh}) near the reconnection site differs from what is measured at L1[18,19] due to 1) variability in propagation times from the L1 point to the Earth, 2) spatial variability of the solar wind structure[20], and 3) evolution of solar wind along that path due to a variety of plasma processes changing the essential parameters influencing the reconnection rate, such as the local magnetic field direction.

Uncertainties arising from instrument error or the problem of definition can be of two types: bias or unbiased random error. A bias is a consistent deviation of the true value from the measurement itself. Physical theories that explain relations between physical parameters or their respective measurements almost always attempt to explain consistent and deterministic deviations between them. In fact, they are disinterested in the random fluctuations in their values. On the other hand, the unbiased random error frequently consists of these random fluctuations and is the random deviation of the measurements from the true value. Physical theories in space science generally explain average changes and not random fluctuations. Hence, an unknown bias can be misinterpreted as a physical effect (i.e., an average change) when a theory is constructed to explain the bias. In short, physical theories are in the business of explaining *physical* biases, and if we ignore bias stemming from random measurement error, we may mistake it for a physical phenomenon.

3. Regression to the mean and the more-probable

A common goal of scientific analysis is to uncover the relations between two or more physical processes. Random errors in the measurement of these processes pose a difficulty in inferring relations between them, for example, in our case, the relation between solar wind driver and the geomagnetic response. However, it is widely believed that averaging the measurements can

remove the effects of random error, as the underestimates will cancel out the overestimates. Here we will show that this widespread belief is only partially true.

A popular technique to find the relations between two or more physical processes is regression analysis of their measurements. The essence of regression analysis is to find the average of the measurement Y^* when the measurement X^* is a specific value 'x'. This is equivalent to the conditional expectation of Y^* given $X^* = x$, mathematically represented as $\langle Y^*|X^* = x\rangle$. Here, Y^* is the dependent variable and X^* is the independent or conditional variable. The averaging of Y^* indeed removes the effect of random error in Y^*, consistent with the widely held belief. But, crucially, the conditional or independent variable X^* cannot be averaged while simultaneously averaging the dependent variable Y^* for a given value of X^*. In other words, the random error in the conditional variable remains unaddressed and is not averaged away[21]. And, surprisingly, this unbiased random error in the conditional variable (X^*) manifests as a bias in the relation inferred between the variables Y^* and X^*. This *regression bias* is such that the average value of Y^* for a given X^* will be closer to the mean of Y, and if the regression bias is non-linear, it can create an appearance of saturation in Y^* for increasing X^*. This statistical phenomenon is a result of a *regression to the mean*[22,23]. In literature, regression to the mean is commonly understood as a statistical phenomenon where extreme measurements are more likely followed by measurements closer to the mean[23]. However, here we argue that the phenomenon has a more fundamental origin.

The regression to the mean ~~itself~~ is not only a statistical phenomenon, though it ~~can appear~~commonly appears as one. It is not a result of a particular statistical method of inferring relations between parameters, although the particular method will be affected by it. At its core, regression to the mean is a property of the relation between the true value of a stochastic process and its measurement. We explain why this property is a fundamental one in Supplementary Discussion 1.d.

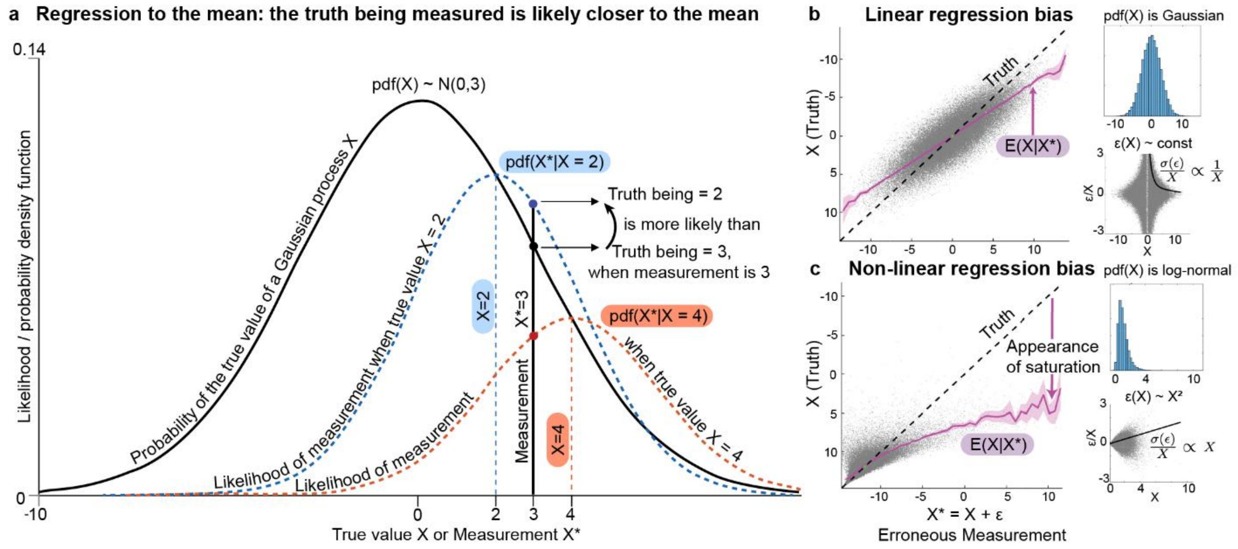

Figure 2 Explaining regression to the mean effect using probability theory and regression bias. a) For a physical parameter X which has an underlying probability distribution, some values are more likely than others, and hence when the measurement is X^*=3 the corresponding true value is more likely to be $X = 2$ than $X = 3$; b) The expected true value of a Gaussian process X has a linear bias when measurement is $X^* = X + \epsilon$ with a constant random error (ϵ) ; c) The expected true value of a log-normal process X has a non-linear bias when the measurement X^* has random error ($\epsilon(X)$)

that varies with the strength of X. ~~Figure reproduced~~This figure is adapted from Sivadas & Sibeck 2022[22], used under CC BY 4.0. The transparent shaded area for regression curves shows a 95% confidence interval.

Any physical process has an underlying probability distribution; therefore, some values of the physical process are more likely than others. For a stochastic process with a Gaussian distribution, the mean value coincides with the most likely or probable value. As a result, any uncertain measurement of the stochastic process is more likely measuring the true value closer to the mean of the stochastic process than more extreme values, by the simple fact that values closer to the mean are more common and hence more likely than the extreme values. In other words, the true value being measured is more likely to be closer to the mean of the stochastic process than the measurement.

Figure 2a demonstrates this result geometrically, where the black line is the probability distribution of a Gaussian process, X. When this process is measured with some uncertainty, the measurement can take the values in the likelihood quantified by the dashed-blue curve when the true value is X=2 and the dashed-red curve when it is X=4. Since the true value is generally inaccessible, the goal of a measurement is to estimate the most probable true value corresponding to the measurement. Hence, when the measurement X*=3 (solid black vertical line), the probability that the corresponding true value X is also equal to 3 (black dot) is remarkably *less likely* than it being equal to 2 (blue dot), simply because X=3 is less likely to occur than X=2 for a Gaussian process with zero mean.

This is the regression to the mean laid bare; the truth being measured regresses towards the mean, and this property of the relation between the truth and measurement manifests in many different forms depending on what one uses the measurements for. In Supplementary Discussion1.c, we explain the regression to the mean effect from five different yet consistent vantage points. For instance, as discussed above, it manifests as 1) a regression bias in the relation inferred between two measurements[22], and 2) a statistical effect where extreme measurements are more likely followed by measurements closer to the mean[23]. Crucially, the regression to the mean is not just a statistical effect as widely believed, but a fundamental logical consequence of the relationship between the true value and its measurement. This result implies that for even a single measurement, the corresponding true value is likely closer to the mean of the stochastic process. For a stochastic process with a general probability distribution, the effect is more aptly named the 'regression to the more-probable'. However, in this paper, we maintain the use of the more popular phrase 'regression to the mean' even when the stochastic process is non-Gaussian.

4. Solar wind uncertainty and error model

Due to regression to the mean, when an extreme value of the solar wind driver is measured at the L1 Lagrange point, it is more likely that the true value of the solar wind driver near Earth is smaller and closer to the mean. Therefore, the geomagnetic response to this smaller driver will also be smaller. And when we do not account for this regression to the mean, as in most previous studies, the smaller geomagnetic response will be wrongly associated with the extreme value of the solar wind driver measured at L1. The more extreme the value of the measured solar wind driver, the greater the mismatch between the true driver and the measured value. As a result, the cross-polar cap potential response to the measured uncertain solar wind driver deviates from linearity during the rare and extreme values and hence, appears to saturate.

To quantitatively test this surprising qualitative argument, we construct an error model $X^* = X(t + dt) + \epsilon$, where X^* is the measured 'uncertain' solar wind driver at L1, and X is the 'true' driving by the shocked solar wind close to the reconnection site. dt is the uncertainty in the propagation time of the solar wind from L1 to the bow shock, and the propagation time of electromagnetic disturbance from the bow shock through to the polar ionosphere. ϵ is the random magnitude changes in the solar wind driver due to spatial and temporal variability influenced by processes during the evolution of the solar wind from L1 and through the bow-shock, and the magnetosheath.

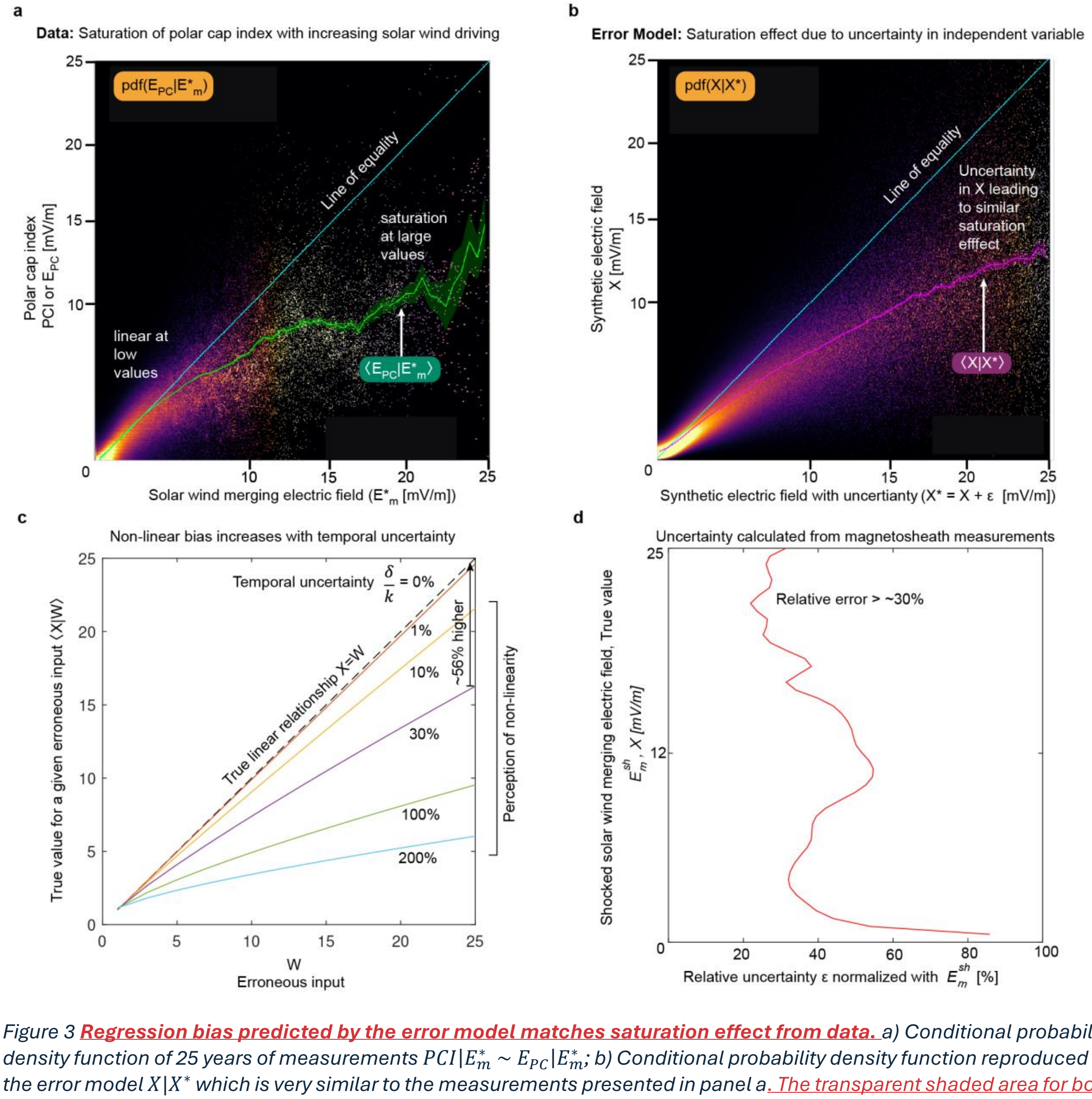

*Figure 3 **Regression bias predicted by the error model matches saturation effect from data.** a) Conditional probability density function of 25 years of measurements $PCI|E_m^* \sim E_{PC}|E_m^*$; b) Conditional probability density function reproduced by the error model $X|X^*$ which is very similar to the measurements presented in panel a. The transparent shaded area for both regression curves shows a 95% confidence interval; c) Non-linear bias caused by uncertainty in time increases with a measure of the uncertainty represented by the dimensionless ratio of the time uncertainty (δ) and the autocorrelation constant (k); d) Quantified relative uncertainty $\epsilon(X)/X$ due to random variations in solar wind driver measured at L1 (E_m^*, X^*) for a measured value of the shocked solar wind driver (E_m^{sh}, X).*

As shown by Sivadas & Sibeck, 2022[22] using a simple Monte-Carlo error model, if X has a Gaussian distribution, and the error ϵ is also Gaussian and independent of X like most instrumental errors (i.e., homoskedastic error[24]), then the likely true value for a given measurement (i.e., the average of X given X^* or $\langle X|X^*\rangle$) will have a linear bias. The purple curve in Figure 2b shows this linear bias as a lower slope than the dashed-black line of equality representing the curve if there were no uncertainty (i.e., the true unbiased relationship). However, suppose the error ϵ depends on X, and varies with the magnitude of X (i.e., heteroskedastic error[24]) like some uncertainties related to the 'problem of definition', and X has a log-normal distribution like many solar wind parameters[25]. In that case, the key point is that the likely true value for a given measurement will have a larger and non-linear bias that mimics a *saturation* of X with increasing X^*. Figure 2c demonstrates this in the purple curve whose slope has a non-linear bias with respect to the black-dashed line of equality representing the true unbiased relationship. Supposing that X is the true solar wind driver near the Earth, and X^* is the uncertain measurement far from Earth, the response Y of the Earth will be driven by the true driver X and, crucially, not by the measurement X^*. If the response Y is linearly related to X (i.e., $Y \propto X$), then naturally, the response Y will also appear to saturate with increasing value of the measurement X^*.

Furthermore, if we keep aside the magnitude uncertainty (i.e., set $\epsilon = 0$), and examine the effect of uncertainty in time dt, we observe that it will lead to a non-linear bias in the average of X given X^* as well, with increasing deviation from linearity with growing uncertainty in time defined by the ratio of the temporal uncertainty (δ) and the autocorrelation time constant (k) of the stochastic process. Figure 3c shows quantitatively how the average of the true value X given the measurement X^* has a non-linear bias in its slope with respect to the dashed-black line of equality representing the true unbiased relationship. This non-linear bias increases with increasing ratio of δ/k, a dimensionless measure of the uncertainty in time. In the Methods section[26], we derive analytically from first principles this non-linear bias and its dependence on temporal uncertainty, which will be relevant for any study with measurement of systems that have a delay in their drivers or response, due to propagation of information, or dynamics of the system e.g., seismic waves from earthquakes[27], or response of medical patients to a specific back pain treatment[28].

For our case of solar wind driving of the geomagnetic system, we make quantitative estimates of the temporal uncertainty dt from previous work and the magnitude uncertainty $\epsilon(X)$ from simultaneous measurements made close to the dayside reconnection site. The calculation of the uncertainty is detailed in the Methods section[26]. Figure 3d presents the relative uncertainty ($\epsilon(X)/X$) with respect to the true shocked solar wind driver in the magnetosheath (X), and it is at least 30% or greater.

Once these uncertainty values are worked into the error model, and X is assumed to have a log-normal distribution with an autocorrelation coefficient of ~100 minutes observed in solar wind measurements, the Monte-Carlo error model solves for X^* and predicts an appearance of saturation of X given X^* (See Figure 3b). This saturated curve, and the conditional probability distribution of X given X^*, matches surprisingly well with the saturation that appears in the data for the polar cap index PCI given the merging electric field estimated from solar wind measurements E_m^* (See Figure 3a). The solution of the error model is consistent with data as viewed through both the conditional expectation $\langle PCI|E_m^*\rangle$ and the conditional probability distribution of $pdf(PCI|E_m^*)$ (See Figure 3a-b). Further validation of the error model with second order statistics of data is presented in the Methods section[26]. Here PCI is analogous to Y in the error model, E_m^* is analogous

to X^*, and E_m^{sh} analogous to X. The similarity in the curves $\langle PCI|E_m^*\rangle$ and $\langle X|X^*\rangle$, indicate that the geomagnetic response is proportional to the true shocked solar wind driver i.e., $PCI \propto E_m^{sh}$ and equivalently $Y \propto X$. The precondition of this non-linear saturation relation between PCI and E_m^* is the heteroskedastic nature of the error in E_m^*, the propagation time uncertainty, and the fact that the solar wind driver is a log-normal process with a particular autocorrelation time constant.

5. Saturation of the cross-polar cap potential explained

This surprising result from the error model shows that uncertainty in the solar wind driver leads to a biased inference that the geomagnetic response saturates with solar wind driving. This uncertainty therefore creates an illusion of saturation when we compare measurements of the solar wind driver to the cross-polar cap index, which is proportional to the cross-polar cap potential[13]. This implies that the ten prevailing theories and models of polar cap potential saturation were developed to explain a biased inference from existing erroneous measurements. As a result, these theories have not been tested or validated against corrected and unbiased data.

We maintain that the original assumption —that the solar wind magnetic field lines connect directly to the polar regions, driving ionospheric convection, and therefore are linearly related to the magnetic fluctuations in ground-magnetometers and thus the polar cap index —holds true[29]. The saturation effect is, surprisingly, an illusion resulting from random error in our measurements of the solar wind driver. This implies that there is currently no evidence to suggest the existence of an upper limit to the energy transferred from the solar wind to the polar ionosphere. In the following section we show that correcting for the effect of uncertainties reveals a linear relationship between the solar wind driver and geomagnetic response, implying we cannot rely on the magnetosphere to dampen the effects of extreme geomagnetic storms.

6. Correcting the effect of uncertainties

There are two ways to address the effect of uncertainties. One approach is to reduce uncertainty in our measurements or estimates of the solar wind driver. For instance, by relying on spacecraft measurements closer to Earth to estimate the driver. Uncertainties are bound to exist in every measurement, and the problem of definition will almost always exist, so the method of reducing uncertainties is unlikely to eliminate them. Alternatively, we can statistically quantify the uncertainties, predict their effect, and offset them from our inference. This method is achievable, albeit challenging, through scientific investigation of the relations between the truth and measurement, and the assumptions that underlie our inferences of the measurements. Here we implement this method of quantifying and correcting the uncertainties.

As shown in the previous two sections, once we calculate uncertainties and develop an error model, it is possible to calculate the non-linear bias caused by the independent variable in the regression analysis. The bias is the consistent deviation between the measurement and the likely true value estimated from the error model $b = X^* - \langle X|X^*\rangle$. Therefore, we can subtract this bias from the erroneous independent variable, and one method to do this is termed "regression calibration"[24,26]. The calibrated variable X^c is simply $X^* - b$. Once we apply this bias correction to the solar wind driver, the relation between the corrected driver ($E_m^c = E_m^* - b$) and the cross-polar cap index is surprisingly linear up to 15 mV/m. Figure 4a shows the saturating green curve formed by using the erroneous data transforms to the linear purple curve after applying this regression calibration. This linearity between E_m^c and PCI is observed after correctly applying the regression calibration operation, which uses as input only $\langle X|X^*\rangle$ from the error model. Hence the linear relationship was

discovered without any assumptions of linearity or non-linearity in the relation between Y and X or PCI and E_m^{sh}. Therefore, regression calibration of the solar wind measurements demonstrates that the true geomagnetic response is linear, and that the saturation of the geomagnetic response is a result of uncertainty in the solar wind driver and the regression to the mean effect. Note that beyond 15 mV/m, the data available is not significant enough to conclude the shape of the relation between the driver and response.

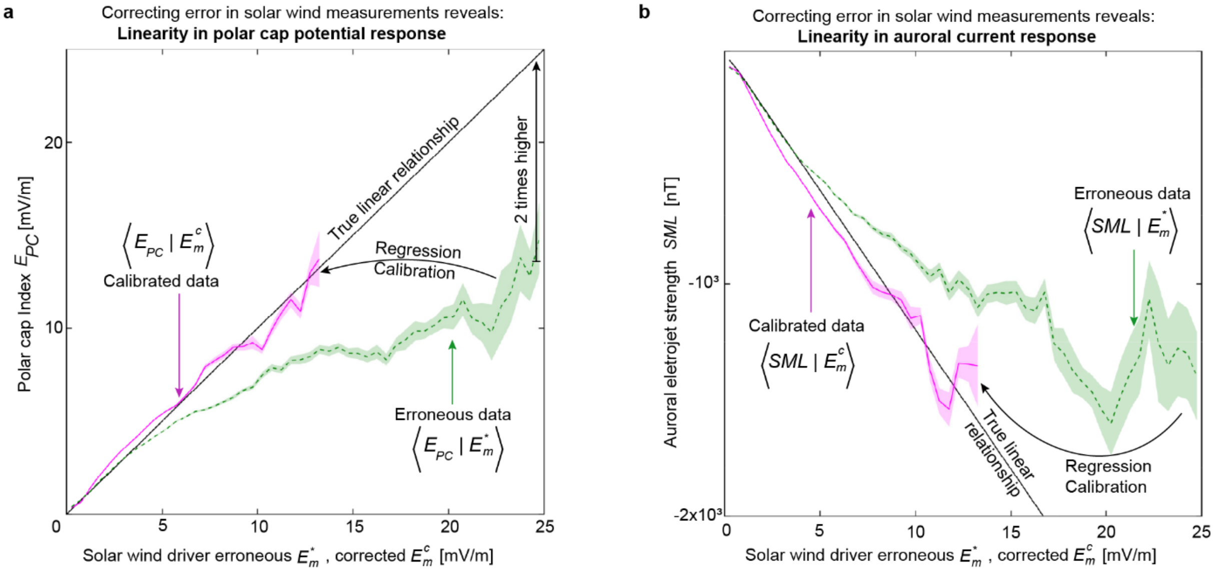

*Figure 4 **Correcting the effect of random errors in solar wind driver values reveals a linear geomagnetic response.** a) Polar cap index varies linearly with solar wind driving on average (purple curve), after correcting the regression bias in the erroneous solar wind driver values (green curve); b) The westward auroral electrojet strength also varies linearly with solar wind driving (E_m^c) on average (purple curve) after the same regression bias correction applied to the erroneous solar wind driver values (E_m^*) (green curve). The transparent shaded area for all the curves shows a 95% confidence interval.*

Our finding that uncertainties in the solar wind driver results in the saturation of the polar cap index, raises the possibility that other measures of the geomagnetic response previously reported to saturate (or not)[30] based on uncertain solar wind driver estimates may be incorrect. The SML index, which is the westward auroral electrojet strength, measured by completely different magnetometers located at high latitudes ($\sim 65°N$) around the planet, also appears to saturate with erroneous solar wind driving estimates. However, the corrected solar wind driver values, calibrated exactly the same way as above, show a linear relationship with the westward auroral electrojet strength as well—a further confirmation that the high-latitude geomagnetic response does not saturate with solar wind driving, once we remove the effect of uncertainties. Figure 4b shows the linear relation of the SML index with corrected solar wind driver in the purple curve, once the regression calibration is applied to the erroneous data in the saturating green curve. It is this linear relationship between the driver and response that a new (or old) physical theory of saturation needs to explain and validate its predictions against. In fact, the development of multiple theories of saturation can be attributed to different inferences from data. Under-sampling bias which is very distinct from the regression to the mean effect, but sometimes confused with it, leads to different saturation limits of the geomagnetic response depending on the sample size of a study (See Supplementary Discussion section 1.a). Further evidence for our conclusion that the saturation effect is the result of uncertainty in the solar wind driver is hiding in plain sight in the space science literature, and is presented in Supplementary Discussion section 1.b.

7. Conclusion and Generalizability

A linear rather than a saturated relation of geomagnetic response to solar wind driver implies extreme space weather will have a greater impact on the planet than previous studies suggests (around 200% more at solar wind strengths extrapolated to 25 mV/m). As we have shown, when correctly interpreted, measurements reveal that the cross-polar cap index and the westward auroral electrojet have a linear relationship with solar wind driving on average, with no saturation limit. It is possible that extreme solar wind driving beyond what we have measured until now may exhibit a saturated geomagnetic response; however, there is no statistical evidence for this so far. Our result is not an alternative theory competing with the existing theories of saturation ~~(Extended Data~~ (Extended Data Table 1), but rather a demonstration that available data does not show evidence for the saturation of geomagnetic response. Hence, it challenges the foundation of existing physical theories of saturation, and they may have to be revisited and validated with the calibrated solar wind driver estimates.

In any field where uncertainty in the driver of a system is significant, and there is interest in the response of the system to extreme and rare instances of driving, the regression to the mean effect can lead to the underestimation of the system's response to extreme drivers. For example, the regression to the mean effect may play a role in climate models, which may underestimate extreme climate events like heatwaves[31], or the impact of strong earthquakes away from their epicenter, or severe medical symptoms and their resolution, like the effectiveness of back-pain treatments[28]. In the above and other fields, biases and even non-linear biases may hide in plain sight and be misunderstood as a physical process.

As artificial intelligence or machine-learning models, which fall under the umbrella of non-linear, non-parametric regression analysis, become popular within physics and other fields with large quantities of measurements, the non-linear bias stemming from heteroskedastic uncertainty or temporal uncertainty is likely present. They may have an impact on prediction and affect any physical inferences drawn from the model outputs, and hence we urge caution in their use and further quantitative investigation on how regression to the mean impacts these models.

The insight that regression to the mean is fundamental to the relation between the truth and its measurement implies that the effect will manifest in different forms within different methods of inference. Hence, all researchers in the fields that attempt to use measurements to infer relations between the physical processes they represent need to be aware of the essence and impact of the logic of regression to the mean. Ignoring this effect can lead researchers and entire fields of inquiry down long, winding paths that deviate from the truth.

MAIN REFERENCES

1. Baker, D. N. What is space weather? *Advances in Space Research* **22**, 7–16 (1998).
2. Gonzalez, W. D. *et al.* What is a geomagnetic storm? *J. Geophys. Res. Space Phys.* **99**, 5771–5792 (1994).
3. Coxon, J. C. *et al.* Extreme Birkeland Currents Are More Likely During Geomagnetic Storms on the Dayside of the Earth. *J. Geophys. Res. Space Phys.* **128**, e2023JA031946 (2023).
4. Eastwood, J. P. *et al.* The Economic Impact of Space Weather: Where Do We Stand? *Risk Analysis* **37**, 206–218 (2017).

5. National Research Council. *Severe Space Weather Events: Understanding Societal and Economic Impacts: A Workshop Report. Severe Space Weather Events--Understanding Societal and Economic Impacts* (National Academies Press, Washington, DC, 2008). doi:10.17226/12507.

6. Dungey, J. W. Interplanetary Magnetic Field and the Auroral Zones. *Phys. Rev. Lett.* **6**, 47 (1961).

7. Cowley, S. ~W. ~H. & Lockwood, M. Excitation and decay of solar wind-driven flows in the magnetosphere-ionosphere system. *Ann. Geophys.* **10**, 103–115 (1992).

8. Kan, J. R. & Lee, L. C. Energy coupling function and solar wind-magnetosphere dynamo. *Geophys. Res. Lett.* **6**, 577–580 (1979).

9. Lockwood, M. Solar Wind—Magnetosphere Coupling Functions: Pitfalls, Limitations, and Applications. *Space Weather* **20**, e2021SW002989 (2022).

10. Troshichev, O. A. & Andrezen, V. G. The relationship between interplanetary quantities and magnetic activity in the southern polar cap. *Planet. Space Sci.* **33**, (1985).

11. Dunham, D. ~W. ISEE-3, the first libration-point satellite. in *Bulletin of the American Astronomical Society* vol. 11 805–806 (1979).

12. Troshichev, O. A. PC index as a ground-based indicator of the solar wind energy incoming into the magnetosphere: (1) relation of PC index to the solar wind electric field EKL. *Frontiers in Astronomy and Space Sciences* vol. 9 Preprint at https://doi.org/10.3389/fspas.2022.1069470 (2022).

13. Troshichev, O., Hayakawa, H., Matsuoka, A., Mukai, T. & Tsuruda, K. Cross polar cap diameter and voltage as a function of PC index and interplanetary quantities . *J. Geophys. Res. Space Phys.* **101**, (1996).

14. Lockwood, M. & McWilliams, K. A. A Survey of 25 Years' Transpolar Voltage Data From the SuperDARN Radar Network and the Expanding-Contracting Polar Cap Model. *J. Geophys. Res. Space Phys.* **126**, e2021JA029554 (2021).

15. Borovsky, J. E., Lavraud, B. & Kuznetsova, M. M. Polar cap potential saturation, dayside reconnection, and changes to the magnetosphere. *J. Geophys. Res. Space Phys.* **114**, n/a-n/a (2009).

16. Shepherd, S. G. Polar cap potential saturation: Observations, theory, and modeling. *J. Atmos. Sol. Terr. Phys.* **69**, 234–248 (2007).

17. Taylor, J. R. (John R. *An Introduction to Error Analysis : The Study of Uncertainties in Physical Measurements*. (University Science Books, 1982).

18. Walsh, B. M., Bhakyapaibul, T. & Zou, Y. Quantifying the Uncertainty of Using Solar Wind Measurements for Geospace Inputs. *J. Geophys. Res. Space Phys.* **124**, 3291–3302 (2019).

19. Morley, S. K., Welling, D. T. & Woodroffe, J. R. Perturbed Input Ensemble Modeling With the Space Weather Modeling Framework. *Space Weather* **16**, 1330–1347 (2018).

20. Milan, S. E., Carter, J. A., Bower, G. E., Fleetham, A. L. & Anderson, B. J. Influence of Off-Sun-Earth Line Distance on the Accuracy of L1 Solar Wind Monitoring. *J. Geophys. Res. Space Phys.* **127**, e2021JA030212 (2022).

21. Borovsky, J. E. Noise, Regression Dilution Bias, and Solar-Wind/Magnetosphere Coupling Studies. *Frontiers in Astronomy and Space Sciences* **0**, 45 (2022).

22. Sivadas, N. & Sibeck, D. G. Regression Bias in Using Solar Wind Measurements. *Frontiers in Astronomy and Space Sciences* **0**, 159 (2022).

23. Barnett, A. G., van der Pols, J. C. & Dobson, A. J. Regression to the mean: what it is and how to deal with it. *Int. J. Epidemiol.* **34**, 215–220 (2005).

24. Carroll, R. J., Ruppert, D., Stefanski, L. A. & Crainiceanu, C. M. Measurement error in nonlinear models: A modern perspective, second edition. *Measurement Error in Nonlinear Models: A Modern Perspective, Second Edition* 1–455 (2006).

25. Veselovsky, I. S., Dmitriev, A. V. & Suvorova, A. V. Lognormal, Normal and Other Distributions Produced by Algebraic Operations in the Solar Wind. *AIP Conf. Proc.* **1216**, 152 (2010).

26. Methods are available at the end of this Main Text.

27. Bonilla, M. G., Mark, R. K. & Lienkaemper, J. J. Statistical relations among earthquake magnitude, surface rupture length, and surface fault displacement. *Bulletin of the Seismological Society of America* **74**, 2379–2411 (1984).

28. Whitney, C. W. & Von Korff, M. Regression to the mean in treated versus untreated chronic pain. *Pain* **50**, 281–285 (1992).

29. Vasyliunas, V. M. Concepts of Magnetospheric Convection. in *The Magnetospheres of the Earth and Jupiter* (ed. Formisano, V.) 179–188 (Springer Netherlands, Dordrecht, 1975).

30. Borovsky, J. E. On the Saturation (or Not) of Geomagnetic Indices. *Frontiers in Astronomy and Space Sciences* **8**, 175 (2021).

31. Kornhuber, K., Bartusek, S., Seager, R., Schellnhuber, H. J. & Ting, M. Global emergence of regional heatwave hotspots outpaces climate model simulations. *Proceedings of the National Academy of Sciences* **121**, e2411258121 (2024).

METHODS

Overview

In this section, we discuss the methods used to demonstrate that polar cap potential saturation is a result of solar wind measurement uncertainty, calibrate the error to reveal the linear response of the polar cap potential and auroral current strengths due to solar wind forcing, and present the analytical derivation of uncertainty in measurement times causing a perception of non-linearity in a linear system's correlation response. The most common symbols used are explained in the Extended Data Table 2.

Solar wind measurement uncertainty

There are several solar wind coupling functions that attempt to quantify the driving, one important one is the solar wind merging electric field or merging geoeffective field E_m^*[8,16].

$$E_m^* = V_{sw} B_{T,sw} \sin^2(\theta_{sw}/2) \quad [1.1]$$

This field is also known as the Kan-Lee electric field and is calculated from solar wind parameters at Lagrange point L1 alone[8]. In equation 1.1, V_{sw} is the solar wind speed in km/s, $B_{T,sw} = \sqrt{B_y^2 + B_z^2}$ is the transverse magnitude of the interplanetary magnetic field (IMF) in nT, and $\theta_{sw} = \tan^{-1}(B_y/B_z)$ is the transverse IMF clock angle in radians. E_m is a positive-valued quantity with the units of the electric field [mV/m], reflecting the assumption that energy flows only from the solar wind to the magnetosphere. The above quantities are measured in Geocentric Solar Magnetospheric (GSM) coordinates using spacecrafts orbiting the L1 point ~230 earth Radii (RE) upstream from Earth. The GSM coordinates are convenient for studying the effects of the IMF components on magnetospheric and ionospheric phenomena. Its X-axis points towards the Sun, and the Z-axis is the projection of the Earth's magnetic dipole axis onto the plane perpendicular to the X-axis. For purely southward IMF, the merging electric field E_m^* is equal to the solar wind electric field $E_{sw} = V_{x,sw} B_z$, where $V_{x,sw}$ is a component of the solar wind velocity along the x-direction.

The dawn-dusk portion of the shocked solar wind convection electric field maps along the equipotential magnetic field lines and drives the plasma convection down in the polar cap ionosphere. The convection corresponds to an electric field across the polar cap in the rest-frame of the Earth (E_{PC}). The polar cap (PC) index is a measure of this field and part of the energy input into the Earth's magnetosphere. The index has gone through many iterations over the past 50 years, but it is essentially the maximum amplitude of variations observed from magnetometers near the south and north pole[12,32,33]. The current version of the index is scaled on a statistical basis of magnetic variations to the merging electric field, such that the index is highly correlated to the merging electric field. This makes the PC index independent of daily and seasonal variations and the local ionospheric properties. PCN is the index derived from the magnetometer in the northern polar cap, while PCS is derived from the southern polar cap. We use the PCI index as a proxy for E_{PC} (i.e. $PCI \sim E_{PC}$), as it combines both the indices to provide a positive-valued index, which is more accurate than the individual indices[33]. PCI=0.5*(PCS+PCN), with a condition that negative values of either PCS or PCN are set to 0. PCI or E_{PC} also has the units of the electric field, and several statistical analyses in literature reveal that it is linearly proportional on average to the electric potential across the polar cap, called the cross-polar cap potential[13,34]. Fluctuations

in PCI can occur due to night-side magnetosphere processes[35], but the above relation is still maintained on average.

The Kan-Lee electric field is an approximation of the component of the reconnection electric field ($E_R = V_{sh}B_{sh}\sin(\theta'/2)$) perpendicular to the geomagnetic field that creates the cross-polar cap electric field (which is equal to $V_{sh}B_{sh}\sin^2(\theta'/2)$ due to a geometrical argument presented first by Kan and Lee)[8]. The approximation assumes that the shear angle between the magnetosheath and magnetospheric magnetic field θ' is equal to θ_{sw}. Furthermore, the approximation assumes $V_{sh}B_{sh} \cong V_{sw}B_{T,sw}$, which is true most of the time 1) as the solar wind velocity component tangential to the bowshock is negligible compared to the normal component, and 2) as the tangential component of the electric field across a normal MHD shock is conserved. This allows the Kan-Lee electric field (E_m^*) to estimate the cross polar electric field E_{PC} with just solar wind parameters. Our examination of data shows that for electric field values <3mV/m, which is 95% of all available data points in 25 years, the Kan-Lee electric field (E_m^*) propagated (or time-shifted) to the polar cap ionosphere from the L1 point is linearly proportional to the cross polar electric field E_{PC}. This is well-documented in literature and is also observed for other forms of the solar wind coupling functions and polar cap index [16,33,36]. However the fact that E_m^* is only an approximation of the true driver of the magnetosphere i.e., the shocked solar wind plasma $E_m^{sh} = V_{sh}B_{sh}\sin^2(\theta'/2)$, whose value is not easily available to us, leads to the uncertainty and non-linear regression bias discussed in this work. Instead, we only have an erroneous estimate of the true coupling function approximately propagated to the polar cap, E_m^*. Hence, we need to reinterpret the literature as saying that low values of the erroneous estimate of the solar wind strengths E_m^* (not E_m^{sh}) correlate linearly with the polar cap potential. And at high values of E_m^*, the polar cap potential saturates. The saturation seems to have the following functional form[33]:

$$E_{PC} = \frac{E_m^*}{\sqrt{1 + (E_m^*/E_0)^2}}$$

We calculate E_m^* by using WIND satellite measurements published in the OMNIWeb database[37]. The database provides the values corrected for the propagation delay of the solar wind from L1 to the bow-shock nose [38–40]. Then as commonly done, we apply a further correction of a constant delay of ~17 minutes to account for the propagation delay from the nose to the polar cap ionosphere[20,41]. Previous literature that discusses the polar cap potential saturation problem estimates E_m^* similarly. We use the WIND data from 1995 to 2019 and the polar cap indices from the same time range. Both data are 1-minute averages, larger time averages will lead to additional uncertainties[42]. We only use data samples when both WIND measurements and polar cap indices are available.

E_m^* can differ from the shocked solar wind driver of the magnetoshere E_m^{sh} in two ways. One is through a consistent deterministic bias, that is determined by the bow shock that slows down the plasma. This deterministic bias is nearly zero as the tangential (and the largest) component of the electric field across the bow-shock remains unchanged. We do not concern ourselves with this deterministic bias, as it will manifest in the data as a physical effect anyway. However, E_m^* can also differ randomly from the true solar wind driver E_m^{sh}, due to fluctuations in the plasma properties caused by physical processes between L1 and the reconnection site, acting in random

directions. These random fluctuations contribute to the uncertainty in E_m^* when we use it as a proxy for the true driver E_m^{sh}. This random error is concerning, as it does not average away, and manifests as a regression bias in data analysis and is easily confused as a physical effect, hence we calculate and correct for it.

We categorize the random uncertainty in E_m^* relative to the true value E_m^{sh} into three primary sources:

1. Uncertainty in propagation delay of the solar wind from L1 to the bow-shock nose (dt_1)
2. Uncertainty in the propagation delay of the effect of solar wind forcing from bow-shock nose to the polar cap ionosphere (dt_2)
3. Random variability in the shocked solar wind due to spatial variation in the incoming solar wind and transformations of its plasma parameters during propagation through the bow shock and the magnetosheath

Knowing the statistical distribution of the above uncertainty will allow us to construct a stochastic model of the estimate E_m^* as a function of the true driver E_m^{sh} mapped to the polar cap ionosphere. In the following subsection, we develop a statistical error model of the estimate of the true shocked solar wind that drives the cross-polar cap convection from an estimate of its stochastic properties and uncertainty distributions. For the calculated uncertainties (Figure 3d), the model predicts the polar cap potential saturation, which is strikingly similar to data from 25 years of observations (see Figure 1b and Figure 3a-b).

Statistical error model

To distinguish between data and model, we will replace E_m^{sh} with X when referring to the random variable corresponding to the shocked solar wind driver in the statistical model. We also replace the polar cap index PCI, which is proportional to the convection electric field E_{PC} in the ionosphere with a counterpart in the model Y_{PC}. Hence, in the model, X is the shocked solar wind driver accurately time-shifted to the polar cap, and X^* is its erroneous estimate (i.e. solar wind driver measured at L1 or E_m^*). We hypothesize the following statistical error model:

$$\mathrm{X}^*(t) = \mathrm{X}(t + dt_1 + dt_2) + \varepsilon(t) \qquad [1.2]$$

We assume that X^*, X, dt_1, dt_2 , and ε are stochastic processes. In other words, they are each a collection of random variables in time ($t \in T$) with an associated probability distribution that determines the random value it might take at a given time t. Below we present our estimates of the probability distributions and autocorrelation functions of these stochastic processes. Using these estimates of X, dt_1, dt_2, and ε, we calculate X^*. After which, we validate the model results by comparing them with the data E_m^*.

Input X: If reconnected open-field lines in the polar cap region do not have large parallel resistances, accurate mapping of the shocked solar wind electric field onto the polar cap should result in $X,$ such that $Y_{PC} \propto$ X. In agreement with this, in data, we see that, the probability density function (pdf) of the PCI index or E_{PC} (and hence Y_{PC}) and solar wind driver E_m^* are very similar, implying the distribution of Y_{PC} is also similar to X. As a result, we assume that the pdf of X is similar to the pdf of E_{PC} (and E_m^*) that we can estimate from data. Note that this is not surprising since E_{PC} is constructed to be highly correlated with E_m^*, and from Kan and Lee's arguments E_m^* is also correlated with E_m^{sh}, and hence all these parameters have similar probability distribution

functions. (Though we have measurements of E_m^{sh} from near-Earth orbiting spacecraft, they are discontinuous and hence do not directly provide an unbiased pdf of X.) Like many solar wind parameters, E_{PC} and E_m^* can be approximated to be a lognormal distribution[25]. Therefore, we assume the pdf of model input X to be a lognormal distribution that closely fits the pdf of E_{PC} from data (See Extended Data Figure 1a~~Extended Data~~). We also assume the adjacent values of X in time are correlated similarly to adjacent values of E_{PC} in time (See Extended Data Figure 1b~~Extended Data~~), as fluctuations in time of shocked solar wind electric field should correlate with that of the cross-polar cap electric field in the ionosphere. In other words, **we assume that in our model, X shares the stochastic properties of the polar cap index or electric field E_{PC}**. If the assumption fails, the results of the model, particularly the second order statistics, will be inconsistent with what is observed in the data. Finally, we assume X is a stationary process, i.e., its probability distribution and autocorrelation function does not change with time.

Uncertainty in propagation delay from L1 to Nose dt_1: Solar wind parameters measured upstream are time-shifted to account for the delay in propagation of the wind to the bow-shock nose. Case and Wild estimate the uncertainty to be on the order of minutes [43]. Based on their results, we assume dt_1 to be an independent random process with a pdf of a Student's t-distribution with shape factor 1.3, mean 0, and standard deviation ~8 minutes (See Extended Data Figure 1c). The distribution is zero-mean and has a longer tail than the normal distribution.

Uncertainty in propagation delay from nose to polar cap dt_2: Changes in the day-side shocked solar wind electric field propagate along equipotential open magnetic field lines to the polar caps. The delay in this propagation is on average 20 minutes but can vary from -5 to 50 minutes[34]. Historically, researchers have used a constant propagation delay (t_2) of about ~20 minutes for this stage of propagation. However, the uncertainty of propagation time here is significant. We model the pdf of t_2 as a Weibull distribution with mean ~ 17 minutes[20], standard deviation ~ 25 minutes, and shape factor 1.3 (See Extended Data Figure 1c). The Weibull distribution keeps the total delay $t_2 + dt_2$ positive and captures the broad spread in the propagation delay documented by Stauning[33,34].

Magnitude uncertainty ε: There are several other reasons for the magnitude of the shocked solar wind driver to be randomly different from its proxy measured at the L1. The IMF clock angle could change substantially after crossing the bow shock, spatial variations in the solar wind can lead to a different part of the wind interacting with the Earth's magnetosphere, and changes in the magnetospheric state or its history can lead to changes in the local plasma and field conditions in magnetosheath. As the solar wind strength increases, the random variation in the field can increase due to the increased spatial structuring of the solar wind, and any clock angle variation during increased field strength can lead to larger variations in geoeffectiveness.

To estimate these random variations, we use direct evidence from a database of simultaneous measurements of plasma and field strengths in the magnetosheath. Using a gradient boost classification algorithm, measurements from near-Earth satellites such as THEMIS, MMS, DoubleStar, and Cluster, are classified into solar wind, magnetosheath, and magnetosphere regions[44]. Our interest is in the measurements made within the magnetosheath, close to the magnetopause in the subsolar region (|Y|<5 R_E, and |Z| < 5 R_E). The distance from the magnetopause boundary is determined using a machine-learning based empirical model of the boundary, which performs better than other magnetopause models available in literature[45]. For

specific values of the magnetosheath measurements (E_m^{sh}), we calculate the variance in the measurements made at L1 and time-shifted to the bow shock (E_m^*). This variance is an estimate of the uncertainty in the solar wind driver E_m^* for a given value of the shocked solar wind driver E_m^{sh}. The "magnitude uncertainty" ε is set to the same variance, and we model it as a zero-mean Gaussian with a standard deviation that varies with the magnitude of X according to data. The magnitude uncertainty is not constant with the strength of the shocked solar wind driver, instead it increases until E_m^{sh}~12 mV/m, after which the statistics become poor and an accurate estimate of the variance becomes challenging. In this regime, we estimate the uncertainty in the solar wind driver relative to measurements of near-Earth satellites just upstream of the bow shock (~10<X<50 R_E). This uncertainty will be the minimum uncertainty for values >12 mV/m (i.e., a conservative estimate of the uncertainty), and this remains roughly the same with increasing shocked solar wind driver value. Figure 3d shows the relative error, i.e. $\sigma(\varepsilon)/X$, and how that varies with X. The 'heteroskedastic' behavior of this uncertainty is consistent with the observed statistical variations in the difference between solar wind driver and the polar cap index shown in Extended Data Figure 2d-e. The variation in their difference increases up to a polar cap index of ~12 mV/m and then remains constant. We also assume that ε has an autocorrelation function similar to that of the difference between the observed solar wind driver E_m^* and the polar cap index $PCI \sim E_{PC}$.

Using the above estimates of uncertainties, we generate an ensemble of time series of X, dt_1, dt_2, and ε, and calculate the corresponding time series of the erroneous estimate X^* using equation [1.2]. The statistical properties of the model output agree remarkably with that of the data.

Validation of Error Model

We validate the error model by comparing the second order statistics of the error model parameters with that of their counterparts in data. The error model predicts the pdf of the solar wind driver E_m^*, the standard deviation of the normalized error, the conditional normalized error distribution, and finally the regression bias stemming from the regression to the mean effect. ~~Details of~~ This validation ~~are~~is the rationale for using the error model and is presented in the Supplementary Methods Section 2.a. Supplementary Methods 2.c summarizes the full computer code used. Additionally, we show a sensitivity analysis of the non-linear regression bias, which increases with increasing uncertainty in the time of propagation (See Figure 3c), and with increasing uncertainty in the magnitude uncertainty ε (See Extended Data Figure 4).

Correcting the error

The regression bias $b = X^* - \langle X|X^* \rangle$, which is the consistent deviation between the measurement and the likely true value given the measurement. The erroneous measurement X^* can now be corrected to X^c by subtracting the bias from it $X^c = X^* - b$, which simply reduces to $X^c =$ $\langle X|X^* \rangle$. Crucially, from the model output, we can calculate the conditional expectation of the true value given the erroneous estimate: $f_r(x) = \langle X|X^* = x \rangle$, i.e., we have a statistical, functional relationship between the true value and the erroneous measurement. For the particular assumptions of uncertainties in measurement, $f_r(x)$ is the best estimate of the true value (X^c or E_m^c) given the erroneous value (X^* or E_m^*). Therefore, $f_r(x)$ can be used to statistically correct the erroneous estimate E_m^* to a most likely estimate of the true value E_m^c. E_m^c is useful for revealing the true statistical correlation with other variables, like the polar cap index E_{PC} or auroral current strengths SML. The process of statistically correcting for the uncertainty using the conditional expectation

of the true value given the erroneous estimate: $E_m^c = f_r(\mathrm{E}_m^*)$ for an unbiased regression analysis is called regression calibration[24]. Figure 4 shows the results of this calibration. SML is the westward auroral electrojet index from the SuperMAG database, which is a measure of the westward currents flowing in the auroral regions[46,47]. It is calculated using ground magnetometers and procedures completely independent of the calculation of the polar cap index. Since regression calibration of E_m^* to E_m^c, results in a linear relationship between both E_m^c and E_{PC} (Figure 4A) and E_m^c and SML (Figure 4B), it suggests our findings are independent of the construction of the ionospheric response variables. We note here that the confidence intervals shown in Figures 1-4 only provide an estimate of the reliability of the regression procedure, i.e., it means 95% probability that repeating the procedure will contain the result and does not mean the true value or the true regression curve will lie within the confidence interval with a 95% probability.

Temporal uncertainty can lead to a non-linear regression function

In Supplementary Methods Section 2.b we analytically derive the following general result that temporal uncertainty in measurement can lead to a perception of non-linearity in a linear system's correlation response. For a linear system with response Y, input X, and the input with measurement error $W = X(t + \Delta)$, the biased system response $\langle Y|W\rangle$ is a function $f_r^*(w, \frac{\sigma_\delta}{k})$. Here σ_δ is a measure of the random uncertainty Δ in the time of measurement. And k is the autocorrelation time constant. The system response is a function of the ratio $\frac{\sigma_\delta}{k}$. This ratio is a measure of uncertainty in the time of measurement. We numerically integrate the function, which is fully described in equation [1.12] in the Supplementary Methods Section 2.b, within the ranges of Δ for specific values of $\frac{\sigma_\delta}{k}$ and generate Figure 3c. The pdf of X is similar to that assumed in the previous section, with the mean $\mu_X = 1.12$ and standard deviation $\sigma_X = 1.15$. The figure shows that $f_r^*(w, \frac{\sigma_\delta}{k})$ varies non-linearly with w when the temporal uncertainty ratio is larger than 0%, compared to $f_r(x) = x$ (which is linear) when $W = X$ or the temporal uncertainty ratio is 0%. Therefore, we have found that the regression function $\langle Y|W\rangle = f_r^*(w, \frac{\sigma_\delta}{k})$ is non-linear with respect to w for values of $\frac{\sigma_\delta}{k} > 0$.

Data Availability

All data we have used is publicly available. We thank GSFC/SPDF OMNIWeb service for the WIND spacecraft measurements [48]. The WIND spacecraft measurements of the solar wind propagated to the bow shock can be accessed from https://spdf.gsfc.nasa.gov/pub/data/omni/high_res_omni/sc_specific/. The MMS, Cluster, DoubleStar, and THEMIS data can be accessed from GSFC/SPDF web service https://spdf.gsfc.nasa.gov/. The polar cap index values PCN and PCS were downloaded from https://pcindex.org/archive. We thank Dr. Oleg Troshichev of the Arctic and Antarctic Research Institute for this data (https://pcindex.org/contacts). The auroral electrojet indices were downloaded from the SuperMAG database https://supermag.jhuapl.edu/indices/. For SuperMAG indices we gratefully acknowledge the SuperMAG collaborators (https://supermag.jhuapl.edu/info/?page=acknowledgement). This Zenodo repository archives the data used in the ~~manuscript~~manuscript[49] (https://doi.org/10.5281/zenodo.17546718).

Computer Code

All the relevant data and the MATLAB and Python codes to process and visualize them have been curated and uploaded in the following GitHub repository https://github.com/nithinsivadas/polar-cap-saturation.git, and archived ~~here~~here[50] (https://doi.org/10.5281/zenodo.17559999). For non-MATLAB and non-Python users the code and its outputs are accessible as html files with detailed comments and plots (See https://nithinsivadas.github.io/polar-cap-saturation/).

METHODS REFERENCES

32. Troshichev, O. & Sormakov, D. PC index as a proxy of the solar wind energy that entered into the magnetosphere: 2. Relation to the interplanetary electric field e KL before substorm onset. *Earth, Planets and Space* **67**, 1–11 (2015).

33. Stauning, P. The polar cap (PC) index combination, PCC: Relations to solar wind properties and global magnetic disturbances. *Journal of Space Weather and Space Climate* **11**, 19 (2021).

34. Stauning, P. The Polar Cap PC Indices: Relations to Solar Wind and Global Disturbances. in *Exploring the Solar Wind* (InTech, 2012). doi:10.5772/37359.

35. Lockwood, M., Cowley, S. W. H. & Freeman, M. P. The excitation of plasma convection in the high-latitude ionosphere. *J. Geophys. Res. Space Phys.* **95**, 7961–7972 (1990).

36. Doyle, M. A. & Burke, W. J. S3-2 measurements of the polar cap potential. *J. Geophys. Res. Space Phys.* **88**, 9125–9133 (1983).

37. King, J. H. & Papitashvili, N. E. Solar wind spatial scales in and comparisons of hourly Wind and ACE plasma and magnetic field data. *J. Geophys. Res. Space Phys.* **110**, 2104 (2005).

38. Weimer, D. R. *et al.* Predicting interplanetary magnetic field (IMF) propagation delay times using the minimum variance technique. *J. Geophys. Res. Space Phys.* **108**, 1026 (2003).

39. Haaland, S., Paschmann, G. & Sonnerup, B. U. Ö. Comment on "A new interpretation of Weimer et al.'s solar wind propagation delay technique" by Bargatze et al. *J. Geophys. Res. Space Phys.* **111**, 6102 (2006).

40. Knetter, T., Neubauer, F. M., Horbury, T. & Balogh, A. Four-point discontinuity observations using Cluster magnetic field data: A statistical survey. *J. Geophys. Res. Space Phys.* **109**, 6102 (2004).

41. Troshichev, O. A., Janzhura, A. S. & Stauning, P. Magnetic activity in the polar caps: Relation to sudden changes in the solar wind dynamic pressure. *J. Geophys. Res. Space Phys.* **112**, 11202 (2007).

42. Laundal, K. M. *et al.* Time-scale dependence of solar wind-based regression models of ionospheric electrodynamics. *Scientific Reports 2020 10:1* **10**, 1–9 (2020).

43. Case, N. A. & Wild, J. A. A statistical comparison of solar wind propagation delays derived from multispacecraft techniques. *J. Geophys. Res. Space Phys.* **117**, 2101 (2012).

44. Nguyen, G. *et al.* Massive Multi-Mission Statistical Study and Analytical Modeling of the Earth's Magnetopause: 1. A Gradient Boosting Based Automatic Detection of Near-Earth Regions. *J. Geophys. Res. Space Phys.* **127**, (2022).

45. Michotte de Welle, B. *et al.* Global Environmental Constraints on Magnetic Reconnection at the Magnetopause From In Situ Measurements. *J. Geophys. Res. Space Phys.* **129**, e2023JA032098 (2024).

46. Gjerloev, J. W. The SuperMAG data processing technique. *J. Geophys. Res. Space Phys.* **117**, 9213 (2012).

47. Newell, P. T. & Gjerloev, J. W. Evaluation of SuperMAG auroral electrojet indices as indicators of substorms and auroral power. *J. Geophys. Res. Space Phys.* **116**, (2011).

48. Lepping, R. P. *et al.* The WIND magnetic field investigation. *Space Science Reviews 1995 71:1* **71**, 207–229 (1995).

49. Sivadas, N. *et al.* Simultaneous measurements of solar wind from L1, shocked solar wind from Magnetosheath, and measures of geomagnetic response from ground-based indices . Preprint at https://doi.org/10.5281/zenodo.17546718 (2026).

50. Sivadas, N. Code for Regression to the Mean of Extreme Geomagnetic Storms . Preprint at https://doi.org/10.5281/zenodo.17559999 (2026).

51. Boyle, C. B., Reiff, P. H. & Hairston, M. R. Empirical polar cap potentials. *J. Geophys. Res. Space Phys.* **102**, 111–125 (1997).

~~50~~52. Pulkkinen, T. I. *et al.* Magnetosheath control of solar wind-magnetosphere coupling efficiency. *J. Geophys. Res. Space Phys.* **121**, 8728–8739 (2016).

~~51~~53. Barnett, A. G., van der Pols, J. C. & Dobson, A. J. Regression to the mean: what it is and how to deal with it. *Int. J. Epidemiol.* **34**, 215–220 (2005).

~~52~~54. Carroll, R. J., Ruppert, D., Stefanski, L. A. & Crainiceanu, C. M. Measurement error in nonlinear models: A modern perspective, second edition. *Measurement Error in Nonlinear Models: A Modern Perspective, Second Edition* 1–455 (2006).

55. Vörös, Z. *et al.* Probability density functions for the variable solar wind near the solar cycle minimum. *J. Geophys. Res. Space Phys.* **120**, 6152–6166 (2015).

56. Borovsky, J. E. Noise and Solar-Wind/Magnetosphere Coupling Studies: Data. *Frontiers in Astronomy and Space Sciences* **0**, (2022).

57. Reiff, P. H., Spiro, R. W. & Hill, T. W. Dependence of polarcap potential drop on interplanetary parameters. *J. Geophys. Res.* **86**, 7639–7648 (1981).

58. Wygant, J. R., Torbert, R. B. & Mozer, F. S. Comparison of S3-3 polar cap potential drops with the interplanetary magnetic field and models of magnetopause reconnection. *J. Geophys. Res. Space Phys.* **88**, 5727–5735 (1983).

59. Weimer, D. R., Reinleitner, L. A., Kan, J. R., Zhu, L. & Akasofu, S.-I. Saturation of the auroral electrojet current and the polar cap potential. *J. Geophys. Res. Space Phys.* **95**, 18981–18987 (1990).

60. Russell, C. T., Luhmann, J. G. & Lu, G. Nonlinear response of the polar ionosphere to large values of the interplanetary electric field. *J. Geophys. Res. Space Phys.* **106**, 18495–18504 (2001).

61. Liemohn, M. W. & Ridley, A. J. Comment on 'Nonlinear response of the polar ionosphere to large values of the interplanetary electric field' by C. T. Russell et al. *Journal of Geophysical Research: Space Physics* vol. 107 SIA 13-1 Preprint at https://doi.org/10.1029/2002JA009440 (2002).

62. Shepherd, S. G., Greenwald, R. A. & Ruohoniemi, J. M. Cross polar cap potentials measured with Super Dual Auroral Radar Network during quasi-steady solar wind and interplanetary magnetic field conditions. *J. Geophys. Res. Space Phys.* **107**, SMP 5-1 (2002).

63. Nagatsuma, T. Saturation of polar cap potential by intense solar wind electric fields. *Geophys. Res. Lett.* **29**, 62–1 (2002).

64. Hairston, M. R., Drake, K. A. & Skoug, R. Saturation of the ionospheric polar cap potential during the October–November 2003 superstorms. *J. Geophys. Res. Space Phys.* **110**, 9–26 (2005).

~~53~~65. Hill, T. W., Dessler, A. J. & Wolf, R. A. Mercury and Mars: The role of ionospheric conductivity in the acceleration of magnetospheric particles. *Geophys. Res. Lett.* **3**, 429–432 (1976).

~~54~~66. Siscoe, G. L. *et al.* Hill model of transpolar potential saturation: Comparisons with MHD simulations. *J. Geophys. Res. Space Phys.* **107**, (2002).

~~55~~67. Merkin, V. G. *et al.* Global MHD simulations of the strongly driven magnetosphere: Modeling of the transpolar potential saturation. *J. Geophys. Res. Space Phys.* **110**, (2005).

~~56~~68. Merkin, V. G. *et al.* Relationship between the ionospheric conductance, field aligned current, and magnetopause geometry: Global MHD simulations. *Planet. Space Sci.* **53**, 873–879 (2005).

~~57~~69. Raeder, J. & Lu, G. Polar cap potential saturation during large geomagnetic storms. *Advances in Space Research* **36**, 1804–1808 (2005).

~~58~~70. Ridley, A. J. A new formulation for the ionospheric cross polar cap potential including saturation effects. *Ann. Geophys.* **23**, 3533–3547 (2005).

~~59~~71. Lopez, R. E. *et al.* Role of magnetosheath force balance in regulating the dayside reconnection potential. *J. Geophys. Res. Space Phys.* **115**, 12216 (2010).

~~60~~72. Pudovkin, M. I., Zaitseva, S. A., Bazhenova, T. A. & Andrezen, V. G. Electric fields and currents in the earth's polar caps. *Planet. Space Sci.* **33**, 407–414 (1985).

~~61~~73. Raeder, J., Wang, Y. L., Fuller-Rowell, T. J. & Singer, H. J. Global Simulation of Magnetospheric Space Weather Effects of the Bastille Day Storm. *Solar Physics 2001 204:1* **204**, 323–337 (2001).

~~62~~74. Hernandez, S., Lopez, R. E., Wiltberger, M. & Lyon, J. G. MHD Simulations of Solar Wind-Geospace Coupling. in *AGU Spring Meeting Abstracts* SM51C-03 (SAO/NASA Astrophysics Data System, 2007).

~~63~~75. Winglee, R. M., Chua, D., Brittnacher, M., Parks, G. K. & Lu, G. Global impact of ionospheric outflows on the dynamics of the magnetosphere and cross-polar cap potential. *J. Geophys. Res.* **107**, (2002).

~~64~~76. Siscoe, G. L., Crooker, N. U. & Siebert, K. D. Transpolar potential saturation: Roles of region 1 current system and solar wind ram pressure. *J. Geophys. Res. Space Phys.* **107**, (2002).

~~65~~77. Ridley, A. J. Alfvén wings at Earth's magnetosphere under strong interplanetary magnetic fields. *Ann. Geophys.* **25**, 533–542 (2007).

~~66~~78. Kivelson, M. G. & Ridley, A. J. Saturation of the polar cap potential: Inference from Alfvén wing arguments. *J. Geophys. Res. Space Phys.* **113**, 5214 (2008).

67. Reiff, P. H., Spiro, R. W. & Hill, T. W. Dependence of polarcap potential drop on interplanetary parameters. *J Geophys Res* **86**, 7639–7648 (1981).

68. Wygant, J. R., Torbert, R. B. & Mozer, F. S. Comparison of S3-3 polar cap potential drops with the interplanetary magnetic field and models of magnetopause reconnection. *J Geophys Res Space Phys* **88**, 5727–5735 (1983).

69. Weimer, D. R., Reinleitner, L. A., Kan, J. R., Zhu, L. & Akasofu, S.-I. Saturation of the auroral electrojet current and the polar cap potential. *J Geophys Res Space Phys* **95**, 18981–18987 (1990).

70. Russell, C. T., Luhmann, J. G. & Lu, G. Nonlinear response of the polar ionosphere to large values of the interplanetary electric field. *J Geophys Res Space Phys* **106**, 18495–18504 (2001).

71. Liemohn, M. W. & Ridley, A. J. Comment on 'Nonlinear response of the polar ionosphere to large values of the interplanetary electric field' by C. T. Russell et al. *Journal of Geophysical Research: Space Physics* vol. 107 SIA 13-1 Preprint at https://doi.org/10.1029/2002JA009440 (2002).

72. Shepherd, S. G., Greenwald, R. A. & Ruohoniemi, J. M. Cross polar cap potentials measured with Super Dual Auroral Radar Network during quasi-steady solar wind and interplanetary magnetic field conditions. *J Geophys Res Space Phys* **107**, SMP 5-1 (2002).

73. Nagatsuma, T. Saturation of polar cap potential by intense solar wind electric fields. *Geophys Res Lett* **29**, 62–1 (2002).

74. Hairston, M. R., Drake, K. A. & Skoug, R. Saturation of the ionospheric polar cap potential during the October–November 2003 superstorms. *J Geophys Res Space Phys* **110**, 9–26 (2005).

Funding:

NASA Heliophysics Guest Investigator Program under Grant # 80NSSC23K0446 (NS,BF)

NSF AGS GEM Award #2453576 (NS, DSO)

UKRI STFC through the Ernest Rutherford Fellowship (ST/X003663/1) (MTW)

NASA Heliophysics Participating Investigator Program under Grant #WBS516741.01.24.01.03 (DS)

Author contributions:

Conceptualization: NS, VS

Methodology: NS

Investigation: NS, VS, DSO, BF

Formal Analysis: NS, VS

Visualization: NS

Software: NS, BMW

Validation: NS, MTW

Funding acquisition: NS, DS, DSO, BF
Project administration: NS, DS
Supervision: DS
Writing – original draft: NS, VS
Writing – review & editing: NS, VS, DS, MTW, BMW, DSO, BF

Competing interests: Authors declare that they have no competing interests.

Materials & Correspondence: Nithin Sivadas, nithinsivadas90@gmail.com

Supplementary Materials

Supplementary Information

1. Supplementary Discussion
    a. Historical development of the polar cap potential saturation problem
    b. Evidence in literature
    c. Regression to the mean from different vantage points
    d. Regression to the mean as a fundamental property of the relation between truth and measurement
2. Supplementary Methods
    a. Validation of the Error Model
    b. Analytical derivation of non-linear regression bias due to uncertainty in time
    c. Summary of data analysis procedure in the computer code

Extended Data Figures 1 to ~~8~~5

Extended Data Table 1 to 2

SUPPLEMENTARY INFORMATION

1. Supplementary Discussion

a. Historical development of the polar cap potential saturation problem

The regression to the mean of extreme solar wind driver values can sometimes be confused with under-sampling bias. Under-sampling is a statistical effect entirely distinct from the regression to the mean effect. The under-sampling bias reduces with increasing samples, however, the regression to the mean remains the same even with infinite samples. It is easy to see this from the analytical derivation in the methods section (See Equation 1.21), which is immune to any sampling bias. However, under-sampling has had an important impact on the development of the polar cap saturation problem[21].

When examining space science literature on this problem, we observe from the 1980s to the 2000s (See Extended Data Figure 5a-b) that as more and more solar wind data accumulates, the polar cap potential's saturation limit movestrends upwards from ~0.5 mV/m to ~10 mV/m. As extreme values of solar wind drivers are rare, their samples in data analysis can be poor. The number of samples increases with time, and hence the bias of under-sampling also diminishes with time. As shown by Extended Data Figure 5,b, due to this bias, observations suggest different relations between solar wind driving and polar cap potential, with different saturation limits and even no saturation (i.e., Boyle et al., 1997[49]1997[51]). Since inferences from data do not agree with each other, the developers of a theory of saturation are also confounded by the question of which observational study they ought to explain. Such a diversity of inferences from data itself confuses the development of theoretical explanations. Hence, it is not surprising that multiple theories have emerged over the years (See Extended Data Table 1Extended Data) without a unifying theory explaining the saturation effect quantitatively, as there are multiple different quantitative relations between solar wind driving and polar cap potential depending on the study.

Our work, that carefully addresses uncertainties in the driver with a sufficiently large database of 25 years of measurements, brings a resolution to the contradiction between the different inferences and shows that the evidence overwhelmingly concludes that the relation between the driver and the response is linear. It is this linear relationship between the driver and response that a new (or old) physical theory or model needs to explain and validate their predictions against.

b. Evidence in Literature

Some previous data-based studies like Boyle et al. (1997)[4951] have found that solar wind driving varies linearly with cross-polar cap potential using spacecraft measurements after a meticulous filtering of data. Pulkkinen et al. 2016[50]2016[52] found that on average, the auroral electrojet index varies linearly with the shocked solar wind electric field in the magnetosheath tangential to the magnetopause (a proxy for the local reconnection electric field). Additionally, they found that this magnetosheath electric field varies non-linearly (or saturates) with the solar wind electric field. Their findings are entirely consistent with our explanation that the variance of solar wind parameters relative to the local magnetosheath values is the source of the saturation effect and not some magnetosheath phenomenon. The increased variance observable within their own data suggests that the variance in the driver leads to the saturation, and not any physical effect that causes a consistent reduction in the local magnetosheath electric field as they suggest. Borovsky 2021[30] found that the ionospheric response of PCI and SML indices varies linearly with solar wind

driving as quantified by the $S_{(1)(9b)}$ index. The $S_{(1)(9b)}$ index is developed using the method of canonical correlation analysis applied to a set of solar wind parameters and another set of geomagnetic responses. From many parameters in both sets, the method extracts a small number of canonical variate pairs that contain the majority of shared information, relegating the uncertainty (and variance) that causes a lack of correlation to an orthogonal variate pair. As we have shown, since the uncertainty leads to the saturation effect, by reducing the uncertainty in the new driver $S_{(1)(9b)}$, Borovsky 2021 implicitly removed the non-linear regression bias in the relation between $S_{(1)(9b)}$ and geomagnetic response parameters like PCI and SML index. Hence, they have a linear relationship through the entire range of the solar wind driver $S_{(1)(9b)}$, consistent with our result.

c. Regression to the mean from different vantage points

There are five vantage points from which we explain the regression to the mean effect.

Vantage point 1: Regression bias. The average value of a dependent variable given the independent variable will be biased closer to the mean value when there is some measurement uncertainty in the independent variable.

Vantage point 2: Repeat measurements. Extreme measurements are more likely followed by measurements closer to the mean. This is the most common understanding of the regression to the mean effect.

Vantage point 3: Likelihood of Stochastic Processes. Any physical process has an underlying probability distribution; therefore, some values of the physical process are more likely than others. For a stochastic process with a Gaussian distribution, the mean value coincides with the most likely or probable value. As a result, any uncertain measurement of the stochastic process is more likely measuring the true value closer to the mean of the stochastic process than more extreme values, by the simple fact that values closer to the mean are more common and hence more likely than the extreme values. In other words, the true value being measured is more likely to be closer to the mean of the stochastic process than the measurement.

Vantage point 4: Probability Theory. Figure 2a geometrically shows that for a Gaussian process, the true value is likely closer to the mean than the measurement. The black line is the probability distribution of a Gaussian process, X. When this process is measured with some uncertainty, the measurement can take the values in the likelihood quantified by the dashed-blue curve when the true value is X=2 and the dashed-red curve when it is X=4. Since the true value is generally inaccessible, the goal of a measurement is to estimate the most probable true value corresponding to the measurement. Hence, when the measurement X*=3 (solid black vertical line), the probability that the corresponding true value X is also equal to 3 (black dot) is remarkably *less likely* than it being equal to 2 (blue dot), simply because X=3 is less likely to occur than X=2 for a Gaussian process with zero mean.

Vantage point 5: Relation between truth and measurement. From vantage point 4, and consistent with other three vantage points, we can infer that regression to the mean is a property of the relation between truth and measurement, the effects of which are observable in statistical methods, and can be examined through probability theory. This new vantage point is the general result presented in this manuscript, further explanation is provided in Supplementary Discussion 1.c. The fact that regression to the mean is not only a statistical effect, but also a property of the

relation between truth and measurement, imply that the effect is present even where a single measurement is made, and hence is likely ubiquitous.

d. Regression to the mean as a fundamental property of the relation between truth and measurement

By fundamental property we mean a property that exists in reality independent of the methodology we use to study it, as opposed to an effect that arises from the methodology. For example, an undersampling bias is an effect that arises purely from the number of samples we use in our analysis. All methods that use infinite samples will never know of the undersampling bias. We argue that the regression to the mean effect, on the contrary, exists in reality as a property of the relation between truth and measurement independent of the methodology we use.

The first evidence for this comes from the fact that the phenomenon is observable in multiple statistical methods that use populations with inherent variability (e.g., different types of regression analysis, repeat measurements, machine learning, estimating conditional probability distributions), and also observable through probability theory when inferring the truth corresponding to the measurement. The second evidence is the mathematical result that can be derived using probability theory, graphically illustrated using Figure 2a, which shows that the effect only depends on the nature of the truth and measurement i.e., their likelihood of occurrence and their uncertainty. This implies that even when one makes a single measurement, the regression to the mean effect leads to a bias in the true value corresponding to the measurement according to the stochastic properties of the phenomenon being measured and the uncertainty in the measurement.

Here, an example of a familiar but different fundamental property of relations between things, e.g., the electric field, might be instructive and help us see the features of a fundamental property. An electric field is a fundamental property of the relationship between two or more charges. This relation, like all relations, has a qualitive aspect and a quantitative aspect. The quality of electric field is that it displaces the two charges relative to each other. The quantitative aspect is that the displacement depends on the nature of both the charges, its sign, magnitude, and distance between them. It was demonstrated to exist through experiments conducted by Faraday, but that does not mean it is only an experimental effect. It was proven to be mathematically consistent by Maxwell, but that does not mean it is only a mathematical effect or "field-theoretic" effect. This relationship between charges exists in reality, irrespective of the tools we use to observe it.

Similarly, when we say regression to the mean or the more-probable, is a fundamental property of the relationship between truth and measurements, it also implies, this relation, like all relations, has a qualitative and quantitative aspect. The quality of this property is that the truth is separated from the measurement by some measure. The distance of separation depends on the nature of the truth and the measurement, i.e., the stochastic properties of the truth, the degree of uncertainty of the measurement, and how it changes with time, space and magnitude of the truth. It can be demonstrated to exist through experiments with a large number of measurements (i.e., statistical methods), but that does not mean it is only a statistical effect. It can be proven to be mathematically consistent using probability theory (Figure 2a), but that does not mean it is only a

probabilistic effect. This relationship between truth and measurement exists in reality, irrespective of the tools we use to observe this relationship.

The existence of this relation between truth and measurement arises only because different from each other. Here we provide a philosophical insight into why that is the case. In our universe, truth is generally only accessible through measurements - this is because the universe is a complex system. Complex systems have many processes, that the most prominent process will have a systematic effect that we can observe, which we may call a "law", or we infer as the physical process of our interest. However, on the flip side, it will have other processes that appear as random perturbations, which may not be of our interest, and hence appear as randomness. This forces a separation between truth and measurements, leading them to be both different things. Truth is not measurement, and measurement is not the truth. However, there is an inseparable connection between them. Truth cannot be accessed without measurements, and measurements have no independent existence without the truth. This firmly establishes the existence of at least one, if not many, relationships between truth and measurement. One such relation is the regression to the mean of the truth, where the randomness manifests as uncertainty in measurements, and stochastic variations in the truth, leading to a separation of the truth from measurement in quantifiable ways.

2. Supplementary Methods

a. Validation of the Error Model

- The model predicts the pdf of the erroneous estimate of the shocked solar wind driver E_m^*. As shown in Extended Data Figure 2a, the pdf of X^*closely fits the pdf of E_m^*.

- The model predicts the standard deviation of the normalized error. A good way to visualize how the uncertainty varies with the magnitude of X^* is to calculate the standard deviation of the normalized error $\sum(X^* - \mathrm{X})/\mathrm{X}$. This quantity largely matches with its counterpart in data $\sum(E_m^* - E_{PC})/E_{PC}$ (See Extended Data Figure 2b). The fact that this match implies the shocked solar wind driver $E_m^{sh} \propto E_{PC}$, and our assumption is consistent with even the second order statistics. This also aligns with the fact that E_{PC} is constructed by maximizing the correlation with E_m^* [12], hence all the contribution to the variance comes from the difference between E_m^{sh} and E_m^*, instead of uncertainties in the measurement of E_{PC}. If the values we assume for the uncertainties dt_1, dt_2, and ε, are inadequate, the model will underestimate or overestimate the statistical variation in the difference between E_m^* and E_{PC} shown in Extended Data Figure 2b.

- Furthermore, the model predicts the conditional normalized error distribution itself. A more detailed picture of the statistical properties of E_m^* can be obtained by plotting the conditional probability density of the normalized error given X or E_{PC}. In Extended Data Figure 2d-e, the left plot is the conditional pdf of normalized error calculated from the data, and the plot on the right is the same from the model. Both have a similar structure, with the spread in error increasing up to ~12 mV/m and then decreasing. This is consistent with our assumptions of the variation of the spatial scale sizes with solar wind strength.

- Finally, the model shows that the estimates of random, unbiased uncertainties in the solar wind input (X^*) lead to a conditional bias in the estimate of the true driver X, with the bias varying with the magnitude of X^*. This bias exactly reproduces that seen in the data. In Extended Data Figure 2c, the black line is plotted from data and shows the conditional bias in the erroneous estimate E_m^* given its strength. It matches remarkably with that calculated by the model – shown in magenta. This non-linearly increasing bias is ultimately a result of the regression to the mean effect[22–24] ~~[51,52]~~[53,54].

Extended Data Figure 3 is another way to explain this effect. The left panel shows the probability distribution of the true value X. Each bin associated with X is assigned a unique color. The panel on the right shows the pdf of the error-prone estimate of X, i.e., X^*. However, the colors still preserve their correspondence to the true value. The share of color in a particular X^* bin describes the proportion of X values misidentified as X^*. In each X^* bin, a larger proportion of lower X values are misidentified as X^*. In other words, for large X^*, X is less than expected. Converting this into the terminology of observations, we get back to the statement of the problem introduced in the main paper: for large values of E_m^*, $E_{PC} \propto E_m^{sh}$ is less than expected or saturates.

b. Analytical derivation of non-linear regression bias due to uncertainty in time

Here we analytically derive the general result that temporal uncertainty in measurement can lead to a perception of non-linearity in a linear system's correlation response. For this, we first assume a linear system with an input random variable X and output response variable Y, such that

$$Y = X \qquad [1.3]$$

The input is measured or estimated with a temporal uncertainty represented by the random variable Δ. The erroneous estimate is W and is related to the true input X as

$$W = X(t + \Delta) \qquad [1.4]$$

where the statistics of $X$and Δ are known

$$X \sim f_X(x)$$
$$\Delta \sim f_\Delta(\delta)$$

Our goal here is to find the erroneous regression function $\langle Y|W\rangle = f_r^*(w)$ and see how different it is from the true regression function $\langle Y|X\rangle$. Based on equation [1.3], $\langle Y|X\rangle$ is simply $f_r(x) = x$. We also note that since the system has a simple linear response $\langle Y|W\rangle = \langle X|W\rangle = f_r^*(w)$. For the rest of the section, our goal is to derive the function $\langle X|W\rangle$ or $f_r^*(w)$.

Firstly, we note that since $W(t)$ is a stochastic process, from equation [1.4], the conditional distribution

$$f_{W|\Delta}(w|\Delta = \delta) = f_{X(t+\delta)}(w)$$

But since X is assumed to be a stationary process,

$$f_X(w) = f_{X(t+\delta)}(w)$$
$$\Rightarrow f_{W|\Delta}(w|\Delta = \delta) = f_X(w) \qquad [1.5]$$

However,

$$f_W(w) = \int f_{W,\Delta}(w,\delta)d\delta$$
$$= \int f_{W|\Delta}(w|\Delta = \delta)f_\Delta(\delta)d\delta \qquad [1.6]$$

And from equation [1.6],

$$f_W(w) = \int f_X(w)f_\Delta(\delta)d\delta$$

As we assume X and Δ are independent, and $\int f_\Delta(\delta)d\delta = 1$, hence

$$f_W(w) = f_X(w) \qquad [1.7]$$

That is, **the marginal distribution of W and X has the same functional form**.

From equations [1.5] and [1.7], it follows that W **and Δ are also independent**.

$$\begin{aligned} f_{W,\Delta}(w,\delta) &= f_{W|\Delta}(w|\delta) f_{\Delta}(\delta) \\ &= f_X(w) f_{\Delta}(\delta) \\ &= f_W(w) f_{\Delta}(\delta) \end{aligned} \quad [1.8]$$

Now we estimate the joint probability distribution function of W, X, and Δ.

$$f_{W,X,\Delta}(w,x,\delta) = f_{W|X,\Delta}(w|x,\delta) f_{X,\Delta}(x,\delta)$$

Since $X$and $\Delta$are independent,

$$f_{W,X,\Delta}(w,x,\delta) = f_{W|X,\Delta}(w|x,\delta) f_X(x) f_{\Delta}(\delta) \quad [1.9]$$

By integrating the above over the range of Δ, we can calculate the marginal pdf of W and X.

$$f_{W,X}(w,x) = \int f_{W|X,\Delta}(w|x,\delta) f_X(x) f_{\Delta}(\delta) d\delta \quad [1.10]$$

Finally, we can calculate the conditional pdf of X given W.

$$f_{X|W}(x|w) = \frac{f_{W,X}(w,x)}{f_W(w)}$$

From equation [1.7], we see that

$$f_{X|W}(x|w) = \frac{f_{W,X}(w,x)}{f_X(w)}$$

Using this and equation [1.10], we get

$$f_{X|W}(x|w) = \int f_{W|X,\Delta}(w|x,\delta) \frac{f_X(x)}{f_X(w)} f_{\Delta}(\delta) d\delta \quad [1.11]$$

Hence from the definition of expectations we get,

$$\langle X|W \rangle = f_r^*(w) = \int x f_{X|W}(x|W=w) dx \quad [1.12]$$

To solve equation [1.12], we need to assume a functional form for the pdf of X and Δ. $f_X(x)$ is assumed to be lognormal distribution, and $f_{\Delta}(\delta)$ to be a normal distribution. A lognormal random variable X has a corresponding normally-distributed random variable Z such that

$$\begin{gathered} Z \sim \phi(z, \mu_Z, \sigma_Z) \\ Z = \log X \\ \mu_Z = \log\left(\frac{\mu_X^2}{\sqrt{\mu_X^2 + \sigma_X^2}}\right) \\ \sigma_Z = \sqrt{\log\left(1 + \frac{\sigma_X^2}{\mu_X^2}\right)} \end{gathered} \quad [1.13]$$

Here, the function ϕ is the general normal distribution.

$$\phi(x,\mu,\sigma) = \frac{1}{\sqrt{2\pi}\sigma} e^{-(x-\mu)^2 / 2\sigma^2} \qquad [1.14]$$

Therefore,

$$f_X(x) = \frac{1}{x}\phi(\log x, \mu_Z, \sigma_Z) \qquad [1.15]$$

Additionally, as $X(t)$ is a stochastic process, the random variable at each time instance correlates to some degree with adjacent time instances. We define this using an autocorrelation function of the following form

$$\rho_X(\delta,k) = e^{-\delta / k} \qquad [1.16]$$

Here $\delta = |t_2 - t_1|$ is the absolute difference in time between two random variables, $X(t_2)$ and $X(t_1)$. For our case, this is also the error in measurement time corresponding to $\Delta \sim f_\Delta(\delta)$. $\boldsymbol{k}$ **is the autocorrelation time constant**.

The normally distributed uncertainty in time is defined as

$$f_\Delta(\delta) = \phi(\delta, 0, \sigma_\delta) \qquad [1.17]$$

Here σ_δ **is a measure of the random uncertainty in the time of measurement** Δ.

From equations [1.13] and [1.16], we can estimate the autocorrelation function corresponding to the normal random variable Z

$$\rho_Z(\delta,k) = \frac{1}{\sigma_Z^2}\log\left(1 + \rho_X(\delta,k)\left[e^{\sigma_Z^2} - 1\right]\right) \qquad [1.18]$$

From the results of the bivariate lognormal distribution, we can derive the functional form of $f_{W|X,\Delta}(w|x,\delta)$ to be the following

$$f_{W|X,\Delta}(w|x,\delta) = \frac{1}{w}\phi\left(\log w, \mu_Z + \rho_Z(\delta,k)[\log x - \mu_Z], \sigma_Z\sqrt{1-\rho_Z(\delta,k)^2}\right) \qquad [1.19]$$

From equations [1.11],[1.12],[1.15],[1.17], and [1.19], we can calculate the regression function$\langle X|W\rangle = f_r^*(w)$.

$$f_r^*(w) = \int_0^\infty \int_{-\infty}^\infty \phi\left(\log w, \mu_Z + \rho_Z(\delta,k)[\log x - \mu_Z], \sigma_Z\sqrt{1-\rho_Z(\delta,k)^2}\right) \frac{\phi(\log x, \mu_Z, \sigma_Z)}{\phi(\log w, \mu_Z, \sigma_Z)}\phi(\delta, 0, \sigma_\delta)\, d\delta dx$$

[1.20]

By integrating the above integral within the ranges of x, and substituting $\frac{\delta}{k} \to u$, we can show that the above integral is a function of the ratio $\frac{\sigma_\delta}{k}$. This ratio as a measure of uncertainty in the time of measurement.

$$f_r^*\left(w,\frac{\sigma_\delta}{k}\right) = \int_{-\infty}^{\infty} w^{\rho_Z(u)} e^{\frac{1}{2}[1-\rho_Z(u)][2m_Z+(1+\rho_Z(u))\sigma_Z^2]} \frac{1}{\sqrt{2\pi}\,(\sigma_\delta/k)} e^{-\frac{u^2}{2(\sigma_\delta/k)^2}} du \qquad where\ u=\delta/k \qquad [1.21]$$

We numerically integrate the above integral within the ranges of Δ for specific values of σ_δ/k and generate Figure 3c. The pdf of X is similar to that assumed in the previous section, with the mean $\mu_X = 1.12$ and standard deviation $\sigma_X = 1.15$. The figure shows that $f_r^*(w,\sigma_\delta/k)$ varies non-linearly with w when the temporal uncertainty ratio is larger than 0%, compared to $f_r(x) = x$ (which is linear) when $W = X$ or the temporal uncertainty ratio is 0%. Therefore, we have found that the regression function $\langle Y|W\rangle = f_r^*(w,\sigma_\delta/k)$ is non-linear with respect to w for values of $\sigma_\delta/k > 0$. Finally, the numerical integration of the analytical solution described by [1.21] was compared with an independent Monte Carlo solution of the error model stated in equation [1.4], with the results being almost identical, confirming that our derivation is correct.

c. Summary of data analysis procedure in the computer code

The primary computer code (Code 1) constructs a Monte-Carlo simulation to solve a non-linear error model to show that the polar cap index saturation observed in data is a result of the regression to mean effect stemming from random error in the shocked solar wind driver estimates.

Step 1: Preparing and loading observations data. First 1-minute resolution WIND spacecraft data already time-shifted to bow-shock nose from the OMNI database, Polar Cap Index (PCI) data, and the magnitude uncertainty (in the form of standard deviation) varying with shocked solar wind driver (derived in Code 3) is loaded. All missing values, and out-of-range values, are converted into a 'nan' value, PCI is calculated from PCN and PCS as described in the Methods section. The Kan-Lee electric field at L1 (E_m^*) is calculated from WIND spacecraft measurements of plasma and field parameters. Finally, all values of E_m^* and PCI that are not simultaneous are removed from the analysis. The difference between E_m^* and PCI from data is also calculated as a measure of the total error or difference between the driver and the response. Finally, the autocorrelation function of E_m^*, PCI, and $E_m^* -$ PCI is calculated.

Step 2: Constructing model input. We compare the pdf of E_m^* and PCI, create a log-normal pdf that best fits the PCI index values, which has mean 1.114 and standard deviation 1.14 and, in the log-space, has mean -0.2518 and standard deviation 0.85. We fit a spline-function on the autocorrelation values and compare the autocorrelation functions of E_m^*, PCI, and $E_m^* -$ PCI. From these values derived from measurements, we create the first input to the model: a stochastic process X which is the hypothetical true stochastic process (i.e., shocked solar wind driver value), with also an autocorrelation functions that is similar to that observed in data. For this we use an external function by Lienhard (2021) to generate multivariate lognormal random numbers with correlation. Then we construct random variables representing time uncertainties: dt1 and dt2 with

distributions and parameters derived from previous literature and discussed in the Methods section. Next we construct a stochastic process $\varepsilon(t)$ representing the magnitude uncertainty, which has zero mean, and a variance varying with the magnitude of X according to the uncertainty derived directly from simultaneous magnetosheath and solar wind measurements using Code 3. $\varepsilon(t)$ also has an autocorrelation function with values similar to that derived from the autocorrelation function of $E_m^* -$ PCI.

Step 3: Solving the Monte-Carlo Error Model. By plugging in the above inputs of X, dt_1, dt_2 and ε, into the following error model $X^* = X\,(t + dt_1 + dt_2) + \varepsilon$, we estimate the stochastic process X^*. These stochastic processes are represented by an ensemble of 16000 realizations, with each realization consisting of 2^{12} time samples (i.e., ~4096 minutes). This can be increased to improve the statistics at the very low probability tail region of the log-normal distribution (i.e., for solar wind driver values > $15\ mV/m^2$). The resulting value of X^* is the solution of the error model. From this we calculate the conditional expectation of $\langle X|X^*\rangle$, and notice that there is an appearance of a saturation effect on the average shocked solar wind driver given solar wind driver values measured at L1. This is then compared with the conditional expectation of $\langle PCI|E_m^*\rangle$, and we notice that the curves have a good correspondence, suggesting the regression to the mean effect is the cause for the saturation of the polar cap index with solar wind driver value measured at L1.

Step 3: Validating the Monte-Carlo Error Model. We verify the reliability of the error model by comparing many second-order statistics, as well as full probability distribution and conditional probability distributions with that of their counter parts in measurements. We list here the different statistics from the model that have been compared to show good correspondence with their counterparts in data: probability density function of input - $pdf(X)$, probability density function of output - $pdf(X^*)$, the autocorrelation function of the input - $\langle X(t)X(t+\tau)\rangle$, autocorrelation function of the output - $\langle X^*(t)X^*(t+\tau)\rangle$, normalized marginal error distribution - $pdf\left(\frac{X^*-X}{X}\right)$, the normalized marginal error distribution conditioned on X - $pdf\left(\frac{X^*-X}{X}|X\right)$, the normalized marginal error distribution conditioned on X^* - $pdf\left(\frac{X^*-X}{X}|X^*\right)$, standard deviation of normalized error - $\sigma(X^*-X)/X$, mean of error - $\langle X^*-X\rangle$ and finally the conditional $pdf(X|X^*)$.

Step 4: Saturation effect in another geomagnetic response parameter (SML). We load the westward auroral electrojet index (SML) from SuperMag database, remove all the data gaps, and select only the data points that are simultaneous with the time-shifted Kan-lee electric field measured from L1. We then assume a counter part for SML in the model, $Y_{SML} = -120X + \epsilon_Y$, where ϵ_Y is a Gaussian-distributed random error in the estimate of the SML value with zero mean and $\sigma_{\epsilon_Y} = 5$ units. When the conditional expectation of Y_{SML} given X^*, $\langle Y_{SML}|X^*\rangle$, is calculated we observe that it also saturates very similar to the saturation of SML index with solar wind driver values.

Step 5: Regression calibration: Correcting for the regression to the mean effect. The bias caused by the regression to the mean effect is quantified by $b = X^* - \langle X|X^* \rangle$, and this can be removed from X^* to estimate the corrected solar wind driver function $X^c = X^* - b = \langle X|X^* \rangle$. This is carried out in the code by a one-dimensional linear interpolation of the solar wind driver value measured at L1 and time-shifted to the bow shock (E_m^*) with a look up table consisting of sample points $x_i \in [0,25]$ and its corresponding values $f(x_i) = \langle X|X^* = x_i \rangle$ for each query point E_m^*. Therefore, the result is $E_m^c = f_r(E_m^*)$. We now plot the conditional expectation $\langle PCI|E_m^c \rangle$ and $\langle SML|E_m^c \rangle$ all derived directly from data. They reveal a linear relation of PCI and SML with the corrected solar wind driver function (or the true shocked solar wind driver value). We refer interested readers to the following literature for further discussion on the distributions of solar wind parameters, and geomagnetic response measures[21,22,30,55,56].

Code 2 integrates the analytical formula in Eq [1.21] to solve for the regression bias stemming from uncertainty in the time of the measurement of a stochastic process. The code outputs a sensitivity analysis of the regression bias for different values of the ratio of time uncertainty to the autocorrelation time constant. Lastly, we validate the analytical solution independently; by developing an error model of only time uncertainty and solving it using the Monte Carlo method. The sensitivity analysis from the analytical result and the Monte Carlo solution match very well, confirming our analytical derivation is correct.

Code 3 uses simultaneous magnetosheath satellite measurements and measurements of spacecraft at L1 from the OMNI database, to calculate the variance of the L1 solar wind driver estimates for a given value of the shocked solar wind driver close to magnetopause subsolar point. The resulting variance is the magnitude uncertainty, and a spline fit of the variation of the magnitude uncertainty with increasing value of the shocked solar wind driver is used in Code 1 to reconstruct the magnitude uncertainty ε and its heteroskedastic variation with the magnitude of X in the error model in Code 3.

Code 4 uses probability theory to show that the true value corresponding to an uncertain measurement of a Gaussian random process is biased towards the mean. This is a demonstration of the regression to the mean effect through the lens of probability theory.

EXTENDED DATA

Extended Data ~~Fig.~~Figure 1~~.~~| Stochastic properties of inputs to the error model. a, The black line shows the pdf of the polar cap index E_{PC} from 1995 to 2019. And the ~~magenta~~purple line shows the lognormal pdf of the model random variable X that best fits the pdf of E_{PC}.

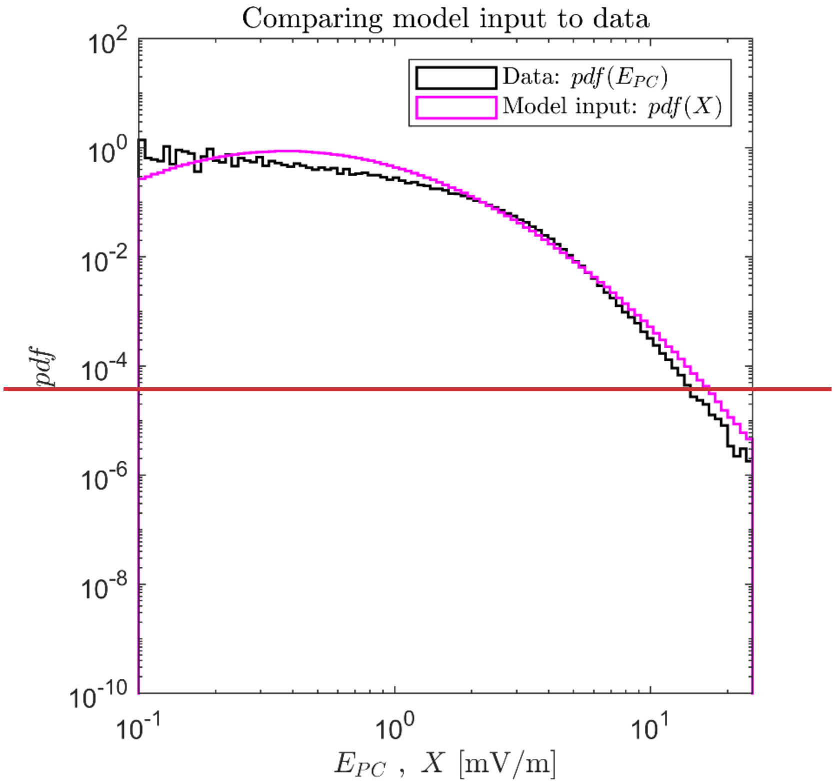

~~Extended Data Fig. 2.~~ b, The black dashed line shows the autocorrelation function of the polar cap index E_{PC} averaged over data from 1995 to 2019. And the ~~magenta~~purple line shows the autocorrelation function of the model random variable X that closely follows the ACF of E_{PC}.

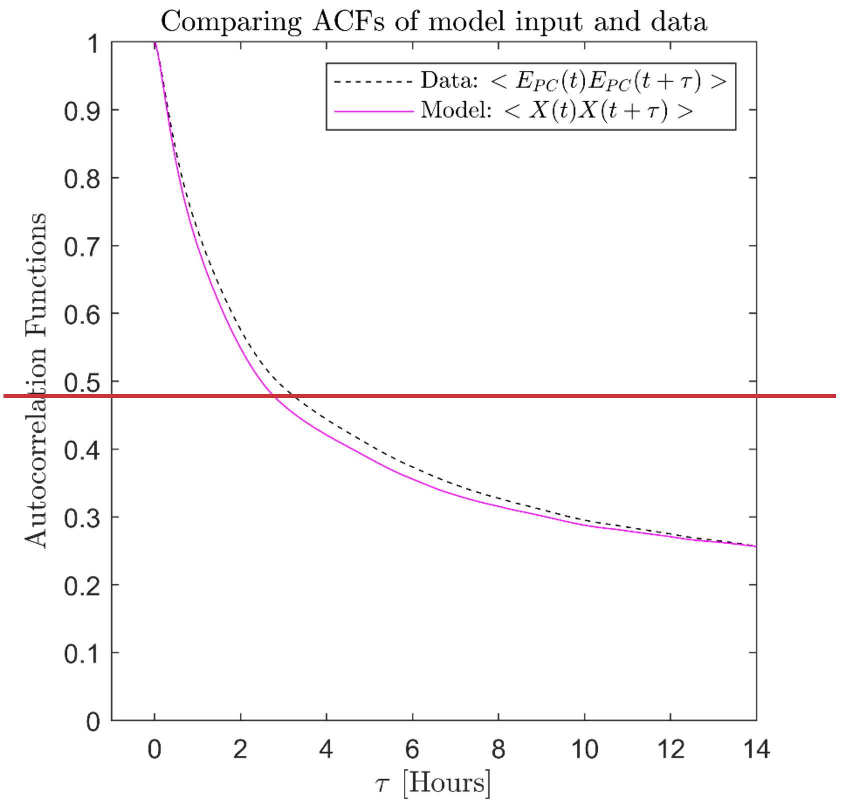

~~Extended Data Fig. 3.~~ c, The black line shows the uncertainty in the propagation delay from L1 to the bow shock nose(dt_1). The blue line shows the uncertainty in the propagation delay from the bow-shock nose to the polar cap ionosphere(dt_2). The magenta line shows the total uncertainty in the propagation delay from L1 to the polar cap($dt_1 + dt_2$).

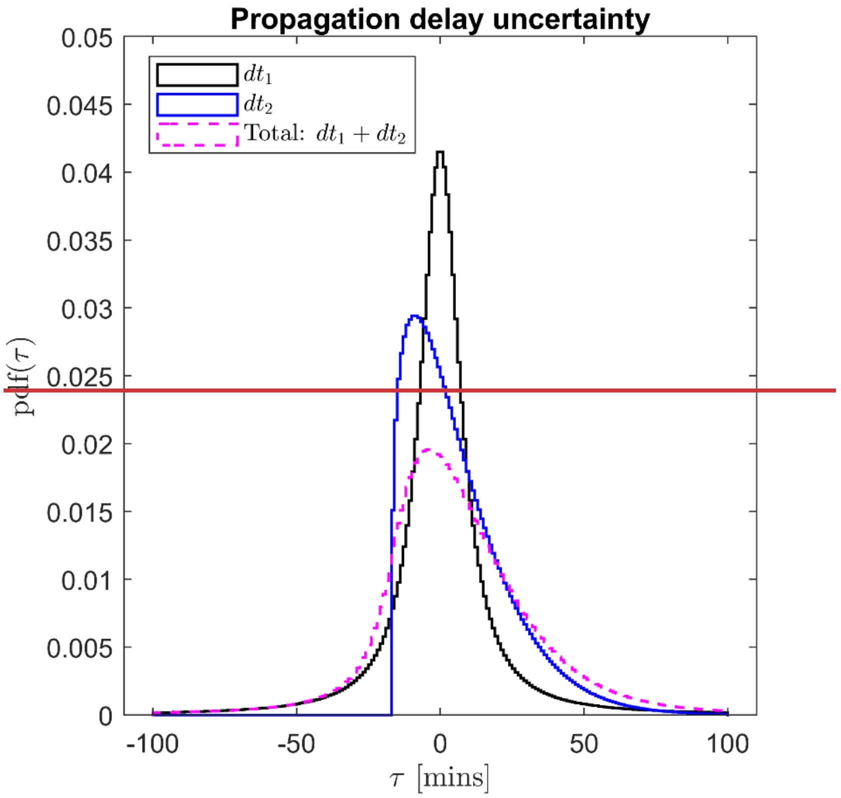

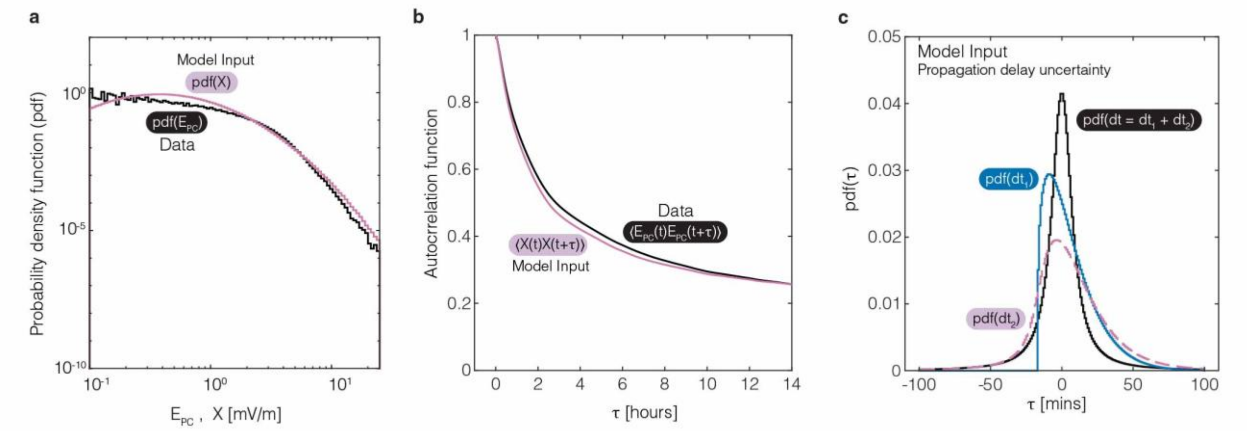

Extended Data ~~Fig. 4.~~Figure 2| Statistics of the output of the error model. a, The model predicts with good correspondence the pdf of the estimate of the merging electric field propagated to the polar cap, ~~almost exactly~~with a probability density difference of less than ~0.1 between data and model for most values. E_m^* is data, and X^* is model.

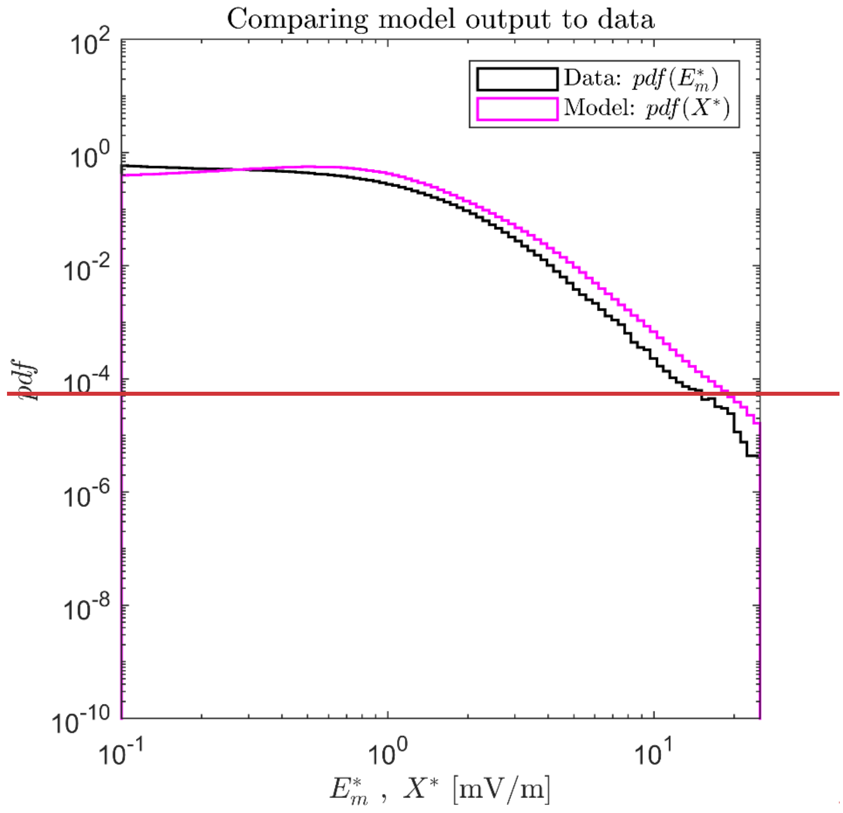

~~Extended Data Fig. 5.~~ b, The model predicts the nature of the random error between the estimate and the true value of the merging electric field projected on the ionosphere. The black line is the normalized uncertainty associated with E_m^* (data), and the purple line is the normalized uncertainty associated with X^* (model). c, There is a non-linear conditional bias in E_m^*: $\langle E_m^* - E_{PC} | E_{PC} \rangle$ that varies with E_{PC}, which is reproduced very well by the model $\langle X^* - \mathrm{X} | \mathrm{X} \rangle$.

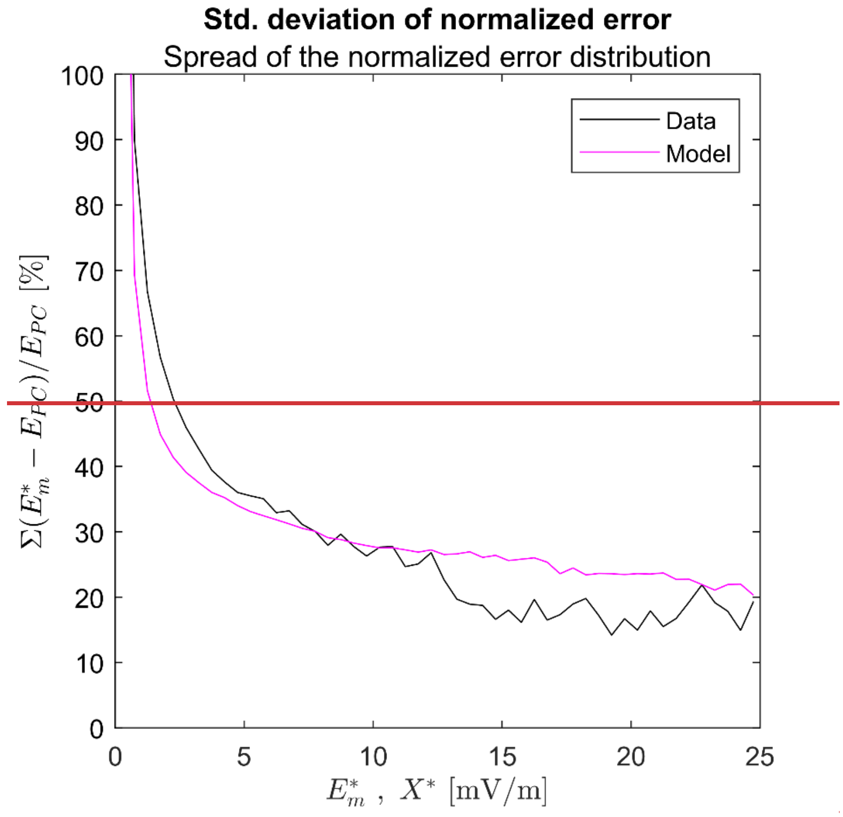

~~**Extended Data Fig. 6.**~~ ~~(Left)~~ **d,** Conditional normalized error distribution from data, ~~(Right)~~**e,** Conditional normalized error distribution from the model. Both data and model show similar trends in how the error varies with increasing strength of the true variable –a steep increase in the spread of the error from 0 to ~12 mV/m in the vertical axis, and then a decrease (or loss of power) beyond that point.

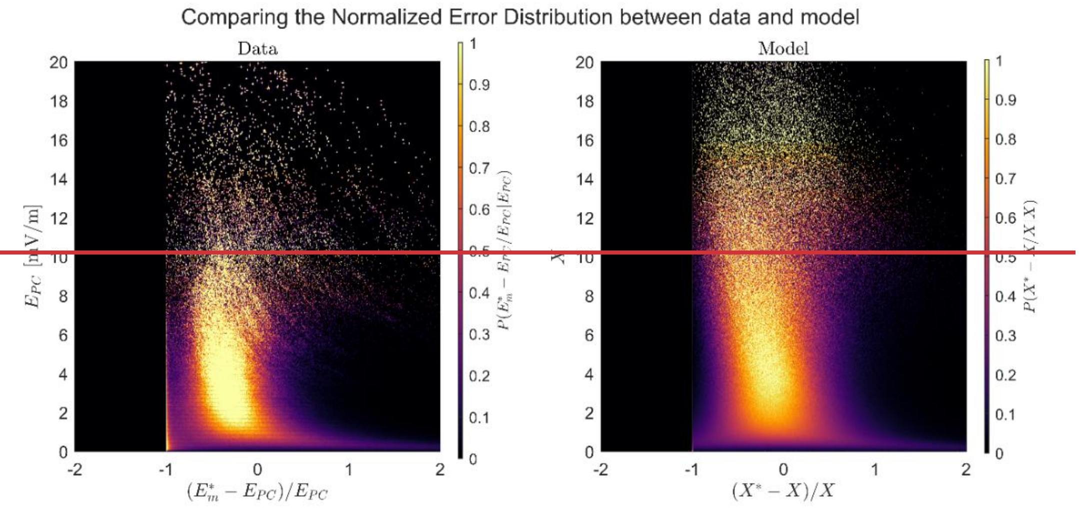
Comparing the Normalized Error Distribution between data and model
Data
Model
E_{PC} [mV/m]
$(E^*_m - E_{PC})/E_{PC}$
$P(E^*_m - E_{PC}/E_{PC}|E_{PC})$
$(X^* - X)/X$

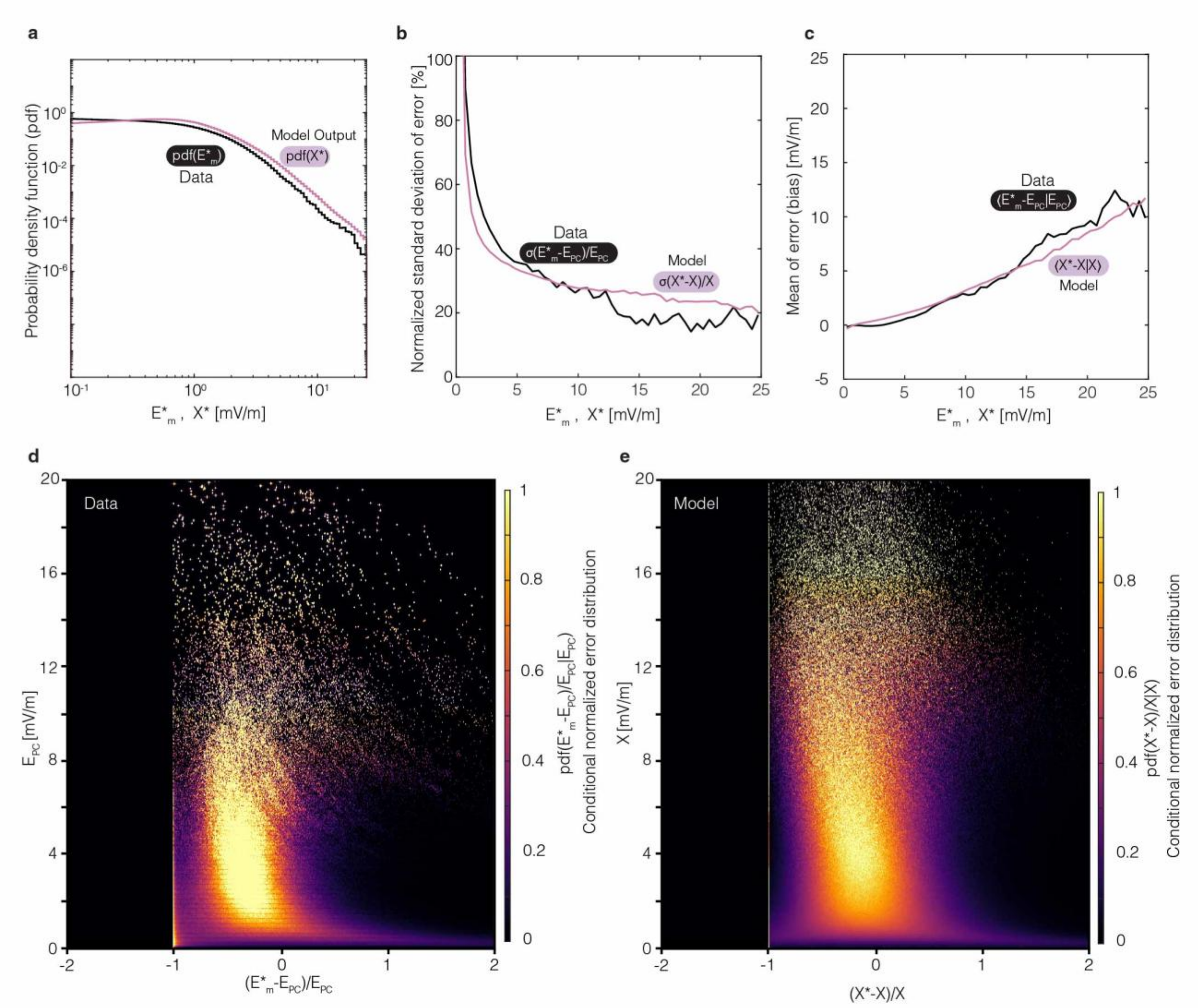
a
b
c
Probability density function (pdf)
Model Output
pdf(X*)
pdf(E*m)
Data
E*m , X* [mV/m]
Normalized standard deviation of error [%]
Data
σ(E*m-EPC)/EPC
Model
σ(X*-X)/X
Mean of error (bias) [mV/m]
Data
(E*m-EPC|EPC)
(X*-X|X)
Model
d
e
Data
Model
EPC [mV/m]
X [mV/m]
pdf(E*m -EPC)/EPC|EPC)
Conditional normalized error distribution
pdf(X*-X)/X|X)
(E*m-EPC)/EPC
(X*-X)/X

Extended Data Figure 3| Another graphical explanation of the regression to the mean effect.

~~Extended Data~~ Fig. 7. ~~There is a non-linear conditional bias in E_m^*: $\langle E_m^* - E_{PC} | E_{PC} \rangle$ that varies with E_{PC}, which is reproduced very well by the model $\langle X^* - X | X \rangle$.~~

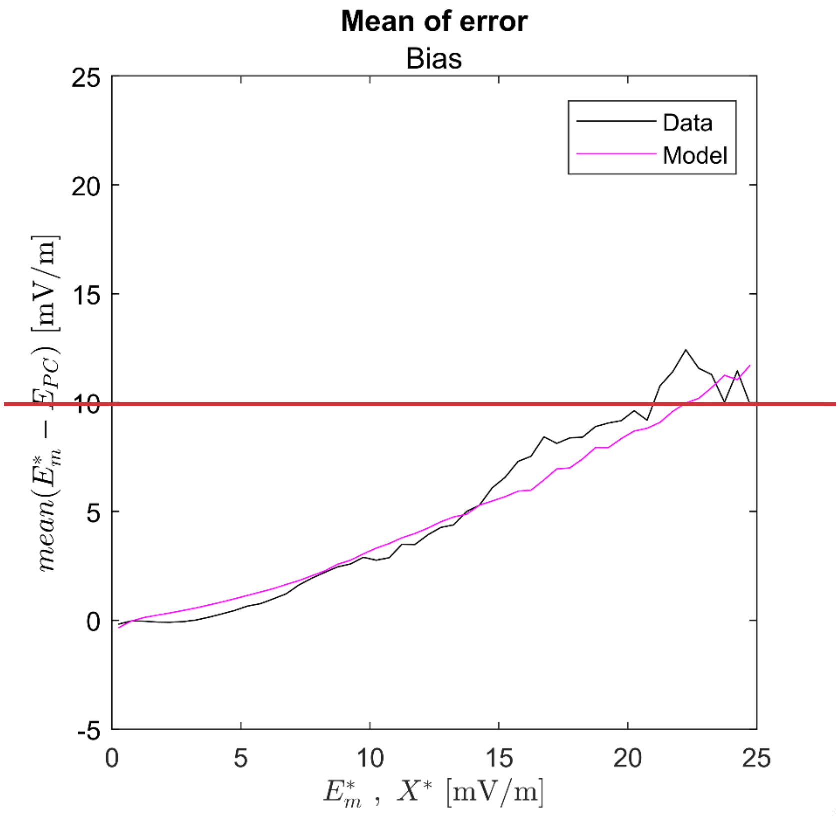

~~Extended Data Fig. 8.~~ (Left) Probability distribution function of the true variable X, with each bin colored uniquely. (Right) The probability distribution function of the inaccurate estimate of X, i.e., X^* with the color representing the magnitude of X. The amount of a particular color per bin represents the proportion of X values misidentified as X^*.

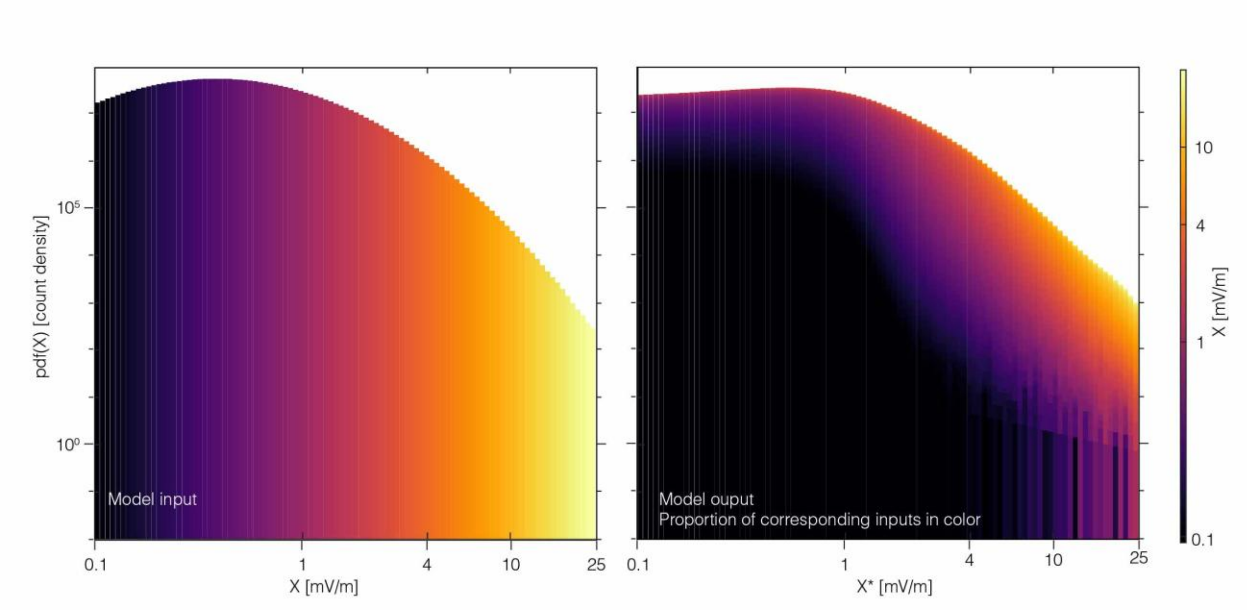

Extended Data

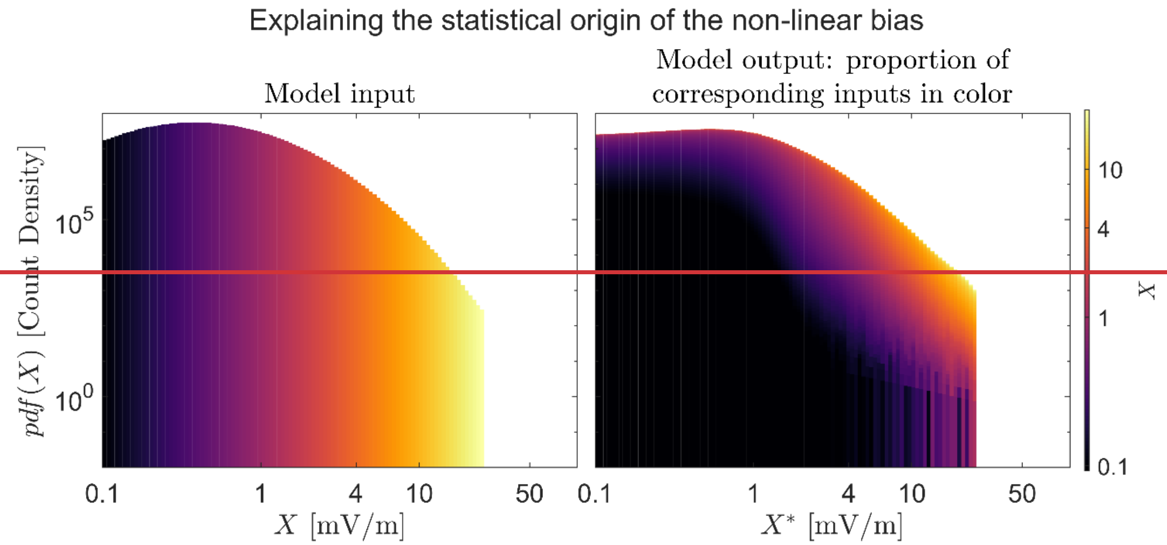

Figure 4| Sensitivity analysis of magnitude uncertainty. a, Non-linear regression bias caused by heteroskedastic magnitude uncertainty increases with a measure of the uncertainty represented by the minimum relative magnitude uncertainty. **b,** The heteroskedastic variation of the magnitude uncertainty with the true value X is shown, for different values of the minimum relative magnitude uncertainty corresponding to the different regression bias curves in panel a.

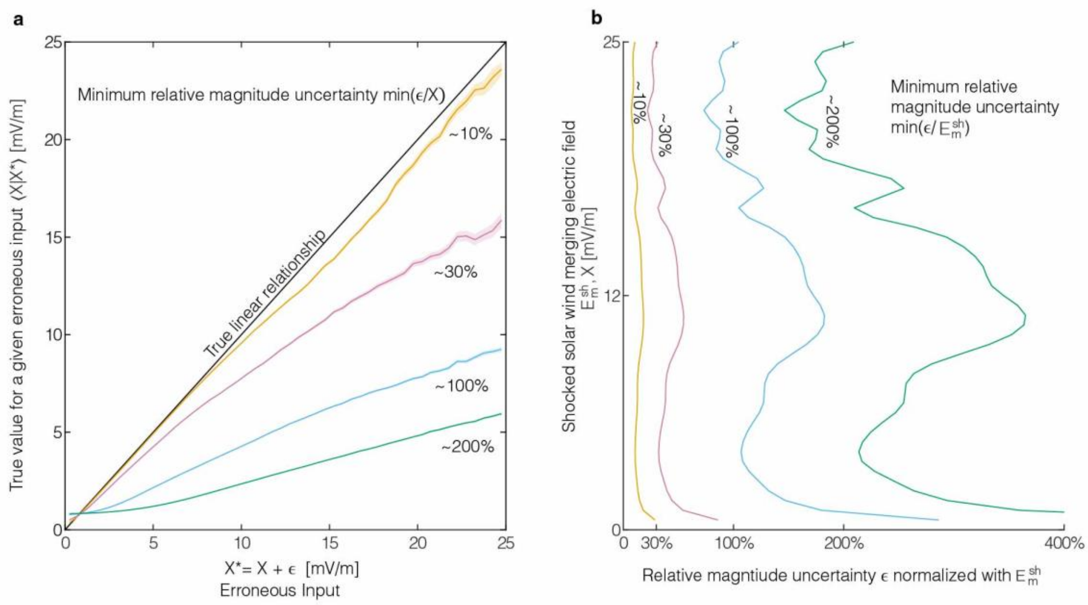

Extended Data Figure 5| Increasing trend of the saturation limits observed by previous observational studies. a,

~~**Extended Data Table 1.** Ten models explaining cross polar cap potential saturation. Table is reproduced from Borovsky et al., 2008[15] with additions.~~

~~Extended Data Table 2.~~ Cross polar cap potential response to solar wind driving is linear up to a saturation limit, which varies depending on the observational study and the methodology they use. All except Boyle et al., 1997 ~~[49]~~[51] infer a saturation beyond the limit in E_m^*. See the studies here: 1 [57], 2 [58], 3 [59], 4 [51], 5 [60], 6 [61], 7 [62], 8 [63], 9 [64]. **b,** The saturation limit of different studies follow an increasing trend (linear regression) with the year of study, with the latest studies including higher number of data samples.

a

Study	Saturation limit in E_m^* [mV/m]	Instruments
1) Reiff et al., 1981	~ 3	LEO Satellites: AE-C, AE-D, S3-3
2) Wygant et al., 1983	~ 0.5	LEO Satellites: S3-3
3) Weimer et al., 1990	~ 4	Magnetometers, AE Index
4) Boyle et al., 1997	~ 8 (found no saturation)	LEO Satellites: DMSP
5) Russell et al., 2001	~ 3	AMIE
6) Liemohn & Ridley, 2002	~ 10	AMIE
7) Shepherd et al., 2002	~ 3	SuperDARN radars
8) Nagatsuma, 2002	~ 5	Magnetometer PC Index
9) Hairston et al., 2005	~ 10	LEO Satellites: DMSP

b

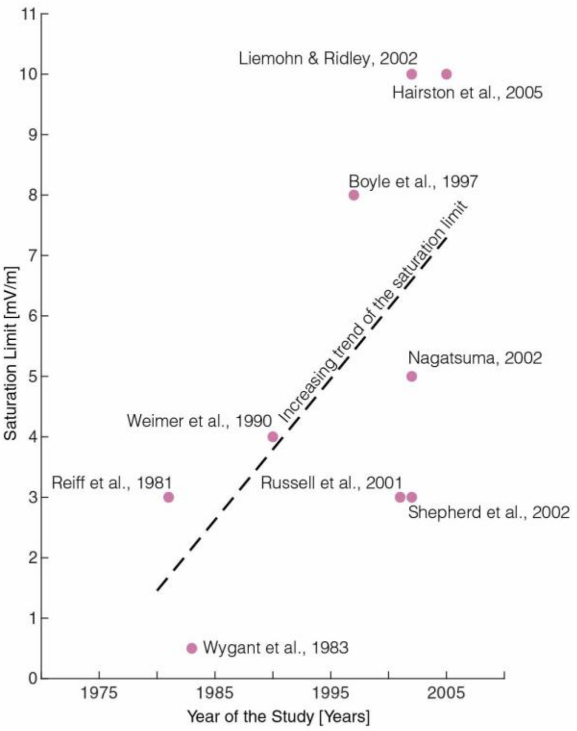

Extended Data Table 1| Theories of polar cap potential saturation. Ten models explaining cross polar cap potential saturation. See the following works for more details about each of the models: 1[65,66], 2[67,68], 3[69,70], 4[71], 5[70,72], 6[73], 7[74], 8[75], 9[76], 10[77,78] and an assessment of most of them[15]. Adapted with permission from [Joseph E. Borovsky, Benoit Lavraud, Maria M. Kuznetsova, Table A1, Polar cap potential saturation, dayside reconnection, and changes to the magnetosphere., Journal of Geophysical Research: Space Science, Publisher: John Wiley and Sons] Copyright 2009 by American Geophysical Union.

Theory/Model	Description
1) Hill model	Reduced dayside magnetosheath field lowers the reconnection rate
2) Flaring model	Flaring lowers the reconnection rate
3) X-line length model	Magnetosphere size changes decreases total reconnection
4) Force-balance model	Magnetosheath flow divergence reduces geoeffective length
5) Magnetosheath flow model	Solar wind conditions alter dayside reconnection rate
6) Lobe-bulge model	Bulging lobes impedes dayside reconnection
7) Bow shock model	Polar cap currents alter bow shock and dayside reconnection rate
8) Ionospheric outflow model	Ionospheric outflow mass-loads magnetospheric convection
9) Ram pressure-saturation model	Dynamic pressure constraints currents
10) MHD generator model	Alfven conductance of the shocked solar wind plasma limits currents

Extended Data Table 2| Common symbols used in this work. Variables representing measurements and their corresponding counter parts in the error model used commonly through the paper are summarized here.

Symbols	Description	Symbols	Description
E_m^*	Solar wind driver, Kan-Lee electric field, merging electric field, measured at L1 Lagrange point and time-shifted to bow-shock	X^*	Measurement, Model counter part of solar wind driver measured at L1 Lagrange point and time-shifted to bow-shock
E_m^{sh}	Shocked solar wind driver measured in the magnetosheath close to the subsolar point near the magnetopause	X	True value, Model counter part of shocked solar wind driver close to the subsolar point in the magnetosheath
E_{sw}	Solar wind convection electric field	ϵ	Zero-mean random error, magnitude uncertainty
E_{PC}	Dawn-dusk convection electric field across the polar cap	Y_{PC}	Model counterpart of dawn-dusk convection electric field across the polar cap, also counter part of PCI
E_m^c	Calibrated solar wind driver	X^c	Calibrated solar wind driver
E_R	Reconnection electric field	b	Regression bias (due to regression to the mean effect)
PCI	Polar cap index, also a proxy for E_{PC}	Y	True value, model counter part of geomagnetic response
PCN	Polar cap index derived from a magnetometer in northern polar cap	Y^*	Erroneous measurement of Y
PCS	Polar cap index derived from a magnetometer in the southern polar cap	dt	Uncertainty in the time of measurement
SML	Westward auroral electrojet index derived from SuperMAG network of ground magnetometers	dt_1	Uncertainty in solar wind propagation delay from L1 to bow shock nose
$B_{T,sw}$	Transverse magnitude of solar wind magnetic field	dt_2	Uncertainty in propagation of the effect of solar wind from bow shock to the polar cap ionosphere
B_z	Magnetic field along the Z-axis of the GSM coordinates	σ_δ	Standard deviation of random uncertainty in time of measurement
B_{sh}	Magnetic field value in the magnetosheath	k	Autocorrelation time constant
V_{sh}	Plasma velocity measured in the magnetosheath	V_{sw}	Solar wind plasma velocity
θ'	Shear angle between the magnetic field vector in the magnetosheath and the magnetosphere at the magnetopause	θ_{sw}	Solar wind magnetic field clock angle

Title: Regression to the Mean of Extreme Geomagnetic Storms

Authors: Nithin Sivadas[1,2*], David Sibeck[2], Varsha Subramanyan[3], Maria-Theresia Walach[4], Dogacan Su Ozturk[5], Banafsheh Ferdousi[6], Bayane Michotte de Welle[2]

Affiliations:

[1]Department of Physics, The Catholic University of America, Washington, D.C., USA

[2] NASA Goddard Space Flight Center, Greenbelt, Maryland, USA

[3]Department of Physics, University of Illinois Urbana-Champaign, USA

[4]Department of Physics, Lancaster University, United Kingdom

[5]Department of Physics, Geophysical Institute, University of Alaska, Fairbanks, AK, USA

[6]Air Force Research Laboratory, Albuquerque, NM, USA

*Corresponding author. Email: nithinsivadas90@gmail.com

Abstract: Extreme space weather events on Earth occur during intervals of strong solar wind driving. The solar wind drives plasma convection and currents in the near-Earth space environment. For low values of the driver, the Earth's response is linear, estimated by parameters such as the polar cap index based on ground magnetometer activity. Curiously, for extreme solar wind driving, the Earth's response appears not to increase beyond a saturation limit. Theorists have advanced a host of explanations for this saturation effect, but there is no consensus. Here, we demonstrate that the saturation is a manifestation of the *regression to the mean* effect arising from random uncertainty in the timing and magnitude of solar wind measurements. Our results reveal that data analysis underpinning the saturation theories is non-linearly biased, thereby challenging the validity of the theories. Correcting for the uncertainties reveals that the Earth's response to solar wind driving is linear throughout, and that the impact of extreme geomagnetic storms can be twice as large as previously thought. We show that regression to the mean is a fundamental property of the relationship between measurement and the truth, where the truth corresponding to the measurement is closer to the mean. This effect is particularly pronounced for uncertain measurements of extreme values and is likely to manifest across various fields, from extreme climate studies to chronic medical pain.

One-Sentence Summary: Extreme geomagnetic storms may have twice the impact on Earth than previously thought.

MAIN TEXT

~~**1. Saturation of Geomagnetic Activity**~~

From assessing the stability of buildings during major earthquakes to estimating the effects of hazardous radiation on biological organisms, a fundamental challenge in science is understanding how systems behave under rare and severe conditions. However, a critical, often-overlooked factor complicates this pursuit: uncertainty in measuring the conditions. In this paper, we demonstrate quantitatively that random error can systematically distort our inferences of the system's response, especially for extreme events. We leverage this crucial insight to resolve a long-standing puzzle in space physics—the apparent saturation of geomagnetic activity during extreme space weather[1]. We encourage readers to read this paper's extensive supplementary sections in the order they are referenced in the text.

~~Space physicists have long studied the effects of solar wind on the dynamics of the plasma and electromagnetic fields around the Earth. In particular,~~**Saturation of Geomagnetic Activity.** Extreme geomagnetic storms with strong solar wind driving can increase the electric current strengths in the near-Earth space environment[2,3], causing nationwide power outages, disruptions in global satellite communication networks, and increased ozone loss in the polar ionosphere [4,5]. The ~~The Earth's magnetic field carves out a cavity in the oncoming solar wind, called the magnetosphere. It mostly keeps out the plasma and magnetic field associated with the solar wind except under specific local magnetic field conditions that lead to the reconnection of solar wind and magnetospheric magnetic field lines. Upstream from the reconnection site, the supersonic and superalfvénic~~ solar wind slows down through a ~~bowshock, forming the magnetosheath just~~bow-shock upstream ~~from the magnetosphere's outer boundary – the magnetopause. When the shocked solar wind magnetic fields lie anti-parallel to those of the Earth, the field can undergo a topological change that opens up the Earth's magnetic field lines and reconnects them to the shocked solar wind magnetic field lines[6]. Through reconnection, the solar wind plasma and electric field can enter the Earth's magnetosphere and drive~~ of the Earth's magnetosphere and drives plasma convection in the ~~magnetosphere. Conveyed to the~~ polar cap ionosphere ~~closer to the Earth, this convection electric field applies an electric potential across the polar cap on average[7]~~ (See Figure 1a, and Supplementary Discussion 1.a~~).~~). The ~~projection of the solar wind convection electric field along the dawn-dusk direction in the polar cap is the Kan-Lee or merging electric field (E_m^*)[8], which we refer to as solar wind driving[9]~~electric field ~~.~~

~~In the 1970s, estimates of the cross-polar cap potential (and its corresponding dawn-dusk electric field) were made from single ground magnetometers situated in the northern and southern polar regions. The argument was that the perturbations of the magnetic field measured by the polar magnetometers give an excellent continuous estimate of the solar wind driving, as the polar regions magnetically connect to the solar wind[10]. Continuous estimates of solar wind driving from satellite missions were not yet available at the time[11]. As the magnetized plasma in the solar wind and magnetosphere is collisionless, the resistance along magnetic field lines is minuscule. Hence, the magnetohydrodynamic theory is valid, ensuring that plasma along an entire field-line moves as one[6]. Therefore, the solar wind electric field,~~ resulting from the motion of the wind~~, at one end of~~ enters the magnetosphere at the day-side boundary through magnetic ~~field line, would map along the field line to the polar ionosphere. This~~reconnection. Magnetohydrodynamic theory implies that this solar wind electric field ~~then~~ drives currents in the ionosphere that cause magnetic

perturbations measured from ground magnetometers. Supporting this theory, early observational studies showed that the cross polar cap index (PCI), a measure of geomagnetic activity, increases linearly on average with the merging electric field (E_m^*), a measure of the solar wind driving[12]driving[6]. The PCI is estimated by a polar magnetometer from the ground in each hemisphere and is proportional to the cross polar cap potential[13,14]potential[7,8], while E_m^* is measured from a spacecraft in the solar wind far upstream of the Earth. As more measurements of rare and extreme solar wind driving accumulated through the years, surprisingly, the correlation between the geomagnetic response and the solar wind driver began to deviate from the linear relation. there appeared to be an upper limit to the cross-polar cap potential beyond which increasing solar wind driving led to no increase in the potential (See Figure 1b green curve).

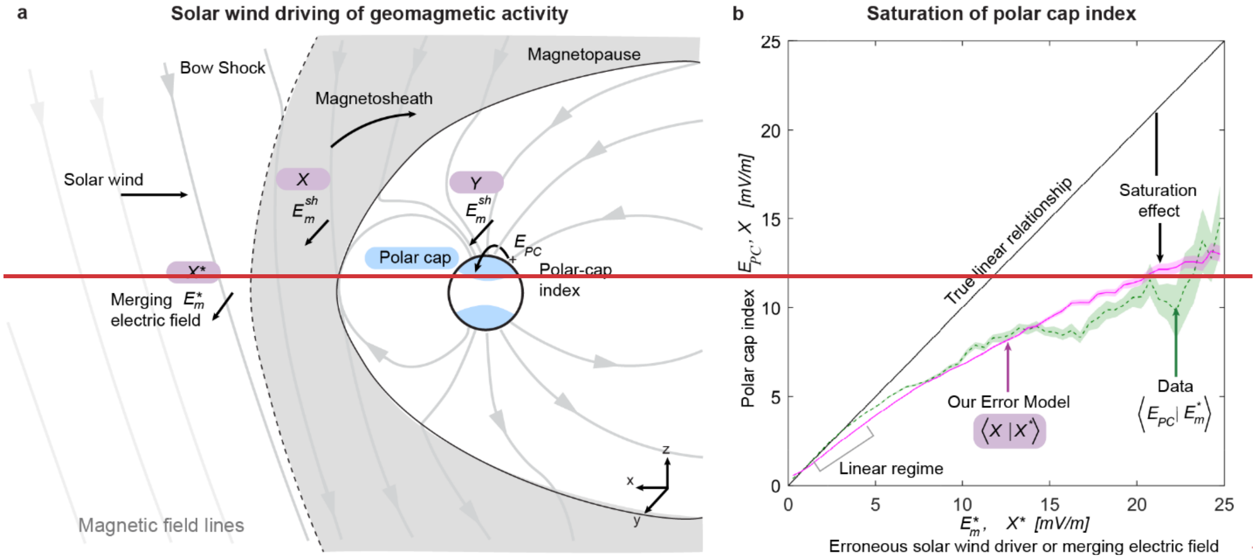

*This**Figure 1* ***Saturation of geomagnetic activity with solar wind driving.*** *a) Solar wind merging electric field (E_m^*) measured upstream of the bow-shock, is transformed to the shocked solar wind driver in the magnetosheath (E_m^{sh}) downstream of the bowshock, which corresponds to the dawn-dusk electric field that maps to the polar ionosphere (E_{PC}); b) Observations from 1995-2019, green curve, shows that on average the cross polar cap index (∝ the dawn-dusk electric field E_{PC}) increases linearly at low values of solar wind driving, but deviates from linearity and saturates at large values of driving. The magenta curve shows that the error model developed in this work predicts the same saturation effect due to uncertainty in the solar wind driver instead of any physical mechanism. The transparent shaded area for both curves shows a 95% confidence interval.*

The new inference that the cross polar cap potential saturates with increasing solar wind driving from later measurements led to the emergence of 10 different but sometimes overlapping theories and models attempting to explain the phenomenon (See Extended Data)[15,16]. The theories fall into two categories, one arguing that the conditions at the solar wind/magnetosphere interaction region (i.e., the dayside magnetosheath) during extreme solar wind driving lead to a consistent diminishing of the rate of reconnection or energy coupling from the solar wind to the magnetosphere. The other set of theories argues that processes within the magnetosphere slow down ionospheric plasma convection during extreme solar wind driving. All theories suggest that the energy transferred into the polar ionosphere during extreme space weather has, in essence, an upper limit. Hence, the theories imply that the Earth's magnetosphere shields us from extreme geomagnetic storms by inhibiting energy transfer from the strong solar wind to the ionosphere, one way or the other.

~~In this paper, we present a radically different proposition. We argue that there is no statistical evidence for the saturation of geomagnetic response to strong solar wind driving, and its appearance in measurements is a result of uncertainty in solar wind driver measurements. We correct for the effect of this uncertainty and demonstrate that the geomagnetic response varies linearly with solar wind driving, implying that the energy transfer from extreme solar wind conditions to the polar ionosphere can be far greater than currently believed. The importance of this work goes beyond space science to any correlation study between a measure of a system's input and its response, revealing a larger response for extreme input values.~~

saturation phenomenon (See Extended Data Table 1)[9,10]. However, here we argue that there is no statistical evidence for the saturation of geomagnetic response to strong solar wind driving, and its appearance in measurements is a result of uncertainty in solar wind driver measurements.

Uncertainty and the Problem of Definition. The solar wind measurements are made mainly far upstream from the day-side reconnection site. They are, hence, an uncertain estimate of the actual solar wind driver close to the reconnection site in the magnetosheath, transformed during its journey, and influenced by local plasma and field conditions. Uncertainties in measurements are commonly attributed to instrumental error, but a surprising fact is that uncertainties can also result from the assumptions that we make while inferring data from measurements. This uncertainty stems from a "problem of definition"[11] (e.g., Supplementary Discussion 1.b). ~~A carpenter might measure the height of a door frame using a measuring tape. However, the door she creates using this height will likely not fit the frame. Even though the least count of the tape is small and does not contribute much to the uncertainty, there is uncertainty due to an implicit assumption that the height of the door is a one-dimensional quantity when it is in reality at least a two-dimensional quantity, i.e., there are multiple different heights for the door and the door frame, varying across its breadth and width. Hence, the experienced carpenter would use the carpenter's square to ensure that the edges of the door are perpendicular to the length, to match the door frame, and reduce this uncertainty. This uncertainty stems from a "problem of definition"[17].~~

~~A similar~~This problem of definition arises when using solar wind measurements made at the Lagrange point L1, ~230 R_E upstream of the reconnection site, as an estimate for the local magnetosheath conditions that drive the coupling of energy and plasma into the magnetosphere. The shocked solar wind driving (E_m^{sh}) near the reconnection site differs from what is measured at ~~L1[18,19]~~L1 [12,13] due to 1) variability in propagation times from the L1 point to the Earth, 2) spatial variability of the solar wind ~~structure[20]~~structure[14], and 3) evolution of solar wind along that path due to a variety of plasma processes changing the essential parameters influencing the reconnection rate, such as the local magnetic field direction.

Uncertainties ~~arising from instrument error or the problem of definition~~ can be of two types: bias or unbiased random error. A bias is a consistent deviation of the true value from the measurement itself. Physical theories that explain relations between physical parameters or their respective measurements almost always attempt to explain consistent and deterministic deviations between them~~. In fact, they are disinterested in~~, excluding the random fluctuations in their values. On the other hand, the unbiased random error frequently consists of these random fluctuations and is the random deviation of the measurements from the true value. Physical theories in space science generally explain average changes and not random fluctuations. Hence, an unknown bias can be misinterpreted as a physical effect (i.e., an average change) when a theory is constructed to explain the bias. In short, physical theories ~~are in the business of explaining~~intend to explain *physical*

biases, and if we ignore bias stemming from random measurement error, we may mistake it for a physical phenomenon.

Regression to the mean and the more-probable. A common goal of scientific analysis is to uncover the relations between two or more physical processes. Random errors in the measurement of these processes pose a difficulty in inferring relations between them, for example, in our case, the relation between solar wind driver and the geomagnetic response. However, it is widely believed that averaging the measurements can remove the effects of random error, as the underestimates will cancel out the overestimates. In Supplementary Discussion 1.c~~Here we will~~, we show that this widespread belief is only partially true.

A popular technique to find the relations between two or more physical processes is regression analysis of their measurements. Here the uncertainty in the dependent variable is averaged away, but the uncertainty in the independent (or conditional) variable manifests as bias in the relation inferred between the variables, known as *regression bias*[15]. If the regression bias is non-linear, it can create an appearance of saturation of the dependent variable for increasing values of the independent variable. This statistical phenomenon is a result of a *regression to the mean*[16,17].~~A popular technique to find the relations between two or more physical processes is regression analysis of their measurements. The essence of regression analysis is to find the average of the measurement Y^* when the measurement X^* is a specific value 'x'. This is equivalent to the conditional expectation of Y^* given $X^* = x$, mathematically represented as $\langle Y^*|X^* = x\rangle$. Here, Y^* is the dependent variable and X^* is the independent or conditional variable. The averaging of Y^* indeed removes the effect of random error in Y^*, consistent with the widely held belief. But, crucially, the conditional or independent variable X^* cannot be averaged while simultaneously averaging the dependent variable Y^* for a given value of X^*. In other words, the random error in the conditional variable remains unaddressed and is not averaged away[21]. And, surprisingly, this unbiased random error in the conditional variable (X^*) manifests as a bias in the relation inferred between the variables Y^* and X^*. This *regression bias* is such that the average value of Y^* for a given X^* will be closer to the mean of Y, and if the regression bias is non-linear, it can create an appearance of saturation in Y^* for increasing X^*. This statistical phenomenon is a result of a *regression to the mean*[22,23].~~ In literature, regression to the mean is commonly understood as a statistical phenomenon where extreme measurements are more likely followed by measurements closer to the ~~mean[23]~~mean[17]. However, ~~here~~ we argue that the phenomenon has a more fundamental origin.

The regression to the mean is not only a statistical phenomenon, though it commonly appears as one. It is not a result of a particular statistical method of inferring relations between parameters, although the particular method will be affected by it. At its core, regression to the mean is a property of the relation between the true value of a stochastic process and its measurement. We explain why this property is a fundamental one in Supplementary Discussion 1.d.

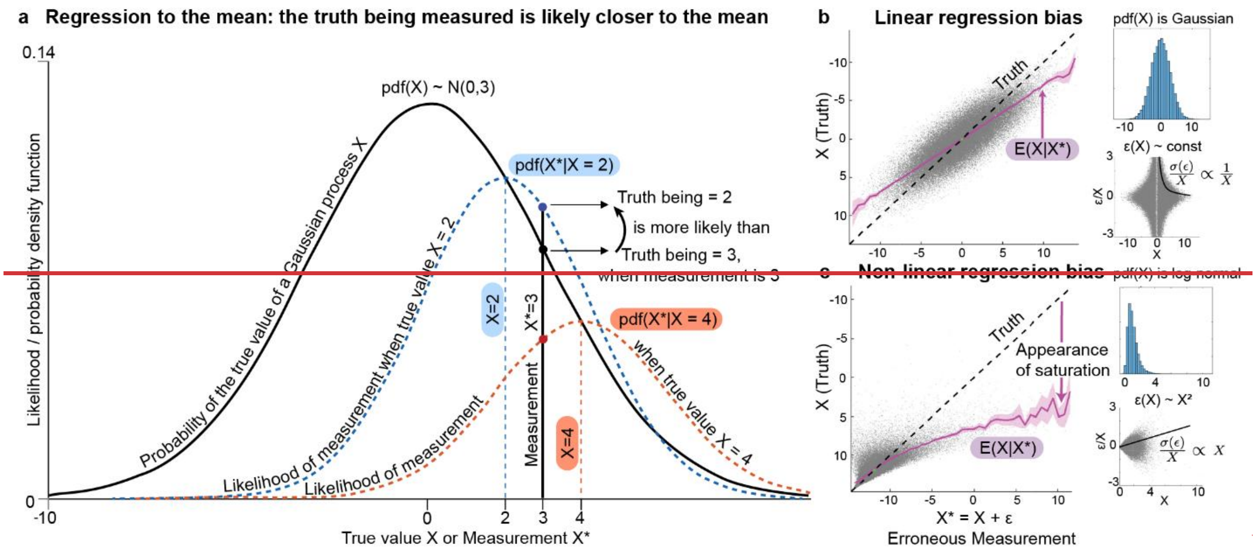

<u>In Supplementary Discussion</u> 1.e~~Figure 2 **Explaining regression to the mean effect using probability theory and regression bias.** a) For a physical parameter X which has an underlying probability distribution, some values are more likely than others, and hence when the measurement is X*=3 the corresponding true value is more likely to be X = 2 than X = 3; b) The expected true value of a Gaussian process X has a linear bias when measurement is X* = X + ϵ with a constant random error (ϵ) ; c) The expected true value of a log-normal process X has a non-linear bias when the measurement X* has random error (ϵ(X)) that varies with the strength of X. This figure is adapted from Sivadas & Sibeck 2022[22], used under CC BY 4.0. The transparent shaded area for regression curves shows a 95% confidence interval.~~

~~Any physical process has an underlying probability distribution; therefore, some values of the physical process are more likely than others. For a stochastic process with a Gaussian distribution, the mean value coincides with the most likely or probable value. As a result, any uncertain measurement of the stochastic process is more likely measuring the true value closer to the mean of the stochastic process than more extreme values, by the simple fact that values closer to the mean are more common and hence more likely than the extreme values. In other words, the true value being measured is more likely to be closer to the mean of the stochastic process than the measurement.~~

~~a demonstrates this result geometrically, where the black line is the probability distribution of a Gaussian process, X. When this process is measured with some uncertainty, the measurement can take the values in the likelihood quantified by the dashed-blue curve when the true value is X=2 and the dashed-red curve when it is X=4. Since the true value is generally inaccessible, the goal of a measurement is to estimate the most probable true value corresponding to the measurement. Hence, when the measurement X*=3 (solid black vertical line), the probability that the corresponding true value X is also equal to 3 (black dot) is remarkably *less likely* than it being equal to 2 (blue dot), simply because X=3 is less likely to occur than X=2 for a Gaussian process with zero mean.~~

~~This is the regression to the mean laid bare; the truth being measured regresses towards the mean, and this property of the relation between the truth and measurement manifests in many different forms depending on what one uses the measurements for. In Supplementary Discussion~~, we explain the regression to the mean effect from five different yet consistent vantage points. For instance, ~~as discussed above,~~ it manifests <u>commonly</u> as 1) a regression bias in the relation inferred between two ~~measurements[22], and~~<u>measurements[16],</u> 2) a statistical effect where extreme measurements are

more likely followed by measurements closer to the mean[17]. Most importantly, it is also 3) a probabilistic effect where the true value being measured is more likely to be closer to the mean of the stochastic process than the measurement (Figure 2~~mean[23]. Crucially, the~~a demonstrates this geometrically).

The essence of regression to the mean is ~~not just a statistical effect as widely believed, but~~that the truth being measured regresses towards the mean, and this property of the relation between the truth and measurement manifests in many different forms depending on what one uses the measurements for. It is a fundamental logical consequence of the ~~relationship~~relation between the true value and its measurement~~. This result implies~~, implying that for even a single measurement, the corresponding true value is likely closer to the mean of the stochastic process. For a stochastic process with a general probability distribution, the effect is more aptly named the 'regression to the more-probable'. ~~However, in this paper, we maintain the use of the more popular phrase 'regression to the mean' even when the stochastic process is non-Gaussian.~~

Solar wind uncertainty and error model. Due to regression to the mean, when an extreme value of the solar wind driver is measured at the L1 Lagrange point, ~~it is more likely that~~ the true ~~value of the solar wind~~ driver value near the Earth is more likely smaller and closer to the mean. Therefore, the geomagnetic response to this smaller driver will also be smaller. ~~And~~ When we do not account for this regression to the mean, as in most previous studies, the smaller geomagnetic response will be wrongly associated with the extreme value of the solar wind driver measured at L1. The more extreme the value of the measured solar wind driver, the greater the mismatch between the true driver and the measured value. As a result, the cross-polar cap potential response to the measured uncertain solar wind driver deviates from linearity during the rare and extreme values and hence, appears to saturate.

To quantitatively test this surprising qualitative argument, we construct an error model $X^* = X(t + dt) + \epsilon$, where X^* is the measured 'uncertain' solar wind driver at L1, and X is the 'true' driving by the shocked solar wind close to the reconnection site. dt is the uncertainty in the propagation time of the solar wind from L1 to the bow-shock, and the propagation time of electromagnetic disturbance from the bow-shock through to the polar ionosphere. ϵ is the random magnitude ~~changes~~change in the solar wind driver due to spatial and temporal variability influenced by processes during the evolution of the solar wind from L1 and through the bow-shock, and the magnetosheath.

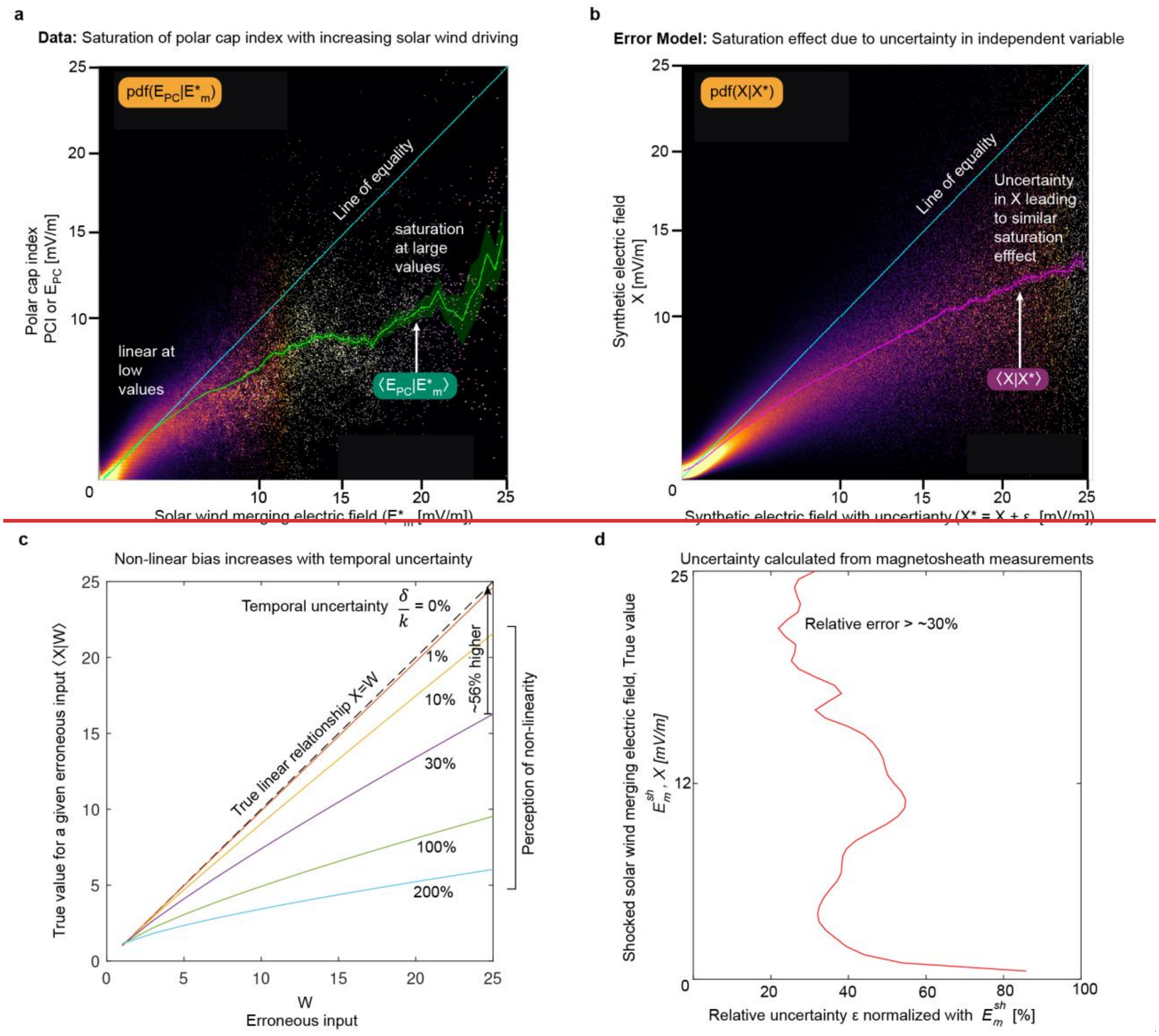

~~*Figure 3* ***Regression bias predicted by the error model matches saturation effect from data.*** *a) Conditional probability density function of 25 years of measurements $PCI|E_m^* \sim E_{PC}|E_m^*$; b) Conditional probability density function reproduced by the error model $X|X^*$ which is very similar to the measurements presented in panel a. The transparent shaded area for both regression curves shows a 95% confidence interval; c) Non-linear bias caused by uncertainty in time increases with a measure of the uncertainty represented by the dimensionless ratio of the time uncertainty (δ) and the autocorrelation constant (k); d) Quantified relative uncertainty $\epsilon(X)/X$ due to random variations in solar wind driver measured at L1 (E_m^*, X^*) for a measured value of the shocked solar wind driver (E_m^{sh}, X).*~~

~~As shown by Sivadas & Sibeck, 2022[22] using a simple Monte Carlo error model, if X has a Gaussian distribution, and the error ϵ is also Gaussian and independent of X like most instrumental errors (i.e., homoskedastic error[24]), then the likely true value for a given measurement (i.e., the average of X given X^* or $\langle X|X^*\rangle$) will have a linear bias. The purple curve in Figure 2b shows this linear bias as a lower slope than the dashed-black line of equality representing the curve if there were no uncertainty (i.e., the true unbiased relationship). However, suppose the error ϵ depends on X, and varies with the magnitude of X (i.e., heteroskedastic error[24]) like some uncertainties related to the 'problem of definition', and X has a log-normal distribution like many solar wind parameters[25]. In that case, the key point is that the likely true value for a given measurement will~~

have a larger and non-linear bias that mimics a *saturation* of X with increasing X^*. Figure 2c demonstrates this in the purple curve whose slope has a non-linear bias with respect to the black-dashed line of equality representing the true unbiased relationship. Supposing that X is the true solar wind driver near the Earth, and X^* is the uncertain measurement far from Earth, the response Y of the Earth will be driven by the true driver X and, crucially, not by the measurement X^*. If the response Y is linearly related to X (i.e., $Y \propto X$), then naturally, the response Y will also appear to saturate with increasing value of the measurement X^*.

Furthermore, if we keep aside the magnitude uncertainty (i.e., set $\epsilon = 0$), and examine the effect of uncertainty in time dt, we observe that it will lead to a non-linear bias in the average of X given X^* as well, with increasing deviation from linearity with growing uncertainty in time defined by the ratio of the temporal uncertainty (δ) and the autocorrelation time constant (k) of the stochastic process. Figure 3c shows quantitatively how the average of the true value X given the measurement X^* has a non-linear bias in its slope with respect to the dashed-black line of equality representing the true unbiased relationship. This non-linear bias increases with increasing ratio of δ/k, a dimensionless measure of the uncertainty in time. In the [26], we derive analytically from first principles this non-linear bias and its dependence on temporal uncertainty, which will be relevant for any study with measurement of systems that have a delay in their drivers or response, due to propagation of information, or dynamics of the system e.g., seismic waves from earthquakes[27], or response of medical patients to a specific back pain treatment[28].

For our case of solar wind driving of the geomagnetic system, We make quantitative estimates of the temporal uncertainty dt from previous work and the magnitude uncertainty $\epsilon(X)$ from simultaneous measurements made close to the dayside reconnection site. The calculation of the uncertainty is detailed in the Methods section[18]. Figure 3d presents the relative uncertainty ($\epsilon(X)/X$) calculated from measurements with respect to the true shocked solar wind driver in the magnetosheath (X), and it is at least 30% or greater. This uncertainty is a heteroskedastic error as it varies with the magnitude of the true value (X) [19].

As shown by Sivadas & Sibeck, 2022[16] using a simple Monte-Carlo error model, when the error is heteroskedastic like some uncertainties related to the ‘problem of definition’, and X has a log-normal distribution like many solar wind parameters[20], the likely true value for a given measurement will have a non-linear bias that mimics a *saturation* of X with increasing X^*(Figure 2c). Figure 3c shows such a non-linear bias can also increase with increasing ratio of δ/k, a dimensionless measure of uncertainty in time (See Supplementary Discussion 1.fOnce these uncertainty values are worked into the error model, and X is assumed). As a result, once the calculated uncertainty values are worked into the error model, and X is set to have a log-normal distribution with an autocorrelation coefficient of ~100 minutes observed in solar wind measurements, the Monte-Carlo error model solves for X^* and predicts an appearance of saturation of X given X^* (See Figure 3b). This saturated curve, and the conditional probability distribution of X given X^*, matches surprisingly well with the saturation that appears in the data for the polar cap index PCI given the merging electric field estimated from solar wind measurements E_m^* (See Figure 3a). The solution of the error model is consistent with data as viewed through both the conditional expectation $\langle PCI|E_m^*\rangle$ and the conditional probability distribution of $pdf(PCI|E_m^*)$ (See a-b). Further validation of the error model with second order statistics of data is presented in the [26]. Here PCI is analogous to Y in the error model, E_m^* is analogous to X^*, and E_m^{sh} analogous to X. The similarity in the curves $\langle PCI|E_m^*\rangle$ and $\langle X|X^*\rangle$, indicate that the geomagnetic response is

~~proportional to the true shocked solar wind driver i.e., $PCI \propto E_m^{sh}$ and equivalently $Y \propto X$.~~ The precondition of this non-linear saturation relation between PCI and E_m^* is the heteroskedastic nature of the error in E_m^*, the propagation time uncertainty, and the fact that the solar wind driver is a log-normal process with a particular autocorrelation time constant (See Supplementary Discussion 1.g~~.~~).

Saturation of the cross-polar cap potential explained. This surprising result from the error model shows that uncertainty in the solar wind driver leads to a biased inference that the geomagnetic response saturates with solar wind driving. ~~This~~The uncertainty therefore creates an illusion of saturation when we compare measurements of the solar wind driver to the cross-polar cap index, which is proportional to the cross-polar cap ~~potential[13]. This implies~~potential[7]. Hence, there is currently no statistical evidence to suggest an upper limit to the energy transferred from the solar wind to the polar ionosphere. This suggests that the ten prevailing theories and models of polar cap potential saturation were developed to explain a biased inference from existing erroneous measurements. As a result, these theories have not been tested or validated against corrected and unbiased data.

We maintain that the original assumption —that the solar wind magnetic field lines connect directly to the polar regions, driving ionospheric convection, and therefore are on average linearly related to the magnetic fluctuations in ground-magnetometers and thus the polar cap index —holds ~~true[29]. The saturation effect is, surprisingly, an illusion resulting from random error in our measurements of the solar wind driver. This implies that there is currently no evidence to suggest the existence of an upper limit to the energy transferred from the solar wind to the polar ionosphere. In the following section~~ true[21]. We show below that correcting for the effect of uncertainties ~~reveals a~~reveal this linear relationship ~~between the solar wind driver and geomagnetic response~~, implying we cannot rely on the magnetosphere to dampen the effects of extreme geomagnetic storms.

Correcting the effect of uncertainties. There are two ways to address the effect of uncertainties. One approach is to reduce uncertainty in our measurements or estimates of the solar wind driver. For instance, by relying on spacecraft measurements closer to Earth to estimate the driver. Uncertainties are bound to exist in every measurement, and the problem of definition will almost always exist, so the method of reducing uncertainties is unlikely to eliminate them. Hence, alternatively, we ~~can~~ statistically quantify the uncertainties, predict their effect, and offset them from our inference. This method is achievable, albeit challenging, through scientific investigation of the relations between the truth and measurement, and the assumptions that underlie our inferences of the measurements. ~~Here we implement this method of quantifying and correcting the uncertainties.~~

As shown ~~in the previous two sections~~, once we calculate uncertainties and develop an error model, ~~it is possible to calculate~~we can derive the non-linear bias caused by the independent variable in the regression analysis. The bias is the consistent deviation between the measurement and the likely true value estimated from the error model $b = X^* - \langle X|X^* \rangle$. Therefore, we can subtract this bias from the erroneous independent variable, and one method to do this is ~~termed~~ “regression calibration”~~[24,26]~~[18,19]. The calibrated variable X^c is simply $X^* - b$. ~~Once we apply~~By correcting this bias ~~correction to~~of the solar wind driver~~, the relation between the corrected driver~~ ($E_m^c = E_m^* - b$~~) and~~), its relation with the cross-polar cap index ~~is~~becomes surprisingly linear up to 15 mV/m. Figure 4a shows the saturating green curve formed by using the erroneous data transforms to the linear purple curve after applying this regression calibration. This ~~linearity between E_m^c and PCI~~

is observed after correctly applying the regression calibration operation, which uses as input only $\langle X|X^*\rangle$ from the error model. Hence the linear relationship between E_m^c and PCI was discovered without any assumptions of linearity or non-linearity in the relation between $Y E_m^{sh}$ and X or PCI, and E_m^{sh} their model counterparts. Therefore, regression calibration of the solar wind measurements demonstrates that the true geomagnetic response is linear, and that the saturation of the geomagnetic response is a result of uncertainty in the solar wind driver and the regression to the mean effect. Note that beyond 15 mV/m, the data available is not significant enough to conclude the shape of the relation between the driver and response. When the same calibration is applied to other measures of geomagnetic activity, such as the westward auroral electrojet (See Figure 4b and Supplementary Discussion 1.h), its relation with the driver becomes linear, providing further evidence that geomagnetic response to solar wind driving does not saturate.

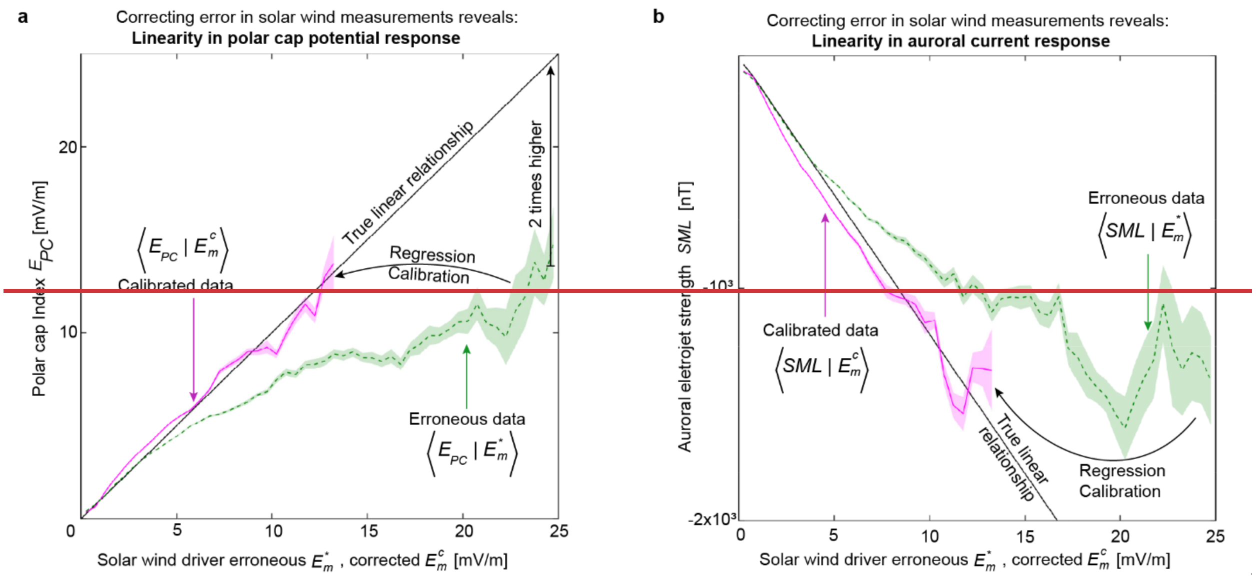

Figure 4 ***Correcting the effect of random errors in solar wind driver values reveals a linear geomagnetic response.*** *a) Polar cap index varies linearly with solar wind driving on average (purple curve), after correcting the regression bias in the erroneous solar wind driver values (green curve); b) The westward auroral electrojet strength also varies linearly with solar wind driving (E_m^c) on average (purple curve) after the same regression bias correction applied to the erroneous solar wind driver values (E_m^*) (green curve). The transparent shaded area for all the curves shows a 95% confidence interval.*

Our finding that uncertainties in the solar wind driver results in the saturation of the polar cap index, raises the possibility that other measures of the geomagnetic response previously reported to saturate (or not)[30] based on uncertain solar wind driver estimates may be incorrect. The SML index, which is the westward auroral electrojet strength, measured by completely different magnetometers located at high latitudes ($\sim 65^\circ N$) around the planet, also appears to saturate with erroneous solar wind driving estimates. However, the corrected solar wind driver values, calibrated exactly the same way as above, show a linear relationship with the westward auroral electrojet strength as well—a further confirmation that the high-latitude geomagnetic response does not saturate with solar wind driving, once we remove the effect of uncertainties. b shows the linear relation of the SML index with corrected solar wind driver in the purple curve, once the regression calibration is applied to the erroneous data in the saturating green curve. It is this linear relationship between the driver and response that a new (or old) physical theory of saturation needs to explain and validate its predictions against. In fact, the development of multiple theories of saturation can be attributed to different inferences from data. Under-sampling bias which is very distinct from

the regression to the mean effect, but sometimes confused with it, leads to different saturation limits of the geomagnetic response depending on the sample size of a study (See Supplementary Discussion section 1.i). Further evidence for our conclusion that the saturation effect is the result of uncertainty in the solar wind driver is hiding in plain sight in the space science literature, and is presented in Supplementary Discussion section 1.j.

Conclusion and Generalizability. When correctly interpreted, measurements reveal a linear rather than a saturated relation of geomagnetic response to solar wind driver on average. This implies extreme space weather will have a greater impact on the planet than previous studies ~~suggests~~suggest (around ~200% more at solar wind strengths extrapolated to ~25 mV/m). ~~As we have shown, when correctly interpreted, measurements reveal that the cross-polar cap index and the westward auroral electrojet have a linear relationship with solar wind driving on average, with no saturation limit.~~ It is possible that extreme solar wind driving beyond what we have measured until now may exhibit a saturated geomagnetic response; however, there is no statistical evidence for this so far. Our result is not an alternative theory competing with the existing theories of saturation (Extended Data Table 1), but rather a demonstration that available data does not show evidence for the saturation of geomagnetic response. Hence, it challenges the foundation of existing physical theories of saturation, and they may have to be revisited and validated with the calibrated solar wind driver estimates.

In any field where uncertainty in the driver of a system is significant, and there is interest in the response of the system to extreme and rare instances of driving, the regression to the mean effect can lead to the underestimation of the system's response to extreme drivers. For example, the regression to the mean effect may play a role in climate models, which may underestimate extreme climate events like ~~heatwaves[31]~~heatwaves[22], or the impact of strong earthquakes away from their epicenter, or severe medical symptoms and their resolution, like the effectiveness of back-pain ~~treatments[28]~~treatments[23]. In the above and other fields, biases and even non-linear biases may hide in plain sight and be misunderstood as a physical process.

As artificial intelligence or machine-learning models, which fall under the umbrella of non-linear, non-parametric regression analysis, become popular within physics and other fields with large quantities of measurements, the non-linear bias stemming from heteroskedastic uncertainty or temporal uncertainty is likely present. They may have an impact on prediction and affect any physical inferences drawn from the model outputs, and hence we urge caution in their use and further quantitative investigation on how regression to the mean impacts these models.

The insight that regression to the mean is fundamental to the relation between the truth and its measurement implies that the effect will manifest in different forms within different methods of inference. Hence, all researchers in the fields that attempt to use measurements to infer relations between the physical processes they represent need to be aware of the essence and impact of the logic of regression to the mean. Ignoring this effect can lead researchers and entire fields of inquiry down long, winding paths that deviate from the truth.

MAIN REFERENCES

1. Baker, D. N. What is space weather? *Advances in Space Research* **22**, 7–16 (1998).
2. Gonzalez, W. D. *et al.* What is a geomagnetic storm? *J. Geophys. Res. Space Phys.* **99**, 5771–5792 (1994).

3. Coxon, J. C. *et al.* Extreme Birkeland Currents Are More Likely During Geomagnetic Storms on the Dayside of the Earth. *J. Geophys. Res. Space Phys.* **128**, e2023JA031946 (2023).

4. Eastwood, J. P. *et al.* The Economic Impact of Space Weather: Where Do We Stand? *Risk Analysis* **37**, 206–218 (2017).

5. National Research Council. *Severe Space Weather Events: Understanding Societal and Economic Impacts: A Workshop Report. Severe Space Weather Events--Understanding Societal and Economic Impacts* (National Academies Press, Washington, DC, 2008). doi:10.17226/12507.

~~6~~6. ~~Dungey, J. W. Interplanetary Magnetic Field and the Auroral Zones. *Phys. Rev. Lett.* **6**, 47 (1961).~~

~~7. Cowley, S. ~W. ~H. & Lockwood, M. Excitation and decay of solar wind-driven flows in the magnetosphere-ionosphere system. *Ann. Geophys.* **10**, 103–115 (1992).~~

~~8. Kan, J. R. & Lee, L. C. Energy coupling function and solar wind-magnetosphere dynamo. *Geophys. Res. Lett.* **6**, 577–580 (1979).~~

~~9. Lockwood, M. Solar Wind—Magnetosphere Coupling Functions: Pitfalls, Limitations, and Applications. *Space Weather* **20**, e2021SW002989 (2022).~~

~~10. Troshichev, O. A. & Andrezen, V. G. The relationship between interplanetary quantities and magnetic activity in the southern polar cap. *Planet. Space Sci.* **33**, (1985).~~

~~11. Dunham, D. ~W. ISEE-3, the first libration-point satellite. in *Bulletin of the American Astronomical Society* vol. 11 805–806 (1979).~~

~~12.~~ Troshichev, O. A. PC index as a ground-based indicator of the solar wind energy incoming into the magnetosphere: (1) relation of PC index to the solar wind electric field EKL. *Frontiers in Astronomy and Space Sciences* vol. 9 Preprint at https://doi.org/10.3389/fspas.2022.1069470 (2022).

~~13~~7. Troshichev, O., Hayakawa, H., Matsuoka, A., Mukai, T. & Tsuruda, K. Cross polar cap diameter and voltage as a function of PC index and interplanetary quantities . *J. Geophys. Res. Space Phys.* **101**, (1996).

~~14~~8. Lockwood, M. & McWilliams, K. A. A Survey of 25 Years' Transpolar Voltage Data From the SuperDARN Radar Network and the Expanding-Contracting Polar Cap Model. *J. Geophys. Res. Space Phys.* **126**, e2021JA029554 (2021).

~~15~~9. Borovsky, J. E., Lavraud, B. & Kuznetsova, M. M. Polar cap potential saturation, dayside reconnection, and changes to the magnetosphere. *J. Geophys. Res. Space Phys.* **114**, n/a-n/a (2009).

~~16~~10. Shepherd, S. G. Polar cap potential saturation: Observations, theory, and modeling. *J. Atmos. Sol. Terr. Phys.* **69**, 234–248 (2007).

~~17~~11. Taylor, J. R. (John R. *An Introduction to Error Analysis : The Study of Uncertainties in Physical Measurements*. (University Science Books, 1982).

~~18~~12. Walsh, B. M., Bhakyapaibul, T. & Zou, Y. Quantifying the Uncertainty of Using Solar Wind Measurements for Geospace Inputs. *J. Geophys. Res. Space Phys.* **124**, 3291–3302 (2019).

~~19~~13. Morley, S. K., Welling, D. T. & Woodroffe, J. R. Perturbed Input Ensemble Modeling With the Space Weather Modeling Framework. *Space Weather* **16**, 1330–1347 (2018).

~~20~~14. Milan, S. E., Carter, J. A., Bower, G. E., Fleetham, A. L. & Anderson, B. J. Influence of Off-Sun-Earth Line Distance on the Accuracy of L1 Solar Wind Monitoring. *J. Geophys. Res. Space Phys.* **127**, e2021JA030212 (2022).

~~21~~15. Borovsky, J. E. Noise, Regression Dilution Bias, and Solar-Wind/Magnetosphere Coupling Studies. *Frontiers in Astronomy and Space Sciences* **0**, 45 (2022).

~~22~~16. Sivadas, N. & Sibeck, D. G. Regression Bias in Using Solar Wind Measurements. *Frontiers in Astronomy and Space Sciences* **0**, 159 (2022).

~~23~~17. Barnett, A. G., van der Pols, J. C. & Dobson, A. J. Regression to the mean: what it is and how to deal with it. *Int. J. Epidemiol.* **34**, 215–220 (2005).

18. Methods are available at the end of this Main Text.

~~24~~19. Carroll, R. J., Ruppert, D., Stefanski, L. A. & Crainiceanu, C. M. Measurement error in nonlinear models: A modern perspective, second edition. *Measurement Error in Nonlinear Models: A Modern Perspective, Second Edition* 1–455 (2006).

~~25~~20. Veselovsky, I. S., Dmitriev, A. V. & Suvorova, A. V. Lognormal, Normal and Other Distributions Produced by Algebraic Operations in the Solar Wind. *AIP Conf. Proc.* **1216**, 152 (2010).

~~26. Methods are available at the end of this Main Text.~~

~~27. Bonilla, M. G., Mark, R. K. & Lienkaemper, J. J. Statistical relations among earthquake magnitude, surface rupture length, and surface fault displacement. *Bulletin of the Seismological Society of America* **74**, 2379–2411 (1984).~~

~~28. Whitney, C. W. & Von Korff, M. Regression to the mean in treated versus untreated chronic pain. *Pain* **50**, 281–285 (1992).~~

~~29~~21. Vasyliunas, V. M. Concepts of Magnetospheric Convection. in *The Magnetospheres of the Earth and Jupiter* (ed. Formisano, V.) 179–188 (Springer Netherlands, Dordrecht, 1975).

~~30.~~22 ~~Borovsky, J. E. On the Saturation (or Not) of Geomagnetic Indices. *Frontiers in Astronomy and Space Sciences* **8**, 175 (2021).~~

~~31~~. Kornhuber, K., Bartusek, S., Seager, R., Schellnhuber, H. J. & Ting, M. Global emergence of regional heatwave hotspots outpaces climate model simulations. *Proceedings of the National Academy of Sciences* **121**, e2411258121 (2024).

23. Whitney, C. W. & Von Korff, M. Regression to the mean in treated versus untreated chronic pain. *Pain* **50**, 281–285 (1992).

FIGURE LEGENDS

Figure 1 ***Saturation of geomagnetic activity with solar wind driving.*** *a) Solar wind merging electric field* (E_m^*) *measured upstream of the bow-shock, is transformed to the shocked solar wind driver in the magnetosheath* (E_m^{sh}) *downstream of the bowshock, which corresponds to the dawn-dusk electric field that maps to the polar ionosphere* (E_{PC})*; b) Observations from 1995-2019, green curve, shows that on average the cross polar cap index (*$\propto$ *the dawn-dusk electric field* E_{PC}*) increases linearly at low values of solar wind driving, but deviates from linearity and saturates at large values of driving. The magenta curve shows that the error model developed in this work predicts the same saturation effect due to uncertainty in the solar wind driver instead of any physical mechanism. The transparent shaded area for both curves shows a 95% confidence interval.*

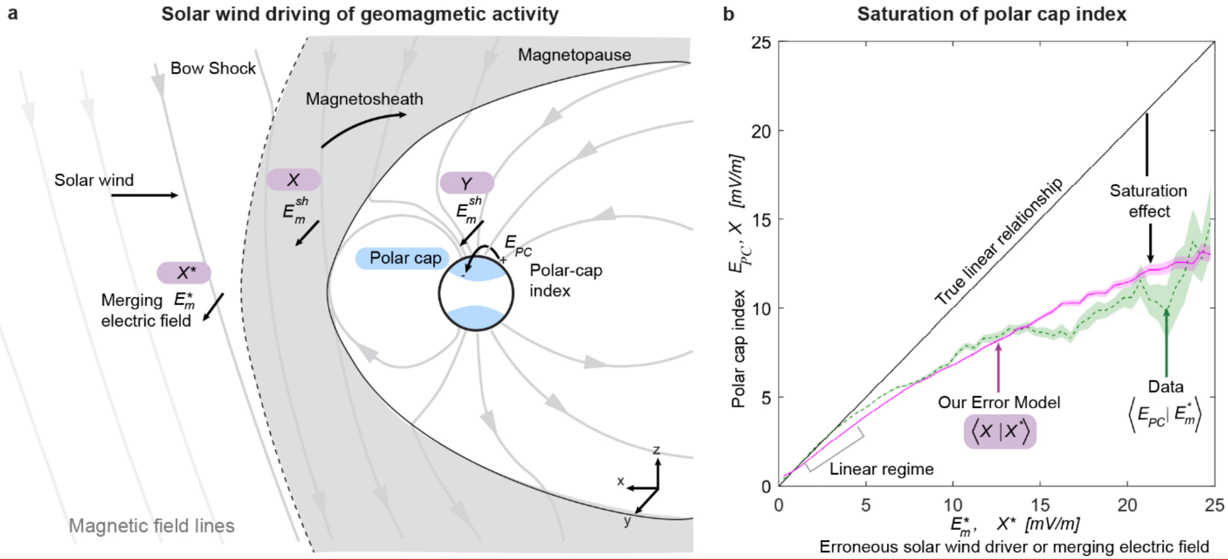

Figure 2 ***Explaining regression to the mean effect using probability theory and regression bias.*** *a) For a physical parameter* X *which has an underlying probability distribution, some values are more likely than others, and hence when the measurement is* X^**=3 the corresponding true value is more likely to be* $X = 2$ *than* $X = 3$*; b) The expected true value of a Gaussian process* X *has a linear bias when measurement is* $X^* = X + \epsilon$ *with a constant random error* (ϵ)*; c) The expected true value of a log-normal process* X *has a non-linear bias when the measurement* X^* *has random error* $(\epsilon(X))$ *that varies with the strength of* X*. This figure is adapted from Sivadas & Sibeck 2022[16], used under CC BY 4.0. The transparent shaded area for regression curves shows a 95% confidence interval.*

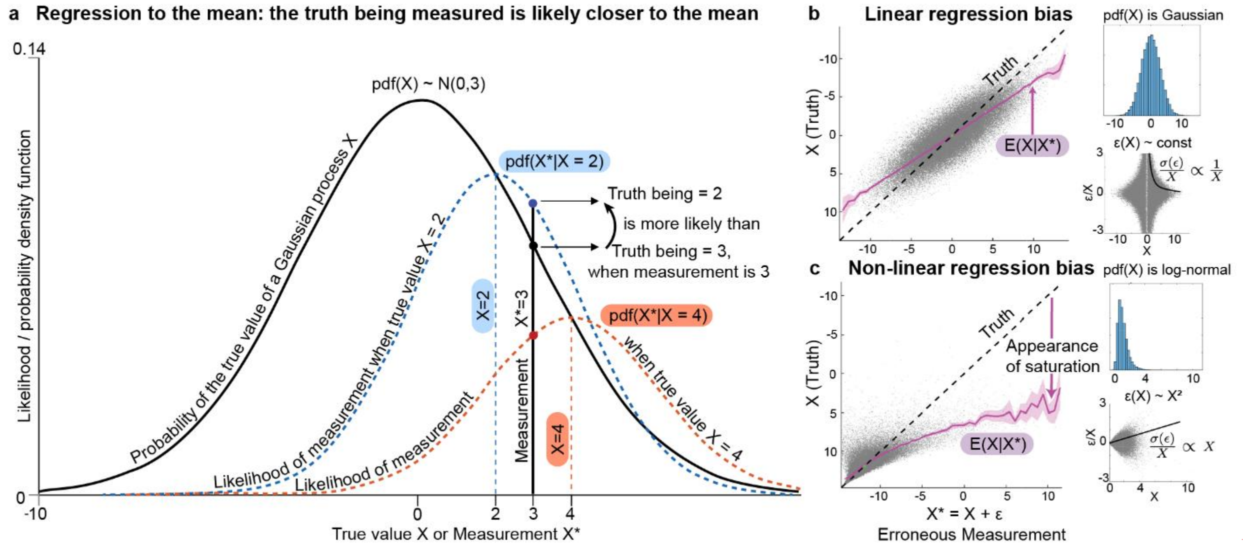

Figure 3 ***Regression bias predicted by the error model matches saturation effect from data.*** *a) Conditional probability density function of 25 years of measurements* $PCI|E_m^* \sim E_{PC}|E_m^*$*; b) Conditional probability density function reproduced by the error model* $X|X^*$ *which is very similar to the measurements presented in panel a. The transparent shaded area for both regression curves shows a 95% confidence interval; c) Non-linear bias caused by uncertainty in time increases with a measure of the uncertainty represented by the dimensionless ratio of the time uncertainty* (δ) *and the autocorrelation constant* (k)*; d) Quantified relative uncertainty* $\epsilon(X)/X$ *due to random variations in solar wind driver measured at L1* (E_m^*, X^*) *for a measured value of the shocked solar wind driver* (E_m^{sh}, X)*.*

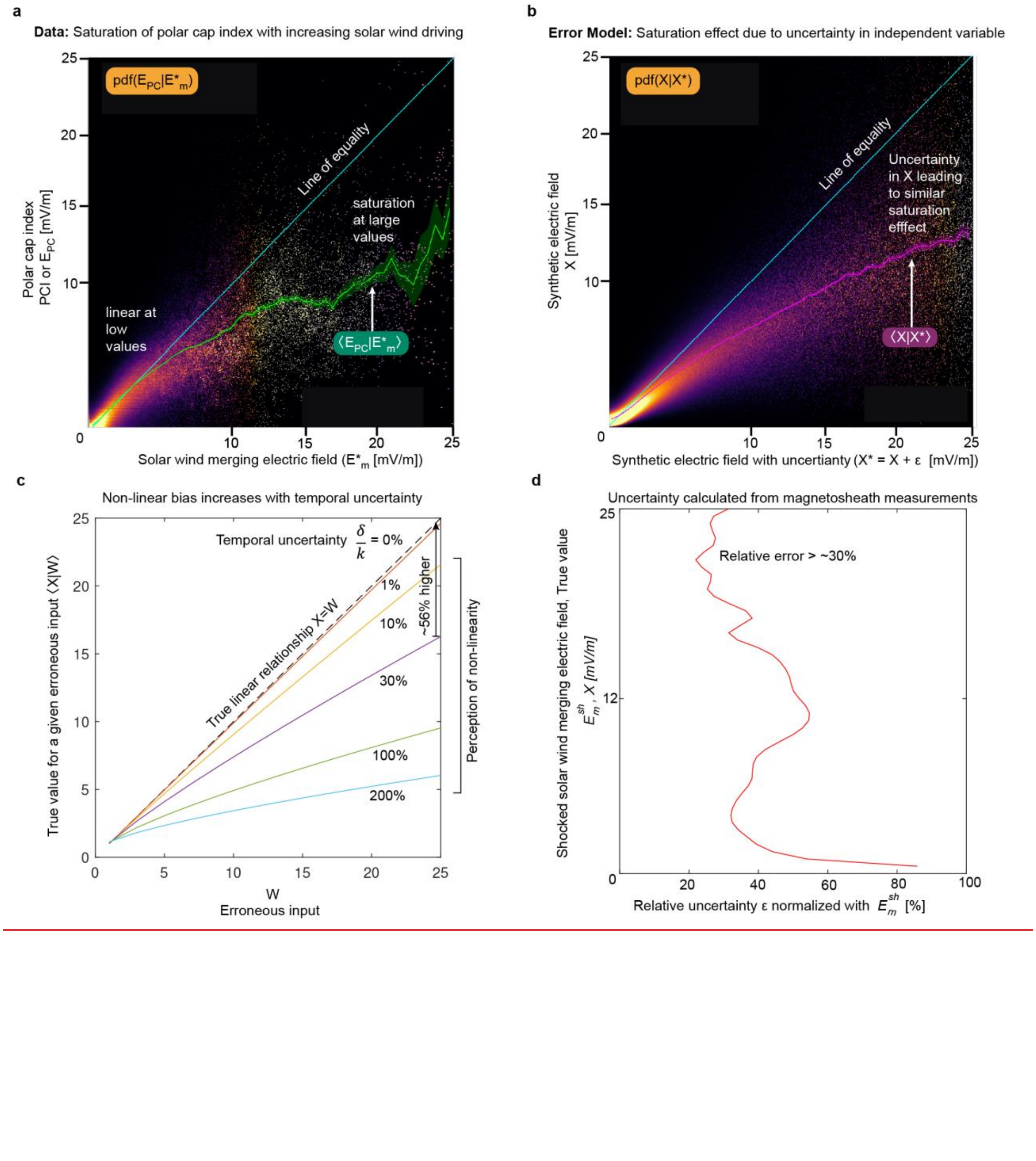

Figure 4 ***Correcting the effect of random errors in solar wind driver values reveals a linear geomagnetic response.*** *a) Polar cap index varies linearly with solar wind driving on average (purple curve), after correcting the regression bias in the erroneous solar wind driver values (green curve); b) The westward auroral electrojet strength also varies linearly with solar wind driving (E_m^c) on average (purple curve) after the same regression bias correction applied to the erroneous solar wind driver values (E_m^*) (green curve). The transparent shaded area for all the curves shows a 95% confidence interval.*

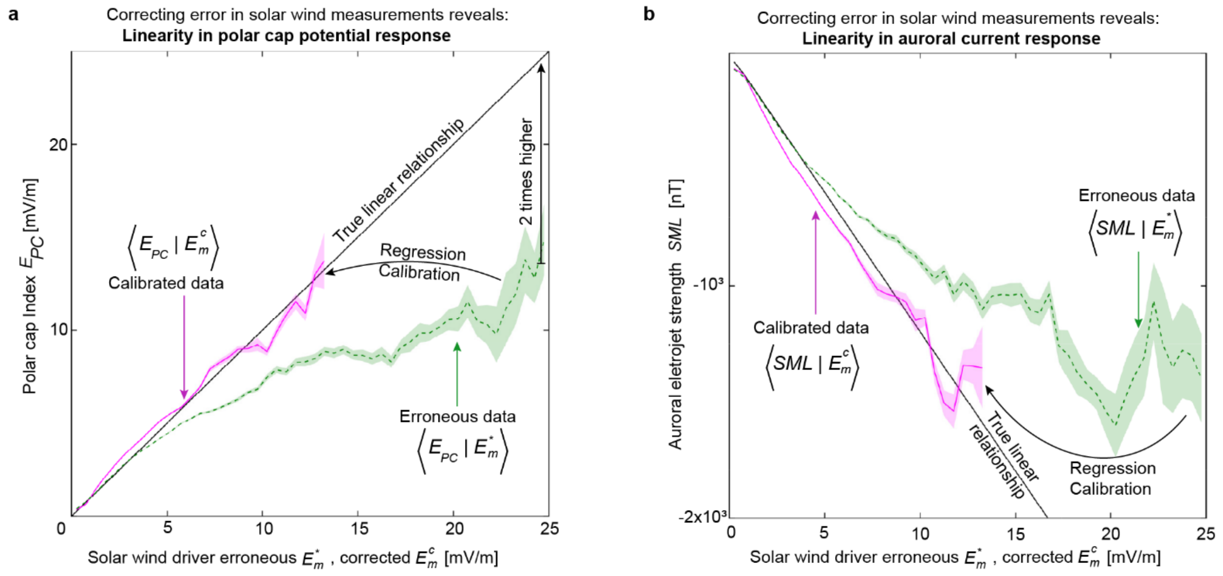

Extended Data Figure 1| Stochastic properties of inputs to the error model. a, The black line shows the pdf of the polar cap index E_{PC} from 1995 to 2019. And the purple line shows the lognormal pdf of the model random variable X that best fits the pdf of E_{PC}. **b,** The black dashed line shows the autocorrelation function of the polar cap index E_{PC} averaged over data from 1995 to 2019. And the purple line shows the autocorrelation function of the model random variable X that closely follows the ACF of E_{PC}. **c,** The black line shows the uncertainty in the propagation delay from L1 to the bow-shock nose(dt_1). The blue line shows the uncertainty in the propagation delay from the bow-shock nose to the polar cap ionosphere(dt_2). The magenta line shows the total uncertainty in the propagation delay from L1 to the polar cap($dt_1 + dt_2$).

Extended Data Figure 2| Statistics of the output of the error model. a, The model predicts with good correspondence the pdf of the estimate of the merging electric field propagated to the polar cap, with a probability density difference of less than ~0.1 between data and model for most values. E_m^* is data, and X^* is model. **b,** The model predicts the nature of the random error between the estimate and the true value of the merging electric field projected on the ionosphere. The black line is the normalized uncertainty associated with E_m^* (data), and the purple line is the normalized uncertainty associated with X^* (model). **c,** There is a non-linear conditional bias in E_m^*: $\langle E_m^* - E_{PC} | E_{PC} \rangle$ that varies with E_{PC}, which is reproduced very well by the model $\langle X^* - X | X \rangle$. **d,** Conditional normalized error distribution from data, **e,** Conditional normalized error distribution from the model. Both data and model show similar trends in how the error varies with increasing strength of the true variable –a steep increase in the spread of the error from 0 to ~12 mV/m in the vertical axis, and then a decrease (or loss of power) beyond that point.

Extended Data Figure 3| Another graphical explanation of the regression to the mean effect. (Left) Probability distribution function of the true variable X, with each bin colored uniquely. (Right) The probability distribution function of the inaccurate estimate of X, i.e., X^* with the color representing the magnitude of X. The amount of a particular color per bin represents the proportion of X values misidentified as X^*.

Extended Data Figure 4| Sensitivity analysis of magnitude uncertainty. a, Non-linear regression bias caused by heteroskedastic magnitude uncertainty increases with a measure of the uncertainty represented by the minimum relative magnitude uncertainty. **b,** The heteroskedastic variation of the magnitude uncertainty with the true value X is shown, for different values of the minimum relative magnitude uncertainty corresponding to the different regression bias curves in panel a.

Extended Data Figure 5| Increasing trend of the saturation limits observed by previous observational studies. a, Cross polar cap potential response to solar wind driving is linear up to a saturation limit, which varies depending on the observational study and the methodology they use. All except Boyle et al., 1997 [24] infer a saturation beyond the limit in E_m^*. See the studies here: 1 [25], 2 [26], 3 [27], 4 [24], 5 [28], 6 [29], 7 [30], 8 [31], 9 [32]. **b,** The saturation limit of different studies follow an increasing trend (linear regression) with the year of study, with the latest studies including higher number of data samples.

Extended Data Table 1| Theories of polar cap potential saturation. Ten models explaining cross polar cap potential saturation. See the following works for more details about each of the models: 1 [33,34], 2 [35,36], 3 [37,38], 4 [39], 5 [38,40], 6 [41], 7 [42], 8 [43], 9 [44], 10 [45,46] and an assessment of most of them[9]. Adapted with permission from [Joseph E. Borovsky, Benoit Lavraud, Maria M. Kuznetsova, Table A1, Polar cap potential saturation, dayside reconnection, and changes to the magnetosphere., Journal of Geophysical Research: Space Science, Publisher: John Wiley and Sons] Copyright 2009 by American Geophysical Union.

Extended Data Table 2| Common symbols used in this work. Variables representing measurements and their corresponding counter parts in the error model used commonly through the paper are summarized here.

METHODS

~~METHODS~~

~~Overview~~

~~In this section, we discuss the methods used to demonstrate that polar cap potential saturation is a result of solar wind measurement uncertainty, calibrate the error to reveal the linear response of the polar cap potential and auroral current strengths due to solar wind forcing, and present the analytical derivation of uncertainty in measurement times causing a perception of non-linearity in a linear system's correlation response.~~ The most common symbols used are explained in the Extended Data Table 2.

Solar wind measurement uncertainty

There are several solar wind coupling functions that attempt to quantify the driving, one important one is the solar wind merging electric field or merging geoeffective field E_m^* ~~[8,16]~~[10,47].

$$E_m^* = V_{sw} B_{T,sw} \sin^2(\theta_{sw}/2) \quad [1.1]$$

This field is also known as the Kan-Lee electric field and is calculated from solar wind parameters at Lagrange point L1 ~~alone[8]~~alone[47]. In equation 1.1, V_{sw} is the solar wind speed in km/s, $B_{T,sw} = \sqrt{B_y^2 + B_z^2}$ is the transverse magnitude of the interplanetary magnetic field (IMF) in nT, and $\theta_{sw} = \tan^{-1}(B_y/B_z)$ is the transverse IMF clock angle in radians. E_m is a positive-valued quantity with the units of the electric field [mV/m], reflecting the assumption that energy flows only from the solar wind to the magnetosphere. The above quantities are measured in Geocentric Solar Magnetospheric (GSM) coordinates using spacecrafts orbiting the L1 point ~230 earth Radii (R_E) upstream from Earth. The GSM coordinates are convenient for studying the effects of the IMF components on magnetospheric and ionospheric phenomena. Its X-axis points towards the Sun, and the Z-axis is the projection of the Earth's magnetic dipole axis onto the plane perpendicular to the X-axis. For purely southward IMF, the merging electric field E_m^* is equal to the solar wind electric field $E_{sw} = V_{x,sw} B_z$, where $V_{x,sw}$ is a component of the solar wind velocity along the x-direction.

The dawn-dusk portion of the shocked solar wind convection electric field maps along the equipotential magnetic field lines and drives the plasma convection down in the polar cap ionosphere. The convection corresponds to an electric field across the polar cap in the rest-frame of the Earth (E_{PC}). The polar cap (PC) index is a measure of this field and part of the energy input into the Earth's magnetosphere. The index has gone through many iterations over the past 50 years, but it is essentially the maximum amplitude of variations observed from magnetometers near the south and north ~~pole[12,32,33]~~pole[6,48,49]. The current version of the index is scaled on a statistical basis of magnetic variations to the merging electric field, such that the index is highly correlated to the merging electric field. This makes the PC index independent of daily and seasonal variations and the local ionospheric properties. PCN is the index derived from the magnetometer in the northern polar cap, while PCS is derived from the southern polar cap. We use the PCI index as a proxy for E_{PC} (i.e. $PCI \sim E_{PC}$), as it combines both the indices to provide a positive-valued index, which is more accurate than the individual ~~indices[33]~~indices[49]. PCI=0.5*(PCS+PCN), with a condition that negative values of either PCS or PCN are set to 0. PCI or E_{PC} also has the units of the electric field, and several statistical analyses in literature reveal that it is linearly proportional on average to the electric potential across the polar cap, called the cross-polar cap ~~potential[13,34].~~potential[7,50].

Fluctuations in PCI can occur due to night-side magnetosphere processes[35]processes[51], but the above relation is still maintained on average.

The Kan-Lee electric field is an approximation of the component of the reconnection electric field ($E_R = V_{sh}B_{sh}\sin(\theta'/2)$) perpendicular to the geomagnetic field that creates the cross-polar cap electric field (which is equal to $V_{sh}B_{sh}\sin^2(\theta'/2)$ due to a geometrical argument presented first by Kan and Lee)[8]. The approximation assumes that the shear angle between the magnetosheath and magnetospheric magnetic field θ' is equal to θ_{sw}. Furthermore, the approximation assumes $V_{sh}B_{sh} \simeq V_{sw}B_{T,sw}$, which is true most of the time 1) as the solar wind velocity component tangential to the bowshock is negligible compared to the normal component, and 2) as the tangential component of the electric field across a normal MHD shock is conserved. This allows the Kan-Lee electric field (E_m^*) to estimate the cross polar electric field E_{PC} with just solar wind parameters. Our examination of data shows that for electric field values <3mV/m, which is 95% of all available data points in 25 years, the Kan-Lee electric field (E_m^*) propagated (or time-shifted) to the polar cap ionosphere from the L1 point is linearly proportional to the cross polar electric field E_{PC}. This is well-documented in literature and is also observed for other forms of the solar wind coupling functions and polar cap index [16,33,36]. However the fact thatThe Kan-Lee electric field E_m^* is only an approximation of the true driver of the magnetosphere i.e., the shocked solar wind plasma $E_m^{sh} = V_{sh}B_{sh}\sin^2(\theta'/2)$, whose value is not easily available to us (See Supplementary Methods 2.a,). This leads to the uncertainty and non-linear regression bias discussed in this work. Instead, we only have an erroneous estimate of the true coupling function approximately propagated to the polar cap, E_m^*. Hence, we need to reinterpret the literature as saying that low values of the erroneous estimate of the solar wind strengths E_m^* (not E_m^{sh}) correlate linearly with the polar cap potential. And at high values of E_m^*, the polar cap potential saturates. The saturation seems to have the following functional form[33]:

$$E_{PC} = \frac{E_m^*}{\sqrt{1 + (E_m^*/E_0)^2}}$$

We calculate E_m^* by using WIND satellite measurements published in the OMNIWeb database[37]database[52]. The database provides the values corrected for the propagation delay of the solar wind from L1 to the bow-shock nose [38–40][53–55]. Then as commonly done, we apply a further correction of a constant delay of ~17 minutes to account for the propagation delay from the nose to the polar cap ionosphere[20,41].ionosphere[14,56]. Previous literature that discusses the polar cap potential saturation problem estimates E_m^* similarly. We use the WIND data from 1995 to 2019 and the polar cap indices from the same time range. Both data are 1-minute averages, larger time averages will lead to additional uncertainties[42].uncertainties[57]. We only use data samples when both WIND measurements and polar cap indices are available.

E_m^* can differ from the shocked solar wind driver of the magnetoshere E_m^{sh} in two ways. One is through a consistent deterministic bias, that is determined by the bow-shock that slows down the plasma. This deterministic bias is nearly zero as the tangential (and the largest) component of the electric field across the bow-shock remains unchanged. We do not concern ourselves with this deterministic bias, as it will manifest in the data as a physical effect anyway. However, E_m^* can also differ randomly from the true solar wind driver E_m^{sh}, due to fluctuations in the plasma properties caused by physical processes between L1 and the reconnection site, acting in random

directions. These random fluctuations contribute to the uncertainty in E_m^* when we use it as a proxy for the true driver E_m^{sh}. This random error is concerning, as it does not average away, and manifests as a regression bias in data analysis and is easily confused as a physical effect, hence we calculate and correct for it.

We categorize the random uncertainty in E_m^* relative to the true value E_m^{sh} into three primary sources:

1. Uncertainty in propagation delay of the solar wind from L1 to the bow-shock nose (dt_1)
2. Uncertainty in the propagation delay of the effect of solar wind forcing from bow-shock nose to the polar cap ionosphere (dt_2)
3. Random variability in the shocked solar wind due to spatial variation in the incoming solar wind and transformations of its plasma parameters during propagation through the bow-shock and the magnetosheath

Knowing the statistical distribution of the above uncertainty will allow us to construct a stochastic model of the estimate E_m^* as a function of the true driver E_m^{sh} mapped to the polar cap ionosphere. In the following subsection, we develop a statistical error model of the estimate of the true shocked solar wind that drives the cross-polar cap convection from an estimate of its stochastic properties and uncertainty distributions. For the calculated uncertainties (Figure 3d), the model predicts the polar cap potential saturation, which is strikingly similar to data from 25 years of observations (see Figure 1b and Figure 3a-b).

Statistical error model

To distinguish between data and model, we will replace E_m^{sh} with X when referring to the random variable corresponding to the shocked solar wind driver in the statistical model. We also replace the polar cap index PCI, which is proportional to the convection electric field E_{PC} in the ionosphere with a counterpart in the model Y_{PC}. Hence, in the model, X is the shocked solar wind driver accurately time-shifted to the polar cap, and X^* is its erroneous estimate (i.e. solar wind driver measured at L1 or E_m^*). We hypothesize the following statistical error model:

$$\mathrm{X}^*(t) = \mathrm{X}(t + dt_1 + dt_2) + \varepsilon(t) \quad [1.2]$$

We assume that X^*, X, dt_1, dt_2 , and ε are stochastic processes. In other words, they are each a collection of random variables in time ($t \in T$) with an associated probability distribution that determines the random value it might take at a given time t. Below we present our estimates of the probability distributions and autocorrelation functions of these stochastic processes. Using these estimates of X, dt_1, dt_2, and ε, we calculate X^*. After which, we validate the model results by comparing them with the data E_m^*.

Input X: If reconnected open-field lines in the polar cap region do not have large parallel resistances, accurate mapping of the shocked solar wind electric field onto the polar cap should result in X, such that $Y_{PC} \propto$ X. In agreement with this, in data, we see that, the probability density function (pdf) of the PCI index or E_{PC} (and hence Y_{PC}) and solar wind driver E_m^* are very similar, implying the distribution of Y_{PC} is also similar to X. As a result, we assume that the pdf of X is similar to the pdf of E_{PC} (and E_m^*) that we can estimate from data. Note that this is not surprising

since E_{PC} is constructed to be highly correlated with E_m^*, and from Kan and Lee's arguments E_m^* is also correlated with E_m^{sh}, and hence all these parameters have similar probability distribution functions. (Though we have measurements of E_m^{sh} from near-Earth orbiting spacecraft, they are discontinuous and hence do not directly provide an unbiased pdf of X.) Like many solar wind parameters, E_{PC} and E_m^* can be approximated to be a lognormal ~~distribution~~[~~25~~]~~.~~distribution[20]. Therefore, we assume the pdf of model input X to be a lognormal distribution that closely fits the pdf of E_{PC} from data (See Extended Data Figure 1a). We also assume the adjacent values of X in time are correlated similarly to adjacent values of E_{PC} in time (See Extended Data Figure 1b), as fluctuations in time of shocked solar wind electric field should correlate with that of the cross-polar cap electric field in the ionosphere. In other words, **we assume that in our model, X shares the stochastic properties of the polar cap index or electric field E_{PC}.** If the assumption fails, the results of the model, particularly the second order statistics, will be inconsistent with what is observed in the data. Finally, we assume X is a stationary process, i.e., its probability distribution and autocorrelation function does not change with time.

Uncertainty in propagation delay from L1 to Nose dt_1: Solar wind parameters measured upstream are time-shifted to account for the delay in propagation of the wind to the bow-shock nose. Case and Wild estimate the uncertainty to be on the order of minutes [~~43~~58]. Based on their results, we assume dt_1 to be an independent random process with a pdf of a Student's t-distribution with shape factor 1.3, mean 0, and standard deviation ~8 minutes (See Extended Data Figure 1c). The distribution is zero-mean and has a longer tail than the normal distribution.

Uncertainty in propagation delay from nose to polar cap dt_2: Changes in the day-side shocked solar wind electric field propagate along equipotential open magnetic field lines to the polar caps. The delay in this propagation is on average 20 minutes but can vary from -5 to 50 ~~minutes~~[~~34~~]minutes[50]. Historically, researchers have used a constant propagation delay (t_2) of about ~20 minutes for this stage of propagation. However, the uncertainty of propagation time here is significant. We model the pdf of t_2 as a Weibull distribution with mean ~ 17 ~~minutes~~[~~20~~]minutes[14], standard deviation ~ 25 minutes, and shape factor 1.3 (See Extended Data Figure 1c). The Weibull distribution keeps the total delay $t_2 + dt_2$ positive and captures the broad spread in the propagation delay documented by ~~Stauning~~[~~33,34~~]Stauning[49,50].

Magnitude uncertainty ε: There are several other reasons for the magnitude of the shocked solar wind driver to be randomly different from its proxy measured at the L1. The IMF clock angle could change substantially after crossing the bow shock, spatial variations in the solar wind can lead to a different part of the wind interacting with the Earth's magnetosphere, and changes in the magnetospheric state or its history can lead to changes in the local plasma and field conditions in magnetosheath. As the solar wind strength increases, the random variation in the field can increase due to the increased spatial structuring of the solar wind, and any clock angle variation during increased field strength can lead to larger variations in geoeffectiveness.

To estimate these random variations, we use direct evidence from a database of simultaneous measurements of plasma and field strengths in the magnetosheath. Using a gradient boost classification algorithm, measurements from near-Earth satellites such as THEMIS, MMS, DoubleStar, and Cluster, are classified into solar wind, magnetosheath, and magnetosphere ~~regions~~[~~44~~]regions[59]. Our interest is in the measurements made within the magnetosheath, close to the magnetopause in the subsolar region (|Y|<5 RE, and |Z| < 5 RE). The distance from the

magnetopause boundary is determined using a machine-learning based empirical model of the boundary, which performs better than other magnetopause models available in ~~literature~~[45]literature[60]. For specific values of the magnetosheath measurements (E_m^{sh}), we calculate the variance in the measurements made at L1 and time-shifted to the bow-shock (E_m^*). This variance is an estimate of the uncertainty in the solar wind driver E_m^* for a given value of the shocked solar wind driver E_m^{sh}. The "magnitude uncertainty" ε is set to the same variance, and we model it as a zero-mean Gaussian with a standard deviation that varies with the magnitude of X according to data. The magnitude uncertainty is not constant with the strength of the shocked solar wind driver, instead it increases until E_m^{sh}~12 mV/m, after which the statistics become poor and an accurate estimate of the variance becomes challenging. In this regime, we estimate the uncertainty in the solar wind driver relative to measurements of near-Earth satellites just upstream of the bow-shock (~10<X<50 R_E). This uncertainty will be the minimum uncertainty for values >12 mV/m (i.e., a conservative estimate of the uncertainty), and this remains roughly the same with increasing shocked solar wind driver value. Figure 3d shows the relative error, i.e. $\sigma(\varepsilon)/X$, and how that varies with X. The 'heteroskedastic' behavior of this uncertainty is consistent with the observed statistical variations in the difference between solar wind driver and the polar cap index shown in Extended Data Figure 2d-e. The variation in their difference increases up to a polar cap index of ~12 mV/m and then remains constant. We also assume that ε has an autocorrelation function similar to that of the difference between the observed solar wind driver E_m^* and the polar cap index $PCI \sim E_{PC}$.

Using the above estimates of uncertainties, we generate an ensemble of time series of X, dt_1, dt_2, and ε, and calculate the corresponding time series of the erroneous estimate X^* using equation [1.2]. The statistical properties of the model output agree remarkably with that of the data.

Validation of Error Model

We validate the error model by comparing the second order statistics of the error model parameters with that of their counterparts in data. The error model predicts the pdf of the solar wind driver E_m^*, the standard deviation of the normalized error, the conditional normalized error distribution, and finally the regression bias stemming from the regression to the mean effect. This validation is the rationale for using the error model and is presented in the Supplementary Methods 2.b. Supplementary Methods 2.d summarizes the full computer code used. Additionally, we show a sensitivity analysis of the non-linear regression bias, which increases with increasing uncertainty in the time of propagation (See Figure 3c), and with increasing uncertainty in the magnitude uncertainty ε (See Extended Data Figure 4).

Correcting the error

The regression bias $b = X^* - \langle X|X^* \rangle$, which is the consistent deviation between the measurement and the likely true value given the measurement. The erroneous measurement X^* can now be corrected to X^c by subtracting the bias from it $X^c = X^* - b$, which simply reduces to $X^c = \langle X|X^* \rangle$. Crucially, from the model output, we can calculate the conditional expectation of the true value given the erroneous estimate: $f_r(x) = \langle X|X^* = x \rangle$, i.e., we have a statistical, functional relationship between the true value and the erroneous measurement. For the particular assumptions of uncertainties in measurement, $f_r(x)$ is the best estimate of the true value (X^c or E_m^c) given the erroneous value (X^* or E_m^*). Therefore, $f_r(x)$ can be used to statistically correct the erroneous estimate E_m^* to a most likely estimate of the true value E_m^c. E_m^c is useful for revealing the true statistical correlation with other variables, like the polar cap index E_{PC} or auroral current strengths

SML. The process of statistically correcting for the uncertainty using the conditional expectation of the true value given the erroneous estimate: $E_m^c = f_r(\mathrm{E}_m^*)$ for an unbiased regression analysis is called regression ~~calibration~~[24]~~.~~calibration[19]. Figure 4 shows the results of this calibration. SML is the westward auroral electrojet index from the SuperMAG database, which is a measure of the westward currents flowing in the auroral ~~regions~~[46,47]regions[61,62]. It is calculated using ground magnetometers and procedures completely independent of the calculation of the polar cap index. Since regression calibration of E_m^* to E_m^c, results in a linear relationship between both E_m^c and E_{PC} (Figure 4A) and E_m^c and SML (Figure 4B), it suggests our findings are independent of the construction of the ionospheric response variables. We note here that the confidence intervals shown in Figures 1-4 only provide an estimate of the reliability of the regression procedure, i.e., it means 95% probability that repeating the procedure will contain the result and does not mean the true value or the true regression curve will lie within the confidence interval with a 95% probability.

Temporal uncertainty can lead to a non-linear regression function

In Supplementary Methods 2.c we analytically derive the following general result that temporal uncertainty in measurement can lead to a perception of non-linearity in a linear system's correlation response. For a linear system with response Y, input X, and the input with measurement error $W = X(t + \Delta)$, the biased system response $\langle Y|W\rangle$ is a function $f_r^*(w, \frac{\sigma_\delta}{k})$. Here σ_δ is a measure of the random uncertainty Δ in the time of measurement. And k is the autocorrelation time constant. The system response is a function of the ratio $\frac{\sigma_\delta}{k}$. This ratio is a measure of uncertainty in the time of measurement. We numerically integrate the function, which is fully described in equation [1.12] in the Supplementary Methods 2.c, within the ranges of Δ for specific values of $\frac{\sigma_\delta}{k}$ and generate Figure 3c. The pdf of X is similar to that assumed in the previous section, with the mean $\mu_X = 1.12$ and standard deviation $\sigma_X = 1.15$. The figure shows that $f_r^*(w, \frac{\sigma_\delta}{k})$ varies non-linearly with w when the temporal uncertainty ratio is larger than 0%, compared to $f_r(x) = x$ (which is linear) when $W = X$ or the temporal uncertainty ratio is 0%. Therefore, we have found that the regression function $\langle Y|W\rangle = f_r^*(w, \frac{\sigma_\delta}{k})$ is non-linear with respect to w for values of $\frac{\sigma_\delta}{k} > 0$.

Data Availability

All data we have used is publicly available. We thank GSFC/SPDF OMNIWeb service for the WIND spacecraft measurements ~~48~~63. The WIND spacecraft measurements of the solar wind propagated to the bow-shock can be accessed from https://spdf.gsfc.nasa.gov/pub/data/omni/high_res_omni/sc_specific/. The MMS, Cluster, DoubleStar, and THEMIS data can be accessed from GSFC/SPDF web service https://spdf.gsfc.nasa.gov/. The polar cap index values PCN and PCS were downloaded from https://pcindex.org/archive. We thank Dr. Oleg Troshichev of the Arctic and Antarctic Research Institute for this data (https://pcindex.org/contacts). The auroral electrojet indices were downloaded from the SuperMAG database https://supermag.jhuapl.edu/indices/. For SuperMAG indices we gratefully acknowledge the SuperMAG collaborators (https://supermag.jhuapl.edu/info/?page=acknowledgement). This Zenodo repository archives the data used in the ~~manuscript~~[49]manuscript[64] (https://doi.org/10.5281/zenodo.17546718).

Computer Code

All the relevant data and the MATLAB and Python codes to process and visualize them have been curated and uploaded in the following GitHub repository https://github.com/nithinsivadas/polar-cap-saturation.git, and archived ~~here[50]~~here[65] (https://doi.org/10.5281/zenodo.17559999). For non-MATLAB and non-Python users the code and its outputs are accessible as html files with detailed comments and plots (See https://nithinsivadas.github.io/polar-cap-saturation/).

METHODS REFERENCES

~~32. Troshichev, O. & Sormakov, D. PC index as a proxy of the solar wind energy that entered into the magnetosphere: 2. Relation to the interplanetary electric field e KL before substorm onset. *Earth, Planets and Space* **67**, 1–11 (2015).~~

~~33. Stauning, P. The polar cap (PC) index combination, PCC: Relations to solar wind properties and global magnetic disturbances. *Journal of Space Weather and Space Climate* **11**, 19 (2021).~~

~~34. Stauning, P. The Polar Cap PC Indices: Relations to Solar Wind and Global Disturbances. in *Exploring the Solar Wind* (InTech, 2012). doi:10.5772/37359.~~

~~35. Lockwood, M., Cowley, S. W. H. & Freeman, M. P. The excitation of plasma convection in the high-latitude ionosphere. *J. Geophys. Res. Space Phys.* **95**, 7961–7972 (1990).~~

~~36. Doyle, M. A. & Burke, W. J. S3-2 measurements of the polar cap potential. *J. Geophys. Res. Space Phys.* **88**, 9125–9133 (1983).~~

~~37. King, J. H. & Papitashvili, N. E. Solar wind spatial scales in and comparisons of hourly Wind and ACE plasma and magnetic field data. *J. Geophys. Res. Space Phys.* **110**, 2104 (2005).~~

~~38. Weimer, D. R. *et al.* Predicting interplanetary magnetic field (IMF) propagation delay times using the minimum variance technique. *J. Geophys. Res. Space Phys.* **108**, 1026 (2003).~~

~~39. Haaland, S., Paschmann, G. & Sonnerup, B. U. Ö. Comment on "A new interpretation of Weimer et al.'s solar wind propagation delay technique" by Bargatze et al. *J. Geophys. Res. Space Phys.* **111**, 6102 (2006).~~

~~40. Knetter, T., Neubauer, F. M., Horbury, T. & Balogh, A. Four-point discontinuity observations using Cluster magnetic field data: A statistical survey. *J. Geophys. Res. Space Phys.* **109**, 6102 (2004).~~

~~41. Troshichev, O. A., Janzhura, A. S. & Stauning, P. Magnetic activity in the polar caps: Relation to sudden changes in the solar wind dynamic pressure. *J. Geophys. Res. Space Phys.* **112**, 11202 (2007).~~

~~42. Laundal, K. M. *et al.* Time-scale dependence of solar wind-based regression models of ionospheric electrodynamics. *Scientific Reports 2020 10:1* **10**, 1–9 (2020).~~

~~43. Case, N. A. & Wild, J. A. A statistical comparison of solar wind propagation delays derived from multispacecraft techniques. *J. Geophys. Res. Space Phys.* **117**, 2101 (2012).~~

~~44. Nguyen, G. *et al.* Massive Multi-Mission Statistical Study and Analytical Modeling of the Earth's Magnetopause: 1. A Gradient Boosting Based Automatic Detection of Near-Earth Regions. *J. Geophys. Res. Space Phys.* **127**, (2022).~~

45. Michotte de Welle, B. *et al.* Global Environmental Constraints on Magnetic Reconnection at the Magnetopause From In Situ Measurements. *J. Geophys. Res. Space Phys.* **129**, e2023JA032098 (2024).

46. Gjerloev, J. W. The SuperMAG data processing technique. *J. Geophys. Res. Space Phys.* **117**, 9213 (2012).

47. Newell, P. T. & Gjerloev, J. W. Evaluation of SuperMAG auroral electrojet indices as indicators of substorms and auroral power. *J. Geophys. Res. Space Phys.* **116**, (2011).

48. Lepping, R. P. *et al.* The WIND magnetic field investigation. *Space Science Reviews 1995 71:1* **71**, 207–229 (1995).

49. Sivadas, N. *et al.* Simultaneous measurements of solar wind from L1, shocked solar wind from Magnetosheath, and measures of geomagnetic response from ground-based indices . Preprint at https://doi.org/10.5281/zenodo.17546718 (2026).

50. Sivadas, N. Code for Regression to the Mean of Extreme Geomagnetic Storms . Preprint at https://doi.org/10.5281/zenodo.2417559999 (2026).

51. Boyle, C. B., Reiff, P. H. & Hairston, M. R. Empirical polar cap potentials. *J. Geophys. Res. Space Phys.* **102**, 111–125 (1997).

52. Pulkkinen, T. I. *et al.* Magnetosheath control of solar wind-magnetosphere coupling efficiency. *J. Geophys. Res. Space Phys.* **121**, 8728–8739 (2016).

53. Barnett, A. G., van der Pols, J. C. & Dobson, A. J. Regression to the mean: what it is and how to deal with it. *Int. J. Epidemiol.* **34**, 215–220 (2005).

54. Carroll, R. J., Ruppert, D., Stefanski, L. A. & Crainiceanu, C. M. Measurement error in nonlinear models: A modern perspective, second edition. *Measurement Error in Nonlinear Models: A Modern Perspective, Second Edition* 1–455 (2006).

55. Vörös, Z. *et al.* Probability density functions for the variable solar wind near the solar cycle minimum. *J. Geophys. Res. Space Phys.* **120**, 6152–6166 (2015).

56. Borovsky, J. E. Noise and Solar-Wind/Magnetosphere Coupling Studies: Data. *Frontiers in Astronomy and Space Sciences* **0**, (2022).

5725. Reiff, P. H., Spiro, R. W. & Hill, T. W. Dependence of polarcap potential drop on interplanetary parameters. *J. Geophys. Res.* **86**, 7639–7648 (1981).

5826. Wygant, J. R., Torbert, R. B. & Mozer, F. S. Comparison of S3-3 polar cap potential drops with the interplanetary magnetic field and models of magnetopause reconnection. *J. Geophys. Res. Space Phys.* **88**, 5727–5735 (1983).

5927. Weimer, D. R., Reinleitner, L. A., Kan, J. R., Zhu, L. & Akasofu, S.-I. Saturation of the auroral electrojet current and the polar cap potential. *J. Geophys. Res. Space Phys.* **95**, 18981–18987 (1990).

6028. Russell, C. T., Luhmann, J. G. & Lu, G. Nonlinear response of the polar ionosphere to large values of the interplanetary electric field. *J. Geophys. Res. Space Phys.* **106**, 18495–18504 (2001).

6129. Liemohn, M. W. & Ridley, A. J. Comment on 'Nonlinear response of the polar ionosphere to large values of the interplanetary electric field' by C. T. Russell et al. *Journal of Geophysical Research: Space Physics* vol. 107 SIA 13-1 Preprint at https://doi.org/10.1029/2002JA009440 (2002).

~~62~~30. Shepherd, S. G., Greenwald, R. A. & Ruohoniemi, J. M. Cross polar cap potentials measured with Super Dual Auroral Radar Network during quasi-steady solar wind and interplanetary magnetic field conditions. *J. Geophys. Res. Space Phys.* **107**, SMP 5-1 (2002).

~~63~~31. Nagatsuma, T. Saturation of polar cap potential by intense solar wind electric fields. *Geophys. Res. Lett.* **29**, 62–1 (2002).

~~64~~32. Hairston, M. R., Drake, K. A. & Skoug, R. Saturation of the ionospheric polar cap potential during the October–November 2003 superstorms. *J. Geophys. Res. Space Phys.* **110**, 9–26 (2005).

~~65~~33. Hill, T. W., Dessler, A. J. & Wolf, R. A. Mercury and Mars: The role of ionospheric conductivity in the acceleration of magnetospheric particles. *Geophys. Res. Lett.* **3**, 429–432 (1976).

~~66~~34. Siscoe, G. L. *et al.* Hill model of transpolar potential saturation: Comparisons with MHD simulations. *J. Geophys. Res. Space Phys.* **107**, (2002).

~~67~~35. Merkin, V. G. *et al.* Global MHD simulations of the strongly driven magnetosphere: Modeling of the transpolar potential saturation. *J. Geophys. Res. Space Phys.* **110**, (2005).

~~68~~36. Merkin, V. G. *et al.* Relationship between the ionospheric conductance, field aligned current, and magnetopause geometry: Global MHD simulations. *Planet. Space Sci.* **53**, 873–879 (2005).

~~69~~37. Raeder, J. & Lu, G. Polar cap potential saturation during large geomagnetic storms. *Advances in Space Research* **36**, 1804–1808 (2005).

~~70~~38. Ridley, A. J. A new formulation for the ionospheric cross polar cap potential including saturation effects. *Ann. Geophys.* **23**, 3533–3547 (2005).

~~71~~39. Lopez, R. E. *et al.* Role of magnetosheath force balance in regulating the dayside reconnection potential. *J. Geophys. Res. Space Phys.* **115**, 12216 (2010).

~~72~~40. Pudovkin, M. I., Zaitseva, S. A., Bazhenova, T. A. & Andrezen, V. G. Electric fields and currents in the earth's polar caps. *Planet. Space Sci.* **33**, 407–414 (1985).

~~73~~41. Raeder, J., Wang, Y. L., Fuller-Rowell, T. J. & Singer, H. J. Global Simulation of Magnetospheric Space Weather Effects of the Bastille Day Storm. *Solar Physics 2001 204:1* **204**, 323–337 (2001).

~~74~~42. Hernandez, S., Lopez, R. E., Wiltberger, M. & Lyon, J. G. MHD Simulations of Solar Wind-Geospace Coupling. in *AGU Spring Meeting Abstracts* SM51C-03 (SAO/NASA Astrophysics Data System, 2007).

~~75~~43. Winglee, R. M., Chua, D., Brittnacher, M., Parks, G. K. & Lu, G. Global impact of ionospheric outflows on the dynamics of the magnetosphere and cross-polar cap potential. *J. Geophys. Res.* **107**, (2002).

~~76~~44. Siscoe, G. L., Crooker, N. U. & Siebert, K. D. Transpolar potential saturation: Roles of region 1 current system and solar wind ram pressure. *J. Geophys. Res. Space Phys.* **107**, (2002).

~~77~~45. Ridley, A. J. Alfvén wings at Earth's magnetosphere under strong interplanetary magnetic fields. *Ann. Geophys.* **25**, 533–542 (2007).

~~78~~46. Kivelson, M. G. & Ridley, A. J. Saturation of the polar cap potential: Inference from Alfvén wing arguments. *J. Geophys. Res. Space Phys.* **113**, 5214 (2008).

47. Kan, J. R. & Lee, L. C. Energy coupling function and solar wind-magnetosphere dynamo. *Geophys. Res. Lett.* **6**, 577–580 (1979).

48. Troshichev, O. & Sormakov, D. PC index as a proxy of the solar wind energy that entered into the magnetosphere: 2. Relation to the interplanetary electric field e KL before substorm onset. *Earth, Planets and Space* **67**, 1–11 (2015).

49. Stauning, P. The polar cap (PC) index combination, PCC: Relations to solar wind properties and global magnetic disturbances. *Journal of Space Weather and Space Climate* **11**, 19 (2021).

50. Stauning, P. The Polar Cap PC Indices: Relations to Solar Wind and Global Disturbances. in *Exploring the Solar Wind* (InTech, 2012). doi:10.5772/37359.

51. Lockwood, M., Cowley, S. W. H. & Freeman, M. P. The excitation of plasma convection in the high-latitude ionosphere. *J. Geophys. Res. Space Phys.* **95**, 7961–7972 (1990).

52. King, J. H. & Papitashvili, N. E. Solar wind spatial scales in and comparisons of hourly Wind and ACE plasma and magnetic field data. *J. Geophys. Res. Space Phys.* **110**, 2104 (2005).

53. Weimer, D. R. *et al.* Predicting interplanetary magnetic field (IMF) propagation delay times using the minimum variance technique. *J. Geophys. Res. Space Phys.* **108**, 1026 (2003).

54. Haaland, S., Paschmann, G. & Sonnerup, B. U. Ö. Comment on “A new interpretation of Weimer et al.’s solar wind propagation delay technique” by Bargatze et al. *J. Geophys. Res. Space Phys.* **111**, 6102 (2006).

55. Knetter, T., Neubauer, F. M., Horbury, T. & Balogh, A. Four-point discontinuity observations using Cluster magnetic field data: A statistical survey. *J. Geophys. Res. Space Phys.* **109**, 6102 (2004).

56. Troshichev, O. A., Janzhura, A. S. & Stauning, P. Magnetic activity in the polar caps: Relation to sudden changes in the solar wind dynamic pressure. *J. Geophys. Res. Space Phys.* **112**, 11202 (2007).

57. Laundal, K. M. *et al.* Time-scale dependence of solar wind-based regression models of ionospheric electrodynamics. *Scientific Reports 2020 10:1* **10**, 1–9 (2020).

58. Case, N. A. & Wild, J. A. A statistical comparison of solar wind propagation delays derived from multispacecraft techniques. *J. Geophys. Res. Space Phys.* **117**, 2101 (2012).

59. Nguyen, G. *et al.* Massive Multi-Mission Statistical Study and Analytical Modeling of the Earth’s Magnetopause: 1. A Gradient Boosting Based Automatic Detection of Near-Earth Regions. *J. Geophys. Res. Space Phys.* **127**, (2022).

60. Michotte de Welle, B. *et al.* Global Environmental Constraints on Magnetic Reconnection at the Magnetopause From In Situ Measurements. *J. Geophys. Res. Space Phys.* **129**, e2023JA032098 (2024).

61. Gjerloev, J. W. The SuperMAG data processing technique. *J. Geophys. Res. Space Phys.* **117**, 9213 (2012).

62. Newell, P. T. & Gjerloev, J. W. Evaluation of SuperMAG auroral electrojet indices as indicators of substorms and auroral power. *J. Geophys. Res. Space Phys.* **116**, (2011).

63. Lepping, R. P. *et al.* The WIND magnetic field investigation. *Space Science Reviews 1995 71:1* **71**, 207–229 (1995).

64. Sivadas, N. *et al.* Simultaneous measurements of solar wind from L1, shocked solar wind from Magnetosheath, and measures of geomagnetic response from ground-based indices . Preprint at https://doi.org/10.5281/zenodo.17546718 (2026).

65. Sivadas, N. Code for Regression to the Mean of Extreme Geomagnetic Storms . Preprint at https://doi.org/10.5281/zenodo. 17559999 (2026).

66. Dungey, J. W. Interplanetary Magnetic Field and the Auroral Zones. *Phys. Rev. Lett.* **6**, 47 (1961).

67. Cowley, S. ~W. ~H. & Lockwood, M. Excitation and decay of solar wind-driven flows in the magnetosphere-ionosphere system. *Ann. Geophys.* **10**, 103–115 (1992).

68. Lockwood, M. Solar Wind—Magnetosphere Coupling Functions: Pitfalls, Limitations, and Applications. *Space Weather* **20**, e2021SW002989 (2022).

69. Troshichev, O. A. & Andrezen, V. G. The relationship between interplanetary quantities and magnetic activity in the southern polar cap. *Planet. Space Sci.* **33**, (1985).

70. Dunham, D. ~W. ISEE-3, the first libration-point satellite. in *Bulletin of the American Astronomical Society* vol. 11 805–806 (1979).

71. Bonilla, M. G., Mark, R. K. & Lienkaemper, J. J. Statistical relations among earthquake magnitude, surface rupture length, and surface fault displacement. *Bulletin of the Seismological Society of America* **74**, 2379–2411 (1984).

72. Borovsky, J. E. On the Saturation (or Not) of Geomagnetic Indices. *Frontiers in Astronomy and Space Sciences* **8**, 175 (2021).

73. Pulkkinen, T. I. *et al.* Magnetosheath control of solar wind-magnetosphere coupling efficiency. *J. Geophys. Res. Space Phys.* **121**, 8728–8739 (2016).

74. Doyle, M. A. & Burke, W. J. S3-2 measurements of the polar cap potential. *J. Geophys. Res. Space Phys.* **88**, 9125–9133 (1983).

75. Barnett, A. G., van der Pols, J. C. & Dobson, A. J. Regression to the mean: what it is and how to deal with it. *Int. J. Epidemiol.* **34**, 215–220 (2005).

76. Carroll, R. J., Ruppert, D., Stefanski, L. A. & Crainiceanu, C. M. Measurement error in nonlinear models: A modern perspective, second edition. *Measurement Error in Nonlinear Models: A Modern Perspective, Second Edition* 1–455 (2006).

77. Vörös, Z. *et al.* Probability density functions for the variable solar wind near the solar cycle minimum. *J. Geophys. Res. Space Phys.* **120**, 6152–6166 (2015).

78. Borovsky, J. E. Noise and Solar-Wind/Magnetosphere Coupling Studies: Data. *Frontiers in Astronomy and Space Sciences* **0**, (2022).

Funding:

NASA Heliophysics Guest Investigator Program under Grant # 80NSSC23K0446 (NS,BF)

NSF AGS GEM Award #2453576 (NS, DSO)

UKRI STFC through the Ernest Rutherford Fellowship (ST/X003663/1) (MTW)

NASA Heliophysics Participating Investigator Program under Grant #WBS516741.01.24.01.03 (DS)

Author contributions:

Conceptualization: NS, VS
Methodology: NS
Investigation: NS, VS, DSO, BF
Formal Analysis: NS, VS
Visualization: NS
Software: NS, BMW
Validation: NS, MTW
Funding acquisition: NS, DS, DSO, BF
Project administration: NS, DS
Supervision: DS
Writing – original draft: NS, VS
Writing – review & editing: NS, VS, DS, MTW, BMW, DSO, BF

Competing interests: Authors declare that they have no competing interests.

Materials & Correspondence: Nithin Sivadas, nithinsivadas90@gmail.com

Supplementary Materials

Supplementary Information

1. Supplementary Discussion
   a. Physics of solar-wind magnetosphere coupling
   b. An example of the problem of definition
   c. Random error in the independent variable does not average away
   ~~a.~~d. Regression to the mean as a fundamental property
   e. Regression to the mean from different vantage points
   f. Regression bias due to magnitude and time uncertainty
   g. Interpretation of the Error Model output
   h. A different measure of geomagnetic response is also linear
   ~~b.~~i. Historical development of the polar cap potential saturation problem
   ~~c.~~j. Evidence in literature

~~d.~~a. ~~Regression to the mean from different vantage points~~

~~e. Regression to the mean as a fundamental property of the relation between truth and measurement~~

2. Supplementary Methods

a. Kan-Lee electric field is an approximation of the true solar wind driver

~~a.~~b. Validation of the Error Model

~~b.~~c. Analytical derivation of non-linear regression bias due to uncertainty in time

~~c.~~d. Summary of data analysis procedure in the computer code

Extended Data Figures 1 to 5

Extended Data Table 1 to 2

SUPPLEMENTARY INFORMATION

1. Supplementary Discussion

a. Physics of solar-wind magnetosphere coupling

The Earth's magnetic field carves out a cavity in the oncoming solar wind, called the magnetosphere. It mostly keeps out the plasma and magnetic field associated with the solar wind except under specific local magnetic field conditions that lead to the reconnection of solar wind and magnetospheric magnetic field lines. Upstream from the reconnection-site, the supersonic and superalfvénic solar wind slows down through a bowshock, forming the magnetosheath just upstream from the magnetosphere's outer boundary—the magnetopause. When the shocked solar wind magnetic fields lie anti-parallel to those of the Earth, the field can undergo a topological change that opens up the Earth's magnetic field lines and reconnects them to the shocked solar wind magnetic field lines[66]. Through reconnection, the solar wind plasma and electric field can enter the Earth's magnetosphere and drive plasma convection in the magnetosphere. Conveyed to the polar ionosphere closer to the Earth, this convection electric field applies an electric potential across the polar cap on average[67] (See Figure 1a). The projection of the solar wind convection electric field along the dawn-dusk direction in the polar cap is the Kan-Lee or merging electric field (E_m^*)[47], which we refer to as solar wind driving[68].

In the 1970s, estimates of the cross-polar cap potential (and its corresponding dawn-dusk electric field) were made from single ground magnetometers situated in the northern and southern polar regions. The argument was that the perturbations of the magnetic field measured by the polar magnetometers give an excellent continuous estimate of the solar wind driving, as the polar regions magnetically connect to the solar wind[69]. Continuous estimates of solar wind driving from satellite missions were not yet available at the time[70]. As the magnetized plasma in the solar wind and magnetosphere is collisionless, the resistance along magnetic field lines is minuscule. Hence, the magnetohydrodynamic theory is valid, ensuring that plasma along an entire field-line moves as one[66]. Therefore, the solar wind electric field, resulting from the motion of the wind, at one end of the magnetic field line, would map along the field line to the polar ionosphere. This electric field then drives currents in the ionosphere that cause magnetic perturbations measured from ground magnetometers. Supporting this theory, the cross polar cap index (PCI), a measure of geomagnetic activity, increases linearly on average with the merging electric field (E_m^*), a measure of the solar wind driving[6]. The PCI is estimated by a polar magnetometer from the ground in each hemisphere and is proportional to the cross polar cap potential[7,8], while E_m^* is measured from a spacecraft in the solar wind far upstream of the Earth. As more measurements of rare and extreme solar wind driving accumulated through the years, surprisingly, the correlation between the geomagnetic response and the solar wind driver began to deviate from the linear relation. There appeared to be an upper limit to the cross-polar cap potential beyond which increasing solar wind driving led to no increase in the potential (See Figure 1b green curve).

The new inference that the cross-polar cap potential saturates with increasing solar wind driving from later measurements led to the emergence of 10 different but sometimes overlapping theories and models attempting to explain the phenomenon (See Extended Data Table 1)[9,10]. The theories fall into two categories, one arguing that the conditions at the solar wind/magnetosphere interaction region (i.e., the dayside magnetosheath) during extreme solar wind driving lead to a consistent

diminishing of the rate of reconnection or energy coupling from the solar wind to the magnetosphere. The other set of theories argues that processes within the magnetosphere slow down ionospheric plasma convection during extreme solar wind driving. All theories suggest that the energy transferred into the polar ionosphere during extreme space weather has, in essence, an upper limit. Hence, the theories imply that the Earth's magnetosphere shields us from extreme geomagnetic storms by inhibiting energy transfer from the strong solar wind to the ionosphere, one way or the other.

In this paper, we present a radically different proposition. We argue that there is no statistical evidence for the saturation of geomagnetic response to strong solar wind driving, and its appearance in measurements is a result of uncertainty in solar wind driver measurements. We correct for the effect of this uncertainty and demonstrate that the geomagnetic response varies linearly with solar wind driving, implying that the energy transfer from extreme solar wind conditions to the polar ionosphere can be far greater than currently believed. The importance of this work goes beyond space science to any correlation study between a measure of a system's input and its response, revealing a larger response for extreme input values.

b. An example of the problem of definition

A carpenter might measure the height of a door frame using a measuring tape. However, the door she creates using this height will likely not fit the frame. Even though the least count of the tape is small and does not contribute much to the uncertainty, there is uncertainty due to an implicit assumption that the height of the door is a one-dimensional quantity when it is in reality at least a two-dimensional quantity, i.e., there are multiple different heights for the door and the door frame, varying across its breadth and width. Hence, the experienced carpenter would use the carpenter's square to ensure that the edges of the door are perpendicular to the length, to match the door frame, and reduce this uncertainty.

c. Random error in the independent variable does not average away

A popular technique to find the relations between two or more physical processes is regression analysis of their measurements. The essence of regression analysis is to find the average of the measurement Y^* when the measurement X^* is a specific value 'x'. This is equivalent to the conditional expectation of Y^* given $X^* = x$, mathematically represented as $\langle Y^* | X^* = x \rangle$. Here, Y^* is the dependent variable and X^* is the independent or conditional variable. The averaging of Y^* indeed removes the effect of random error in Y^*, consistent with the widely held belief. But, crucially, the conditional or independent variable X^* cannot be averaged while simultaneously averaging the dependent variable Y^* for a given value of X^*. In other words, the random error in the conditional variable remains unaddressed and is not averaged away[15]. And, surprisingly, this unbiased random error in the conditional variable (X^*) manifests as a bias in the relation inferred between the variables Y^* and X^*. This *regression bias* is such that the average value of Y^* for a given X^* will be closer to the mean of Y, and if the regression bias is non-linear, it can create an appearance of saturation in Y^* for increasing X^*. This statistical phenomenon is a result of a *regression to the mean*[16,17].

~~a. Historical development of the polar cap potential saturation problem~~

~~The regression to the mean of extreme solar wind driver values can sometimes be confused with under-sampling bias. Under-sampling is a statistical effect entirely distinct from the regression to~~

the mean effect. The under-sampling bias reduces with increasing samples, however, the regression to the mean remains the same even with infinite samples. It is easy to see this from the analytical derivation in the methods section (See Equation 1.21), which is immune to any sampling bias. However, under-sampling has had an important impact on the development of the polar cap saturation problem[21].

When examining space science literature on this problem, we observe from the 1980s to the 2000s (See Extended Data Figure 5a-b) that as more and more solar wind data accumulates, the polar cap potential's saturation limit trends upwards from ~0.5 mV/m to ~10 mV/m. As extreme values of solar wind drivers are rare, their samples in data analysis can be poor. The number of samples increases with time, and hence the bias of under-sampling also diminishes with time. As shown by Extended Data Figure 5b, due to this bias, observations suggest different relations between solar wind driving and polar cap potential, with different saturation limits and even no saturation (i.e., Boyle et al., 1997[51]). Since inferences from data do not agree with each other, the developers of a theory of saturation are also confounded by the question of which observational study they ought to explain. Such a diversity of inferences from data itself confuses the development of theoretical explanations. Hence, it is not surprising that multiple theories have emerged over the years (See Extended Data Table 1) without a unifying theory explaining the saturation effect quantitatively, as there are multiple different quantitative relations between solar wind driving and polar cap potential depending on the study.

Our work, that carefully addresses uncertainties in the driver with a sufficiently large database of 25 years of measurements, brings a resolution to the contradiction between the different inferences and shows that the evidence overwhelmingly concludes that the relation between the driver and the response is linear. It is this linear relationship between the driver and response that a new (or old) physical theory or model needs to explain and validate their predictions against.

b.a. Evidence in Literature

Some previous data-based studies like Boyle et al. (1997)[51] have found that solar wind driving varies linearly with cross-polar cap potential using spacecraft measurements after a meticulous filtering of data. Pulkkinen et al. 2016[52] found that on average, the auroral electrojet index varies linearly with the shocked solar wind electric field in the magnetosheath tangential to the magnetopause (a proxy for the local reconnection electric field). Additionally, they found that this magnetosheath electric field varies non-linearly (or saturates) with the solar wind electric field. Their findings are entirely consistent with our explanation that the variance of solar wind parameters relative to the local magnetosheath values is the source of the saturation effect and not some magnetosheath phenomenon. The increased variance observable within their own data suggests that the variance in the driver leads to the saturation, and not any physical effect that causes a consistent reduction in the local magnetosheath electric field as they suggest. Borovsky 2021[30] found that the ionospheric response of PCI and SML indices varies linearly with solar wind driving as quantified by the $S_{(1)(9b)}$ index. The $S_{(1)(9b)}$ index is developed using the method of canonical correlation analysis applied to a set of solar wind parameters and another set of geomagnetic responses. From many parameters in both sets, the method extracts a small number of canonical variate pairs that contain the majority of shared information, relegating the uncertainty (and variance) that causes a lack of correlation to an orthogonal variate pair. As we have shown, since the uncertainty leads to the saturation effect, by reducing the uncertainty in the new driver $S_{(1)(9b)}$, Borovsky 2021 implicitly removed the non-linear regression bias in the relation between

~~$S_{(1)(9b)}$ and geomagnetic response parameters like PCI and SML index. Hence, they have a linear relationship through the entire range of the solar wind driver $S_{(1)(9b)}$, consistent with our result.~~

~~c.~~a.~~Regression to the mean from different vantage points~~

~~There are five vantage points from which we explain the regression to the mean effect.~~

~~**Vantage point 1: Regression bias.** The average value of a dependent variable given the independent variable will be biased closer to the mean value when there is some measurement uncertainty in the independent variable.~~

~~**Vantage point 2: Repeat measurements.** Extreme measurements are more likely followed by measurements closer to the mean. This is the most common understanding of the regression to the mean effect.~~

~~**Vantage point 3: Likelihood of Stochastic Processes.** Any physical process has an underlying probability distribution; therefore, some values of the physical process are more likely than others. For a stochastic process with a Gaussian distribution, the mean value coincides with the most likely or probable value. As a result, any uncertain measurement of the stochastic process is more likely measuring the true value closer to the mean of the stochastic process than more extreme values, by the simple fact that values closer to the mean are more common and hence more likely than the extreme values. In other words, the true value being measured is more likely to be closer to the mean of the stochastic process than the measurement.~~

~~**Vantage point 4: Probability Theory.** Figure 2a geometrically shows that for a Gaussian process, the true value is likely closer to the mean than the measurement. The black line is the probability distribution of a Gaussian process, X. When this process is measured with some uncertainty, the measurement can take the values in the likelihood quantified by the dashed-blue curve when the true value is X=2 and the dashed-red curve when it is X=4. Since the true value is generally inaccessible, the goal of a measurement is to estimate the most probable true value corresponding to the measurement. Hence, when the measurement X*=3 (solid black vertical line), the probability that the corresponding true value X is also equal to 3 (black dot) is remarkably *less likely* than it being equal to 2 (blue dot), simply because X=3 is less likely to occur than X=2 for a Gaussian process with zero mean.~~

~~**Vantage point 5: Relation between truth and measurement.** From vantage point 4, and consistent with other three vantage points, we can infer that regression to the mean is a property of the relation between truth and measurement, the effects of which are observable in statistical methods, and can be examined through probability theory. This new vantage point is the general result presented in this manuscript, further explanation is provided in Supplementary Discussion 1.d. The fact that regression to the mean is not only a statistical effect, but also a property of the relation between truth and measurement, imply that the effect is present even where a single measurement is made, and hence is likely ubiquitous.~~

d. Regression to the mean as a fundamental property of the relation between truth and measurement

By fundamental property we mean a property that exists in reality independent of the methodology we use to study it, as opposed to an effect that arises from the methodology. For example, an undersampling bias is an effect that arises purely from the number of samples we use in our

analysis. All methods that use infinite samples will never know of the undersampling bias. We argue that the regression to the mean effect, on the contrary, exists in reality as a property of the relation between truth and measurement independent of the methodology we use.

The first evidence for this comes from the fact that the phenomenon is observable in multiple statistical methods that use populations with inherent variability (e.g., different types of regression analysis, repeat measurements, machine learning, estimating conditional probability distributions), and also observable through probability theory when inferring the truth corresponding to the measurement. Supplementary Discussion 1.e explains regression to the mean effect from different vantage points. The second evidence is the mathematical result that can be derived using probability theory, graphically illustrated using Figure 2a, which shows that the effect only depends on the nature of the truth and measurement i.e., their likelihood of occurrence and their uncertainty. This implies that even when one makes a single measurement, the regression to the mean effect leads to a bias in the true value corresponding to the measurement according to the stochastic properties of the phenomenon being measured and the uncertainty in the measurement.

Here, an example of a familiar but different fundamental property of relations between things, e.g., the electric field, might be instructive and help us see the features of a fundamental property. An electric field is a fundamental property of the relationship between two or more charges. This relation, like all relations, has a qualitive aspect and a quantitative aspect. The quality of electric field is that it displaces the two charges relative to each other. The quantitative aspect is that the displacement depends on the nature of both the charges, its sign, magnitude, and distance between them. It was demonstrated to exist through experiments conducted by Faraday, but that does not mean it is only an experimental effect. It was proven to be mathematically consistent by Maxwell, but that does not mean it is only a mathematical effect or “field-theoretic” effect. This relationship between charges exists in reality, irrespective of the tools we use to observe it.

Similarly, when we say regression to the mean or the more-probable, is a fundamental property of the relationship between truth and measurements, it also implies, this relation, like all relations, has a qualitative and quantitative aspect. The quality of this property is that the truth is separated from the measurement by some measure. The distance of separation depends on the nature of the truth and the measurement, i.e., the stochastic properties of the truth, the degree of uncertainty of the measurement, and how it changes with time, space and magnitude of the truth. It can be demonstrated to exist through experiments with a large number of measurements (i.e., statistical methods), but that does not mean it is only a statistical effect. It can be proven to be mathematically consistent using probability theory (Figure 2a), but that does not mean it is only a probabilistic effect. This relationship between truth and measurement exists in reality, irrespective of the tools we use to observe this relationship.

The existence of this relation between truth and measurement arises only because different from each other. Here we provide a philosophical insight into why that is the case. In our universe, truth is generally only accessible through measurements - this is because the universe is a complex system. Complex systems have many processes, that the most prominent process will have a systematic effect that we can observe, which we may call a “law”, or we infer as the physical process of our interest. However, on the flip side, it will have other processes that appear as random perturbations, which may not be of our interest, and hence appear as randomness. This forces a

separation between truth and measurements, leading them to be both different things. Truth is not measurement, and measurement is not the truth. However, there is an inseparable connection between them. Truth cannot be accessed without measurements, and measurements have no independent existence without the truth. This firmly establishes the existence of at least one, if not many, relationships between truth and measurement. One such relation is the regression to the mean of the truth, where the randomness manifests as uncertainty in measurements, and stochastic variations in the truth, leading to a separation of the truth from measurement in quantifiable ways.

~~2. Supplementary Methods~~

e. Regression to the mean from different vantage points

There are five vantage points from which we explain the regression to the mean effect.

Vantage point 1: Regression bias. The average value of a dependent variable given the independent variable will be biased closer to the mean value when there is some measurement uncertainty in the independent variable. Explained in Supplementary Discussion 1.c.

Vantage point 2: Repeat measurements. Extreme measurements are more likely followed by measurements closer to the mean. This is the most common understanding of the regression to the mean effect.

Vantage point 3: Likelihood of Stochastic Processes. Any physical process has an underlying probability distribution; therefore, some values of the physical process are more likely than others. For a stochastic process with a Gaussian distribution, the mean value coincides with the most likely or probable value. As a result, any uncertain measurement of the stochastic process is more likely measuring the true value closer to the mean of the stochastic process than more extreme values, by the simple fact that values closer to the mean are more common and hence more likely than the extreme values. In other words, the true value being measured is more likely to be closer to the mean of the stochastic process than the measurement.

Vantage point 4: Probability Theory. Figure 2a geometrically shows that for a Gaussian process, the true value is likely closer to the mean than the measurement. The black line is the probability distribution of a Gaussian process, X. When this process is measured with some uncertainty, the measurement can take the values in the likelihood quantified by the dashed-blue curve when the true value is X=2 and the dashed-red curve when it is X=4. Since the true value is generally inaccessible, the goal of a measurement is to estimate the most probable true value corresponding to the measurement. Hence, when the measurement X*=3 (solid black vertical line), the probability that the corresponding true value X is also equal to 3 (black dot) is remarkably *less likely* than it being equal to 2 (blue dot), simply because X=3 is less likely to occur than X=2 for a Gaussian process with zero mean.

Vantage point 5: Relation between truth and measurement. From vantage point 4, and consistent with other three vantage points, we can infer that regression to the mean is a property of the relation between truth and measurement, the effects of which are observable in statistical methods, and can be examined through probability theory. This new vantage point is the general result presented in this manuscript, further explanation is provided in Supplementary Discussion 1.d. The fact that regression to the mean is not only a statistical effect, but also a property of the relation between truth and measurement, imply that the effect is present even where a single measurement is made, and hence is likely ubiquitous.

f. Regression bias due to magnitude and time uncertainty

As shown by Sivadas & Sibeck, 2022[16] using a simple Monte-Carlo error model, if X has a Gaussian distribution, and the error ϵ is also Gaussian and independent of X like most instrumental errors (i.e., homoskedastic error[19]), then the likely true value for a given measurement (i.e., the average of X given X^* or $\langle X|X^*\rangle$) will have a linear bias. The purple curve in Figure 2b shows this linear bias as a lower slope than the dashed-black line of equality representing the curve if there

were no uncertainty (i.e., the true unbiased relationship). However, suppose the error ϵ depends on X, and varies with the magnitude of X (i.e., heteroskedastic error[19]) like some uncertainties related to the 'problem of definition', and X has a log-normal distribution like many solar wind parameters[20]. In that case, the key point is that the likely true value for a given measurement will have a larger and non-linear bias that mimics a *saturation* of X with increasing X^*. Figure 2c demonstrates this in the purple curve whose slope has a non-linear bias with respect to the black-dashed line of equality representing the true unbiased relationship. Supposing that X is the true solar wind driver near the Earth, and X^* is the uncertain measurement far from Earth, the response Y of the Earth will be driven by the true driver X and, crucially, not by the measurement X^*. If the response Y is linearly related to X (i.e., $Y \propto X$), then naturally, the response Y will also appear to saturate with increasing value of the measurement X^*.

Furthermore, if we keep aside the magnitude uncertainty (i.e., set $\epsilon = 0$), and examine the effect of uncertainty in time dt, we observe that it will lead to a non-linear bias in the average of X given X^* as well, with increasing deviation from linearity with growing uncertainty in time defined by the ratio of the temporal uncertainty (δ) and the autocorrelation time constant (k) of the stochastic process. Figure 3c shows quantitatively how the average of the true value X given the measurement X^* has a non-linear bias in its slope with respect to the dashed-black line of equality representing the true unbiased relationship. This non-linear bias increases with increasing ratio of δ/k, a dimensionless measure of the uncertainty in time. In the Methods section[18], we derive analytically from first principles this non-linear bias and its dependence on temporal uncertainty, which will be relevant for any study with measurement of systems that have a delay in their drivers or response, due to propagation of information, or dynamics of the system e.g., seismic waves from earthquakes[71], or response of medical patients to a specific back pain treatment[23].

g. Interpretation of the Error Model output

Once the calculated uncertainty values are worked into the error model, and X is assumed to have a log-normal distribution with an autocorrelation coefficient of ~100 minutes observed in solar wind measurements, the Monte-Carlo error model solves for X^* and predicts an appearance of saturation of X given X^* (See Figure 3b). This saturated curve, and the conditional probability distribution of X given X^*, matches surprisingly well with the saturation that appears in the data for the polar cap index PCI given the merging electric field estimated from solar wind measurements E_m^* (See Figure 3a). The solution of the error model is consistent with data as viewed through both the conditional expectation $\langle PCI|E_m^* \rangle$ and the conditional probability distribution of $pdf(PCI|E_m^*)$ (See Figure 3a-b). Further validation of the error model with second order statistics of data is presented in the Methods section[18]. Here PCI is analogous to Y in the error model, E_m^* is analogous to X^*, and E_m^{sh} analogous to X. The similarity in the curves $\langle PCI|E_m^* \rangle$ and $\langle X|X^* \rangle$, indicate that the geomagnetic response is proportional to the true shocked solar wind driver i.e., $PCI \propto E_m^{sh}$ and equivalently $Y \propto X$. The precondition of this non-linear saturation relation between PCI and E_m^* is the heteroskedastic nature of the error in E_m^*, the propagation time uncertainty, and the fact that the solar wind driver is a log-normal process with a particular autocorrelation time constant.

h. A different measure of geomagnetic response is also linear

Our finding that uncertainties in the solar wind driver results in the saturation of the polar cap index, raises the possibility that other measures of the geomagnetic response previously reported

to saturate (or not)[72] based on uncertain solar wind driver estimates may be incorrect. The SML index, which is the westward auroral electrojet strength, measured by completely different magnetometers located at high latitudes ($\sim 65°N$) around the planet, also appears to saturate with erroneous solar wind driving estimates. However, the corrected solar wind driver values, calibrated exactly the same way as above, show a linear relationship with the westward auroral electrojet strength as well—a further confirmation that the high-latitude geomagnetic response does not saturate with solar wind driving, once we remove the effect of uncertainties. Figure 4b shows the linear relation of the SML index with corrected solar wind driver in the purple curve, once the regression calibration is applied to the erroneous data in the saturating green curve.

i. Historical development of the polar cap potential saturation problem

The regression to the mean of extreme solar wind driver values can sometimes be confused with under-sampling bias. Under-sampling is a statistical effect entirely distinct from the regression to the mean effect. The under-sampling bias reduces with increasing samples, however, the regression to the mean remains the same even with infinite samples. It is easy to see this from the analytical derivation in the methods section (See Equation 1.21), which is immune to any sampling bias. However, under-sampling has had an important impact on the development of the polar cap saturation problem[21].

When examining space science literature on this problem, we observe from the 1980s to the 2000s (See Extended Data Figure 5a-b) that as more and more solar wind data accumulates, the polar cap potential's saturation limit trends upwards from ~0.5 mV/m to ~10 mV/m. As extreme values of solar wind drivers are rare, their samples in data analysis can be poor. The number of samples increases with time, and hence the bias of under-sampling also diminishes with time. As shown by Extended Data Figure 5b, due to this bias, observations suggest different relations between solar wind driving and polar cap potential, with different saturation limits and even no saturation (i.e., Boyle et al., 1997[24]). Since inferences from data do not agree with each other, the developers of a theory of saturation are also confounded by the question of which observational study they ought to explain. Such a diversity of inferences from data itself confuses the development of theoretical explanations. Hence, it is not surprising that multiple theories have emerged over the years (See Extended Data Table 1) without a unifying theory explaining the saturation effect quantitatively, as there are multiple different quantitative relations between solar wind driving and polar cap potential depending on the study.

Our work, that carefully addresses uncertainties in the driver with a sufficiently large database of 25 years of measurements, brings a resolution to the contradiction between the different inferences and shows that the evidence overwhelmingly concludes that the relation between the driver and the response is linear. It is this linear relationship between the driver and response that a new (or old) physical theory or model needs to explain and validate their predictions against.

j. Evidence in Literature

Some previous data-based studies like Boyle et al. (1997)[24] have found that solar wind driving varies linearly with cross-polar cap potential using spacecraft measurements after a meticulous filtering of data. Pulkkinen et al. 2016[73] found that on average, the auroral electrojet index varies linearly with the shocked solar wind electric field in the magnetosheath tangential to the magnetopause (a proxy for the local reconnection electric field). Additionally, they found that this magnetosheath electric field varies non-linearly (or saturates) with the solar wind electric field.

Their findings are entirely consistent with our explanation that the variance of solar wind parameters relative to the local magnetosheath values is the source of the saturation effect and not some magnetosheath phenomenon. The increased variance observable within their own data suggests that the variance in the driver leads to the saturation, and not any physical effect that causes a consistent reduction in the local magnetosheath electric field as they suggest. Borovsky 2021[72] found that the ionospheric response of PCI and SML indices varies linearly with solar wind driving as quantified by the $S_{(1)(9b)}$ index. The $S_{(1)(9b)}$ index is developed using the method of canonical correlation analysis applied to a set of solar wind parameters and another set of geomagnetic responses. From many parameters in both sets, the method extracts a small number of canonical variate pairs that contain the majority of shared information, relegating the uncertainty (and variance) that causes a lack of correlation to an orthogonal variate pair. As we have shown, since the uncertainty leads to the saturation effect, by reducing the uncertainty in the new driver $S_{(1)(9b)}$, Borovsky 2021 implicitly removed the non-linear regression bias in the relation between $S_{(1)(9b)}$ and geomagnetic response parameters like PCI and SML index. Hence, they have a linear relationship through the entire range of the solar wind driver $S_{(1)(9b)}$, consistent with our result.

2. Supplementary Methods

a. Kan-Lee electric field is an approximation of the true solar wind driver

The Kan-Lee electric field is an approximation of the component of the reconnection electric field ($E_R = V_{sh}B_{sh}\sin(\theta'/2)$) perpendicular to the geomagnetic field that creates the cross-polar cap electric field (which is equal to $V_{sh}B_{sh}\sin^2(\theta'/2)$ due to a geometrical argument presented first by Kan and Lee)[47]. The approximation assumes that the shear angle between the magnetosheath and magnetospheric magnetic field θ' is equal to θ_{sw}. Furthermore, the approximation assumes $V_{sh}B_{sh} \cong V_{sw}B_{T,sw}$, which is true most of the time 1) as the solar wind velocity component tangential to the bowshock is negligible compared to the normal component, and 2) as the tangential component of the electric field across a normal MHD shock is conserved. This allows the Kan-Lee electric field (E_m^*) to estimate the cross polar electric field E_{PC} with just solar wind parameters. Our examination of data shows that for electric field values <3mV/m, which is 95% of all available data points in 25 years, the Kan-Lee electric field (E_m^*) propagated (or time-shifted) to the polar cap ionosphere from the L1 point is linearly proportional to the cross polar electric field E_{PC}. This is well-documented in literature and is also observed for other forms of the solar wind coupling functions and polar cap index [10,49,74].

~~a.~~b. Validation of the Error Model

- The model predicts the pdf of the erroneous estimate of the shocked solar wind driver E_m^*. As shown in Extended Data Figure 2a, the pdf of X^*closely fits the pdf of E_m^*.

- The model predicts the standard deviation of the normalized error. A good way to visualize how the uncertainty varies with the magnitude of X^* is to calculate the standard deviation of the normalized error $\sum(X^* - \mathrm{X})/\mathrm{X}$. This quantity largely matches with its counterpart in data $\sum(E_m^* - E_{PC})/E_{PC}$ (See Extended Data Figure 2b). The fact that this match implies the shocked solar wind driver $E_m^{sh} \propto E_{PC}$, and our assumption is consistent with even the second order statistics. This also aligns with the fact that E_{PC} is constructed by maximizing the correlation with E_m^* [~~126~~], hence all the contribution to the variance comes from the difference between E_m^{sh} and E_m^*, instead of uncertainties in the measurement of E_{PC}. If the values we assume for the uncertainties dt_1, dt_2, and ε, are inadequate, the model will underestimate or overestimate the statistical variation in the difference between E_m^* and E_{PC} shown in Extended Data Figure 2b.

- Furthermore, the model predicts the conditional normalized error distribution itself. A more detailed picture of the statistical properties of E_m^* can be obtained by plotting the conditional probability density of the normalized error given X or E_{PC}. In Extended Data Figure 2d-e, the left plot is the conditional pdf of normalized error calculated from the data, and the plot on the right is the same from the model. Both have a similar structure, with the spread in error increasing up to ~12 mV/m and then decreasing. This is consistent with our assumptions of the variation of the spatial scale sizes with solar wind strength.

- Finally, the model shows that the estimates of random, unbiased uncertainties in the solar wind input (X^*) lead to a conditional bias in the estimate of the true driver X, with the bias

varying with the magnitude of X^*. This bias exactly reproduces that seen in the data. In Extended Data Figure 2c, the black line is plotted from data and shows the conditional bias in the erroneous estimate E_m^* given its strength. It matches remarkably with that calculated by the model – shown in magenta. This non-linearly increasing bias is ultimately a result of the regression to the mean ~~effect~~[22–24,53,54]effect[16,17,19 75,76].

Extended Data Figure 3 is another way to explain this effect. The left panel shows the probability distribution of the true value X. Each bin associated with X is assigned a unique color. The panel on the right shows the pdf of the error-prone estimate of X, i.e., X^*. However, the colors still preserve their correspondence to the true value. The share of color in a particular X^* bin describes the proportion of X values misidentified as X^*. In each X^* bin, a larger proportion of lower X values are misidentified as X^*. In other words, for large X^*, X is less than expected. Converting this into the terminology of observations, we get back to the statement of the problem introduced in the main paper: for large values of E_m^*, $E_{PC} \propto E_m^{sh}$ is less than expected or saturates.

~~b.~~c. Analytical derivation of non-linear regression bias due to uncertainty in time

Here we analytically derive the general result that temporal uncertainty in measurement can lead to a perception of non-linearity in a linear system's correlation response. For this, we first assume a linear system with an input random variable X and output response variable Y, such that

$$Y = X \quad [1.3]$$

The input is measured or estimated with a temporal uncertainty represented by the random variable Δ. The erroneous estimate is W and is related to the true input X as

$$W = X(t + \Delta) \quad [1.4]$$

where the statistics of X and Δ are known

$$X \sim f_X(x)$$
$$\Delta \sim f_\Delta(\delta)$$

Our goal here is to find the erroneous regression function $\langle Y|W\rangle = f_r^*(w)$ and see how different it is from the true regression function $\langle Y|X\rangle$. Based on equation [1.3], $\langle Y|X\rangle$ is simply $f_r(x) = x$. We also note that since the system has a simple linear response $\langle Y|W\rangle = \langle X|W\rangle = f_r^*(w)$. For the rest of the section, our goal is to derive the function $\langle X|W\rangle$ or $f_r^*(w)$.

Firstly, we note that since $W(t)$ is a stochastic process, from equation [1.4], the conditional distribution

$$f_{W|\Delta}(w|\Delta = \delta) = f_{X(t+\delta)}(w)$$

But since X is assumed to be a stationary process,

$$f_X(w) = f_{X(t+\delta)}(w)$$
$$\Rightarrow f_{W|\Delta}(w|\Delta = \delta) = f_X(w) \quad [1.5]$$

However,

$$f_W(w) = \int f_{W,\Delta}(w, \delta) d\delta$$
$$= \int f_{W|\Delta}(w|\Delta = \delta) f_\Delta(\delta) d\delta \quad [1.6]$$

And from equation [1.6],

$$f_W(w) = \int f_X(w) f_\Delta(\delta) d\delta$$

As we assume X and Δ are independent, and $\int f_\Delta(\delta) d\delta = 1$, hence

$$f_W(w) = f_X(w) \qquad [1.7]$$

That is, **the marginal distribution of W and X has the same functional form**.

From equations [1.5] and [1.7], it follows that W **and Δ are also independent**.

$$\begin{aligned} f_{W,\Delta}(w,\delta) &= f_{W|\Delta}(w|\delta) f_\Delta(\delta) \\ &= f_X(w) f_\Delta(\delta) \\ &= f_W(w) f_\Delta(\delta) \end{aligned} \qquad [1.8]$$

Now we estimate the joint probability distribution function of W, X, and Δ.

$$f_{W,X,\Delta}(w,x,\delta) = f_{W|X,\Delta}(w|x,\delta) f_{X,\Delta}(x,\delta)$$

Since $X$and $\Delta$are independent,

$$f_{W,X,\Delta}(w,x,\delta) = f_{W|X,\Delta}(w|x,\delta) f_X(x) f_\Delta(\delta) \qquad [1.9]$$

By integrating the above over the range of Δ, we can calculate the marginal pdf of W and X.

$$f_{W,X}(w,x) = \int f_{W|X,\Delta}(w|x,\delta) f_X(x) f_\Delta(\delta) d\delta \qquad [1.10]$$

Finally, we can calculate the conditional pdf of X given W.

$$f_{X|W}(x|w) = \frac{f_{W,X}(w,x)}{f_W(w)}$$

From equation [1.7], we see that

$$f_{X|W}(x|w) = \frac{f_{W,X}(w,x)}{f_X(w)}$$

Using this and equation [1.10], we get

$$f_{X|W}(x|w) = \int f_{W|X,\Delta}(w|x,\delta) \frac{f_X(x)}{f_X(w)} f_\Delta(\delta) d\delta \qquad [1.11]$$

Hence from the definition of expectations we get,

$$\langle X|W \rangle = f_r^*(w) = \int x f_{X|W}(x|W=w) dx \qquad [1.12]$$

To solve equation [1.12], we need to assume a functional form for the pdf of X and Δ. $f_X(x)$ is assumed to be lognormal distribution, and $f_\Delta(\delta)$ to be a normal distribution. A lognormal random variable X has a corresponding normally-distributed random variable Z such that

$$
\begin{gathered}
Z \sim \phi(z, \mu_Z, \sigma_Z) \\
Z = \log X \\
\mu_Z = \log\left(\frac{\mu_X^2}{\sqrt{\mu_X^2 + \sigma_X^2}}\right) \\
\sigma_Z = \sqrt{\log\left(1 + \frac{\sigma_X^2}{\mu_X^2}\right)}
\end{gathered}
\qquad [1.13]
$$

Here, the function ϕ is the general normal distribution.

$$\phi(x, \mu, \sigma) = \frac{1}{\sqrt{2\pi}\sigma} e^{-(x-\mu)^2 / 2\sigma^2} \qquad [1.14]$$

Therefore,

$$f_X(x) = \frac{1}{x}\phi(\log x, \mu_Z, \sigma_Z) \qquad [1.15]$$

Additionally, as $X(t)$ is a stochastic process, the random variable at each time instance correlates to some degree with adjacent time instances. We define this using an autocorrelation function of the following form

$$\rho_X(\delta, k) = e^{-\delta / k} \qquad [1.16]$$

Here $\delta = |t_2 - t_1|$ is the absolute difference in time between two random variables, $X(t_2)$ and $X(t_1)$. For our case, this is also the error in measurement time corresponding to $\Delta \sim f_\Delta(\delta)$. ***k* is the autocorrelation time constant**.

The normally distributed uncertainty in time is defined as

$$f_\Delta(\delta) = \phi(\delta, 0, \sigma_\delta) \qquad [1.17]$$

Here σ_δ **is a measure of the random uncertainty in the time of measurement** Δ.

From equations [1.13] and [1.16], we can estimate the autocorrelation function corresponding to the normal random variable Z

$$\rho_Z(\delta, k) = \frac{1}{\sigma_Z^2}\log\left(1 + \rho_X(\delta, k)\left[e^{\sigma_Z^2} - 1\right]\right) \qquad [1.18]$$

From the results of the bivariate lognormal distribution, we can derive the functional form of $f_{W|X,\Delta}(w|x, \delta)$ to be the following

$$f_{W|X,\Delta}(w|x,\delta) = \frac{1}{w}\phi\left(\log w, \mu_Z + \rho_Z(\delta,k)[\log x - \mu_Z], \sigma_Z\sqrt{1-\rho_Z(\delta,k)^2}\right) \quad [1.19]$$

From equations [1.11],[1.12],[1.15],[1.17], and [1.19], we can calculate the regression function$\langle X|W\rangle$= $f_r^*(w)$.

$$f_r^*(w) = \int_0^\infty \int_{-\infty}^\infty \frac{\phi\left(\log w, \mu_Z + \rho_Z(\delta,k)[\log x - \mu_Z], \sigma_Z\sqrt{1-\rho_Z(\delta,k)^2}\right)}{\frac{\phi(\log x, \mu_Z, \sigma_Z)}{\phi(\log w, \mu_Z, \sigma_Z)}\phi(\delta, 0, \sigma_\delta)} d\delta dx$$

[1.20]

By integrating the above integral within the ranges of x, and substituting $\frac{\delta}{k} \to u$, we can show that the above integral is a function of the ratio $\frac{\sigma_\delta}{k}$. This ratio as a measure of uncertainty in the time of measurement.

$$f_r^*\left(w, \frac{\sigma_\delta}{k}\right) = \int_{-\infty}^{\infty} w^{\rho_Z(u)} e^{\frac{1}{2}[1-\rho_Z(u)][2m_Z + (1+\rho_Z(u))\sigma_Z^2]} \frac{1}{\sqrt{2\pi}\,(\sigma_\delta/k)} e^{-\frac{u^2}{2(\sigma_\delta/k)^2}} du$$

$where\ u = \delta/k$
[1.21]

We numerically integrate the above integral within the ranges of Δ for specific values of σ_δ/k and generate Figure 3c. The pdf of X is similar to that assumed in the previous section, with the mean $\mu_X = 1.12$ and standard deviation $\sigma_X = 1.15$. The figure shows that $f_r^*(w, \sigma_\delta/k)$ varies non-linearly with w when the temporal uncertainty ratio is larger than 0%, compared to $f_r(x) = x$ (which is linear) when $W = X$ or the temporal uncertainty ratio is 0%. Therefore, we have found that the regression function $\langle Y|W\rangle = f_r^*(w, \sigma_\delta/k)$ is non-linear with respect to w for values of $\sigma_\delta/k > 0$. Finally, the numerical integration of the analytical solution described by [1.21] was compared with an independent Monte Carlo solution of the error model stated in equation [1.4], with the results being almost identical, confirming that our derivation is correct.

~~c.~~d.Summary of data analysis procedure in the computer code

The primary computer code (Code 1) constructs a Monte-Carlo simulation to solve a non-linear error model to show that the polar cap index saturation observed in data is a result of the regression to mean effect stemming from random error in the shocked solar wind driver estimates.

Step 1: Preparing and loading observations data. First 1-minute resolution WIND spacecraft data already time-shifted to bow-shock nose from the OMNI database, Polar Cap Index (PCI) data, and the magnitude uncertainty (in the form of standard deviation) varying with shocked solar wind driver (derived in Code 3) is loaded. All missing values, and out-of-range values, are converted into a 'nan' value, PCI is calculated from PCN and PCS as described in the Methods section. The Kan-Lee electric field at L1 (E_m^*) is calculated from WIND spacecraft measurements of plasma

and field parameters. Finally, all values of E_m^* and PCI that are not simultaneous are removed from the analysis. The difference between E_m^* and PCI from data is also calculated as a measure of the total error or difference between the driver and the response. Finally, the autocorrelation function of E_m^* , PCI, and $E_m^* -$ PCI is calculated.

Step 2: Constructing model input. We compare the pdf of E_m^* and PCI, create a log-normal pdf that best fits the PCI index values, which has mean 1.114 and standard deviation 1.14 and, in the log-space, has mean -0.2518 and standard deviation 0.85. We fit a spline-function on the autocorrelation values and compare the autocorrelation functions of E_m^* , PCI, and $E_m^* -$ PCI. From these values derived from measurements, we create the first input to the model: a stochastic process X which is the hypothetical true stochastic process (i.e., shocked solar wind driver value), with also an autocorrelation functions that is similar to that observed in data. For this we use an external function by Lienhard (2021) to generate multivariate lognormal random numbers with correlation. Then we construct random variables representing time uncertainties: dt1 and dt2 with distributions and parameters derived from previous literature and discussed in the Methods section. Next we construct a stochastic process $\varepsilon(t)$ representing the magnitude uncertainty, which has zero mean, and a variance varying with the magnitude of X according to the uncertainty derived directly from simultaneous magnetosheath and solar wind measurements using Code 3. $\varepsilon(t)$ also has an autocorrelation function with values similar to that derived from the autocorrelation function of $E_m^* -$ PCI.

Step 3: Solving the Monte-Carlo Error Model. By plugging in the above inputs of X, dt_1, dt_2 and ε, into the following error model $X^* = X\,(t + dt_1 + dt_2) + \varepsilon$, we estimate the stochastic process X^*. These stochastic processes are represented by an ensemble of 16000 realizations, with each realization consisting of 2^{12} time samples (i.e., ~4096 minutes). This can be increased to improve the statistics at the very low probability tail region of the log-normal distribution (i.e., for solar wind driver values > 15 mV/m^2). The resulting value of X^* is the solution of the error model. From this we calculate the conditional expectation of $\langle X|X^* \rangle$, and notice that there is an appearance of a saturation effect on the average shocked solar wind driver given solar wind driver values measured at L1. This is then compared with the conditional expectation of $\langle PCI|E_m^* \rangle$, and we notice that the curves have a good correspondence, suggesting the regression to the mean effect is the cause for the saturation of the polar cap index with solar wind driver value measured at L1.

Step 3: Validating the Monte-Carlo Error Model. We verify the reliability of the error model by comparing many second-order statistics, as well as full probability distribution and conditional probability distributions with that of their counter parts in measurements. We list here the different statistics from the model that have been compared to show good correspondence with their counterparts in data: probability density function of input - $pdf(X)$, probability density function of output - $pdf(X^*)$, the autocorrelation function of the input - $\langle X(t)X(t+\tau) \rangle$, autocorrelation function of the output - $\langle X^*(t)X^*(t+\tau) \rangle$, normalized marginal error distribution - $pdf\left(\frac{X^*-X}{X}\right)$,

the normalized marginal error distribution conditioned on X - $pdf\left(\frac{X^*-X}{X}|X\right)$, the normalized marginal error distribution conditioned on X^* - $pdf\left(\frac{X^*-X}{X}|X^*\right)$, standard deviation of normalized error - $\sigma(X^*-X)/X$, mean of error - $\langle X^*-X\rangle$ and finally the conditional $pdf(X|X^*)$.

Step 4: Saturation effect in another geomagnetic response parameter (SML). We load the westward auroral electrojet index (SML) from SuperMag database, remove all the data gaps, and select only the data points that are simultaneous with the time-shifted Kan-lee electric field measured from L1. We then assume a counter part for SML in the model, $Y_{SML} = -120X + \epsilon_Y$, where ϵ_Y is a Gaussian-distributed random error in the estimate of the SML value with zero mean and $\sigma_{\epsilon_Y} = 5$ units. When the conditional expectation of Y_{SML} given X^*, $\langle Y_{SML}|X^*\rangle$, is calculated we observe that it also saturates very similar to the saturation of SML index with solar wind driver values.

Step 5: Regression calibration: Correcting for the regression to the mean effect. The bias caused by the regression to the mean effect is quantified by $b = X^* - \langle X|X^*\rangle$, and this can be removed from X^* to estimate the corrected solar wind driver function $X^c = X^* - b = \langle X|X^*\rangle$. This is carried out in the code by a one-dimensional linear interpolation of the solar wind driver value measured at L1 and time-shifted to the bow-shock (E_m^*) with a look up table consisting of sample points $x_i \in [0,25]$ and its corresponding values $f(x_i) = \langle X|X^* = x_i\rangle$ for each query point E_m^*. Therefore, the result is $E_m^c = f_r(E_m^*)$. We now plot the conditional expectation $\langle PCI|E_m^c\rangle$ and $\langle SML|E_m^c\rangle$ all derived directly from data. They reveal a linear relation of PCI and SML with the corrected solar wind driver function (or the true shocked solar wind driver value). We refer interested readers to the following literature for further discussion on the distributions of solar wind parameters, and geomagnetic response ~~measures[21,22,30,55,56].~~ measures[15,16,72,77,78].

Code 2 integrates the analytical formula in Eq [1.21] to solve for the regression bias stemming from uncertainty in the time of the measurement of a stochastic process. The code outputs a sensitivity analysis of the regression bias for different values of the ratio of time uncertainty to the autocorrelation time constant. Lastly, we validate the analytical solution independently; by developing an error model of only time uncertainty and solving it using the Monte Carlo method. The sensitivity analysis from the analytical result and the Monte Carlo solution match very well, confirming our analytical derivation is correct.

Code 3 uses simultaneous magnetosheath satellite measurements and measurements of spacecraft at L1 from the OMNI database, to calculate the variance of the L1 solar wind driver estimates for a given value of the shocked solar wind driver close to magnetopause subsolar point. The resulting variance is the magnitude uncertainty, and a spline fit of the variation of the magnitude uncertainty with increasing value of the shocked solar wind driver is used in Code 1 to reconstruct the magnitude uncertainty ε and its heteroskedastic variation with the magnitude of X in the error model in Code 3.

Code 4 uses probability theory to show that the true value corresponding to an uncertain measurement of a Gaussian random process is biased towards the mean. This is a demonstration of the regression to the mean effect through the lens of probability theory.

EXTENDED DATA

Extended Data Figure 1| Stochastic properties of inputs to the error model. a, The black line shows the pdf of the polar cap index E_{PC} from 1995 to 2019. And the purple line shows the lognormal pdf of the model random variable X that best fits the pdf of E_{PC}. **b,** The black dashed line shows the autocorrelation function of the polar cap index E_{PC} averaged over data from 1995 to 2019. And the purple line shows the autocorrelation function of the model random variable X that closely follows the ACF of E_{PC}. **c,** The black line shows the uncertainty in the propagation delay from L1 to the bow-shock nose(dt_1). The blue line shows the uncertainty in the propagation delay from the bow-shock nose to the polar cap ionosphere(dt_2). The magenta line shows the total uncertainty in the propagation delay from L1 to the polar cap$(dt_1 + dt_2)$.

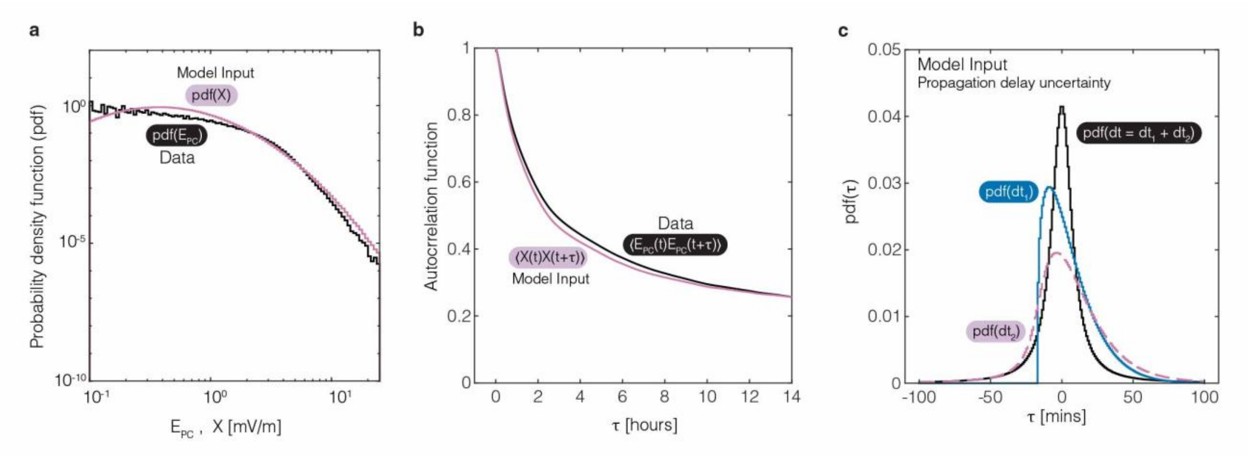

Extended Data Figure 2| Statistics of the output of the error model. a, The model predicts with good correspondence the pdf of the estimate of the merging electric field propagated to the polar cap, with a probability density difference of less than ~0.1 between data and model for most values. E_m^* is data, and X^* is model. **b,** The model predicts the nature of the random error between the estimate and the true value of the merging electric field projected on the ionosphere. The black line is the normalized uncertainty associated with E_m^* (data), and the purple line is the normalized uncertainty associated with X^* (model). **c,** There is a non-linear conditional bias in E_m^*: $\langle E_m^* - E_{PC} | E_{PC} \rangle$ that varies with E_{PC}, which is reproduced very well by the model $\langle X^* - \mathrm{X} | \mathrm{X} \rangle$. **d,** Conditional normalized error distribution from data, **e,** Conditional normalized error distribution from the model. Both data and model show similar trends in how the error varies with increasing strength of the true variable –a steep increase in the spread of the error from 0 to ~12 mV/m in the vertical axis, and then a decrease (or loss of power) beyond that point.

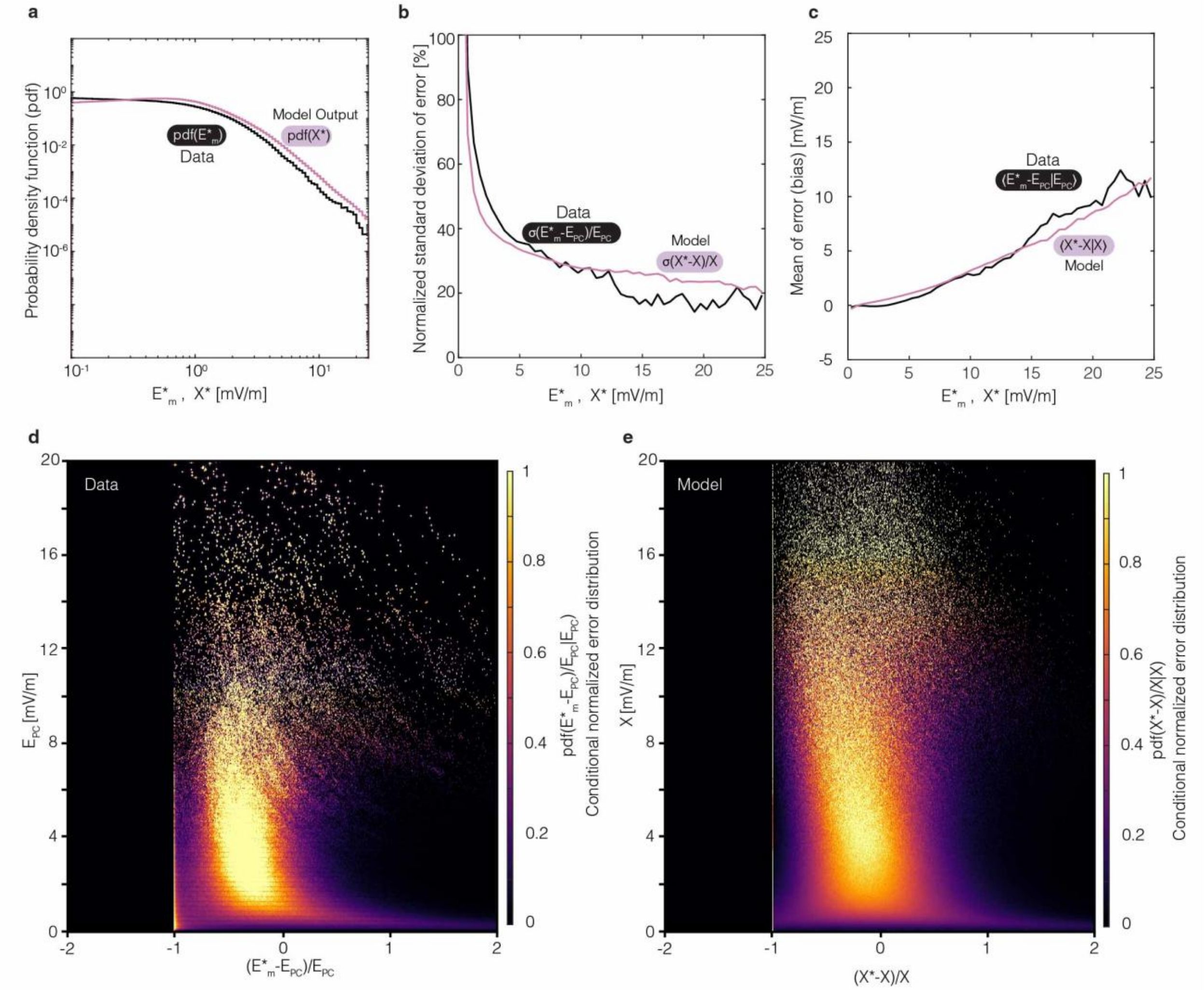

Extended Data Figure 3| Another graphical explanation of the regression to the mean effect. (Left) Probability distribution function of the true variable X, with each bin colored uniquely. (Right) The probability distribution function of the inaccurate estimate of X, i.e., X^* with the color representing the magnitude of X. The amount of a particular color per bin represents the proportion of X values misidentified as X^*.

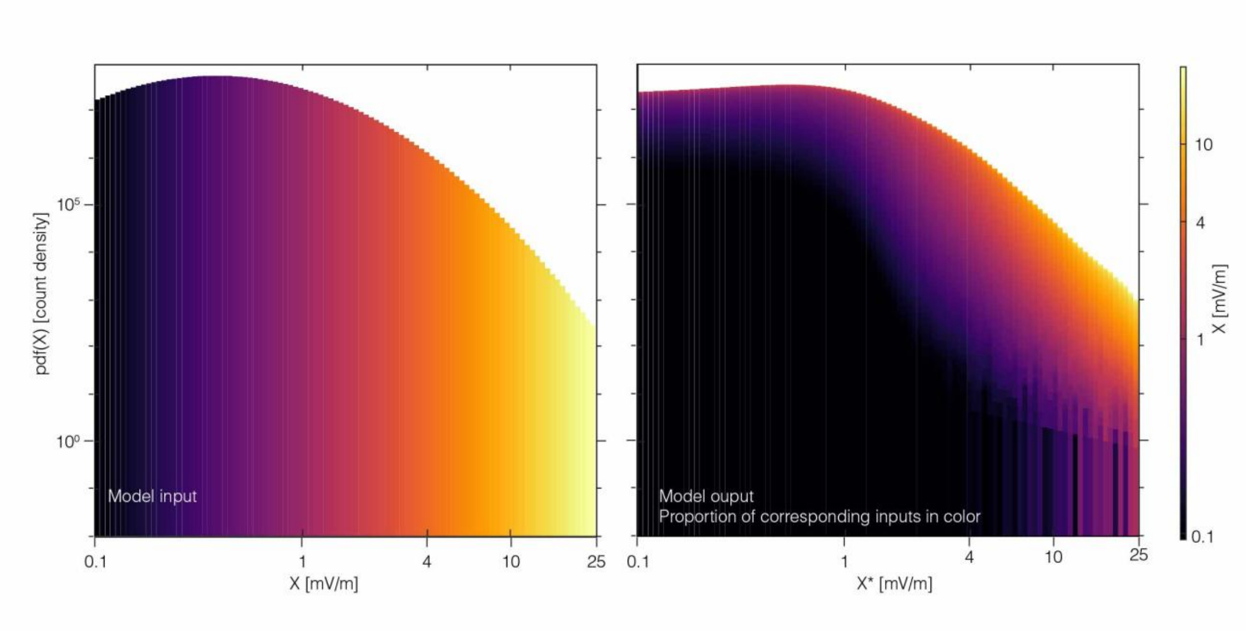

Extended Data Figure 4| Sensitivity analysis of magnitude uncertainty. a, Non-linear regression bias caused by heteroskedastic magnitude uncertainty increases with a measure of the uncertainty represented by the minimum relative magnitude uncertainty. **b,** The heteroskedastic variation of the magnitude uncertainty with the true value X is shown, for different values of the minimum relative magnitude uncertainty corresponding to the different regression bias curves in panel a.

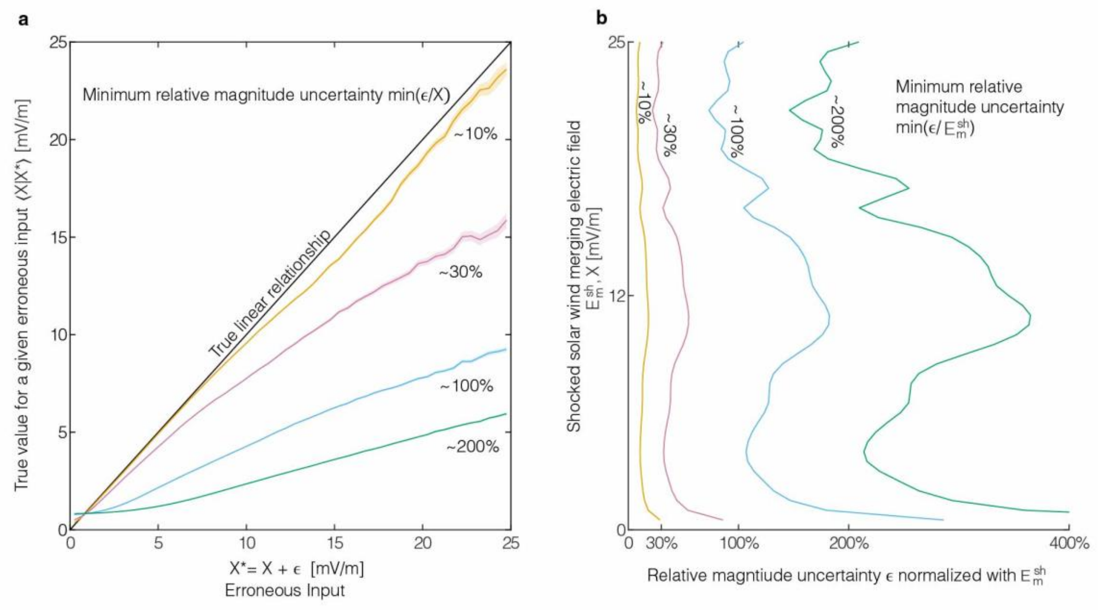

Extended Data Figure 5| Increasing trend of the saturation limits observed by previous observational studies. **a,** Cross polar cap potential response to solar wind driving is linear up to a saturation limit, which varies depending on the observational study and the methodology they use. All except Boyle et al., 1997 [~~51~~24] infer a saturation beyond the limit in E_m^*. See the studies here: 1 [~~57~~25], 2 [~~58~~26], 3 [~~59~~27], 4 [~~51~~24], 5 [~~60~~28], 6 [~~61~~29], 7 [~~62~~30], 8 [~~63~~31], 9 [~~64~~32]. **b,** The saturation limit of different studies follow an increasing trend (linear regression) with the year of study, with the latest studies including higher number of data samples.

a

Study	Saturation limit in E_m^* [mV/m]	Instruments
1) Reiff et al., 1981	~ 3	LEO Satellites: AE-C, AE-D, S3-3
2) Wygant et al., 1983	~ 0.5	LEO Satellites: S3-3
3) Weimer et al., 1990	~ 4	Magnetometers, AE Index
4) Boyle et al., 1997	~ 8 (found no saturation)	LEO Satellites: DMSP
5) Russell et al., 2001	~ 3	AMIE
6) Liemohn & Ridley, 2002	~ 10	AMIE
7) Shepherd et al., 2002	~ 3	SuperDARN radars
8) Nagatsuma, 2002	~ 5	Magnetometer PC Index
9) Hairston et al., 2005	~ 10	LEO Satellites: DMSP

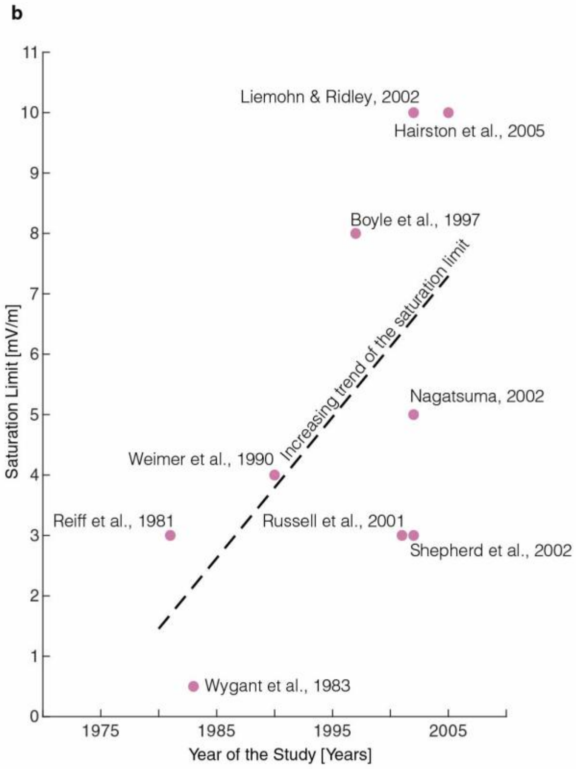

Extended Data Table 1| Theories of polar cap potential saturation. Ten models explaining cross polar cap potential saturation. See the following works for more details about each of the models: ~~1[65,66]~~1[33,34], 2 ~~[67,68]~~[35,36] , 3 ~~[69,70]~~[37,38], 4 ~~[71]~~[39], 5 ~~[70,72]~~[38,40], 6 ~~[73]~~[41], 7 ~~[74]~~[42], 8 ~~[75]~~[43], 9 ~~[76]~~[44],10 ~~[77,78]~~[45,46] and an assessment of most of ~~them[15]~~them[9]. Adapted with permission from [Joseph E. Borovsky, Benoit Lavraud, Maria M. Kuznetsova, Table A1, Polar cap potential saturation, dayside reconnection, and changes to the magnetosphere., Journal of Geophysical Research: Space Science, Publisher: John Wiley and Sons] Copyright 2009 by American Geophysical Union.

Theory/Model	Description
1) Hill model	Reduced dayside magnetosheath field lowers the reconnection rate
2) Flaring model	Flaring lowers the reconnection rate
3) X-line length model	Magnetosphere size changes decreases total reconnection
4) Force-balance model	Magnetosheath flow divergence reduces geoeffective length
5) Magnetosheath flow model	Solar wind conditions alter dayside reconnection rate
6) Lobe-bulge model	Bulging lobes impedes dayside reconnection
7) Bow shock model	Polar cap currents alter bow shock and dayside reconnection rate
8) Ionospheric outflow model	Ionospheric outflow mass-loads magnetospheric convection
9) Ram pressure-saturation model	Dynamic pressure constraints currents
10) MHD generator model	Alfven conductance of the shocked solar wind plasma limits currents

Extended Data Table 2| Common symbols used in this work. Variables representing measurements and their corresponding counter parts in the error model used commonly through the paper are summarized here.

Symbols	Description	Symbols	Description
E_m^*	Solar wind driver, Kan-Lee electric field, merging electric field, measured at L1 Lagrange point and time-shifted to bow-shock	X^*	Measurement, Model counter part of solar wind driver measured at L1 Lagrange point and time-shifted to bow-shock
E_m^{sh}	Shocked solar wind driver measured in the magnetosheath close to the subsolar point near the magnetopause	X	True value, Model counter part of shocked solar wind driver close to the subsolar point in the magnetosheath
E_{sw}	Solar wind convection electric field	ϵ	Zero-mean random error, magnitude uncertainty
E_{PC}	Dawn-dusk convection electric field across the polar cap	Y_{PC}	Model counterpart of dawn-dusk convection electric field across the polar cap, also counter part of PCI
E_m^c	Calibrated solar wind driver	X^c	Calibrated solar wind driver
E_R	Reconnection electric field	b	Regression bias (due to regression to the mean effect)
PCI	Polar cap index, also a proxy for E_{PC}	Y	True value, model counter part of geomagnetic response
PCN	Polar cap index derived from a magnetometer in northern polar cap	Y^*	Erroneous measurement of Y
PCS	Polar cap index derived from a magnetometer in the southern polar cap	dt	Uncertainty in the time of measurement
SML	Westward auroral electrojet index derived from SuperMAG network of ground magnetometers	dt_1	Uncertainty in solar wind propagation delay from L1 to bow shock nose
$B_{T,sw}$	Transverse magnitude of solar wind magnetic field	dt_2	Uncertainty in propagation of the effect of solar wind from bow shock to the polar cap ionosphere
B_z	Magnetic field along the Z-axis of the GSM coordinates	σ_δ	Standard deviation of random uncertainty in time of measurement
B_{sh}	Magnetic field value in the magnetosheath	k	Autocorrelation time constant
V_{sh}	Plasma velocity measured in the magnetosheath	V_{sw}	Solar wind plasma velocity
θ'	Shear angle between the magnetic field vector in the magnetosheath and the magnetosphere at the magnetopause	θ_{sw}	Solar wind magnetic field clock angle

Response to Referees 2

Referee 1 1
Referee 3 2
List of changes from Revision v1 to Revision v2 17
References 21

To the Editor and Referees:

The following is our response to the reviewer's comments. We label the referee's comment as **RCv2** and label our response **ARv2** (Authors' Response).

A list of revisions is provided under "List of changes from Revision v1 to Revision v2 ". These changes are also provided below next to the label Revision, when a revision is necessary. The line numbers and section numbers provided in this letter are associated with the **Marked-up revision file** and **Marked-up supplementary file**, which is the version of the revised paper with markup including the editorial changes that we have made to shorten the main text further. The line numbers of the marked-up supplementary file continue from the main marked-up revision file.

Referee 1

Referee #1 (Remarks to the Author):

I have read through the reply letter and the revised manuscript. I think that the authors have made excellent work to validate their analyses and polish the article with more important details and more convincing results.

I agree with the statement in the article that the regression-to-the-mean effect is not an alternative theory competing with the existing theories of saturation but a demonstration for further validation of the latter. This conclusion has important implications in both space sciences and various other fields.

I would like to recommend the acceptance of the manuscript at the present form. Meanwhile, I will draw the attention of the authors in the following aspect, which may be of interest to future investigation: there exists the coupled nature of the magnetosphere-ionosphere-atmosphere system under the impact of solar activity. How to deal with this coupling complexity so as to accurately quantify the regression-to-the-mean effect can be intriguing.

Referee #1 (Remarks on code availability):

The code provides a very useful aid to understand what the authors have done to deal with correcting the effect of uncertainties in association with measurements of solar wind driving and geomagnetic responses of Earth.

ARv2 1: We are grateful for the referee's comments, and for noting that the manuscript's "conclusion has important implications in both space sciences and various other fields". We will also keep in mind the referee's suggestion regarding magnetosphere-ionosphere-atmosphere coupling for future work.

Referee 3

Referee #3 (Remarks to the Author):

RCv2 3.0.0.1: This manuscript aims to explain a likely common source of non-linear bias in regression analyses, specifically applied to the case of strong solar wind driving of Earth's magnetosphere, exemplified by the response in polar cap voltage (or proxies thereof). I appreciate the thoroughness of many of the authors' responses. Some of my concerns remain unaddressed or inadequately addressed and these are discussed below. My overall finding is that the manuscript is both interesting and useful but is overstating its novelty and is occasionally overly confident or narrow in context in the service of telling a coherent story. Whether this manuscript is suitable for Nature is clearly an editorial decision, but I believe the work would be much more compelling and impactful if the uncertainties and sensitivities were adequately explored and this has only been partly addressed in the revisions.

I will limit my comments to my previous comments and the responses, so items will be identified by the numbers used in the author responses.

ARv2 3.0.0.1: We thank the reviewer's commitment to make the manuscript more compelling and impactful, and as suggested by the reviewer we have further explored the uncertainties and their sensitivities in this revision (See RCv2 3.8.2).

Regarding the novelty of the work, we note that we have found no scientific publication that describes this general result – that regression to the mean is a fundamental property. Moreover, in 40 years of space science literature, no study has mentioned regression to the mean or proposed it as an explanation for geomagnetic response saturation (See RCv2 3.5.2). By identifying it as a property of the relationship between truth and measurement, we are able to provide a resolution to the long-standing mystery of the saturation of geomagnetic response.

RCv2 3.0.1: I find the argument based on the fraction of data displaying saturation uncompelling. There are numerous physical examples of saturation that represent a change in real behavior when approaching driving that exceeds the norm. For example, in MOSFET (field effect transistors) there is an approximately linear response to applied voltage until a saturation limit. Whether a saturation limit is in the regime of typical driving or extreme driving is irrelevant to whether or not there is a real saturation effect. The argument that linearity in part of the parameter space implies that our expectation should be that it remains linear is not supported. Please note that I am explicitly not saying that I believe there is saturation in the magnetospheric driving, just that the argument here is inadequately supported. In the manuscript the authors show that a regression bias should exist, but the extent to which that invalidates the previous studies showing saturation is not sufficiently demonstrated. This was the central point in my previous review.

ARv2 3.0.1: We agree with the reviewer that "linearity in part of the parameter space" does not imply "that it remains linear" in other parts of the parameter space. That is, even if 95% of the data shows that geomagnetic response is linear, it doesn't imply that the geomagnetic response of 5% of the outliers is linear. However, the regression to the mean effect has a more significant impact on the extreme and rare values as compared to values that are more common, and hence we cannot infer relationships from measurement without considering the regression bias. As we know, since none of the previous observational studies in literature mention or consider regression bias, we cannot take their conclusion of a saturation effect as sufficiently demonstrated.

RCv2 3.0.2: Yes, the authors correctly point out that the model presented is a model resulting in a non-linear bias. I had intended this as a description of an additive (i.e., linear) model, which does not necessarily require that the relationship between predictor and outcome is linear. It is also true that the "true" driver and outcome have a linear relationship in the presented model. Certainly my phrasing was poorly chosen and ambiguous, however, the main point was that the model presented can approximately reproduce the form of observed saturation and that is a necessary but not sufficient condition for claiming that the saturation is due to nonlinear bias. The comment continued to then question whether sufficient evidence had been presented to establish that the outcome was robust to changes in the estimated parameters.

ARv2 3.0.2.1: We understand that the error model at initial glance can be thought of as an additive model, however, the heteroskedastic error $\epsilon(X)$ makes the error model $X^* = X(t+dt) + \epsilon(X)$, non-linear. This is because $\epsilon(X)$ is a non-linear function of X, and not just cX for some constant c.

ARv2 3.0.2.2: Regarding the statement "It is also true that the "true" driver and outcome have a linear relationship in the presented model.", we note that in our error model, the only relation is between the true and measured drivers - hence the outcome cannot be gleaned from the error model. Hence, in the presented [error] model there is no linear relationship between the true driver and the outcome. The regression bias due to uncertainties is the output (or outcome) of our error model. Once, the regression bias correction is applied to the solar wind driver measurement, a relationship - whether linear or non-linear - is revealed from a regression analysis of the outcome and the corrected solar wind driver. It so happens that for the estimated uncertainties presented in the paper, the response to the corrected driver is linear.

ARv2 3.0.2.3: We believe the removal of regression bias from data analysis is necessary before inferring the true relation between solar wind driving and geomagnetic response. Calculating an accurate regression bias can be done using an error model that is validated. We agree with the reviewer, it is not sufficient that the non-linear regression bias that we calculate "reproduces the form of observed saturation" (observed without correcting for the regression bias). However, the confidence in the non-linear regression bias that we estimate from the model comes from the validation of the second order statistics of the error model parameters with the counter part of the data. It is not only that the model reproduces the form of observed saturation, but it also reproduces the form of the pdf of the solar wind driver, the standard deviation of the normalized error, the conditional normalized error distributions, and the conditional probability density functions. Physical models of saturation presented in previous literature do not validate themselves with any of these second (and higher) order statistics, in fact, many of them are unable to validate the observed saturated curve as well. Hence, our error model satisfies the necessary condition according to the reviewer, and in our view, it also satisfies the sufficient condition (validation with second order statistics of data) that is not satisfied by any of the physical models in the literature.

RCv2 3.0.3.a: The authors claim that the inputs to the error model are constructed directly from data and measurements, but it's not clear that this is completely supported in the manuscript. I appreciate the addition of extended data figure 4, which goes some way to addressing the gap. The response states that the assumptions used are derived directly from measurements, which is true only if "derived" is used to mean "based on". The response further states that the model results are validated "making a sensitivity analysis superfluous". I disagree on this point. Showing qualitative agreement is a useful validation (though I note it is not quantitative) but it would still be important to understand how robust the result is to differences in the assumed parameters as this is not addressed by the presented validation.

ARv2 3.0.3.a.1: We would like to clarify the phrase "making a sensitivity analysis superfluous". Here, we mean that validation of model output with data is the necessary condition for determining the validity of its results. A sensitivity analysis is a method that examines how uncertainties in the input affects the model output, with the aim of 'assessing whether altering any of the assumptions made leads to a different final interpretation or conclusions' (Viel et al., 1995). In studies of the geomagnetic response to solar wind driving, an implicit model is assumed, i.e., the shocked solar wind driver is on average the same as the solar wind driver measured at L1 and hence we can use the latter as the driver of the magnetospheric system ($X^{*}{\sim}X$ or $E_m^{*}{\sim}E_m^{sh}$). Our work assesses whether altering this assumption by including uncertainties $X^{*} = X(t + dt) + \epsilon(X)$, leads to a different final interpretation of the geomagnetic response to solar wind driving. And we conclude it does in a specific way, the saturation of the response is a result of random errors in the driver that we use, i.e., the implicit model we use in space science has a problem with its assumptions. In other words, our manuscript is already a sensitivity analysis of an existing implicit model ($X^{*} = X$). We can call this an uncertainty analysis as well. The reviewer believes this is not sufficient, we ought to carry out a sensitivity analysis over the sensitivity analysis we have already done. That is, we should examine the uncertainty we have in the uncertainties we have estimated. Such second order sensitivity analysis is rarely carried out in our field and outside, and it is rarely thought of as necessary. Furthermore, if one has an uncertainty in the uncertainty of a parameter, the final uncertainty is likely a convolution of the two. Therefore, the first uncertainty is the minimum uncertainty you have on the parameter. Uncertainty in one's estimate of uncertainty, only increases one's uncertainty. In other words, the first uncertainty analysis will be a conservative estimate, already guaranteeing the robustness of the result of the uncertainty analysis. This is the reason why we believe the second-order sensitivity analysis is not necessary, but we understand it is of interest to the reviewer, and it is also of interest to us.

ARv2 3.0.3.a.2: We are in agreement with the reviewer that such a second-order sensitivity analysis is useful to carry out. Figure 3c (New Extended Data Figure 4c), and Extended Data Figure 4a-b, show that the model is not sensitive to fluctuations. These figures show that a 10% difference in time or magnitude uncertainty leads to only small changes in the regression bias. However, in this revision we take the quantitative analysis even further and show that the error model has a low sensitivity to the inputs and the output is robust to changes in the input**. If the model were sensitive, for small changes in input, there will be a large change in the output, instead we find that for a small change in the input, there is an even smaller change in the output.** We include the new sensitivity plots in this response (See RCv2 3.8.2) and thereby address the reviewer's concern of robustness.

RCv2 3.0.3.b: The claim that the assumptions in the error model are derived directly from measurements is not entirely supported. The dt1 estimate is based on a single paper and the chosen distribution and parameters are not explained. Similarly, the estimate of dt2 cites a couple of papers, but is light on justification of the specific distribution and parameters. On line 525 the authors state that "[historically], researchers have used a constant propagation delay (t2) of about ~20 minutes" without support. The support is better for the magnitude uncertainty, but separating the data-derived quantities from the assumptions should be clearer. Finally, the authors note that "the statistics become poor" at high values of sheath electric field, but no estimates of uncertainty are given. Were this review for a different journal I might be inclined to just treat these as assumptions and recommend softening the language somewhat to not draw the conclusions in such a strong fashion. Given that this is for Nature, I feel that the bar should be higher than it has been drawn in this manuscript.

ARv2 3.0.3.b: For dt_1 estimate, we add further references and several paragraphs of additional explanation of the way in which the distribution and its parameters are estimated. For dt_2 estimate, we do the same. For magnitude uncertainty, we calculate the uncertainty using simultaneous L1 and magnetosheath measurements up to 12mV/m and thereafter calculate the uncertainty using simultaneous L1 and solar wind measurements upstream of the bow shock. For the phrase "statistics becomes poor" at high values of sheath electric field (>12 mV/m), we clarify that at high values there are less than 10 data points available per bin and no extreme events in the dataset of simultaneous L1 and sheath measurements used to calculate the magnitude uncertainty.

Revision:

2. We add an additional reference to the latest cross-correlation study to estimate the uncertainty of dt_1 in the Methods section: "Based on their results and consistent with other studies[59], we assume dt_1 to be an independent random process with a pdf of a Student's t-distribution with shape factor 1.3, mean 0, and standard deviation ~8 minutes (See Extended Data Fig. 2c and Supplementary Methods 2.bExtended Data).)." (Line 552-555)

10. We justify the choice of dt_1 distribution and add additional references. We also point out that dt_1 is estimated by using cross-correlation analysis of data. "Similarly, for the time uncertainty dt_1, we use a standard deviation of ~8 minutes and a Student's t-distribution with shape factor 1.3 to simulate the narrow but heavy-tail distribution seen in Case & Wild's results[58]. Case & Wild estimate propagation delays directly by cross-correlating solar wind measurements from ACE and Cluster satellites, and compare these calculated delays with propagation delay estimates from the OMNI database we use. The regression bias caused by our estimated Student's t-distribution is slightly lower than the equivalent normal distribution with ~8 minutes 1σ standard deviation (i.e., it is a conservative estimate). Recently, Tasnim et al.[59] estimated the propagation delay using a combination of cross-correlation and validation methods, reporting a ~18-minute 2σ standard deviation in propagation delay uncertainty, consistent with our estimate." (Line 1166-1175)

11. We justify the choice of dt_2 distribution, and not that it is also estimated using cross-correlation analysis from data. "For the time uncertainty dt_2, we use a standard deviation of ~25 minutes, consistent with the results of Milan et al.[14] and Stauning[50]. Milan et al.[14] derive the propagation delay to the ionosphere using cross-correlation of field-aligned currents and solar wind B_z. They find a time-lag distribution peaking at ~17 minutes with a broad peak ranging from 10-30 minutes. Stauning's[50] cross-correlation of PCN index with the solar wind merging electric field just upstream of the bow shock shows propagation delays from -5 to 50 minutes with an average of 20 minutes. We estimate the probability distribution of dt_2 as a Weibull distribution (shape factor 1.3) to capture the broad spread and ensure a positive total delay. The total regression bias caused by this distribution is again slightly lower than that of the equivalent normal distribution with a ~25-minute standard deviation, making it a conservative estimate. In other words, using a normal distribution for propagation time uncertainties would yield a similar regression bias as seen in our results, implying the results are insensitive to the heaviness of the distribution's tail." (Line 1176-1187)

12. We point to references that support the usage in literature of a constant propagation delay (t_2) "The delay in this propagation is on average 20 minutes~17 minutes[14] but can vary from -5 to 50 minutes[50]. Historically, researchers have used a constant propagation delay (t_2) of about ~20 minutes10-30 minutes[27,32]"(Line 560-562)

9. We clarify the only assumption made in estimating the magnitude uncertainty is at high values of the sheath electric field, when we have less than 10 points available per bin, we use the uncertainties calculated from simultaneous L1 and solar wind measurements upstream of the bow shock. This is therefore a conservative estimate of the magnitude uncertainty. "For magnitude uncertainty, when E_m^{sh} > 12 mV/m, we used uncertainties from simultaneous L1 measurements and solar wind values upstream of the bow shock, adopting a conservative estimate of the uncertainty (~30% relative error). For simultaneous magnetosheath measurements, the sample size is small, with fewer than 10 data points per bin, suggesting no extreme geomagnetic storms fall within the constraints of simultaneous L1 and sheath measurements near the subsolar point." (Line 1160-1165)

RCv2 3.0.5.2: The explanation of how the saturation effect impacts this specific problem is new. There is, of course, a sliding scale of novelty and this manuscript does have genuinely novel aspects. Whether this is sufficiently novel to justify a paper in Nature is the context for the previous comment, and my opinion appears to be different from the other reviewers. However, while I agree with the authors' statement that " even if one believes that this insight is not new, it is important to point out …", it is again in the context of this submission being to Nature that I'm not convinced that "important to point out" warrants publication in Nature as opposed to the plethora of other respectable journals. However, that is clearly an editorial decision.

ARv2 3.0.5.2: We agree with the reviewer that the phrase "important to point out" does not imply that the point that is being made in novel. However, one reason we think the results we present are novel is because we have not found any scientific publication that describes the general result – that regression to the mean is a fundamental property. And furthermore, in 40 years of space science literature, no study has mentioned regression to the mean or proposed it as an explanation for geomagnetic response saturation (See RCv2 3.5.2). By identifying it as a property of the relationship between truth and measurement, we are able to provide a resolution to the long-standing mystery of the saturation of geomagnetic response.

RCv2 3.0.6: The authors have perhaps over-interpreted this comment. My use of the Reiff result was simply illustrative and not intended to be a sole point of comparison. I also agree with the authors that the specific result is new and likely impactful (though the degree is debatable). I disagree that the authors have shown that the general result is particularly new though it is, again, useful.

ARv2 3.0.6: We are happy to note that the reviewer agrees that the general result is useful. And regarding its novelty, we have extensively searched literature within the field and outside, and we have so far found no evidence that the general result (i.e., the regression to the mean is a fundamental logical consequence of the relationship between the true value and its measurement) has been published elsewhere (See RCv2 3.5.2). Hence, our conclusion that it's novel.

RCv2 3.2: Making a testable prediction is indeed necessary, but being testable and being tested are two different things. Interpretation of the Hill paper as "clearly" arising "from some observational inference" is a possible interpretation, but the paper is not presented that way. The inference, if it was a motivating factor, could have been based on physical intuition (i.e., drawn from understanding of the physics underlying the presented model) with a throwaway reference to unshown data being added later. I find this to be an overstatement designed to tell a simple story rather than embracing the nuance.

ARv2 3.2: We agree with the reviewer that there are different possible interpretations of how Hill et al., 1976 arrived at their model. We are aware that Hill, Reiff, and Rassbach were colleagues who worked together, and the model arises from pre-existing knowledge that observations circulating at that time showed saturation effect in data. Evidence that Hill et al., already knew about a saturation effect shown by observations is present in their paper itself as we previously mentioned: "For Earth, the limiting potential of about 55 kV" [calculated from their theory] "is in reasonable accord with observations". Furthermore, we do not make any explicit statements regarding the Hill model in our paper, we make a statement that is generally true in our field, that the models are developed to explain the observational fact. Therefore, we do not believe this is an overstatement. The story of the polar cap potential saturation is far from simple; we have tried to explain this complexity as objectively as we can, given the literature and within the constraints of our word limit.

RCv2 3.3.a: New extended data figure 5 is problematic. The inclusion of Boyle et al. with a saturation limit despite their explicit statement that they did not see evidence of saturation is misleading. The line to guide the eye is also misleading. Note that the studies from 2002-2005 span the range of 3-10 mV/m, and no trend is evident. In the spirit of quantitative support, I calculated the linear correlation for the displayed points (with and without the Boyle study) and found that while the correlation was approximately 0.6 in both cases, the confidence intervals on the correlation included zero. Thus showing the trend line without noting that it's also consistent with no trend is misleading.

ARv2 3.3.a: Confidence Intervals are not applicable here, as the correlation being estimated is the correlation of the reported saturation limits of the 9 studies, and not an estimate of the correlation of reported saturation limits of some underlying unknown population of observational studies. Hence the true Pearson correlation of reported saturation limits versus year of the study is 0.61, and nothing else.

We agree with the reviewer that the Pearson correlation coefficient of the increasing trend line is 0.61. We do not calculate the confidence interval (CI) because this statistic is **not applicable** here as we explain below. Despite this inapplicability, if we still calculate the CI, we get the following interval [-0.07, 0.91]. The reviewer states that as "the confidence intervals on the correlation included zero", this implies that the trend line is "also consistent with no trend". In other words, there is a probability that the Pearson cross correlation is also 0. This statement is false, and hence we do not include this statement in the manuscript.

To explain why a confidence interval of [-0.07, 0.91] on the correlation coefficient does not imply the true value of the correlation coefficient can be 0, we must explain the fundamentals of the statistic - the confidence interval.

We quote from a very instructive study Hoekstra et al., (2014). "A CI is a numerical interval constructed around the estimate of a parameter." Here the reviewer's choice of parameter is the Pearson correlation coefficient. "Such an interval does not, however, directly indicate a property of the parameter; instead, **it indicates a property of the procedure,** as is typical for a frequentist technique". CI is a statistic that is based on frequentist philosophy as opposed to the Bayesian philosophy. "Specifically, we may find that a particular procedure," in the reviewer's case the calculation of correlation of a sample, "when used repeatedly across a series of hypothetical data sets (i.e., the sample space), yields intervals that contain the true parameter value in 95% of the cases." Cumming and Fidler, (2015) points out that this implies it is misleading to say that "there is 0.95 chance our 95% CI includes [the population's parameter]", instead we might say "we are 95% confident our interval includes [the population's parameter]". Hoekstra et al., (2014) goes on to say "The key point is that the CIs do not provide for a statement about the parameter as it relates to the particular sample at hand; instead, they provide for a statement about the performance of the procedure of drawing such intervals in repeated use. Hence, it is **incorrect to interpret a CI as the probability that the true value is within the interval."**

If one tries to estimate an unknown population's correlation coefficient with access to only the correlation coefficient of a known sample, all we can conclude is that we can be 95% confident that the interval [-0.07, 0.91] includes the unknown population's correlation coefficient.

However, very importantly, the Extended Data Figure 5 (New Extended Data Fig. 6) presents reported saturation limits by observational studies in particular years in the past. This set of data is the population of interest and is not a sample of some unknown probability distribution of reported saturation limits whose parameters we wish to calculate. That is, there is no superset of an unknown number of

observational studies published between 1980-2005, whose parameters we are trying to estimate. The correlation coefficient of this data is therefore the population's correlation coefficient. In other words, the population's correlation coefficient is **known**, and one is not working with a sample or subset of an unknown population. As a result, the Pearson correlation coefficient of the data points shown in Extended Data Figure 5 (New Extended Data Fig. 6) is 0.61 and nothing else. It is important to note that the population's statistic (in our case, the correlation coefficient) is an objective material quantity, and "any single [confidence] interval either does or does not include [the population's statistic]" (Cumming and Fidler, 2015).

As a result, we state under the figure caption that the correlation coefficient is 0.61, following the lead of the reviewer. However, it is not true that this correlation coefficient can also be 0.

Revision:
7. In the Extended Data Fig. 5 (New Extended Data Fig. 6.) we change the figure caption as follows: "**b,** The saturation limit of different studies ~~follow~~presented here follows an increasing trend ~~(linear regression)~~ with the year of study~~,~~ (Pearson correlation coefficient ~0.61), with the latest studies including higher number of data samples." (Line 409-410 and Line 1542-1544)

RCv2 3.3.b: The Boyle study is also interesting in this case. Given that the nonlinear bias should be present in any such study, how did their data selection avoid the apparent saturation seen in other studies? Other questions that arise here include the data sources. Presenting this uncritically as an increasing trend sidesteps the natural question of how much the present model explains of the apparent saturation in previous studies.

ARv2 3.3.b: The evaluation of the source of saturation of previous studies is not a part of this manuscript, and we note that there are many sources for non-linearities that occur in previous studies, including under-sampling, choice of data (e.g., filtering), and type of instruments.

We agree that the question "how much the present model explains of the apparent saturation in previous studies" is not answered by our study. It is not the goal of our study. The only inference that we draw from our result regarding previous observational studies is that those studies mentioned in Supplementary Discussion Section 1.j are consistent with our result. This question the reviewer raises is, however, very interesting to us personally, and we already have a project with an international team of 11 scientists to review this previous literature, both observational and simulation-based, to examine and publish this as a review paper.

The fact that multiple studies see different saturation effects, and no saturation, already shows that non-linear bias can vary based on other effects like under-sampling bias, or bias due to instrumentation limitation (like in the case of the coverage area of ground-based radar), or also the satellites used to measure the solar wind driving. We agree with the reviewer that Boyle et al., 1997 is an interesting case, and we state in our manuscript in Supplementary Section 1.j that they find the linear relation "after a meticulous filtering of data". The details of this filtering will be one of the subjects of the above-mentioned review paper and it is not necessary to justify the results of our manuscript. However, we believe Boyle et al., 1997 uses solar wind data with a lower uncertainty and filters data to minimize the uncertainty in the solar wind driving. For example, they use the IMP-8 spacecraft which is in an orbit that is ~7 times closer to the Earth's bow shock than the L1 satellite. More importantly, Boyle et al., 1997 is the only study to filter data such that all the IMF components, Bx, By, Bz are individually quasi-

steady over a 4-hour time window. This filtering of data has the unique effect of not only increasing the autocorrelation time constant and thereby reducing the propagation or time uncertainty, but, it also reduces the magnitude uncertainty as it may reduce the uncertainty in the IMF clock angle, which is an important contributor to the magnitude uncertainty as is evident from Supplementary Methods 2.a. Furthermore, Boyle et al., 1997 also use multiple other filters in the geomagnetic response measurements (DMSP spacecraft) which will also be affecting their results. We suspect that these filters, that are not carried out by other studies in the cohort, are likely eliminating all the saturated data points from Boyle's final data set, and hence Boyle only finds evidence for a linear relation within their filtered data.

Also, we include Boyle et al., in Extended Data Figure 5 (or New Extended Data Fig. 6), as Boyle finds evidence for a linear relation up to ~8mV/m, however saturation could occur at higher values. We can place Boyle et al at 8mV or higher. We chose to place it at 8mV as it is the conservative option given our statement of an increasing trend of reported saturation limits with increasing year of study. We also note in the table and caption that Boyle finds no evidence of saturation in their work to accurately represent the results to the reader.

RCv2 3.4: Again, the authors' summary of my comment is not consistent with my intent (or, I believe, with the full comment). The quote I provided from Reiff et al. was, in fact, the key rather than the specifics of how they dealt with it. That is, they identified that the relationship between interplanetary drivers and the polar cap voltage was sensitive to the way in which the sheath field is inferred from the solar wind field. The conclusion was that the observed relationship between upstream solar wind and polar cap voltage seemed nonlinear, even though the relationship between the proximate driver (sheath field) and the response is linear. That is one of the insights presented here that certainly has not been emphasized in other literature to my knowledge. They obviously did not explain that in the same way as the authors do in the present manuscript. I noted in my comment that Reiff et al. explained this using a physical process which is clearly different from the present explanation. What I was pointing out is that the authors present the fact that a linear relationship between sheath field and response can show a nonlinear relationship between upstream field and response as being a new insight rather than noting that this was already posited by Reiff et al and thus this provides an explanation for their realization. The response claims that any theory involving reduction in dayside reconnection rate claims this position, but I do not recall this being clearly stated in other literature and I do not see this being given as context for the present work. My concern here is in the accurate presentation of prior literature, sufficient credit where due for prior work, and a clear delineation between the contributions of this work and what has come before.

ARv2 3.4: Reiff et al., 1981's does not provide sufficient evidence to show that " a linear relationship between sheath field and response can show a nonlinear relationship between upstream field and response", as they use no sheath measurements in their analysis. Hence, we credit Reiff et al., 1981 as the first study to present evidence for an apparent saturation, which we believe is an accurate presentation of the literature.

We share the reviewer's concern of accurate presentation of prior literature, sufficient credit to be given to prior work, and a clear delineation between the contributions of this work and what has come before.

We are sorry that we misunderstood the intent of referee's comment RC3.4 in the previous review report. We now understand this is referee's main point "What I was pointing out is that the authors present the fact that a linear relationship between sheath field and response can show a nonlinear relationship between upstream field and response as being a new insight". We do not claim anywhere in our manuscript that a linear relationship between sheath field and response can show a non-linear relationship between upstream field and response. We claim instead that zero-mean random error in the field measured at L1 relative to the sheath field leads to an appearance of non-linear relationship between upstream field and response. If Reiff et al. 1981 posits the claim that a linear relationship between sheath field and response can show a non-linear relationship between upstream field and response, Reiff et al., 1981 had no sheath measurements to back up that claim. For example, Reiff et al., 1981 only posits this: "[t]he degree of correlation between interplanetary parameters and the polar cap potential drop turns out to be quite sensitive to the way in which the magnetosheath field B1 is inferred from the (observed) solar wind field Bsw.", which is not sufficient evidence to claim there is a linear response to the sheath field. On the other hand, Pulkkinen et al., 2016 had sheath measurements to provide evidence for this claim, and hence we have noted it already in Supplementary section 1.j. line 1121-1129 and stated that "Their findings are entirely consistent with our explanation…". We have prioritized noting results supported by evidence in our manuscript to keep the manuscript within the constrained word limits. As a result, we instead chose to give credit to Reiff et al., 1981's main contribution - it being the first study to document evidence for an apparent saturation of the polar cap potential with solar wind driving in Extended Data Figure 5a or New Extended Data Fig. 6a.

RCv2 3.5.1: The authors' response here presents a number of useful descriptions of the phenomenon, but fundamentally I believe that the disagreement here is in what "statistical" means. I am likely to not be alone in interpreting each of the five vantage points as being statistical in nature. As anyone who has spent significant time with measurement knows, the measurement is not the "truth". Even a single count of a cosmic ray in a detector is biased in a way that can be explained by Poisson statistics - yes, it's more complicated than that, but the point is that being a fundamental issue not tied to any specific method does not mean it's not a statistical effect, at least not in the way that the term is widely understood. The authors' own explanation relies on "the stochastic properties of the truth, the degree of uncertainty of the measurement" (which would widely be interpreted as being statistical) and while this is important to highlight and may be valuable to bring to a wider audience, it's not a new insight.

ARv2 3.5.1: We completely agree with the reviewer that "being a fundamental issue not tied to any specific method does not mean it's not a statistical effect". In fact, through Figure 2 (New Extended Data Fig. 1) we show it's a probabilistic effect, and Vantage point 5 shows that it's a fundamental effect. There is a very fundamental relation between position and momentum which is famously known: the product of the uncertainties in position and momentum is greater than or equal to $h/4\pi$. This is a fundamental property of the relation between measurement of position and momentum, which is a probabilistic effect, and yet fundamental i.e., it is independent of methodology you use to measure position and momentum. We point out that regression-to-the-mean effect is also a fundamental effect and not only a statistical effect. We have not yet seen any publication that explains this, and hence we believe it is a new insight (See RCv2 3.5.2).

RCv2 3.5.2: As noted in my previous comment, I do not believe that this interpretation is strictly accurate. A new description of something that is known can be valuable but does not make the result new. The "new vantage points" are useful, if broadly equivalent, descriptions. But they do not represent new insight, just a nice presentation of insights already known.

ARv2 3.5.2: As we point out above (RCv2 3.0.0.1, RCv2 3.0.5.2, RCv2 3.0.6, and RCv2 3.5.1), we have not seen the insight "regression to the mean is a fundamental property of the relationship between measurement and truth" mentioned anywhere in previous literature, hence we believe it's a new insight.

Apart from the fact that our general result is not yet published elsewhere, we also note that it's not already known in our field based on the following arguments:

1. 40 years have passed in our field, since multiple studies have analyzed polar cap potential and other geomagnetic response variations with solar wind driving. None of those studies mentioned or even suggested the possibility of the regression to the mean effect leading to a bias in the truth.
2. The presentation of such a possibility, that saturation effect may be a result of the regression to the mean effect, elicits disbelief from other space scientists, and the results we obtain appear to be controversial in our field.

If the general result were well-known, we should not expect the above two phenomena. Which leads us to think that other fields may have biases from the regression to the mean effect that remain misinterpreted as evidence for some physical phenomenon.

RCv2 3.6.1: The response here is perhaps stronger than can be supported by the presented evidence. It doesn't show that the underlying data show no saturation. It shows that a nonlinear bias arising from nonlinear error can display a similar saturation effect. The degree to which the saturation arises from the regression to the mean effect (i.e., what fraction of the apparent saturation is due to this effect, which obviously depends on the assumed dt1, dt2 and epsilon) is important to clearly characterize to show that this invalidates, rather than modifies, the previous studies showing saturation. The sensitivity plots help to show that this can, for given assumptions, give a degree of saturation entirely consistent with the data. They also show that the result could indeed be sensitive to those assumptions and the difference (at least to me) between this being a Nature paper or being published elsewhere lies in showing that the result holds for a reasonable and justifiable range of parameters.

ARv2 3.6.1: The non-linearity is a function of dt1, dt2, epsilon, prior probability distribution, autocorrelation coefficient. Because it's a function of them, we agree with the reviewer that there is not any doubt that the results have some sensitivity to them. However, the regression function (or regression bias) is not the only quantity that is a function of the above. The probability distribution of the solar wind driver, the standard deviation of the normalized error, and the conditional normalized error distribution are all a function of and are sensitive to the above inputs. This is the reason we view the validation procedure of comparing the second order statistics with the data as the important, necessary and sufficient evidence to justify the input values. Furthermore, we show how the auroral electrojet index, a completely different geomagnetic response, also varies linearly with the corrected solar wind driver. Nevertheless, we do agree with the reviewer that it's useful to understand the sensitivity of the regression bias to the input parameters, and hence we have previously provided Extended Data Figure 4 and Figure 3c (New Extended Data Fig. 4). However, we understand this can be further strengthened, and hence, in this revision, we provide further detailed explanation and investigation of the sensitivity which demonstrates that the error model output is robust to changes in the inputs (See RCv2 3.8.2).

RCv2 3.8.1: Noting the difference in probability density is not a quantitative measure of uncertainty. There are numerous methods to assess correspondence, and even seeing similar qualitative results for different assumptions would go a long way to addressing the robustness of the result. While the authors have drawn attention to the log scale potentially highlighting small differences in the tail, I note that this also minimizes the much larger differences at higher probability density.

ARv2 3.8.1: We agree with the reviewer that understanding how the results vary with different uncertainty values is useful to evaluate the robustness of the result. As the reviewer noted, the Extended Figure 4 and Figure 3c (New Extended Data Fig. 4), show sensitivity analysis for the time and magnitude uncertainties, and it shows that a reasonable variation in uncertainties of 10% does not have any changes in the non-linearity of the regression bias, and only results in a small change in the linear slope of the regression curve. However, we understand the reviewer believes a more clearer presentation will further strengthen the robustness of the results, and we present this immediately below in RCv2 3.8.2.

RCv2 3.8.2: As discussed earlier, the input parameters are not entirely "calculated directly from data". While the paper does not show any tuning of parameters, that was not the question asked. If the assumed parameters of the error distributions are changed, given that many of them are loosely derived, how does the result vary. It may well be that the result is relatively insensitive in a reasonable range of parameter-space or assumption-space, but this is not clear and is somewhat important to showing that the presented explanation is indeed a full explanation and not just an explanation that reduces the amount of saturation. Figure AR-4 does indeed show some of this although it is not directly connected to the degree of apparent saturation expected. I disagree that this is beyond the scope of this work and while I welcome the authors' intent to address this in some future paper, I also believe that it is necessary for establishing the impact of the presented work in an unbiased manner. I would expect that this is explored to some extent in a Nature paper to justify the publication in Nature.

ARv2 3.8.2: We demonstrate below that the reviewer's intuition that "it may well be that the result is relatively insensitive in a reasonable range of the parameter-space or assumption-space" is correct. The sensitivity of regression bias to the spread of the true value, time uncertainty, and magnitude uncertainty, is low. We add quantitative values of the sensitivity to the manuscript Supplementary Methods 2.b and point out that any values greater than the estimated time and magnitude uncertainty, or lower than the estimated variance of the true value, will result in the full explanation of the amount of saturation observed by our error model. This means, our result is valid not just around the input values, but more than half of the input parameter space. We thank the reviewer for encouraging us to explore this to some extent, and we look forward to publishing a study that examines this even further in the future.

We understand reviewer's concern that the result may only be applicable within a narrow range of the input parameters, and in other words the result may be highly sensitive to the input parameter-space. However, we found that the reviewer's intuition " It may well be that the result is relatively insensitive in a reasonable range of parameter-space or assumption-space" correct. Using Eq 1.21, the regression bias calculated by the analytical derivation of the error model with time uncertainty, we are able to calculate the sensitivity of the regressed (or saturated) true value to the input parameters of time uncertainty and the variance of the true value. In AR-Figure 5a, we show examples of high sensitivity of the result for variation in a hypothetical input parameter. The input parameter is marked by the

vertical dashed black line. We should expect a highly sensitive model to show either a very steep slope around the input parameter value (e.g., purple curve) or a parabola (e.g., blue curve). This implies when the input parameter deviates away from the black line, the regression bias changes substantially. However, we should note that in the case of the purple curve, any change of the input parameter to the left-side of the parameter space implies all saturation (and more) is due to regression bias, and hence no saturation can exist in the results after they are corrected from regression bias. In comparison to these highly sensitive curves, a no-sensitivity scenario is depicted by the straight horizontal black line. Results of the sensitivity analysis by changing the input parameter of the probability distribution of truth - the standard deviation std(X) by +/- ~30% of the measured value: 1.15 mV/m, show a low sensitivity of the result +/-5.9% at X*=25 mV/m, which is within the minimum relative magnitude uncertainty of 30%. The result will have the highest sensitivity at X*=25mV/m, and hence we have shown it as the purple line (See AR-Figure 5b). Similarly, the result of the sensitivity analysis done by changing the input parameter of time-uncertainty dt by +/- ~30% of the estimated total value (dt1+dt2): 33 min, leads to a relative change in the regression bias by +/-9.65% at X*=25mV/m (See AR-Figure 5c). This is also within the relative magnitude uncertainty of 30%, and hence the results are of low sensitivity. Similarly, using the Monte Carlo simulation, we calculated the sensitivity of the saturated true value to the magnitude uncertainty. Here we find a similar result of the sensitivity analysis, where the input parameter of magnitude uncertainty was changed by +/-~30% of the given value in Figure 3d (i.e., New Figure 3c), leading to a relative change in the regression bias by +/-12.18% (See AR-Figure 5d). This is also within the relative magnitude uncertainty of 30%, and hence the result is of low sensitivity. If the results were highly sensitive, we should expect that for a small change in the error model input, there should be a large change in the regression bias. However, here we see that a change in input leads to a much smaller change in the regression bias. We had thought that this was evident from Extended Data Figure 4 and Figure 3c (New Extended Data Figure 4), however we thank the reviewer for encouraging us to state this quantitatively.

Finally, we would like to note that when we change the shapes of the uncertainty distributions of dt1 and dt2 from Student's t-distribution and Weibull distribution to Normal distributions with equivalent standard deviations, the regression bias resulting from the time uncertainty remains nearly the same, with the assumption of a Normal distribution increasing the regression bias slightly (See AR-Figure 6). This implies that the results are also insensitive to the heavy-tail quality of the time uncertainties. And our choice of distributions is a conservative one, making our conclusions even stronger. We present the summary of these arguments in the Supplementary Methods Section 2.b.

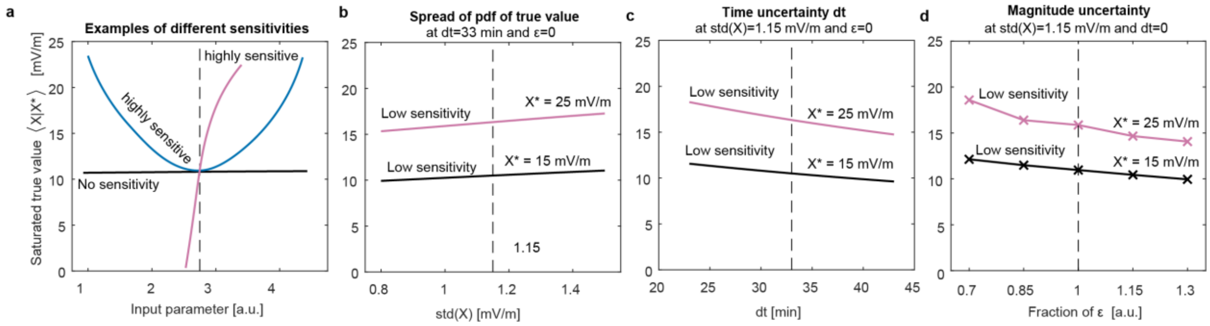

AR-Figure 5| Sensitivity analysis of regression bias with variation in input parameters. a, Example plot showing the difference between highly sensitive input and input with no sensitivity. **b,** Low sensitivity of regression bias to changes in the input distribution of X (pdf of X), when time uncertainty is dt=33 min and ϵ=0. This is calculated from the analytical derivation. **c,** Low sensitivity of regression bias to changes in time uncertainty, when $\sigma(X)$=1.15 mV/m and ϵ=0. This is calculated from the analytical derivation. **d,** Low sensitivity of regression bias to changes in the magnitude uncertainty, when $\sigma(X)$=1.15 mV/m and ϵ=0. Here the sensitivity curve for X^*=25 mV/m is more variable due to lower samples at 25 mV/m and the result is calculated using the Monte-Carlo simulation rather than the analytical equations like for panels b and c.

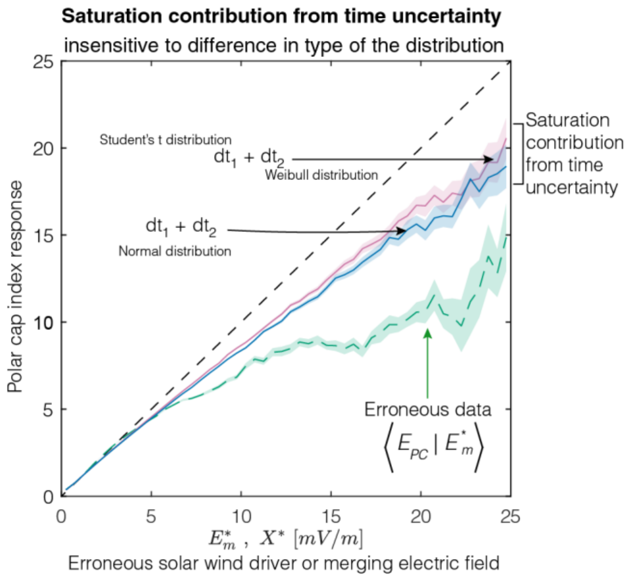

AR-Figure 6| Sensitivity analysis with the shape of the time uncertainty distributions. Regression bias contribution from time uncertainties estimated in the manuscript (estimated to be a heavy-tail distribution) is similar to and slightly lesser than when the shape of the time uncertainty distribution is assumed to be a Normal distribution with an equivalent standard deviation of the time uncertainty. The uncertainty distribution we have estimated in our work is more conservative than a standard Gaussian assumption of the uncertainty.

Revision:
12. Added the quantitative values of the sensitivity of the regression bias to inputs of the error model and note that the bias is robust to changes in inputs. "Using the Monte Carlo simulation, we estimate that a 30% change in our estimated magnitude uncertainty will lead to only a ~12.18% change in the regression bias, with all other parameters held constant and dt=0. Using the analytical equation [1.21] in Supplementary Methods 2.c, we estimate that a 30% change in our estimated time uncertainty will lead to only a ~9.65% change in the regression bias, with all other parameters held constant and $\epsilon = 0$. Similarly, we estimate that a 30% change in our estimated standard deviation of X will lead to only a ~5.9% change in the regression bias given dt=33 minutes and $\epsilon = 0$. These numbers show that the regression bias predicted by the error model is insensitive to a 30% error in the model inputs, especially since the sensitivity is less than the minimum relative magnitude error of ~30% (See Figure 2c). Therefore, for a change in the input, we see a much smaller change in the error model's output. In other words, the error model is robust to change in the estimated input parameters. Note all these values are calculated for regression bias observed in X for a given X*=25mV/m, as it is the most extreme and rare measurement and has the maximum sensitivity of all the measurements."(Line 1188-1200)

Revision:
13. Added lines noting the insensitivity of the results to the heavy-tail nature of the distribution of time uncertainty.
"The regression bias caused by our estimated Student's t-distribution is slightly lower than the equivalent normal distribution with ~8 minutes 1σ standard deviation (i.e., it is a conservative estimate). Recently, Tasnim et al.[59] estimated the propagation delay using a combination of cross-correlation and validation methods, reporting a ~18-minute 2σ standard deviation in propagation delay uncertainty, consistent with our estimate." (Line 1170-1175)
"The total regression bias caused by this distribution is again slightly lower than that of the equivalent normal distribution with a ~25-minute standard deviation, making it a conservative estimate. In other words, using a normal distribution for propagation time uncertainties would yield a similar regression bias as seen in our results, implying the results are insensitive to the heaviness of the distribution's tail."(Line 1183-1187)

Hence, we believe that our result that available data does not show evidence for saturation of geomagnetic response is robust and has the following evidence to substantiate it:

1. Non-zero random error in solar wind driver estimated from measurements and previous results show a regression bias that matches the apparent saturation in data.
2. The second (and higher) order statistics implied by the error model are validated by the second (and higher) order statistics in data.
3. Correcting the solar wind driver measurements for the estimated regression bias from uncertainties reveals a linear relationship between solar wind driver and polar cap index.
4. The same correction also reveals a linear relation between solar wind driver and a different measure of geomagnetic response: westward auroral electrojet index.
5. An analytical derivation proving the regression bias is generated by zero-mean random error supports the existence of the effect, and its independence from the sample of data used for the analysis.
6. A sensitivity analysis derived from the above analytical derivation and monte-Carlo simulation shows that the regression bias estimated by the error model is robust to changes in the input parameters in the error model: such as the spread of the true distribution-$\sigma(X)$, propagation time uncertainty- dt, magnitude uncertainty-ϵ.

List of changes from Revision v1 to Revision v2

(Line numbers mentioned in this document correspond to **Markup Revision File** and **Markup Supplementary File**)

Revisions based on reviewer 3 comments:

1. The error model is validated against data and robust to changes in the inputs (See Supplementary Methods 2.b and Extended Data Fig. 3).). (Line 164-165)

2. We add an additional reference to the latest cross-correlation study to estimate the uncertainty of dt_1 in the Methods section: "Based on their results and consistent with other studies[59], we assume dt_1 to be an independent random process with a pdf of a Student's t-distribution with shape factor 1.3, mean 0, and standard deviation ~8 minutes (See Extended Data Fig. 2c and Supplementary Methods 2.bExtended Data).)." (Line 552-555)

3. We point to references that support the usage in literature of a constant propagation delay (t_2) "The delay in this propagation is on average 20 minutes~17 minutes[14] but can vary from -5 to 50 minutes[50]. Historically, researchers have used a constant propagation delay (t_2) of about ~20 minutes10-30 minutes[27,32]"(Line 560-562)

4. Supplementary Methods summarizes the full computer code used. Additionally, we showHere we also discuss a sensitivity analysis of the non-linear regression bias, which increases with increasing uncertainty in the time of propagation (See c),, and with increasing uncertainty in the magnitude uncertainty $\epsilon\epsilon$ (See Extended Data Fig. 4). The analysis shows that output of the error model is robust to the input uncertainties and variance of input X. Supplementary Methods 2.d summarizes the full computer code used. (Line 614-619)

5. For low values of the temporal uncertainty ratio $\frac{\sigma_\delta}{k} < \sim 0.1$, the regression bias is nearly linear. (Line 660-661)

6. Added new reference: 59.59. Tasnim, S. *et al.* Estimation and assessment of solar wind propagation time from the Lagrange point L1 to Earth's bow shock. *Frontiers in Astronomy and Space Sciences* **12**, 1675769 (2025).

7. In the Extended Data Fig. 5 (New Extended Data Fig. 6.) we change the figure caption as follows: "**b,** The saturation limit of different studies followpresented here follows an increasing trend (linear regression) with the year of study, (Pearson correlation coefficient ~0.61), with the latest studies including higher number of data samples." (Line 409-410)

8. Added paragraph in Supplementary Methods 2b pointing out that our estimates of time and magnitude uncertainties are conservative "As time and magnitude uncertainty increase, the regression bias also increases. A lower uncertainty than estimated by us will result in a decrease in regression bias. Therefore, a conservative estimate of both time and magnitude uncertainty is made by us to present a defensible case for our result." (Line 1157-1159)

9. We clarify the only assumption made in estimating the magnitude uncertainty is at high values of the sheath electric field, when we have less than 10 points available per bin, we use the uncertainties

calculated from simultaneous L1 and solar wind measurements upstream of the bow shock. This is therefore a conservative estimate of the magnitude uncertainty. "For magnitude uncertainty, when $E_m^{sh} > 12$ mV/m, we used uncertainties from simultaneous L1 measurements and solar wind values upstream of the bow shock, adopting a conservative estimate of the uncertainty (~30% relative error). For simultaneous magnetosheath measurements, the sample size is small, with fewer than 10 data points per bin, suggesting no extreme geomagnetic storms fall within the constraints of simultaneous L1 and sheath measurements near the subsolar point." (Line 1160-1165)

10. We justify the choice of dt_1 distribution and add additional references. We also point out that dt_1 is estimated by using cross-correlation analysis of data. "Similarly, for the time uncertainty dt_1, we use a standard deviation of ~8 minutes and a Student's t-distribution with shape factor 1.3 to simulate the narrow but heavy-tail distribution seen in Case & Wild's results[58]. Case & Wild estimate propagation delays directly by cross-correlating solar wind measurements from ACE and Cluster satellites, and compare these calculated delays with propagation delay estimates from the OMNI database we use. The regression bias caused by our estimated Student's t-distribution is slightly lower than the equivalent normal distribution with ~8 minutes 1σ standard deviation (i.e., it is a conservative estimate). Recently, Tasnim et al.[59] estimated the propagation delay using a combination of cross-correlation and validation methods, reporting a ~18-minute 2σ standard deviation in propagation delay uncertainty, consistent with our estimate." (Line 1166-1175)

11. We justify the choice of dt_2 distribution, and not that it is also estimated using cross-correlation analysis from data. "For the time uncertainty dt_2, we use a standard deviation of ~25 minutes, consistent with the results of Milan et al.[14] and Stauning[50]. Milan et al.[14] derive the propagation delay to the ionosphere using cross-correlation of field-aligned currents and solar wind B_z. They find a time-lag distribution peaking at ~17 minutes with a broad peak ranging from 10-30 minutes. Stauning's[50] cross-correlation of PCN index with the solar wind merging electric field just upstream of the bow shock shows propagation delays from -5 to 50 minutes with an average of 20 minutes. We estimate the probability distribution of dt_2 as a Weibull distribution (shape factor 1.3) to capture the broad spread and ensure a positive total delay. The total regression bias caused by this distribution is again slightly lower than that of the equivalent normal distribution with a ~25-minute standard deviation, making it a conservative estimate. In other words, using a normal distribution for propagation time uncertainties would yield a similar regression bias as seen in our results, implying the results are insensitive to the heaviness of the distribution's tail." (Line 1176-1187)

12. Added the quantitative values of the sensitivity of the regression bias to inputs of the error model and note that the bias is robust to changes in inputs. "Using the Monte Carlo simulation, we estimate that a 30% change in our estimated magnitude uncertainty will lead to only a ~12.18% change in the regression bias, with all other parameters held constant and dt=0. Using the analytical equation [1.21] in Supplementary Methods 2.c, we estimate that a 30% change in our estimated time uncertainty will lead to only a ~9.65% change in the regression bias, with all other parameters held constant and $\epsilon = 0$. Similarly, we estimate that a 30% change in our estimated standard deviation of X will lead to only a ~5.9% change in the regression bias given dt=33 minutes and $\epsilon = 0$. These numbers show that the regression bias predicted by the error model is insensitive to a 30% error in the model inputs, especially since the sensitivity is less than the minimum relative magnitude error of ~30% (See Figure 2c). Therefore, for a change in the input, we see a much smaller change in the error model's output. In other words, the error model is robust to change in the estimated input

parameters. Note all these values are calculated for regression bias observed in X for a given X*=25mV/m, as it is the most extreme and rare measurement and has the maximum sensitivity of all the measurements."(Line 1188-1200)

13. Added lines noting the insensitivity of the results to the heavy-tail nature of the distribution of time uncertainty.
"The regression bias caused by our estimated Student's t-distribution is slightly lower than the equivalent normal distribution with ~8 minutes 1σ standard deviation (i.e., it is a conservative estimate). Recently, Tasnim et al.[59] estimated the propagation delay using a combination of cross-correlation and validation methods, reporting a ~18-minute 2σ standard deviation in propagation delay uncertainty, consistent with our estimate." (Line 1170-1175)
"The total regression bias caused by this distribution is again slightly lower than that of the equivalent normal distribution with a ~25-minute standard deviation, making it a conservative estimate. In other words, using a normal distribution for propagation time uncertainties would yield a similar regression bias as seen in our results, implying the results are insensitive to the heaviness of the distribution's tail."(Line 1183-1187)

Editorial Changes:
Reduced the number of words from ~**2935** to ~**2899**. Number of figures reduces from 4 to 3. Total pages within **5 pages** of Nature Article format.

Add references to Extended Data items in the Main Text:
14. (See Extended Data Table 2. for description of symbols). (Line 54)
15. (See Figure 1b and Extended Data Fig. 6green curve).). (Line 59-60)
16. (See Extended Data Table 1Extended Data) (Line 62)
17. For instance, it manifests commonly as 1) a regression bias in the relation inferred between two measurements[16](See Extended Data Fig. 1b, and Extended Data Fig. 5), (Line 113-114)
18. (See Methods[18] and Extended Data Fig. 2). (Line 143-144)
19. The error model is validated against data and robust to changes in the inputs (See Supplementary Methods 2.b and Extended Data Fig. 3).). (Line 164-165)

Reduce the number of words:
20. The calculation of the uncertainty is detailed in the . dc presents the relative uncertainty $(\epsilon(X)/X)$ calculated from measurements with respect to the true shocked solar wind driver in the magnetosheath $(X$), and). It is at least 30% or greater. This uncertainty %, and is a heteroskedastic error as it variesi.e., varying with the magnitude of the true value (X) [19]. (Line 144-147)
21. the likely true value for a given measurement will haveacquires a non-linear bias that mimicsmimicking a *saturation* of X with increasing X^*(Extended Data Fig. 1c(c). c shows). We prove analytically that such a non-linear bias can also increaseincreases with increasing ratio of δ/k , a dimensionless measure of uncertainty in time (See Supplementary Discussion 1.f, Extended Data Fig. 4c, Supplementary Methods 2.c). As a result, once). Incorporating the calculated uncertainty values are workeduncertainties into the error model, andwith X is set to have a log-normal distribution with an autocorrelation coefficient of ~100 minutes observed in solar wind measurements, the Monte-Carlo error model solves for X^* and predicts an appearance of saturation of X given X^* (See Figure 2b). This saturated curve, and the conditional probability distribution of

X given X^*, matches surprisingly well with the apparent saturation ~~that appears~~ in the data (See Figure 2a). (Line 151-160)

22. As shown, once we calculate uncertainties and develop an error model, we can derive the non-linear bias ~~caused by the independent variable~~ in the regression analysis. ~~The bias is the consistent deviation between the measurement and the likely true value estimated from the error model $b = X^* - \langle X|X^* \rangle$. Therefore,~~ We can subtract this bias $b = X^* - \langle X|X^* \rangle$ from the erroneous independent variable~~, and one method to do this is~~ using "regression calibration"[18,19] (See Methods~~.~~). The calibrated variable X^c is simply $X^* - b$~~. By correcting this bias of~~, and it only requires the error model output $\langle X|X^* \rangle$. Applying this bias correction to the solar wind driver ($E_m^c = E_m^* - b$~~), its relation with the cross-polar cap index becomes~~) reveals a surprisingly linear relationship with the cross-polar cap index up to 15 mV/m~~. a shows the saturating green curve formed by using the erroneous data transforms to the linear purple curve after applying this regression calibration. This calibration uses as input only $\langle X|X^* \rangle$ from the error model. Hence the linear relationship between E_m^c and PCI was discovered~~ – without assuming any ~~assumptions of~~ linearity or non-linearity in the underlying relation between E_m^{sh} and PCI, ~~and~~or their error model counterparts. Figure 3~~Therefore,~~a shows the saturating green curve from erroneous data transforming into the linear purple curve after regression calibration~~ of the solar wind measurements~~. This demonstrates that the true geomagnetic response is linear, and that the apparent saturation of the geomagnetic response ~~is a result of~~results from uncertainty in the solar wind driver and the regression to the mean effect. ~~Note that~~ Beyond 15 mV/m, ~~the~~insufficient data ~~available is not significant enough to conclude~~prevents conclusions about the shape of the relation between the driver and response. (Line 191-208)

Reordering figures to shrink the paper size:

23. Figure 1 has been made smaller and made further in alignment with Nature format.

24. Figure 2 has been moved to Extended Data Fig. 1

25. Figure 3c has been moved to Extended Data Fig. 4c; and Figure 3 reduced to a 3 panel figure, and relabeled to Figure 2.

26. Figure 4 has been reduced in size, and relabeled to Figure 3.

27. Extended Data Fig. 1 has been changed to Extended Data Fig. 2. A correction was made to the labelling/typo in panel c.

28. Extended Data Fig. 2 has been changed to Extended Data Fig. 3.

29. Extended Data Fig. 3 is now Extended Data Fig. 5.

30. Extended Data Fig. 4 now includes a new panel c, which was previously Figure 3c.

31. Extended Data Fig. 5 is now Extended Data Fig. 6.

Improvements in the wording:

32. ~~On the other hand~~In contrast, the unbiased random error ~~frequently~~often consists of these random fluctuations and is the random deviation of the measurements from the true value. (Line 84-85)

33. Physical theories in space science generally explain average changes ~~and~~, not random fluctuations. (Line 86)

34. The Weibull distribution keeps the total delay $t_2 + dt_2$ positive and captures the broad spread in the propagation ~~delay documented by Stauning~~[49]delay[14,49,50]. (Line 566-567)

35. E_m^* can differ from the shocked solar wind driver ~~of~~in the ~~magnetoshere~~magnetosheath E_m^{sh} in two ways. (Line 484-485)

36. All methods that use ~~infinite~~sufficient samples will never know of the undersampling bias. (Line 948)

37. There are five vantage points from which we can explain the regression to the mean effect. (Line 999)

38. This is consistent with our estimate of magnitude uncertainty in Figure 2 (Line 1227-1228)

References

1. Viel JF, Pobel D, Carre A. Incidence of leukaemia in young people around the La Hague nuclear waste reprocessing plant: a sensitivity analysis. *Stat Med.* **14**(21–22):2459–2472. (1995) https://doi.org/10.1002/sim.4780142114.

2. Cumming, G. & Fidler, F. Confidence Intervals. *Zeitschrift für Psychologie / Journal of Psychology* **217**, 15–26 (2009).

3. Hoekstra, R., Morey, R.D., Rouder, J.N. *et al.* Robust misinterpretation of confidence intervals. *Psychon Bull Rev* **21**, 1157–1164 (2014). https://doi.org/10.3758/s13423-013-0572-3

4. Reiff, P. H., Spiro, R. W. & Hill, T. W. Dependence of polar cap potential drop on interplanetary parameters. *J. Geophys. Res.* **86**, 7639–7648 (1981).

5. Hill, T. W., Dessler, A. J. & Wolf, R. A. Mercury and Mars: The role of ionospheric conductivity in the acceleration of magnetospheric particles. *Geophys. Res. Lett.* **3**, 429–432 (1976).

Response to Referees 3

To the Editor and Referees:

The following is our response to the reviewer's comments. We label the referee's comment as **RCv3** and label our response **ARv3** (Authors' Response).

A list of revisions is provided under "List of changes from Revision v2 to Revision v3 ". These changes are also provided below next to the label Revision, when a revision is necessary. The line numbers and section numbers provided in this letter are associated with the **Marked-up revision file** and **Marked-up supplementary file**, which is the version of the revised paper with markup including the editorial changes that we have made to shorten the main text further. Line numbers mentioned in this document correspond to **Markup Main Article file (MA)** and **Supplementary File (SF)**.

Referee 3

Referee #3 (Remarks to the Author):

The authors have substantially addressed my comments with the inclusion of the additional discussion regarding the estimates of dt1 and dt2, as well as the additional support from the figures included in the response (Figure AR-6 especially). The revisions to the text associated with these responses has provided good additional support. I have a few remaining comments below that I would like the editor and/or authors to consider that I believe would strengthen this work.

RCv3 3.1: I would still caution against overly strong statements throughout the work that are not fully supported. For example, the abstract states that "we demonstrate that the saturation is a manifestation of the regression to the mean effect arising from random uncertainty in the timing and magnitude of solar wind measurements" while on page 37 the authors say "the regression to the mean effect is the cause for the saturation of the polar cap index with solar wind driver value measured at L1". The authors have demonstrated that an apparent saturation can be explained by this effect, and with the current revision that the expected degree of apparent saturation is close to that observed and is reasonably robust to the assumptions, not that the saturation (i.e., all observed saturation) explicitly is a manifestation of this effect.

ARv3 3.1: We agree with the reviewer that 'all observed saturation' is not a result of the regression to the mean effect. Only the saturation observed due to uncertainty in the solar wind driver, is a result of the regression to the mean effect. In order to avoid misunderstanding in the summary paragraph, we have modified the following line.

Revision:
1. Line MA 20-22: "Here, we demonstrate that ~~the~~this saturation is a manifestation of the *regression to the mean* effect arising from random uncertainty in the timing and magnitude of solar wind measurements."

RCv3 3.2: Regarding the authors' assertion that the model is not additive (ARv2 3.0.2.1): As written the model is an additive model (X* = X + e, or X* = X(t+dt1+dt2) + e), and this remains true if the systematic terms and error term do not interact, even if those terms are nonlinear. A heteroscedastic error term does not necessarily imply that the model is not additive. If the underlying model is not additive then the presentation masks the interaction terms and the interaction should be made explicit so that the reader does not see an additive model.

ARv3 3.2: As discussed in Carroll et al., 2006, Section 4.5.2, an error model of the form **X*=XU** is a multiplicative error model. If this model were to be modified as **X* = X(1+U)**, it would still be a multiplicative error model. This implies **X* = X + XU**. And if **e = XU**, then, **X* = X + e**, it is still a multiplicative error model. Finally, if **e** is further modified to **e = f(X)U**, it is clear it is not an additive error model, instead it is a combination of the classical error model and multiplicative error model. It can be simply stated as a non-linear error model. This is why our error model cannot be solved by linearizing the error terms like most simple additive/linear error models and instead we need to use a Monte-Carlo method to solve them. To emphasize we carry out the following modification.

Revision:
2. In Line MA 599-600 we modify the sentence as "We validate ~~the~~this non-linear error model by comparing the second order statistics of the error model parameters with that of their counterparts in data."

Carroll, R. J., Ruppert, D., Stefanski, L. A. & Crainiceanu, C. M. Measurement error in nonlinear models: A modern perspective, second edition. *Measurement Error in Nonlinear Models: A Modern Perspective, Second Edition* 1–455 (2006).

RCv3 3.3: While considering this it also occurred to me that the model could be phrased as an example of Jensen's inequality, where near a peak the variation is locally concave and thus, due to uncertainty, the measured value would be biased downward, and vice versa for troughs. This is then the regression to the mean described (although without reference to Jensen's inequality) and implies that the error is dependent on the convexity/concavity (second derivative) of the data over any given interval. This would also provide a simple conceptual explanation for the results of Boyle et al., since the convexity/concavity of each interval studied should be net close to zero and thus the bias will be close to zero. Providing a conceptual explanation for the Boyle result within the same framework as the explanation for apparent saturation increases the support for the work.

ARv3 3.3: In statistics textbooks (e.g. Pishro-Nik, 2014) Jensen's inequality is described as follows:

As variance of every random variable is a positive value: $Var(X) = \langle X^2 \rangle - \langle X \rangle^2 \geq 0$. Hence $\langle X^2 \rangle \geq \langle X \rangle^2$. Given this result, if we define a function $f(x)$ which is convex i.e., a line segment between two points on the function lies above the function, then we can write the above inequality as $\langle f(X) \rangle \geq f(\langle X \rangle)$. The inequality arises because of the fact that, for a convex function $f : I \rightarrow R$, and $x_1, x_2, \ldots, x_n$ in I and nonnegative real numbers α_i such that $\alpha_1 + \alpha_2 + \cdots + \alpha_n = 1$, we have $f(\alpha_1 x_1 + \alpha_2 x_2 + \cdots + \alpha_n x_n) \leq \alpha_1 f(x_1) + \alpha_2 f(x_2) + \cdots + \alpha_n f(x_n)$. Now if X is a discrete random variable with n possible values $x_1, x_2, \ldots, x_n$, we can choose $\alpha_i = P(X = x_i) = P_X(x_i)$. Then the above equation becomes $f(\langle X \rangle) \leq \langle f(X) \rangle$, the statement of Jensen's inequality. Here it is very clear that the function f is entirely independent and different from the form of the probability density function of X i.e., P_X. In the reviewer's statement "the model could be phrased as an example of Jensen's inequality, where near a peak the variation is locally concave and thus, due to uncertainty, the measured value

would be biased downward", it is implied what is concave or convex is the probability density functions i.e., P_X. Hence Jensen's inequality is inapplicable here. Furthermore, the regression bias we are estimating is in the conditional expectation of the true value given the uncertain measurement $\langle X|X^* \rangle$, and an error model that relates the random variables $X^* = X(t + dt) + \epsilon$, where $X^*, X, dt,$ and ϵ, are all random variables. Neither in the error model or the regression bias do we estimate the expectation of a function of a random variable i.e., $\langle f(X) \rangle$. This is further demonstration that **our error model cannot be phrased as an example of Jensen's inequality.** As a result, we do not see Jensen's inequality providing any explanation for the results in Boyle et al., 1997.

H. Pishro-Nik, "Introduction to probability, statistics, and random processes", available at https://www.probabilitycourse.com, Kappa Research LLC, 2014.

Boyle, C. B., Reiff, P. H. & Hairston, M. R. Empirical polar cap potentials. J. Geophys. Res. Space Phys. 102, 111–125 (1997).

List of changes from Revision v2 to Revision v3

(Line numbers mentioned in this document correspond to **Markup Main Article file (MA)** and **Supplementary File (SF)**)

Revisions based on reviewer 3 comments:

1. (Line MA 20-22): "Here, we demonstrate that ~~the~~this saturation is a manifestation of the *regression to the mean* effect arising from random uncertainty in the timing and magnitude of solar wind measurements."
2. In Line MA 599-600 we modify the sentence as "We validate ~~the~~this non-linear error model by comparing the second order statistics of the error model parameters with that of their counterparts in data."

Editorial Changes:

Reduced the number of words of Main Text from ~**2899** to ~**2579**.

3. Modified the title according to editor's suggestion: "**Title: Regression to the Mean can Explain Saturation of ~~Extreme~~ Geomagnetic Storms**"
4. We have removed repetitive content from Main Text, Methods, and Supplementary Files, and allowed the details to be present in the Supplementary and Methods, while removing it from the Main Text. Nearly all the changes in Main Text seen in the Markup document are these removals.
5. All Word Equations in the Main Text and Methods have been converted into regular Font and Symbols.
6. Moved the Data and Code Availability to the end of the Main Text references.
7. Rewrote the Author Contributions Statement and Funding Statement to match Nature's style.
8. Added a Disclaimer from Co-author Banafsheh Ferdousi's current institution: "Disclaimer: Approved for public release; distribution is unlimited. Public Affairs release approval # AFRL20262399. The views expressed are those of the author and do not necessarily reflect the official policy or position of the Department of the Air Force, the Department of Defense, or the U.S. government." (Line MA 868-870)
9. Added DOI to the Reference 9: "Borovsky, J. E., Lavraud, B. & Kuznetsova, M. M. Polar cap potential saturation, dayside reconnection, and changes to the magnetosphere. *J. Geophys. Res. Space Phys.* **114**, ~~n/a-n/a~~doi:10.1029/2009JA014058 (2009)."

Reduce the number of words in Supplementary by removing content already states in the Main Text or Methods

10. (Line SF 34-28): " Through the years, as more measurements of rare and extreme solar wind driving accumulated ~~through the years, surprisingly~~, the correlation between the geomagnetic response and the solar wind driver began to deviate from the linear relation. There appeared to be an upper limit to the cross-polar cap potential ~~beyond which~~with increasing ~~solar wind~~ driving ~~led to no increase in the potential~~ (See Figure 1b green curve)."
11. (Line SF 39-40): "The new inference that the cross-polar cap potential saturates with increasing solar wind driving ~~from later measurements~~ led to"
12. (Line SF 51-53): "In this paper, we present a radically different proposition. We argue that there is no statistical evidence for the saturation ~~of geomagnetic response to strong solar wind driving~~, and its appearance in measurements is a result of uncertainty in ~~solar wind~~the driver ~~measurements~~."

13. (Line SF 69-70): "~~A popular technique to find the relations between two or more physical processes is regression analysis of their measurements.~~ The essence of regression analysis is to find the average of the"

Improvements in the wording/ Correcting Typos:

14. (Line SF 249-252): "The ~~precondition~~preconditions of this non-linear saturation relation between PCI and E_m^* ~~is~~are: 1) the heteroskedastic nature of the error in E_m^*, 2) the propagation time uncertainty, ~~and~~3) the fact that the solar wind driver is a log-normal process with a particular autocorrelation time constant."
15. Replacing 'standard deviation' of student's t-distribution with 'scale parameter' in Line MA 543-544 and Line SF 340.

Additions

16. Added a reference to a bigger dataset of magnetosheath measurements from which we had used a subset, in Reference 80: Michotte de Welle, B., aunai, . nicolas ., Nguyen, G., Ghisalberti, A., Connor, H., Génot, V., Jeandet, A., & Lavraud, B. (2026). MANGO Dataset : Magnetosphere Atlas from Normalized Geospace Observations [Data set]. *Zenodo*. https://doi.org/10.5281/zenodo.19671706. Cited this in the Data Availability statement Line MF 821-822: "The broader dataset of magnetosheath measurements is published here (https://doi.org/10.5281/zenodo.19671706)[80]."
